# Supplementary figures and images for: Morphological description of a novel synthetic allotetraploid(A1A1G3G3) of Gossypium herbaceum L.and G.nelsonii Fryx. suitable for disease-resistant breeding applications
Source: PLoS One. 2020 Dec 3;15(12):e0242620. doi: 10.1371/journal.pone.0242620 (PMC7714114; doi:10.1371/journal.pone.0242620)

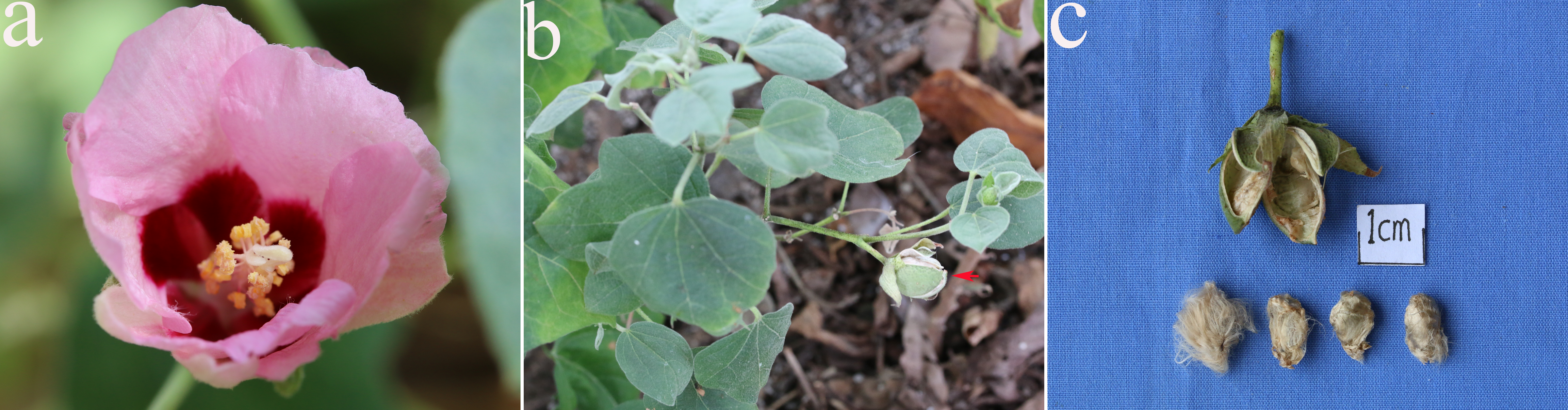

Supplement: S1 Fig — (a) Tetraploid plants with a large pink flower accompanied by yellow pollen. (b) The growth process of the oval boll. (c) Four seeds with brown fibers. (TIF) [file pone.0242620.s001.tif]

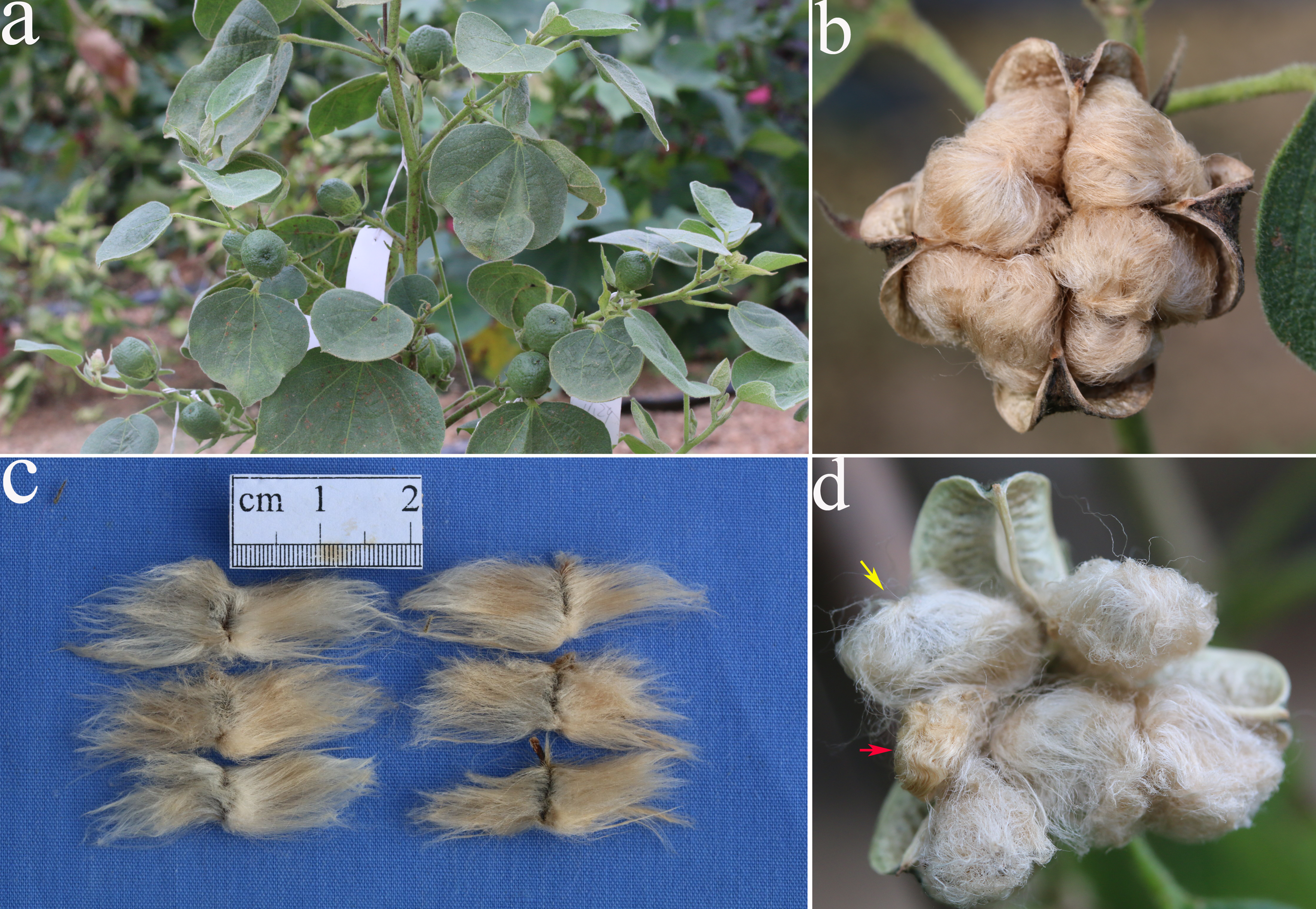

Supplement: S2 Fig — (a) Multiple bolls of S1 on multiple fruit branches with the sunken navel of the boll tip showing an ergonomic shape with strong bolls. (b) Four-chambered boll with brown fibers. (c) The brown fibers with fiber length of 13 mm. (d) Three-chambered boll with light green and brown cotton fibers. (TIF) [file pone.0242620.s002.tif]

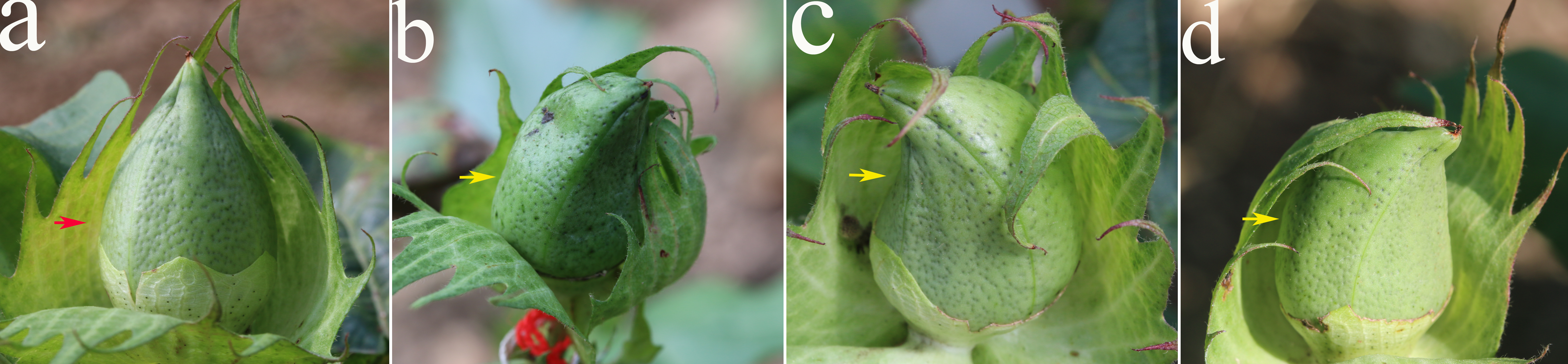

Supplement: S3 Fig — (a)The normal conical boll of TM-1 shown by the red arrow. The abnormally conical boll of F1 on the right (b) and on the left (c). (d) The deformed hybrid boll of F1 indicated by yellow arrows. (TIF) [file pone.0242620.s003.tif]
