# Supplementary material for: Morphological description of a novel synthetic allotetraploid(A1A1G3G3) of Gossypium herbaceum L.and G.nelsonii Fryx. suitable for disease-resistant breeding applications
Source: PLoS One. 2020 Dec 3;15(12):e0242620. doi: 10.1371/journal.pone.0242620 (PMC7714114; doi:10.1371/journal.pone.0242620)
Supplement: Attachment — Submitted filename: PONE-D-20-02970_reviewer (1) -1.pdf [file pone.0242620.s004.pdf]

PLOS ONE

Morphological and genetic description of a novel synthetic allotetraploid of Gossypium herbaceum L. and Gossypium nelsonii Fryx.

--Manuscript Draft--

**Manuscript Number:**	PONE-D-20-02970
**Article Type:**	Research Article
**Full Title:**	Morphological and genetic description of a novel synthetic allotetraploid of Gossypium herbaceum L. and Gossypium nelsonii Fryx.
**Short Title:**	A novel synthetic allotetraploid of Gossypium herbaceum L. and Gossypium nelsonii Fryx.
**Corresponding Author:**	Xiaomin Yin<br>Chinese academy of tropical agricultural sciences<br>Haikou, CHINA
**Keywords:**	Genetic evolution; Gossypium; Interspecific hybridisation; Synthetic allotetraploid
**Abstract:**	The cotton pathogen Verticillium dahliae Kleb. infects cotton during the planting process and during its growth and seriously reduces yields. While wild cotton exhibits high levels of resistance to this pathogen, cultivated upland cotton does not. In this study, we created a new germplasm of cotton through remote hybridisation during our search for a resistant source of wild cotton. G. herbaceum L. (♀) was crossed with G. nelsonii Fryx. (♂) to produce an interspecies F 1 hybrid. Colchicine was used to double its chromosomal content, leading to the successful generation of a synthetic S 1 tetraploid. The newly created S 1 tetraploid was characterised by morphological analysis, polymorphism detection, ploidy analysis, and microscopic observation of chromosome numbers. The S 1 tetraploid showed morphological variations in its flowers, cotton bolls, and fibres and was partially resistant to Verticillium dahliae Kleb . The S 1 tetraploid was hybridised with upland cotton to produce two types of cotton with different characteristics. This successful crossbreeding of S 1 provides direct evidence that interspecies hybridisation can facilitate the development of new types of cotton . This newly synthesised tetraploid cotton species is a valuable resource for cotton resistance breeding and for the enrichment of cotton genetic resources.
**Order of Authors:**	Di Chen
	Bingqiang Xu
	Shun Song
	Yingdui He
	Lixia Wang
	Yu Ge
	Rulin Zhan
	Xiaomin Yin
**Additional Information:**	
**Question**	**Response**
**Financial Disclosure**<br><br>Enter a financial disclosure statement that describes the sources of funding for the work included in this submission. Review the submission guidelines for detailed requirements. View published research articles from *PLOS ONE* for specific examples.	YES - Specify the role(s) played.This research work was financially supported by the National Natural Science Foundation of China (No. 31704178) and Central Public-interest Scientific institution Basal Research Fund for Chinese Academy of Tropical Agricultural Sciences(No.1630092017004).

Powered by Editorial Manager® and ProduXion Manager® from Aries Systems Corporation

This statement is required for submission and **will appear in the published article** if the submission is accepted. Please make sure it is accurate.<br><br>**Unfunded studies**<br>Enter: *The author(s) received no specific funding for this work.*<br><br>**Funded studies**<br>Enter a statement with the following details:<br>• Initials of the authors who received each award<br>• Grant numbers awarded to each author<br>• The full name of each funder<br>• URL of each funder website<br>• Did the sponsors or funders play any role in the study design, data collection and analysis, decision to publish, or preparation of the manuscript?<br>• **NO** - Include this sentence at the end of your statement: *The funders had no role in study design, data collection and analysis, decision to publish, or preparation of the manuscript.*<br>• **YES** - Specify the role(s) played.<br><br>* typeset	
**Competing Interests**<br><br>Use the instructions below to enter a competing interest statement for this submission. On behalf of all authors, disclose any competing interests that could be perceived to bias this work—acknowledging all financial support and any other relevant financial or non-financial competing interests.<br><br>This statement **will appear in the published article** if the submission is accepted. Please make sure it is accurate. View published research articles from *PLOS ONE* for specific examples.	The authors have declared that no competing interests exist.

Powered by Editorial Manager® and ProduXion Manager® from Aries Systems Corporation

**NO authors have competing interests**<br><br>Enter: *The authors have declared that no competing interests exist.*<br><br>**Authors with competing interests**<br><br>Enter competing interest details beginning with this statement:<br><br>*I have read the journal's policy and the authors of this manuscript have the following competing interests: [insert competing interests here]*<br><br>* typeset	
**Ethics Statement**<br><br>Enter an ethics statement for this submission. This statement is required if the study involved:<br><br>• Human participants<br>• Human specimens or tissue<br>• Vertebrate animals or cephalopods<br>• Vertebrate embryos or tissues<br>• Field research<br><br>Write "N/A" if the submission does not require an ethics statement.<br><br>General guidance is provided below. Consult the submission guidelines for detailed instructions. **Make sure that all information entered here is included in the Methods section of the manuscript.**	Haikou Experimental Station, Chinese Academy of Tropical Agricultural Sciences

**Format for specific study types**<br><br>**Human Subject Research (involving human participants and/or tissue)**<br>• Give the name of the institutional review board or ethics committee that approved the study<br>• Include the approval number and/or a statement indicating approval of this research<br>• Indicate the form of consent obtained (written/oral) or the reason that consent was not obtained (e.g. the data were analyzed anonymously)<br><br>**Animal Research (involving vertebrate animals, embryos or tissues)**<br>• Provide the name of the Institutional Animal Care and Use Committee (IACUC) or other relevant ethics board that reviewed the study protocol, and indicate whether they approved this research or granted a formal waiver of ethical approval<br>• Include an approval number if one was obtained<br>• If the study involved *non-human primates*, add *additional details* about animal welfare and steps taken to ameliorate suffering<br>• If anesthesia, euthanasia, or any kind of animal sacrifice is part of the study, include briefly which substances and/or methods were applied<br><br>**Field Research**<br><br>Include the following details if this study involves the collection of plant, animal, or other materials from a natural setting:<br>• Field permit number<br>• Name of the institution or relevant body that granted permission	
**Data Availability**<br><br>Authors are required to make all data underlying the findings described fully available, without restriction, and from the time of publication. PLOS allows rare exceptions to address legal and ethical concerns. See the PLOS Data Policy and FAQ for detailed information.	Yes - all data are fully available without restriction

A Data Availability Statement describing where the data can be found is required at submission. Your answers to this question constitute the Data Availability Statement and **will be published in the article**, if accepted.<br><br>**Important:** Stating 'data available on request from the author' is not sufficient. If your data are only available upon request, select 'No' for the first question and explain your exceptional situation in the text box.<br><br>Do the authors confirm that all data underlying the findings described in their manuscript are fully available without restriction?	
**Describe where the data may be found in full sentences. If you are copying our sample text, replace any instances of XXX with the appropriate details.**<br><br>• If the data are **held or will be held in a public repository**, include URLs, accession numbers or DOIs. If this information will only be available after acceptance, indicate this by ticking the box below. For example: *All XXX files are available from the XXX database (accession number(s) XXX, XXX.)*.<br>• If the data are all contained **within the manuscript and/or Supporting Information files**, enter the following: *All relevant data are within the manuscript and its Supporting Information files.*<br>• If neither of these applies but you are able to provide **details of access elsewhere**, with or without limitations, please do so. For example:<br><br>*Data cannot be shared publicly because of [XXX]. Data are available from the XXX Institutional Data Access / Ethics Committee (contact via XXX) for researchers who meet the criteria for access to confidential data.*<br><br>*The data underlying the results presented in the study are available from (include the name of the third party*	All relevant data are within the manuscript and its Supporting Information files.

and contact information or URL).<br>• This text is appropriate if the data are owned by a third party and authors do not have permission to share the data.<br><br>* typeset	
Additional data availability information:	Tick here if the URLs/accession numbers/DOIs will be available only after acceptance of the manuscript for publication so that we can ensure their inclusion before publication.

Powered by Editorial Manager® and ProduXion Manager® from Aries Systems Corporation

Manuscript Click here to download Manuscript renamed_27fe5.pdf

Morphological and genetic description of a novel synthetic allotetraploid of *Gossypium herbaceum* L. and *Gossypium nelsonii* Fryx.

Di Chen, Bingqiang Xu, Shun Song, Yingdui He, Lixia Wang, Yu Ge, Rulin Zhan*, and Xiaomin Yin*

Haikou Experimental Station, Chinese Academy of Tropical Agricultural Sciences (CATAS), Haikou, China

Correspondence and requests for materials should be addressed to Z.R.L.and Y.X.M. Haikou Experimental Station, Chinese Academy of Tropical Agricultural Sciences (CATAS), Haikou, 66767898, China. Email: yinxm8303@126.com

Abstract

The cotton pathogen *Verticillium dahliae* Kleb. infects cotton during the planting process and during its growth and seriously reduces yields. While wild cotton exhibits high levels of resistance to this pathogen, cultivated upland cotton does not. In this study, we created a new germplasm of cotton through remote hybridisation during our search for a resistant source of wild cotton. *G. herbaceum* L. (♀) was crossed with *G. nelsonii* Fryx. (♂) to produce an interspecies F_1 hybrid. Colchicine was used to double its chromosomal content, leading to the successful generation of a synthetic S_1 tetraploid. The newly created S_1 tetraploid was characterised by morphological analysis, polymorphism detection, ploidy analysis, and microscopic observation of chromosome numbers. The S_1 tetraploid showed morphological variations in its flowers, cotton bolls, and fibres and was partially resistant to *Verticillium dahliae* Kleb. The S_1 tetraploid was hybridised with upland cotton to produce two types of cotton with different characteristics. This successful crossbreeding of S_1 provides direct evidence that interspecies hybridisation can facilitate the development of new types of cotton. This newly synthesised tetraploid cotton species is a valuable resource for cotton resistance breeding and for the enrichment of cotton genetic resources.

Keywords: Genetic evolution, *Gossypium*, Interspecific hybridisation, Synthetic allotetraploid.

Introduction

Cotton (genus, *Gossypium*; family *Malvaceae*) is the most important natural raw material in the textile industry. Verticillium wilt is a disease that causes severe yield losses in cotton growing areas [1]. The pathogen causing this disease is a soil-borne fungus, *Verticillium dahliae* Kleb., which was first reported in Virginia, USA, in 1914 [2]. This disease has now spread to most of cotton growing areas worldwide, and there are no effective prevention and control methods available. Currently cultivated upland cotton lacks resistance to verticillium wilt, whereas wild cotton remains highly resistant to this disease [14]. Thus, this study aimed to identify and isolate the source of resistance in wild cotton by interspecies hybridisation.

Remote hybridisation has long been known to be an important means for studying the evolution of species and for the artificial synthesis of new species. There are 55 species of the genus *Gossypium* worldwide, including 48 diploids ($2n = 2x = 26$) and 7 tetraploids ($2n = 4x = 52$) [3,4]. Over the decades, a number of interspecies hybrids have been successfully produced, including *G. arboreum* L. × *G. raimondii* Ulbr. [5], *G. hirsutum* L. × *G. sturtianum* Willis [6], *G. arboreum* L. × *G. bickii* Prokh. [7], *G. hirsutum* L. × *G. klotzschianum* Anderss. [8], *G. hirsutum* L. × *G. trilobum* Skov. [9], *G. arboreum* L. × *G. anomalum* Wawra & Peyr. [10], *G. hirsutum* L. × *G. anomalum* Wawra & Peyr. [11], and *G. herbaceum* L. × *G. australe* F. Muell. [12]. However, to our knowledge, there are no reports of an allotetraploid ($A_1A_1C_4C_4$) that has been formed from a cross between *G. herbaceum* L. and *G. nelsonii* Fryx.

The cultivated diploid cotton species *G. herbaceum* L. ($2n = 2x = 26$, A_1A_1) originated in Africa, whereas the wild diploid cotton species *G. nelsonii* Fryx. ($2n = 2x = 26$, C_4C_4) originated in Oceania. *G. nelsonii* Fryx. has excellent drought tolerance and is resistant to *Verticillium dahliae* Kleb. Due to interspecies isolation, it has been difficult for the two species to intercross under natural conditions. Thus, in this study, we crossed *G. herbaceum* L. (♀) with *G. nelsonii* Fryx. (♂) to produce an interspecies

F_1 hybrid. We grafted the F_1 hybrid plants to obtain 100 clones and doubled its chromosomal content using colchicine to successfully obtain a synthetic tetraploid, which we designated S_1 (2n = 4x = 52, $A_1A_1C_4C_4$). We characterised this new S_1 cotton germplasm by performing morphological analyses, polymorphism detection, and ploidy analysis (by flow cytometry and microscopic observation of chromosome numbers). The assessment of the disease resistance of the S_1 tetraploid and its application in further hybridisation experiments are also reported.This newly germplasm can enrich the genetic resources of cotton and provide important materials for disease resistance breeding of cotton.

Materials and Methods

Plant materials

The cotton varieties used in this study were *G. herbaceum* L. (♀), *G. nelsonii* Fryx. (♂), Cotton cv. *Jimian11*, and cotton TM-1. The diploid *G. herbaceum* L. of the cultivated species 'Hongxing Caomian' (2n = 2x = 26, A_1A_1) was used as the female parent and the diploid *G. nelsonii* Fryx. (2n = 2x = 26, C_4C_4) was used as the male parent for interspecies hybridisation. Pure *G. herbaceum* L. seeds were obtained by strict self-pollination for 40 generations. Pollen from *G. nelsonii* Fryx. was collected from the China National Wild Cotton Nursery in Sanya, China [13]. Cotton cv. *Jimian11*, the standard susceptibility control for the national identification of cotton varieties in China, was used in experiments to identify new resistant synthetic allotetraploids. Upland cotton TM-1 was the female plant source used for crossbreeding at the final experimental stage of this study.

Interspecies hybridisation

Distant hybridisation was carried out in Sanya, China, in the winter of 2015. The female flowers were removed from *G. herbaceum* L. (♀) and the stigmas covered with a wax tube to avoid cross-pollination during flowering the following day. The following day, pollen from the male *G. nelsonii* Fryx. parent was collected and used to pollinate the female parent between 9 a.m. and 12 noon. Flowers that have been pollinated were marked. More than a month later, the hybrid seeds were harvested from the female parent plant and planted in a nutrient bowl. On April 20, 2016, when

the seedlings were two weeks old, they were transplanted into a field at Sanya, China [13,14].

Clonal propagation and colchicine treatment

Only very few hybrid seeds were obtained by interspecies hybridisation, and the F_1 hybrid was sterile. To provide sufficient experimental materials for chromosomal doubling, the F_1 hybrid generation was cloned by grafting and preserved in Sanya, China on July 15, 2016. Chromosome doubling experiments were conducted under natural field conditions, on selected original or cloned plants with a height of 50 cm. Approximately 2 mm was removed from the apical growth point of these plants while retaining the apical tender branches. Colchicine (0.3%) was dipped into the cotton balls, which were wrapped around the top of the tender branches, and covered with plastic bags to prevent evaporation. After 6 hours of treatment, the plastic bags and cotton balls were removed. Five days later, the leaves were sprayed with a solution of potassium dihydrogen phosphate (at a concentration of 1,000 times), once every 7 days, until new shoots grew. Throughout the experiment, side branches that did not receive both treatments were removed [13]. On October 15, 2016, the treated plants were transferred to seedling pouches to grow under natural field conditions in Sanya, China.

Morphological analyses

The morphological characteristics of parents, hybrids, and tetraploid plants were analysed and recorded as previously described [14]. Information about the stems and leaves, plant height, and plant type during the vegetative growth period was recorded. The shape and colour of the flowers, calyxes, and bracts, the colour of the filaments, anthers and pollen, and the features of the flower base, the shape and colour of fruits in boll stage, and the shape and colour of fibres in cotton boll production stage were also recorded. A Canon digital camera (EOS 70D) set at RF 35mm F1.8 MACRO was used to photograph the various tissues shapes of cotton. Adobe Photoshop 7.0 software from Adobe company was used to edit the morphological images of cotton.

Polymorphism identification by the SSR molecular marker

Plant genomic DNA from the F_1 hybrid, tetraploid S_1 and their parents was extracted according to a previously published method with minor modifications **[**14**]**. Plant DNA was extracted from young leaves near the growth point and genomic DNA was quantitatively and qualitatively analysed by agarose gel electrophoresis. Six SSR primers were selected for PCR amplification (Table 4), which was performed in a DNA Mastercycler (Eppendorf). The PCR reaction components and optimised reaction conditions were based on previous literature **[**13**]**. PCR products were resuspended in double distilled water and analysed by agarose gel electrophoresis. The gels were photographed using a Canon digital camera (EOS 70D) and imaged edited in Adobe Photoshop 7.0 software.

Ploidy identification by flow cytometry

Selected young leaves that are 1 cm long and 1 cm wide were cut into small pieces with a razor blade and mixed with 1 ml of Galbraith buffer (45 mM $MgCl_2$, 30 mM Sodium Citrate, 20 mM 3-(N-morpholino) propanesulfonic acid [MOPS], 0.1% (v/v) Triton X, pH=7 **[**16**]**) before filtering through a 50 micron nylon mesh. The filtrate was centrifuged at 1,000 xg for 5 min to pellet the cells. Propidium iodide solution (200 ml of a 50 mg/l solution) was added to the cells, which were incubated in the dark for 20 min **[**39**]**. DNA content was analysed by flow cytometry (Sysmex-Partec CyFlow® Cube 8) with excitation set at 488 nm. Diploid *G. herbaceum* L. was used as the external reference standard (code: 2C). Samples suspected to be diploid (*G. herbaceum* L. × *G. nelsonii* Fryx. F_1, code: 2C) and those suspected to be tetraploid (*G. herbaceum* L. × *G. nelsonii* Fryx. S_1, code: 4C) were mixed prior to testing. One diploid peak indicated that both tested plants were diploid; whereas the presence of two peaks at a 1:2 ratio indicated the presence of a diploid and a tetraploid plant. Adobe Photoshop 7.0 software was used to edit and process the images detected by flow cytometry.

Microscopic observation of chromosome numbers

When the hybrid began to germinate and the root tip grew to 2 cm long, approximately 5 mm of the tip was cut off between at 9:00 am to 10:00 am, and the apex was immersed into a saturated solution of p-dichlorobenzene at room temperature for 6 h. The treated materials was rinsed twice with distilled water and

immediately fixed in freshly prepared Carnoy's fluid (ethanol: acetic acid at 3:1 v/v [13]). After 24 h, the root tip was rinsed with distilled water and cut into pieces in 1 M of hydrochloride. Two root tips were dispensed in each 1.5 ml centrifuge tube and dissociated at 60℃ in a water bath for 10-15 min. The separated root tips were then stained with Carbol fuchsin solution on the slide, and identified by microscopy. Micrographs of cells and chromosomes were collected using German Leica biological microscope DM2500 and camera system MC170. Adobe Photoshop 7.0 software was used to edit the pictures of cells and chromosomes.

Identification of disease resistance

The Dahlia Vd080 strain that causes cotton fusarium blight was obtained from the Cotton Research Institute of the Chinese Academy of Agricultural Sciences. The pathogen strain was frozen in 25% glycerin (v/v) at -80℃ for long-term preservation, and the strain was activated on potato dextrose agar (PDA) culture medium before inoculation. The pathogen was cultured in PDA at 28℃ for 7 days to obtain the strain culture solution (conidia solution) according to previous reports[18]. The final concentration was adjusted to 10^6 conidia/ml with sterile distilled water. The S_1 allotetraploid was tested using upland Cotton *Jimian11*, which was susceptible to *Verticillium dahliae* Kleb. as controls. Each variety was divided into three treatment groups of thirty plants per group, with six bowls for each . Commercial vermiculite and sun-dried river sand were used as substrates to prepare bottomless paper bowls. Plantlets at the five-leaf stage were supplied, and the roots were inoculated with a spore suspension (10^6 conidia/ml) to induce cotton verticillium wilt. The cotton seedlings were planted under controlled greenhouse conditions to observe the pathogenesis of verticillium wilt. Disease was classified and indexed according to a previously published ranking system as follows[19]: Rank zero: healthy; Rank 1: disease occurred on one or two cotyledons; Rank 2: disease occurred on one true leaf, but terminal bud was healthy; Rank 3: disease occurred on more than two pieces of true leaves, but the terminal bud remained healthy; Rank 4: the entire plant was morbid and/or dead. The incidence and disease index were calculated based on the survey results. All cotton varieties were photographed with a Canon digital camera (EOS 70D) [20]. Microscopic observation and image acquisition of pathogenic bacteria spores and hyphae were carried out using a biological microscope (Leica

DM2500 with an MC170 camera system) and edited in Adobe Photoshop 7.0 software.

Crossbreeding experiments

In the winter of 2017, the hybridisation of the upland cotton TM-1 (genome AADD) and the newly synthesised allotetraploid S_1 ($A_1A_1C_4C_4$) was carried out in Sanya, China. The flowers of upland cotton TM-1 were removed and the stigmas covered with a wax tube to avoid cross-pollination by pollen from other plants. Between 9 a.m. to 12 noon. the following day, allotetraploid S_1 plants were was selected to pollinate the female upland cotton TM-1 and marked. After at least one month, hybrid seeds could be obtained from upland cotton TM-1. The seeds were planted in a nutrient bowl and transplanted to the field two weeks later, on April 20, 2017. All cotton varieties were photographed with a Canon digital camera (EOS 70D) and edited in Adobe Photoshop 7.0 software.

Results

Morphological analysis of the diploid F_1 hybrid and the synthetic S_1 tetraploid

Production of the F_1 hybrid. The breeding of the F_1 hybrid was initiated from artificial hybrid bolls. One boll per plant containing three seeds was obtained. After sowing, all three hybrid plants germinated and developed. The appearance and morphology of the three F_1 hybrids were similar, with broad leaves. On average, each boll from the hybrid plants contained one to three seeds. All three F_1 hybrid plants were infertile at later growth stages and did not bear fruit. This phenomenon was not unexpected and was consistent with the law of distant hybrid infertility, as these plants were the offspring of interspecies hybridisation. Plants presumed to be true hybrids were propagated by grafting. More than 100 plants were grafted to prevent the loss of the interspecies hybrid. The stem apices of grafted plants were treated with 0.3% colchicine for 6 h during the squaring stage. Sixty-one days later, we were surprised to find that one of the grafted plants had produced a large pink flower with abundant yellow pollen (Fig. 1A), which we designated F_1. Five days after the flowers died, an ovate boll grew, ripened, and split, to produce four seeds with brown fibres (Fig. 1B, 1C).

Production of the S_1 tetraploid. We crossed *G. herbaceum* L. (♀) (Fig. 2A)with *G. nelsonii* Fryx. (♂)(Fig. 2B) to produce an interspecies F_1 hybrid.The S_1 tetraploid was produced from seeds of the fertile F_1 plant(Fig. 2C) described above. Four plump seeds were sown, one of which germinated successfully and grew into an adult plant (Fig. 2D). This S_1 plant was shorter than the F_1 parental plant, with thicker leaves, petals, and calyxes compared to its F_1 parent. Importantly, this S_1 tetraploid was fertile and produced a large number of bolls. Therefore, it is likely that the chromosomal content of the newly grown S_1 tetraploid had successfully doubled. In contrast, the F_1 plant produced only one boll, and only once. These observations suggest that successful doubling of the cotton chromosome did not happen very often and that the diploid F_1 might have been successfully transformed into a new derivative by colchicine treatment. The genome of the new synthetic tetraploid S_1 was 2n = 4x = 52, $A_1A_1C_4C_4$.

Plant shape and growth, and flowering. After the plants had developed into adults, they were surveyed. *G. herbaceum* L., F_1, and S_1 had lower branches that were longer than the upper branches, giving the plants a tower-like appearance (Fig. 2A, 2C, 2D). In contrast *G.* nelsonii Fryx. had upper, middle, and lower branches of approximately the same length; these plants looked like long canisters (Fig. 2B). Similar to *G. herbaceum* L., both F_1 and S_1 exhibited prolific growth with extensive monopodial branching (Fig. 2). Flower buds of the F_1 plant appeared 40 days after planting (Fig. 3c). The *G. herbaceum* L, F_1, and S_1 flowered all year round (Fig. 3A, 3C, 3D), but the flowering time of *G. nelsonii* Fryx. was limited, from November in the first year to June in the second year (Fig. 3B). The S_1 flowers had corollas of different shapes, colours, and sizes (Supplementary Figure 1A-H). The shapes were cylindrical, funnel, bell, and semi-closed or closed; sizes were considered big, medium, and small, corresponding to 40, 25, and 15 mm in length, respectively; and the colours included light red, pink, and creamy white (Supplementary Figure 1A-H). The closed pollination in the S_1 generation was inherited from the male parents, because closed pollination often occurred when male parents were at low temperature, whereas it did not occur in the female parents (Supplementary Figure 1H).

Bolls and fibres. The diploid F_1 could not bear bolls because it was sterile. In contrast, the S_1 tetraploid was fertile and showed characteristics of precocious puberty; for

example, the S_1 tetraploid that was sown in the field took 4 days to germinate, which was 2 days earlier than either parent. Sowing to flowering was completed in 40 days in the S_1 tetraploid, which was 5 days and 3 days earlier than that in the female and male parental plants, respectively. Subsequently, flowering to cotton boll opening in the S_1 occurred in 35 days, again, 5 days and 3 days earlier than that in the female and male parental plants, respectively. Therefore, the S_1 tetraploid had a growth cycle of 99 days. The S_1 tetraploid had strong fruit production capacity (Supplementary Figure 2), producing up to 50 bolls per plant after 4 months of growth in the field (Supplementary Figure 2). Cotton bolls of various shapes, including pear-shaped, lemon-shaped, pebbly-shaped, and irregular shapes, were produced (Fig.4C). Some of these shapes were never produced by either parent; in particular, a pear-shaped boll with a sunken navel on its surface (Fig. 4C). These boll characteristics were significantly different from the female parents from which the fruit tips were raised. Additionally, the fruit surface of the S_1 tetraploid was covered with short fuzzy hair, a trait inherited from the male parent (Fig. 4C). The boll of the S_1 tetraploid contained three or four ventricles. In Sanya, China, the S_1 tetraploid bloomed and bore fruit all year round and was highly fertile. In summary, the S_1 tetraploid had strong boll-forming abilities, indicating that the plant has potential for high yield, which is an important agronomic factor that improves the yield of upland cotton.

The S_1 tetraploid produced open and unimpeded cotton bolls, containing up to 16 fertile seeds per boll. Its fibre colour was mainly inherited from the male parent including light brown, brown, and light green, whereas fibres of the female parents were white (Fig.4D,E,F). In the male parent plants, fibres grew upright (Fig. 4E) and were shaped like upright iron needles. In contrast, fibres of female parents grew in a spiral (Fig. 4D) and were shaped like fixed telephone lines. In contrast, the fibres of the S_1 tetraploid showed wavy growth and were shaped like human curls, representing an intermediate phenotype of both parents (Fig. 4F). In all species, hairs grew in the direction of the seed tip. The cotton bolls of the S_1 tetraploid were also diverse and included four-chambered bolls with brown fibres and three-chambered bolls with light green and brown fibres (Supplementary Figure 3A,B). The average fibre length of the S_1 tetraploid was approximately 13 mm, which was longer than those produced by male parents but shorter than those produced in female parents (Supplementary Figure 3C).

Comparisons of the biological traits of F_1 and S_1 with their parental plants. The biological characteristics of the parents, F_1 and S_1 are detailed in Tables 1,2. In the fields of Sanya, China, the female *G. herbaceum* L. parent grows for up to 3 years. After the first year of growth, the plant reaches approximately 80 cm in height (Fig. 2A), and the main stem reaches approximately 1.5 cm in diameter. *G. herbaceum* L. is tower-shaped with short and compact branches. It bears bell-shaped flowers with yellow corollas, pink basal patches, pink filaments, yellowish anthers, yellow pollen, a cup-shaped calyx, heart-shaped bracts that grow separately rather than together, and is broad-leaved. The female *G. herbaceum* L. produces smooth conical bolls that are green and red in colour (Fig. 4A). There is a distinct conical bulge at the top of the boll, and the bolls are 3-chambered with grey-white spiral cotton fibres. The male *G. nelsonii* Fryx. parent also grows for 3 years. After the first year of growth, the plant reaches approximately 200 cm in height (Fig. 2B), and the main stem grows to approximately 2 cm in diameter. *G. nelsonii* Fryx. is cylindrical with short and compact branches, bears tubular-shaped flowers with pale pink corollas, purple basal spots, red filaments and anthers, milky pollen, a wavy calyx, sword-shaped bracts that are detached rather than united, and has ovoid leaf blades. The male *G. nelsonii* Fryx. parent plant produces oval, green cotton bolls with fine hair on the surface. There are no distinctive features on the boll tips (Fig. 4B), and the bolls are 3-chambered with dark brown upright fibres (Fig. 4E).

After the first year of growth, the diploid F_1 plant reached 300 cm in height (Fig.2C) and was taller than either parent plant. Its main stem diameter grew to 3.0 cm, also wider than either parent. It had longer, loosely spaced branches, but was infertile (Tables 1, 2). Most of the biological characteristics of the F_1 plants were inherited from the female *G. herbaceum* L. parent, including a tower-like appearance, trumpet flowers, green main stems, leaves, and petioles, yellow anthers, and pollen. The leaf length and width, detached bracts, and column length were also similar to the female parent. A few traits were inherited from the male *G.nelsonii* Fryx. parent, including pink corollas, light pink filaments, and medium-sized spots on the petal base. Some traits were inherited from both parents and of an intermediate phenotype; these included a serrated calyx, sword-shaped bolls, red spots on the petal base, lobated leaves, and teeth-like bracts. Some features of the F_1 hybrid were not observed in either parent, including production of taller plants, larger main stems, more fuzzy

stems and leaves, long and looser branches, and lobate leaf lobes. Some leaves of the F_1 were broad-leaved, oval, and semi-circular in shape, displaying leaf characteristics of both parents.

After successfully doubling of the growth points of the diploid F_1 hybrids with colchicine, the plants obtained were designated the S_1 tetraploid generation. The S_1 plants had restored fertility and could produce bolls. After the first year of growth, S_1 plants reached 150 cm in height (Fig. 2D), which was an average of both parents. Its main stem diameter reached 2.5 cm, which was larger than either parent, and it produced longer, more loosely spaced branches. S_1 plants showed some biological traits that were distinct from the F_1 hybrid. They were shorter, and their leaves were thicker and greener. The leaves were broad-leaved, ovoid or semi-circular in shape, and the flowers were trumpet-, funnel-shaped, cylindrical, closed, or half closed. The colours of the corollas were pink, light pink, milky white, red, and white, simultaneously displaying characteristics of both parents and the F_1 parents. Some biological characteristics of the S_1 generation were inherited from the female *G. herbaceum* L. parent, and were similar to the F_1 hybrid, namely, tower-like plants, green main stems and petioles, yellow anthers, and pollen. A few traits were inherited from the male *G. nelsonii* Fryx. parent, namely, serrated calyx and green stems and bolls. Some traits were intermediate of both parents, namely plant height, leaf length and width, triangle dentate bracts, ensiform bracts, and light green and brown fibres. Some features of the S_1 generation showed new variations beyond their parental phenotypes, such as boll shape, wavy fibres, the colour and size of the spot on the petal base, fuzzier stems and leaves, longer and looser branches, lobate leaf lobes, and column length. In particular, the bolls of S_1 were pear-shaped, with sunken navels, oval, irregular, or round. The column length were long, medium, or short. The size of the petal base spot was medium or small, and it produced some branches but had no basal plaques.

Polymorphism detection in diploid F_1 and tetraploid S_1 using simple sequence repeat (SSR) molecular markers

To better understand the authenticity of hybrids and their molecular differences compared to their parents, SSR analysis was performed using six SSR primer pairs (Table 4) and all yielded microsatellite products. Among these products, the bands

amplified by primers NAU1157, NAU1355, NAU1052, JESPR156, BNL1679, and NAU3093 in the F_1 and S_1 hybrids showed complementary contributions from both parents (Fig. 5A). In addition to bands that were inherited from the parents, novel bands in the F_1 and S_1 hybrid plants were amplified by primers NAU1052 and NAU3093 (Fig.5Ac,f), indicating that mutations and chromosomal recombination had occurred between the parental genomes during hybridisation. The other three primers did not amplify new bands (Fig.5Aa,b,d,e). In summary, both F_1 and S_1 were confirmed as true hybrids by field morphological observation and by SSR molecular marker identification.

Validation of the synthetic allotetraploid S_1 by flow cytometry and microscopy

The flow cytometry test results showed that the peak threshold value of the female of *G. herbaceum* L (External standard reference) was at approximately 200 channels (Fig. 5B), and the peaks of the mixed diploid F_1 and tetraploid S_1 were at approximately 200 and 400 channels respectively (Fig. 5C). The results showed that the F_1 was diploid, which was the same as the *G. herbaceum* L. reference, and that S_1 was tetraploid.

By microscopic observation, 52 chromosomes were counted in the root tips of S_1 cells in the middle stage of mitosis (Fig. 5D, black arrow), indicating that the F_1 was diploid (i.e. had 26 chromosomes; Fig. 5E, black arrow) and had successfully doubled into tetraploids after colchicine treatment.

Preliminary identification of resistance to *Verticillium dahliae* Kleb. in S_1 tetraploids

Cotton seedlings were inoculated with the fungal pathogen (*Verticillium dahliae* Kleb.) (Fig. 6A, black arrow) in the greenhouse. The susceptible control, upland cotton *Jimian-11*, had a large number of yellow leaves 7 days after inoculation, and a large number of leaves fell off the plant 25 days after inoculation, followed by death (Fig. 6B, red arrow). In contrast, the S_1 tetraploid and its parents that had been inoculated with the same pathogen (Fig. 6C,D,E) showed no yellowing and shedding of leaves. No dead seedlings were found throughout the experimental growth period that lasted 60 days. Therefore, according to the previously published "disease classification" and

"disease index" system [41]the disease level of the control group was ranked level 4. Both S_1 and its parents had a disease ranking level of 0, which mean that the plants were resistant to the disease. Disease resistance of the S_1 tetraploid was inherited from the male parent.

Crossbreeding of the S_1 allotetraploid

The production of F_1-1 and F_1-2 hybrids. To transfer favourable resistance genes to upland cotton and to determine whether disease resistance of the S_1 (♂) could be preserved upon breeding, we hybridised it with upland cotton TM-1 (♀). The female bolls of TM-1 produced by natural pollination were not deformed (Fig. 7A), but most of the hybrid bolls were malformed, and the bolls were not full (Fig. 7B,C,D), producing only a small amount of seeds. On average, only two to five seeds were obtained per boll. Interestingly, after a strict hybridisation, two seeds that were obtained from a hybrid boll grew into different types of plants in the later stage, including one with red flowers and another one with white flowers (designated F_1-1 and F_1-2, respectively, Fig. 8C, 9D).

Biological traits. The biological traits of F_1-1 and F_1-2 and the parental plants are summarised in Tables 3. The female cotton TM-1 parent was tower-like with longer and looser branches (Fig. 8A) and bore bell-shaped flowers with milky corollas, milky filaments, milky anthers and pollen, without basal patches, a cup-shaped calyx, heart-shaped bracts of united growth, and was broad-leaved (Fig. 8A). Some male S_1 plants were tower-like, with shorter and more compact branches (Fig. 8B) and had bell-shaped flowers with pink corollas, milky filaments, yellow anthers and pollens, red basal patches, a serrated calyx, sword-shaped bracts that grew separately rather than together, and heart-shaped or broad leaves (Fig. 8B).

The F_1-1 and F_1-2 shared some characteristics but not all (Tables 3; Fig. 9C, 9D). The shared traits included tower-like plants, broad leaves, trumpet flowers, looser branches, and green stems and petioles that simultaneously display characteristics of both parents. However, F_1-1 was more similar to the male parent and F_1-2 was more similar to the female parent. The biological characteristics of the F_1-1 that were inherited from the male parent included fuzzy stems and leaves, a serrated calyx, shorter branches, pink corollas, red petal base spots, yellow anthers and pollen, and

bracteoles that grew separately. The biological characteristics of F_1-2 that were inherited from the female TM-1 parent included longer branches, stems, and leaves that were not covered with fuzz, a wavy calyx, heart-shaped bract of united growth, white and red corollas, no petal base spots, and milky anthers and pollen.

Specific and major differences between F_1-1 and F_1-2 were found in the flower colours, bolls, and plant fuzziness. F_1-1 had pink flowers, which were similar to the parental S_1 (Fig. 9C), whereas F_1-2 had white flowers, similar to the female TM-1 parent, although its pollen grains were larger (Fig. 9D). F_1-1 only bore small numbers of cotton bolls in the wild after artificial pollination, and there were no seeds and no fibres in the bolls. The stem of the F_1-1 was fuzzy during the seedling stage; in larger seedlings, fuzz covered the stems and leaves. The surfaces of the F_1-1 bolls were also marked by a small amount of fuzz, a trait that is inherited from the male S_1 (Fig. 9F, 9G). In contrast, the stem of F_1-2 was not fuzzy throughout the seedling stages. F_1-2 also produced non-fuzzy cotton bolls in the wild, a trait inherited from the female TM-1 parent (Fig. 9E,H).

To summarise, the two F_1 hybrids from the same parent showed significant morphological differences, indicating that complex genetic recombination events had occurred during distant hybridisation of two different species. This is an advantage of distant hybridisation, which produces hybrid progeny that provide abundant intermediate materials for cotton breeding, and also expanded the resources available for cotton genetic research.

It should be noted that we have only preliminarily assessed the authenticity of the F_1 generation obtained from crosses between TM-1 and S_1 through morphological characterisation. Further studies are required to confirm they are true hybrids using cytology and molecular genetics techniques.

Discussion

Wild cotton varieties are a great treasury of improved cultivars [21]. There are at least 30 varieties of wild cotton, widely distributed in the Americas, Australia, Africa, and Asia that have undergone long-term natural selection to accumulate rich genetic diversity conferring adaptability to adverse conditions and resistance to

environmental onslaughts and diseases. The cultivated varieties present today have also undergone a long period of artificial selection, to significantly improve yields and fibre quality compared to wild varieties. However, even after long-term intensive farming and artificial selection, some favourable resistant phenotypes have not developed in cultivated varieties, and were even were excluded by excessive selection. Therefore, despite the continuous improvements in yields and fibre quality, their disease resistance had weakened. Resistance breeding programmes are now being conducted to transfer the disease resistance genes from wild cotton to cultivated cotton, and to re-diversify the genetic makeup of cultivated cotton.

The superior qualities of cotton fibre, including its length, strength, fineness as well as disease resistance of wild cotton has been successfully transferred to cultivated cotton [22-24]. However, diploid wild cotton and tetraploid cultivated cotton are biologically isolated. While increased genetic diversity could be achieved from distant hybridisation, they are often genetically incompatible, forming a barrier to full utilisation of wild diploid cotton varieties [25-28]. In the present study, we successfully obtained an infertile interspecies F_1 hybrid through distant hybridisation, overcoming the genetic incompatibility barriers between two distant species. We further doubled the chromosomal content of this F_1 hybrid with colchicine treatment to overcome the significant challenge of interspecies hybrid sterility, to successfully synthesise a new tetraploid cotton species, S_1 ($A_1A_1C_4C_4$).

Hybrid separation still occurred in the subsequent S_1 generations. We found that the offspring of S_1 gradually exhibited growth decline in the field. The S_2 generation could still grow normally, blossom, and bear fruit, but the S_3 and S_4 generations had limited growth and flowered less. Plants of the S_5 generation had severely restricted growth and could not blossom, indicating that S_1 was a genetically unstable germplasm that carried the risk of natural separation. The genetic instability of S_1 severely hindered the utilisation of new cotton germplasm for breeding. We were unable to resolve this issue in the present study; thus, the control of hybrid separation for successful establishment of stable genetic strains needs to be explored in future research.

The S_1 generation had high potential for breeding due to its boll-forming properties, diversity of fibre colours, and fibre quality. Boll formation is the key intrinsic factor

that determines cotton yield. After 4 months of growth in the field, the number of bolls per S_1 plant reached 50 bolls per plant, an important characteristic for high yield breeding applications. We also found that a variety of coloured fibres developed in S_1 cotton bolls, including brown fibres in a 4-chambered boll and light green and brown cotton fibres in a 3-chambered boll (Supplementary Figure 3A,B). These observations indicated that the fibre characteristics of S_1 were recessive and dynamic. Notably, the fibre Micronaire reading (A measure of air permeability of compressed cotton fibres) of the S_1 fibres was 9.6, determined using the Macron Airflow Meter in the fibre-testing centre of the Cotton Research Institute of Chinese Academy of Agricultural Sciences. This reading was far above the parental range and may be the highest value reported to date. The S_1 fibres were very thick and provided us with the raw materials for making non-woven fabrics. These traits are also of great value in breeding programmes.

Molecular markers are specific pieces of DNA that reflect differences in the genomes of individuals or populations [29]. Since the discovery that microsatellite markers are uniformly distributed on cotton chromosomes, SSR markers have been used to monitor the infiltration of heterologous chromosome fragments in the offspring of interspecies hybrids [30,31]. In the present study, SSR detection revealed the presence of new specific bands in hybrids (Fig. 5Ac,f) that were not detected in the parents, indicating that new combinations or mutations of chromosomes are present in the interspecies progeny (Genomic A and Genomic C). Our results also support the hypothesis that distant hybridisation is an important driver of new mutations and species in plant evolution [32,33].

There is potential for genes from the new synthetic allotetraploid to fuse with favourable genes from *G. herbaceum* L. and *G. nelsonii* Fryx., including genes that encode good fibre quality, or resistance to diseases, pests, and drought. This will allow their transfer them to upland cotton through hybridisation, to further improve the resistance and fibre quality of upland cotton [34]. We tested this possibility by performing preliminary disease-resistance testing of the new S_1 species in the greenhouse, and found that the new species presented high disease resistance to the fungal pathogen *Verticillium dahliae* Kleb. When the new species were transplanted to cultivated land where disease occurs all year round, we confirmed that the new

species also showed high disease resistance to wilt verticillium in the field. This important agronomic character provided a new and important resource for cotton disease resistance breeding programmes.

When we examined whether the newly synthesised allotetraploid S_1 could be applied in cotton breeding by hybridisation with upland cotton TM-1, we found two types of plants in the F_1 hybrid offspring, namely F_1-1 with red flowers and F_1-2 with white flowers. F_1-1 plants died upon onset of anthesis, indicating that this plant type could not be applied in crossbreeding. F_1-2 plants grew normally until flowering and fruiting, but their seeds could not germinate normally, indicating that this type had the potential for breeding applications but additional research is required to overcome key difficulties in promoting seed germination each generation. This phenomenon of unsuccessful germination of F_1-2 seeds and root tip wilting had previously been observed during the early generation of the amphiploid *G. arboretum* L. hybridised with *G .bickii* Prokh. [35]. The variation in hybrid phenotypes may be related to gene overlap, new genetic variation, chromosome rearrangement, or a variety of other factors [36-39].

Thus far, we have only performed preliminary characterisations of the newly synthesised allotetraploid cotton ($A_1A_1C_4C_4$). We propose to perform more in-depth and comprehensive research to extract valuable information from this new cotton tetraploid, so as to provide a more accurate theoretical basis for cotton breeding and genetic evolution.

References

1. El-Zik,KM.Integrated control of Verticillium wilt of cotton. Phytopathology. 1985;6,1025e1032.
2. Carpenter,CW.The *Verticillium* wilt problem.Phytopathology. 1914;4,393.
3. Wang,K.,Wendel,J.&Hua,J.Designations for individual genomes and chromosomes in Gossypium.J.Cotton.Res. 2018;1,3;10.1186/s42397-018-0002-1 .
4. Fryxell,P.A revised taxonomic interpretation of *Gossypium* L.(Malvaceae).Rheedea.1992;2(2),108-65.
5. Endrizzi,J.E.,Phillips,L.L.A hybrid between *Gossypium arboreum* L.and *G.raimondii* Ulb.Can.J.Genet Cytol.1960;2(4),311-319.
6. Muramoto H.Hexaploid cotton: some plant and fiber properties.Crop Sci. 1969;9(1),27-9.
7. Li B,Zhang B,Zhang X,Niu Y.Studies on the Hybrid between *G. arboreum* L and *G.bickii* Prokh.Acta Genetica Sinica. 1987;14(2),121-6.
8. Sun,Y.et al.Production and characterization of somatic hybrids between upland cotton (*Gossypium hirsutum*) and wild cotton (*G.klotzschianum* Anderss) via electrofusion.Theor.Appl.Genet. 2004;109(3),472-479.
9. Yu,X.et al.Characteristics of fertile somatic hybrids of *G.hirsutum* L.and *G.trilobum* generated via protoplast fusion.Theor.Appl.Genet. 2012;125(7),1503-1516.
10. Newaskar,G.S.,Chimote,V.P.,Mehetre,S.S.,Jadhav,A.S. Interspecific hybridization in *Gossypium* L.:characterization of progenies with different ploidy-confirmed multigenomic backgrounds.Plant Breed. 2013;132(2),211-216.
11. Zhang,X.et al.Morphological,cytological and molecular analyses of a synthetic hexaploid derived from an interspecific hybrid between *Gossypium hirsutum* and *Gossypium anomalum*.The Crop Journal. 2014;2(5),272-7.
12. Liu,Q.et al.A New Synthetic Allotetraploid (A1A1G2G2) between *Gossypium herbaceum* and *G. australe*:Bridging for Simultaneously Transferring Favorable Genes from These Two Diploid Species into Upland Cotton.PLoS One. 2015;10(4), e0123209;10.1371/journal.pone.0123209.
13. Wu,Y.,Chen,D.,Zhu,S.,Zhang,L.,Li,L.A New Sythetic Hybrid(A1D5) between *Gossypium herbaceum* and *G. raimondii* and Its Morphological, Cytogenetic, Molecular Characterization. PLoS One. 2017;12(2),e0169833;10.1371/journal.pone.016983 .
14. Chen,D.,Wu,Y.,Zhang,X.,Li,F.Analysis of [*Gossypium capitis-viridis*×(*G.hirsutum*× *G.australe*)^2]Trispecific Hybrid and Selected Characteristics.PLoS One. 2015;10(6),e0127023;10.1371/journal.pone.0127023.
15. Paterson,A.H.,Brubaker,C.L.,Wendel,J.F.,A rapid method for extraction of cotton(*Gossypium* spp.)genomic DNA suitable for RFLP or PCR analysis.Plant.Mol.Biol.Rep. 1993;11(2),122-127.
16. Galbraith,D.W.,et al.Rapid flow cytometric analysis of the cell cycle in intact plant tissues.Science. 1983;220,1049-1051.
17. Zhang,X.H.,et al.Interspecific hybridization, polyploidization and backcross of *Brassica oleracea var. alboglabra* with *B. rapa var.purpurea* morphologically recapitulate the evolution of Brassica vegetables.Sci. Rep. 2016;10.1038/srep18618.

18. Yin,X.M.,et al. Characterisation of Early Events in Banana Root Infected with the GFP-tagged *Fusarium oxysporum* f. sp. *cubense*. Acta Hortic. 2011;(897),371-376.
19. Ping,M.A.,HUANG,H.C.,LI,S.Z.,TANG,W.H.A new inoculation method for verticillium wilt on cotton and its application in evaluating pathogenesis and host resistance.Acta Phytopathol. Sin. 2004;34(6),356-541.
20. PENG,S.,et al.Study on a new rapid inoculation method for Verticillium wilt and fusarium wilt of cotton.J.Cotton Sci. 2008;20(3),174-178.
21. Endrizzi,J.,Turcotte,E.,Kohel,R.Genetics,cytology and evolution of *Gossypium*.Adv.Genet. 1985;23,271-375.
22. Campbell,B.,Williams,V.,Park,W.Using molecular markers and field performance data to characterize the Pee Dee cotton germplasm resources.Euphytica. 2009;169,285-301.
23. Culp,T.,Harrell,D.Breeding methods for improving yield and fiber quality of upland cotton (*Gossypium hirsutum* L.).Crop Science. 1973;13,686-689().
24. Qian,S.et al.Studies on the hybrid of *G.hirsutum* L.and *G.anomalum* Wawr.& Peyr.and application in breeding.Sci.Agric.Sinica. 1992;25,44-51.
25. Oakes,A.J.A Sterility in certain *Gossypium* hybrids.I. Prefertilization phenomena.Can.J.Genet Cytol. 1966;8(4),818-829.
26. Sacks,E.J.Ovule rescue efficiency of *Gossypium hirsutum*× *G. arboreum* progeny from fieldgrown fruit is affected by media composition and antimicrobial compounds.PCTOC. 2008;93,15-20.
27. Ganesh,R.S.,Hari,R.S.,Thiruvengadam,V.,Kannan,B.J.Prefertilization barriers to interspecific hybridization involving *Gossypium hirsutum* and four diploid wild species.Plant Breed. 2008;127,295-300.
28. Li,B.L.,Zhang,B.J.,Zhang,X.R.,Niu,Y.Z.Studies on the Hybrid between *G.arboreum* L and *G.bickii* Prokh.Acta Genetica Sinica. 1987;14,121-126.
29. Benbouza,H.,et al.Introgression of the low-gossypol seed & high-gossypol plant trait in upland cotton:Analysis of [(*Gossypium hirsutum*×*G. raimondii*)×*G.sturtianum*] trispecific hybrid and selected derivatives using mapped SSRs.MOL BREEDING. 2010;25(2),273-86.
30. Siu,L.,Saha,S.,Stelly,D.,Burr,B.,Cantrell,R.Chromosomal assignment of microsatellite loci in cotton.J. Hered. 2000;91(4),326-32.
31. Barton,N.H.,The role of hybridization in evolution.Mol.Ecol. 10(3),551-68(2001).
32. Jiao,Y.,et al.Ancestral polyploidy in seed plants and angiosperms. Nature. 2011;473(7345),97-100.
33. Soltis,P.S.,Soltis,D.E.The role of genetic and genomic attributes in the success of polyploids.Proc Natl Acad Sci. 2000;97(13),7051-7057.
34. Chen,Y.,et al. A new synthetic amphiploid (AADDAA) between *Gossypium hirsutum* and *G. arboreum* lays the foundation for transferring resistances to Verticillium and drought.PLoSOne. 2015;10(6),e0128981;10.1371/journal.pone.0128981.
35. Kim,N.Z.,et al.Altered circadian rhythms regulate growth vigour in hybrids and allopolyploids.Nature. 2009;457(7227),327-331.
36. Kashkush,K.,Feldman,M.,Levy,A.A.Gene loss,silencing and activation in a newly synthesized wheat allotetraploid.Genetics. 2002;160(4),1651-9.
37. Rieseberg,L.H.Evolution:replacing genes and traits through hybridization.Curr. Biol. 2009;19(3),119-22.

38. Rowe,H.C.,Rieseberg,L.H.Genome-scale transcriptional analyses of first-generation interspecific sunflower hybrids reveals broad regulatory compatibility.BMC Genomics. 2013;14,342.
39. Shaked,H.,Kashkush,K.,Ozkan,H.,Feldman,M.,Levy,A.A.Sequence elimination and cytosine methylation are rapid and reproducible responses of the genome to wide hybridization and allopolyploidy in wheat.Plant Cell. 2001;13(8),1749-59.

Acknowledgements

This research work was financially supported by the National Natural Science Foundation of China (No. 31704178) and Central Public-interest Scientific institution Basal Research Fund for Chinese Academy of Tropical Agricultural Sciences(No.1630092017004).

We are grateful to Dr Kunbo Wang and Dr Fang Liu of the Cotton Research Institute of Chinese Academy of Agricultural Sciences for kindly providing us pollen of *G. nelsonii* Fryx. to use at the National Wild Cotton Nursery, Sanya, China.

Author Contributions

X.M.Y. conceived this work, D.C. and X.M.Y. performed the experiments, R.L.Z. wrote the manuscript, B.Q.X., L.X.W., and Y.G. interpreted the data and figures, and S.S. and Y.D.H. reviewed the manuscript.

Additional information

Competing financial interests: The authors declare no competing financial interest.

Figure Legends

Figure 1. Growth and formation of the synthetic tetraploid, S_1. (**A**) Tetraploid plants with a large pink flower accompanied by yellow pollen. (**B**) The growth process of the oval boll. (**cC**) Four seeds with brown fibres.

Figure 2. Pictures of parent and hybrid plants. (**A**) The female *G. herbaceum* L. parent had a tower shape with shorter and more compact branches. (**B**) The male *G. nelsonii* Fryx. was cylindrical with short and compact branches. (**C**) The diploid F_1 hybrid was tower-shaped, with longer branches and looser spacing but were infertile. (**D**) The tetraploid, S_1, were tower-shaped with long fruit branches and loose spacing. They were fertile.

Figure 3. Pictures of leaves and flowers. (**A**) *G. herbaceum* L. bore bell-shaped flowers with yellow corollas, pink basal patches, pink filaments, yellowish anthers, yellow pollen, cup-shaped calyxes, heart-shaped bracts that grow separately rather than together, and were broad-leaved. (**B**) *G. nelsonii* Fryx. bore tubular-shaped flowers with pale pink corollas, purple basal spots, red filaments and anthers, milky pollen, wavy calyxes, lanceolate bracts that were detached rather than united, and ovoid leaf blades. (**C**) The F_1 hybrid had bell-shaped flowers with alternating red and white corollas, red basal patches, pink filaments, yellow anthers and pollen, serrated calyxes, sword-shaped bracts that grow separately rather than together, and had broad-leaved, ovoid, and subcircular leaves. (**D**) The S_1 had trumpet and funnel-shaped flowers with alternating red and white corollas, red basal patches, light pink filaments, yellow anthers and pollen, serrated and wavy calyxes, triangle dentate and ensiform bracts that grew separately rather than together, and had broad-leaved, ovoid, and subcircular leaves.

Figure 4. Pictures of cotton bolls and fibres. (A) *G. herbaceum* L. had conical, smooth green and red bolls. The bulge at the top of the boll was distinctly cone-shaped. (B) *G. nelsonii* Fryx. had oval, green cotton bolls with fine hairs on the surface. The boll tip was not obvious. (C) Green pear-shaped boll of S_1 covered with downy hairs. (D) *G. herbaceum* L. had 3-chambered bolls with grey-white spiral cotton fibres. (E) *G. nelsonii* Fryx. had 3-chambered bolls with dark brown upright fibres. (F) The S_1 had 3-chambered bolls with brown fibres, and the seed hairs grew upright.

Figure 5. Validation of S_1 by SSR polymorphisms,flow cytometry and microscopy. (A) Microsatellite loci identified by SSR primers including NAU1157(a),NAU1355(b),NAU1052(c),JESPR156(d),BNL1679(e),NAU3093(f).the novel bands produced in F_1 and S_1 are indicated by red and blue arrows, (1, *G. herbaceum* L.;2, *G. nelsonii* Fryx.;3, the F_1;4, the S_1).(B)Distribution peak of *G. herbaceum* L.at approximately 200 channels.(C)Distribution peak of the tetraploid S_1 at approximately 400 channels . Fluorescence intensities of propidium-iodide stained genomic DNA. (D)The 26 chromosomes in F_1 hybrid.(E)The 52 chromosomes in S_1 tetraploid.The root tip cells at the end of mitosis(red arrows) and the chromosomes(black arrows) stained with Carbol fuchsin solution under 40× microscope.

Figure 6. Identification of resistance to *Verticillium dahliae* Kleb. (A) The pathogen of the candida conidia is indicated with the black arrow. (B) Disease-susceptible upland cotton *Jimian11*, with disease symptoms of yellowing, necrosis, and leaf abscission indicated by red arrows. (C) The resistant S_1.(D) resistant *G. nelsonii* Fryx. (E) resistant *G. herbaceum* L.

Figure 7. Cotton bolls from cotton TM-1 and from the F_1 hybrid of TM-1 × S_1 tetraploid. (A) The normal conical boll of TM-1 shown by the red arrow. (B) The abnormally conical boll of TM-1 × S_1 F_1 plants on the right and on the left(C), indicated by yellow arrows. (D) The deformed hybrid boll of F_1 plants indicated by yellow arrows.

Figure 8. Cotton TM-1, the S_1 tetraploid and F_1-1, F_1-2 plants. (A) Cotton TM-1 was tower-like with longer and looser branches. (B) S_1 was tower-like with shorter and more compact branches. (C) Hybrid F_1-1 was tower-like with shorter and looser branches and pink flowers (red arrow). (D) Hybrid F_1-2 was tower-like with longer and looser branches and had white flowers (white arrow).

Figure 9. Pictures of leaves, flowers, and bolls of cotton TM-1, the S_1 tetraploid, and of F_1-1 and F_1-2 plants. (A) Cotton TM-1 bore bell-shaped flowers with milky corollas, milky filaments, milky anthers and pollen, without basal patches, wavy calyxes, heart-shaped bracts grown together, and were broad-leaved. (B) S_1 had bell-shaped flowers with pink corollas, milky filaments, yellow anthers and pollen, red

basal patches, serrated calyxes, sword-shaped bracts grown separately rather than together, and had heart-shaped or broad leaves. (C) Hybrid F_1-1 had bell-shaped flowers with pink corollas, milky filaments, yellow anthers and pollen, red basal patches, serrated calyxes, heart-shaped bracts grown separately rather than together, and broad leaves. (D) Hybrid F_1-2 bore bell-shaped flowers which were milky white in the middle with a light red corolla border, milky filaments, milky anthers and pollen, without basal patches, wavy calyxes, heart-shaped bracts grown together, and broad leaves. (E) A normal conical boll of cotton TM-1. (F) A green pear-shaped boll of S_1 with a sunken navel. (G) A downy green boll of F_1-1 that was deformed, indicated by the red arrow. (H) A normal conical boll of F_1-2.

Table 1. Comparison of shape and colours of plant parts in F_1 and S_1

Shape/ colours	*G. herbaceum* L. (♀)	*G. nelsonii* Fryx. (♂)	Diploid F_1	Tetraploid S_1
Plant	Tower	Cylindrical	Tower	Tower
Leaf	Broad-leaved	Ovoid	Broad-leaved, ovoid, subcircular	Broad-leaved, ovoid, subcircular
Flower	Trumpet	Funnel-shaped	Trumpet	Trumpet, Funnel-shaped, cylindrical, closed, half closed
Calyx	Cup-shaped	Wavy	Serration	Serrated, Wavy
Bract	Heart-shaped	Lanceolate	Sword-shaped	Triangle Dentate, ensiform
Boll	Conical	Oval	/	Pear-shaped, sunken navel, oval, irregular round boll
Fibre	Spiral	Upright	/	Wavy
Stem	Green	Brown	Green	Green
Leaf	Green	Grey green	Green	Grey green
Corolla	Yellow	Light pink	Pink	Pink, Light pink, Milky white, Red and white
Petal base spot	Pink	Purple	Red	Red, Purple, Rose, None
Filaments	White	Pink	Light pink	Light pink
Anther	Yellow	Red	Light yellow	Yellow
Pollen	Yellow	Pink	Yellow	Yellow
Boll	Red and green	Green	/	Green
Fibre	Grey-white	Dark brown	/	Light green, Brown

Table 2. Comparison of quantifiable parameters in F_1 and S_1

Quantifiable parameters	*G. herbaceum* L. (♀)	*G. nelsonii* Fryx. (♂)	Diploid F_1	Tetraploid S_1
Plant height (cm)	80	200	300	150
Main stem diameter (cm)	1.5	2	3.0	2.5
Leaf length (cm)	4~6	5~9	4~6	4~8
Leaf width (cm)	4~6	4~6	4~6	4~7
Leaf lobation	3~5	0~1	0~3	0~3
Bract tooth	5~9	1	4~6	4~6
Locule	3	3	/	3~4
Seeds per locule	1-6	1-5	/	1~5
Bracteole	Joint	No joint	No joint	No joint, Jointed
Stem fuzz	Less	Medium	More	More
Leaf fuzz	No	Medium	More	More
Leaf Lobes	Parted	No	Lobate	Lobate
Column length	Medium	Long	Medium	Medium, Long, short
The size of Petal base spot	Small	Medium	Medium	Medium, Small, None
Boll tip protuberance degree	Medium	Weak	/	None, Weak
Branch length	Short	Short	Long	Long
Branch openness	Compact	Compact	Looser	Looser

Table 3. Comparison ofshape and colours of plant parts in F_1-1 and F_1-2

Shape/colours	TM-1 (♀)	A selected S_1 (♂)	Hybrid F_1-1	Hybrid F_1-2
Plant	Tower	Tower	Tower	Tower
Leaf	Broad-leaved	Broad-leaved, Ovoid, Heart-shaped	Broad-leaved	Broad-leaved
Flower	Trumpet	Trumpet	Trumpet	Trumpet
Calyx	Cup-shaped	Serration	Wavy	Cup-shaped
Bract	Heart-shaped	Sword-shaped	Heart-shaped	Heart-shaped
Boll	Conical	Pear-shaped	Deformed Conical	Deformed conical
Branch length	Longer	Shorter	Shorter	Longer
Branch openness	Looser	Compact	Looser	Looser
Bracteole	Joint	No joint	No joint	Jointed
Stem	Red	Red	Green	Green
Leaf	Green	Green	Green	Green
Corolla	Milky	Pink	Pink	White and red
Petal base spot	None	Red	Red	None
Filaments	Milky	Milky	Milky	Milky
Anther	Milky	Yellow	Yellow	Milky
Pollen	Milky	Yellow	Yellow	Milky

Table 4. SSR polymorphism primer sequences

Primer number	Forward sequence (5'-3')	Reverse sequence (5'-3')
NAU1157	GAGTTTGGTTCTGGGTTGAG	GATCCTTTTCATCTCCTCCA
NAU1355	ATCTGTTTACGCCACTCTCC	CCAGCCTTTGACATTTTTCT
NAU1052	CGCAGATAAAGGATGGATTT	AGAGCTGGAGGACATAACAAA
JESPR156	GCCTTCAATCAATTCATACG	GAAGGAGAAAGCAACGAATTAG
BNL1679	AATTGAGTGATACTAGCATTTC AGC	AAAGGGATTTGCTGGCAGTA
NAU3093	GTCTTGAACCGGAACTTGAT	TCCTGTTGAACACCAAAGTG

The primer sequences were obtained from http://www.cottonmarker.org/cgi-bin/cmd_search_marker.cgi

Figures:

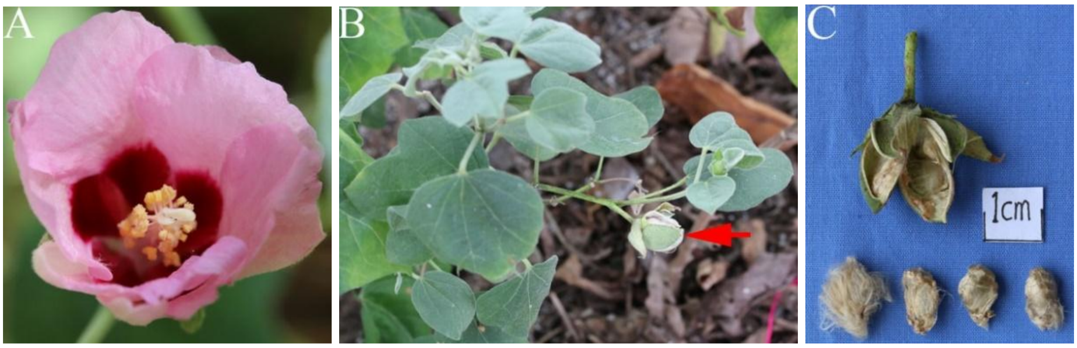

Figure 1

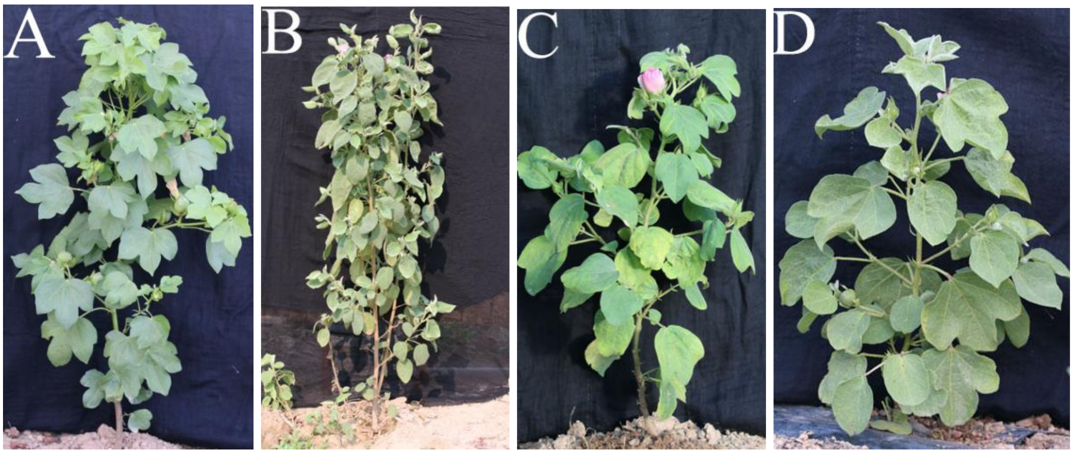

Figure 2

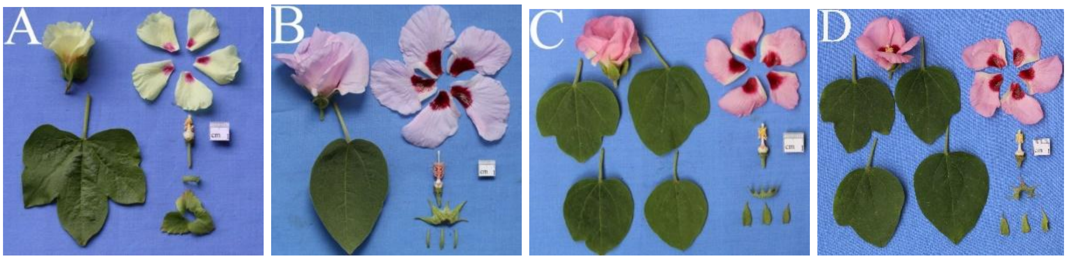

Figure 3

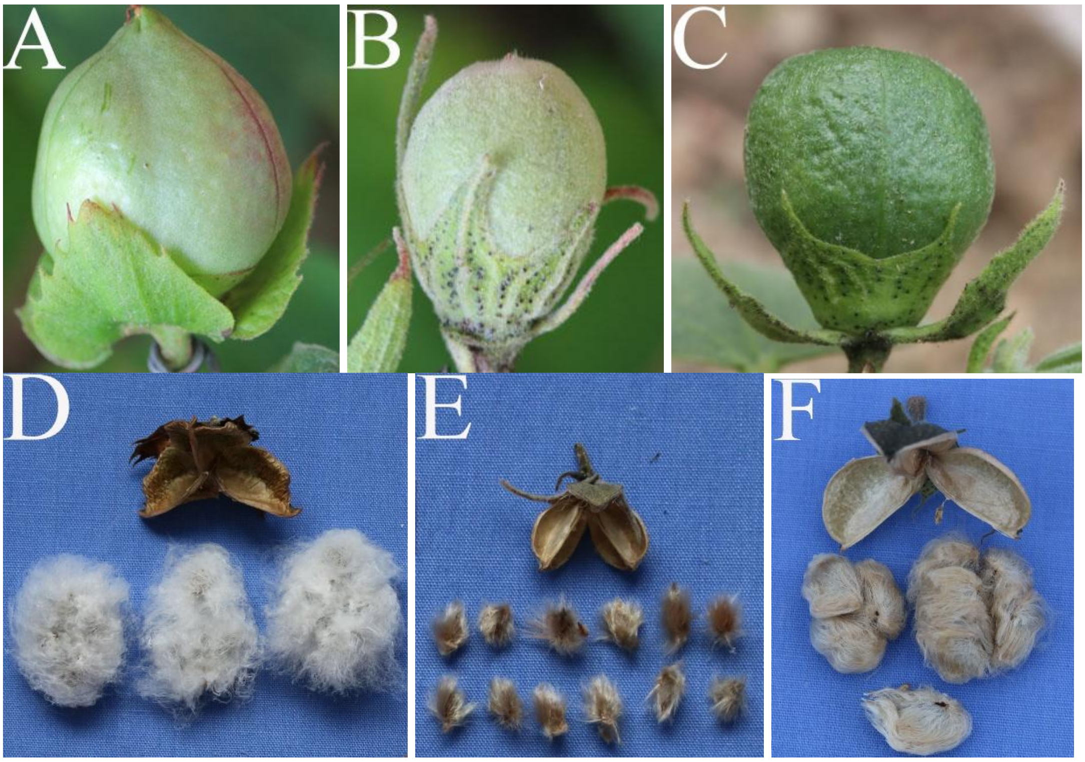

Figure 4

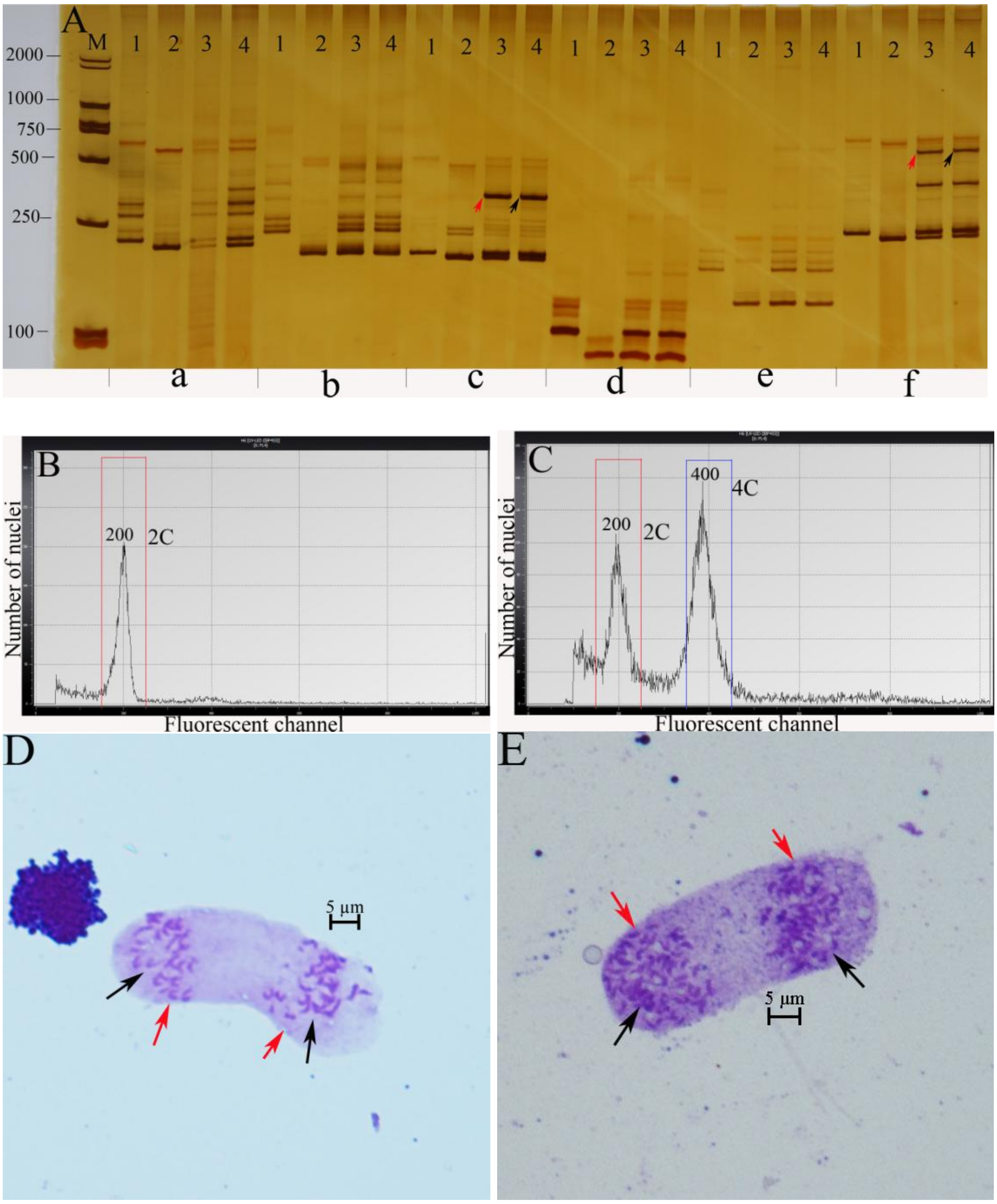
A
M
1 2 3 4 1 2 3 4 1 2 3 4 1 2 3 4 1 2 3 4 1 2 3 4
2000
1000
750
500
250
100
a
b
c
d
e
f
B
200
2C
Number of nuclei
Fluorescent channel
C
200
2C
400
4C
Number of nuclei
Fluorescent channel
D
5 µm
E
5 µm

Figure 5

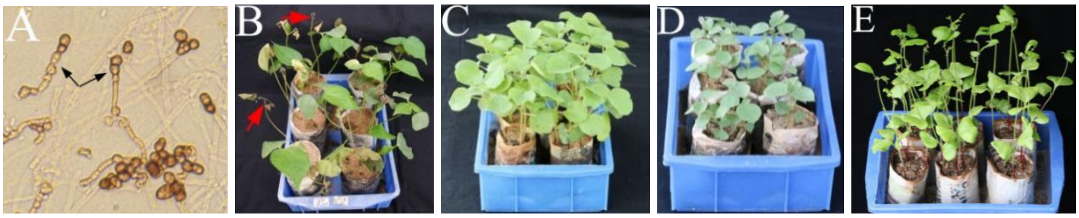

Figure 6

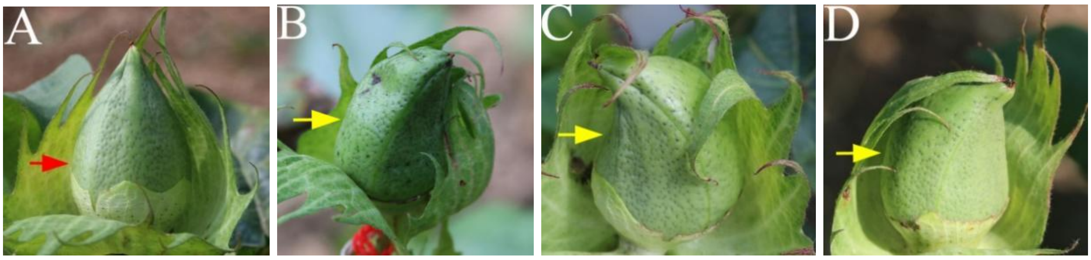

Figure 7

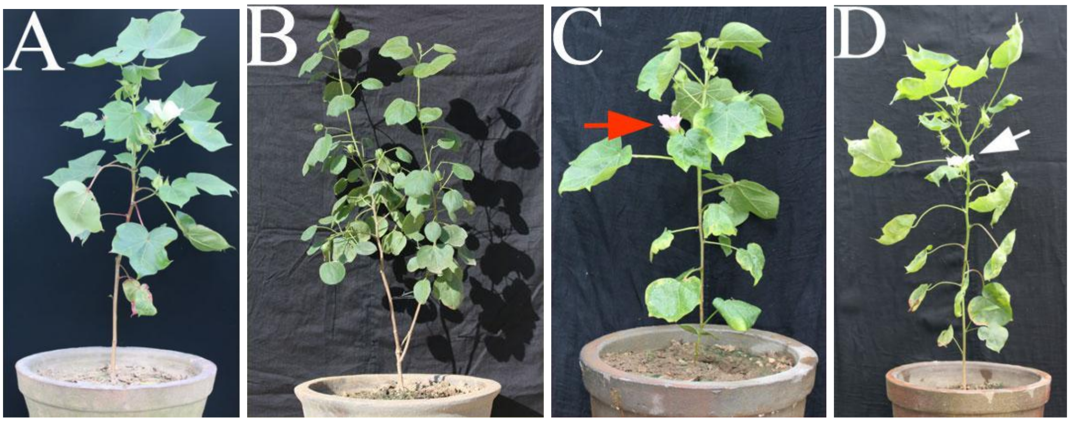

Figure 8

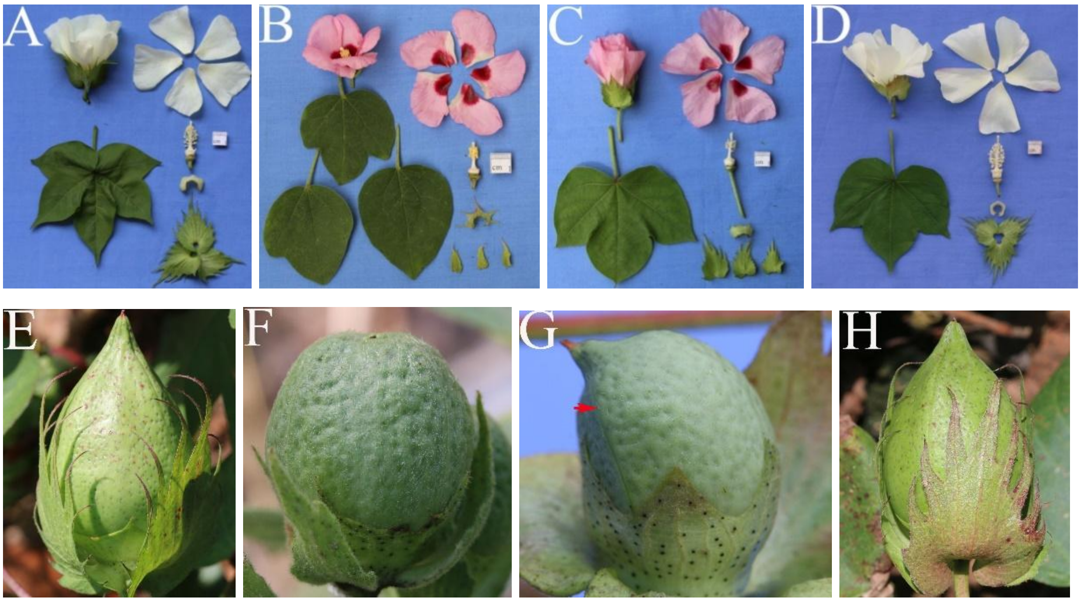

Figure 9

Click here to access/download
Supporting Information
Supporting Informationof chendi from CATAS,0131.docx
