# Supplementary material for: Morphological description of a novel synthetic allotetraploid(A1A1G3G3) of Gossypium herbaceum L.and G.nelsonii Fryx. suitable for disease-resistant breeding applications
Source: PLoS One. 2020 Dec 3;15(12):e0242620. doi: 10.1371/journal.pone.0242620 (PMC7714114; doi:10.1371/journal.pone.0242620)
Supplement: Attachment — Submitted filename: PONE-D-20-02970_R1 (2).pdf [file pone.0242620.s007.pdf]

PLOS ONE

Morphological and genetic description of a novel synthetic allotetraploid of Gossypium herbaceum L. and Gossypium nelsonii Fryx.

--Manuscript Draft--

**Manuscript Number:**	PONE-D-20-02970R1
**Article Type:**	Research Article
**Full Title:**	Morphological and genetic description of a novel synthetic allotetraploid of Gossypium herbaceum L. and Gossypium nelsonii Fryx.
**Short Title:**	Morphological and genetic description of a novel synthetic allotetraploid
**Corresponding Author:**	Di Chen<br>Chinese Academy of Tropical Agricultural Sciences<br>Haikou, CHINA
**Keywords:**	genetic evolution, Gossypium, interspecific hybridization, synthetic allotetraploid
**Abstract:**	Gossypium herbaceum L. is a cultivated, diploid cotton species (2n = 2x = 26, A_1A_1) with superior resistance to drought, sap-feeding insects, and cotton leaf curl virus. Gossypium nelsonii Fryx. is a wild, diploid cotton species (2n = 2x = 26, G_3G_3) that produces strong fibers and is resistant to drought, aphids, and Verticillium wilt and can delay pigment gland formation. These cotton species were used to synthesize an allotetraploid cotton species for use as a foundation for the future transfer of beneficial genes to cultivated cotton. Here, we used G. herbaceum (♀) and G. nelsonii (♂) for interspecific hybridizations to obtain F_1 generation. The apical growth points were immersed in colchicine to induce chromosome doubling. Tetraploid S_1 cotton bolls were then obtained from the diploid F_1 plants to successfully synthesize tetraploid cotton plants. Morphological markers, detecting DNA polymorphism,following ploidy analysis and microscopic observation were used to identify the tetraploid S_1 plants, and then self-pollination was conducted. The S_1 plants showed good growth without significant segregation, indicating that the compatibility among them had been maintained. The S_2 plants showed obvious segregation, indicating some incompatibility. The S_2 seedlings that were cultivated in nutrient pots were inoculated with Verticillium wilt, but they did not develop the disease and had an immune phenotype. The S_1 interspecific hybrid S_1 plants were hybridized with cultivated G. hirsutum L. to measure the compatibility of the S_1 plants and G. hirsutum L. and to determine if they could be used for cotton species improvements. The results showed that these plants had some compatibility and that fertile hybrid progeny could be obtained. The newly synthesized allotetraploid S_1 line will have important application value in polyploidy evolution studies and could act as a bridge to transfer beneficial genes from two diploid parental cotton species to G. hirsutum L.
**Order of Authors:**	Xiaomin Yin
	Rulin Zhan
	Shun Song
	Yingdui He
	Lixia Wang
	Yu Ge
	Di Chen
**Response to Reviewers:**	1. The description of phenotypic morphological analysis of the diploid F1 hybrid and the synthetic S1 tetraploid is poorly organized. The author's investigation of morphological in materials and methods has not been described in detail. Even with reference to the methods of previous reference, no specific survey data and criteria are given. In the survey results, the key traits such as the germinating stage and the flowering stage are unclear. Moreover, wild cotton is mostly perennial wild material, and its germinating stage and flowering stage belongs to agronomic traits. It is not suitable for investigation and comparative analysis of wild materials.

Powered by Editorial Manager® and ProduXion Manager® from Aries Systems Corporation

__Answer：We have added the key traits such as germination and flowering of new germplasm cotton.The F1 hybrid were sown in a pot and all germinated after 5 days. After the first true leaves were level, the seedlings were transplanted to the field. Floral buds appeared after 43-45 days and flowering occurred after 68-70 days. The S1 were sown in a pot and all germinated after 5 days. After the first true leaves were level, the seedlings were transplanted to the field. Floral buds appeared after 45-50 days and flowering occurred after 70-75 days. Boll opening occurred after 107-115 days.
The new cotton germplasm was created by using wild cotton through distant hybridization and is the descendant of wild cotton.When investigating and analyzing it, it involves genetic problems.To explain the genetic problems of new germplasm, the source needs to be traced back. Therefore, it is necessary to list wild cotton parents as a reference in the study in order to analyze and explain.The reviewer mentioned that wild cotton is mostly perennial wild material, this statement is correct.
In fact, all cotton is a perennial plant in tropical regions, including wild cotton and cultivated cotton (such as G. hirsutum L. G. barbadense L.G. herbaceum L.G. arboreum L.). The morphological traits of various cotton species grown in the same environment difference.
2. Statistical analysis of leaf shape, plant height, boll shape and other traits is not appropriate. Because the leaf shape will change according to the different growth stages of the plant, the plant height will vary greatly due to changes in the growth environment, and the boll shape will also vary in different positions of the plant. The number of bolls per plant is also strongly related to nutritional status and planting density, so the author's description of these traits seems meaningless.
__Answer：Plant morphology is a characteristic expression of a species, mainly including plant morphology traits such as plant type, stem, leaf, flower and cotton boll.Regarding the color of the floral organs of the plant, the presence or absence of the floral basal spot, the characteristics of the bracts, the amount of plant hairs, and the boll fertility is a trait that can be stably inherited and does not change with the external environment.In a limited time, these descriptions of the new germplasm we created, please dear editors and review experts to understand.We describe morphology and only want to show the new cotton germplasm as much as possible. If we talk about its significance, the birth event of new cotton germplasm is meaningful in itself.

3. When statistically analysis the differences in phenotypic traits, it is necessary to distinguish between qualitative and quantitative traits. For F1, in the case of a limited single plant, it cannot be compared with a tetraploid with a larger number of offspring. As for the results of the investigation against Verticillium wilt, as it is a quantitative trait, it is recommended to compare and analyze the resistance differences of different isolated plants of S1.
__Answer：Due to the low number of diploid F1 hybrids and tetraploid S1 plants, these two materials cannot be used for cotton Verticillium wilt resistance experiments.In fact, in this study, we used the descendant material S2 of S1 to carry out disease resistance identification of seedlings in indoor nutrition bowls（Fig.5）, because S2 material is not only a large number, but also an ideal separation group with rich morphological diversity .In the next step, we will plant S2 in a specific field nursery, and analyze and compare the differences in disease resistance of different S2 isolated plants.
4. For the identification and analysis of F1 true or false hybrids, when doing cytological analysis, the chromosomes of both parents and the chromosome of F1 should be observed.
__Answer：We have shown the cytological observations of both parents, F1 and S1 in the article.
5. When conducting a flow cytometer test, both parents, F1, S1, and the TM-1 materials, should be tested and investigated to increase the scientificity and credibility of the test.
__Answer：We have shown the results of flow cytometer test of female parent, F1, S1 and TM-1 in this article.

6. At present, according to the taxonomic standards of the cotton genus, it has been recognized that Gossypium nelsonii Fryxell has been classified as the G3 species. So the newly synthesized double cotton germplasm should be A1A1G3G3.
__Answer：We have changed to A1A1G3G3 in the article.
7. According to the cotton genus classification and distribution research, 53-54 cotton species have been discovered and named, instead of 55.
__Answer：We have changed to 54 kinds in the article.

Powered by Editorial Manager® and ProduXion Manager® from Aries Systems Corporation

	Reviewer #2:<br>The manuscript has potential for publication, i the authors have achieved good results, the discussion needs to be improved, with mention to other works, in the discussion it is necessary to mention other results found in the difference of ploidy, the use of colchicine, as well as resistance to Verticillium dahliae, which is one of the main objectives, however, the work has all merit for publication.<br>__Answer：We have added relevant content to the discussion of the article. The tetraploid S1 is a new germplasm obtained by chromosome doubling of the diploid F1 hybrid and is a polyploidy event in cotton. This not only restores fertility to sterile diploid F1 hybrids and the tetraploid S1 generation obtained showed segregation, which increases the phenotypic mutations in the species, enabling breeders to select for required traits. After the polyploid was generated, its stress resistance not only did not decrease but biotic stress resistance was maintained. For example, the preliminary examination showed that the disease resistance of the tetraploid S2 was 1 grade better than the female parent (G. herbaceum) and the same level as the male parent (G. nelsonii), and its disease resistance inheritance was closer to the male parent. In addition, the fiber quality of the tetraploid S2 exceeded that of both parents. From this we can see that diploid F1 hybrid polyploidy has important significance for cotton evolution, as the genome structure changed and the species genetic diversity enriched as well as the species adaptability and fiber quality were improved.<br><br>Reviewer #3:<br>WHAT THE AUTHORS NEED TO DO FOR IMPROVE THEIR PAPER• I THEORIZED THAT YOU MUST FOLLOW A LANGUGE EDITING FIRSTLY AND AFTERWARDS WE COULD CAPABLE TO FOLLOW DETAILED CORRECTIONS AND SUGGESTIONS.• I AM SORRY BUT THERE MAINY TERMS AND SENTENCES THAT MUST BE REWRITE USING BETTER TERMINOLOGY.ALL THE SENTENCES, TERMS AND WORDS UNDERLINED INTO THE TEXT BY YELLOW COLOUR SHOULD BE CHANGED.• PLEASE FOLLOW A LANGUAGE EDITIND FROM A PERSON WHO HAS ENGLISH AS NATIVE LANGUAGE BECAUSE YOU LOOSE MUCH OF THE VALUE OF YOUR WORK .<br>PLEASE SEE CAREFULLY OUR ADVICES INTO THE FILE COMMENTS & SUGGESTIONS (1)<br>__Answer：We have made changes to the questions raised by the review experts.
**Additional Information:**	
**Question**	**Response**
**Financial Disclosure**<br><br>Enter a financial disclosure statement that describes the sources of funding for the work included in this submission. Review the submission guidelines for detailed requirements. View published research articles from *PLOS ONE* for specific examples.<br><br>This statement is required for submission and **will appear in the published article** if the submission is accepted. Please make sure it is accurate.	YES - Specify the role(s) played.This research work was financially supported by the National Natural Science Foundation of China (No. 31704178) and Central Public-interest Scientific institution Basal Research Fund for Chinese Academy of Tropical Agricultural Sciences(No.1630092017004).

Powered by Editorial Manager® and ProduXion Manager® from Aries Systems Corporation

**Unfunded studies**<br>Enter: *The author(s) received no specific funding for this work.*<br><br>**Funded studies**<br>Enter a statement with the following details:<br>• Initials of the authors who received each award<br>• Grant numbers awarded to each author<br>• The full name of each funder<br>• URL of each funder website<br>• Did the sponsors or funders play any role in the study design, data collection and analysis, decision to publish, or preparation of the manuscript?<br>• **NO** - Include this sentence at the end of your statement: *The funders had no role in study design, data collection and analysis, decision to publish, or preparation of the manuscript.*<br>• **YES** - Specify the role(s) played.<br><br>* typeset	
**Competing Interests**<br><br>Use the instructions below to enter a competing interest statement for this submission. On behalf of all authors, disclose any competing interests that could be perceived to bias this work—acknowledging all financial support and any other relevant financial or non-financial competing interests.<br><br>This statement **will appear in the published article** if the submission is accepted. Please make sure it is accurate. View published research articles from *PLOS ONE* for specific examples.	The authors have declared that no competing interests exist.

**NO authors have competing interests**<br><br>Enter: *The authors have declared that no competing interests exist.*<br><br>**Authors with competing interests**<br><br>Enter competing interest details beginning with this statement:<br><br>*I have read the journal's policy and the authors of this manuscript have the following competing interests: [insert competing interests here]*<br><br>* typeset	
**Ethics Statement**<br><br>Enter an ethics statement for this submission. This statement is required if the study involved:<br><br>• Human participants<br>• Human specimens or tissue<br>• Vertebrate animals or cephalopods<br>• Vertebrate embryos or tissues<br>• Field research<br><br>Write "N/A" if the submission does not require an ethics statement.<br><br>General guidance is provided below. Consult the submission guidelines for detailed instructions. **Make sure that all information entered here is included in the Methods section of the manuscript.**	No permits were required for the described study, which complied with all relevant regulations.

Powered by Editorial Manager® and ProduXion Manager® from Aries Systems Corporation

**Format for specific study types**<br><br>**Human Subject Research (involving human participants and/or tissue)**<br>• Give the name of the institutional review board or ethics committee that approved the study<br>• Include the approval number and/or a statement indicating approval of this research<br>• Indicate the form of consent obtained (written/oral) or the reason that consent was not obtained (e.g. the data were analyzed anonymously)<br><br>**Animal Research (involving vertebrate animals, embryos or tissues)**<br>• Provide the name of the Institutional Animal Care and Use Committee (IACUC) or other relevant ethics board that reviewed the study protocol, and indicate whether they approved this research or granted a formal waiver of ethical approval<br>• Include an approval number if one was obtained<br>• If the study involved *non-human primates*, add *additional details* about animal welfare and steps taken to ameliorate suffering<br>• If anesthesia, euthanasia, or any kind of animal sacrifice is part of the study, include briefly which substances and/or methods were applied<br><br>**Field Research**<br><br>Include the following details if this study involves the collection of plant, animal, or other materials from a natural setting:<br>• Field permit number<br>• Name of the institution or relevant body that granted permission	
**Data Availability**<br><br>Authors are required to make all data underlying the findings described fully available, without restriction, and from the time of publication. PLOS allows rare exceptions to address legal and ethical concerns. See the PLOS Data Policy and FAQ for detailed information.	Yes - all data are fully available without restriction

A Data Availability Statement describing where the data can be found is required at submission. Your answers to this question constitute the Data Availability Statement and **will be published in the article**, if accepted.<br><br>**Important:** Stating 'data available on request from the author' is not sufficient. If your data are only available upon request, select 'No' for the first question and explain your exceptional situation in the text box.<br><br>Do the authors confirm that all data underlying the findings described in their manuscript are fully available without restriction?	
**Describe where the data may be found in full sentences. If you are copying our sample text, replace any instances of XXX with the appropriate details.**<br><br>• If the data are **held or will be held in a public repository**, include URLs, accession numbers or DOIs. If this information will only be available after acceptance, indicate this by ticking the box below. For example: *All XXX files are available from the XXX database (accession number(s) XXX, XXX.*).<br>• If the data are all contained **within the manuscript and/or Supporting Information files**, enter the following: *All relevant data are within the manuscript and its Supporting Information files.*<br>• If neither of these applies but you are able to provide **details of access elsewhere**, with or without limitations, please do so. For example:<br><br>*Data cannot be shared publicly because of [XXX]. Data are available from the XXX Institutional Data Access / Ethics Committee (contact via XXX) for researchers who meet the criteria for access to confidential data.*<br><br>*The data underlying the results presented in the study are available from (include the name of the third party*	All relevant data are within the manuscript and its Supporting Information files.

and contact information or URL).<br>• This text is appropriate if the data are owned by a third party and authors do not have permission to share the data.<br><br>* typeset	
Additional data availability information:	

Manuscript Click here to access/download;Manuscript;renamed_b9f2d.doc

Morphological and genetic description of a novel synthetic allotetraploid of *Gossypium herbaceum* L. and *Gossypium nelsonii* Fryx.

Xiaomin Yin[#], Rulin Zhan[#],Shun Song, Yingdui He, Lixia Wang, Yu Ge, Di Chen [*]

Haikou Experimental Station, Chinese Academy of Tropical Agricultural Sciences (CATAS), Haikou, China

Correspondence and requests for materials should be addressed to C.D. Haikou Experimental Station, Chinese Academy of Tropical Agricultural Sciences (CATAS), Haikou, 86+0898-66774403, China. Email: chendi209@163.com

Abstract

The cotton pathogen *Verticillium dahliae* Kleb. infects cotton during the planting process and during its growth and seriously reduces yields. While wild cotton exhibits high levels of resistance to this pathogen, cultivated upland cotton does not. In this study, we created a new germplasm of cotton through remote hybridisation during our search for a resistant source of wild cotton. *G. herbaceum* L. (♀) was crossed with *G. nelsonii* Fryx. (♂) to produce an interspecies F_1 hybrid. Colchicine was used to double its chromosomal content, leading to the successful generation of a synthetic S_1 tetraploid. The newly created S_1 tetraploid was characterised by morphological analysis, polymorphism detection, ploidy analysis, and microscopic observation of chromosome numbers. The S_1 tetraploid showed morphological variations in its flowers, cotton bolls, and fibres and was partially resistant to *Verticillium dahliae* Kleb. The S_1 tetraploid was hybridised with upland cotton to produce two types of cotton with different characteristics. This successful crossbreeding of S_1 provides direct evidence that interspecies hybridisation can facilitate the development of new types of cotton. This newly synthesised tetraploid cotton species is a valuable resource for cotton resistance breeding and for the enrichment of cotton genetic resources.

Keywords: Genetic evolution, *Gossypium*, Interspecific hybridisation, Synthetic allotetraploid.

Introduction

Cotton (genus, *Gossypium*; family *Malvaceae*) is the most important natural raw material in the textile industry. Verticillium wilt is a disease that causes severe yield losses in cotton growing areas [1]. The pathogen causing this disease is a soil-borne fungus, *Verticillium dahliae* Kleb., which was first reported in Virginia, USA, in 1914 [2]. This disease has now spread to most of cotton growing areas worldwide, and there are no effective prevention and control methods available. Currently cultivated upland cotton lacks resistance to verticillium wilt, whereas wild cotton remains highly resistant to this disease [14]. Thus, this study aimed to identify and isolate the source of resistance in wild cotton by interspecies hybridisation.

Remote hybridisation has long been known to be an important means for studying the evolution of species and for the artificial synthesis of new species. There are 55 species of the genus *Gossypium* worldwide, including 48 diploids (2n = 2x = 26) and 7 tetraploids (2n = 4x = 52) [3,4]. Over the decades, a number of interspecies hybrids have been successfully produced, including *G. arboreum* L. × *G. raimondii* UIbr. [5], *G. hirsutum* L. × *G. sturtianum* Willis [6], *G. arboreum* L. × *G. bickii* Prokh. [7], *G. hirsutum* L. × *G. klotzschianum* Anderss. [8], *G. hirsutum* L. × *G. trilobum* Skov. [9], *G. arboreum* L. × *G. anomalum* Wawra & Peyr. [10], *G. hirsutum* L. × *G. anomalum* Wawra & Peyr. [11], and *G. herbaceum* L. × *G. australe* F. Muell. [12]. However, to our knowledge, there are no reports of an allotetraploid ($A_1A_1C_4C_4$) that has been formed from a cross between *G. herbaceum* L. and *G. nelsonii* Fryx.

The cultivated diploid cotton species *G. herbaceum* L. (2n = 2x =26, A_1A_1) originated in Africa whereas the wild diploid cotton species *G. nelsonii* Fryx. (2n = 2x = 26, C_4C_4) originated in Oceania. *G. herbaceum* L. and *G. nelsonii* Fryx. has excellent drought tolerance and are resistant to *Verticillium dahliae* Kleb. Due to interspecies isolation, it has been difficult for the two species to intercross under natural conditions. Thus, in this study, we crossed *G. herbaceum* L. (♀) with *G. nelsonii* Fryx. (♂) to produce an interspecies F_1 hybrid. We grafted the F_1 hybrid plants to obtain 100 clones and doubled its chromosomal content using colchicine to successfully obtain a synthetic tetraploid, which we designated S_1 (2n = 4x = 52, $A_1A_1C_4C_4$). We

characterised this new S_1 cotton germplasm by performing morphological analyses, polymorphism detection, and ploidy analysis (by flow cytometry and microscopic observation of chromosome numbers). The assessment of the disease resistance of the S_1 tetraploid and its application in further hybridisation experiments are also reported.This newly germplasm can enrich the genetic resources of cotton and provide important materials for disease resistance breeding of cotton.

Materials and Methods

Plant materials

The cotton varieties used in this study were *G. herbaceum* L. (♀), *G. nelsonii* Fryx. (♂), Cotton cv. *Jimian11,* and cotton TM-1. The diploid *G. herbaceum* L. of the cultivated species ‘Hongxing Caomian’ (2n = 2x = 26, A_1A_1) was used as the female parent and the diploid *G. nelsonii* Fryx. (2n = 2x = 26, C_4C_4) was used as the male parent for interspecies hybridisation. Pure *G. herbaceum* L. seeds were obtained by strict self-pollination for 40 generations. Pollen from *G. nelsonii* Fryx. was collected from the China National Wild Cotton Nursery in Sanya, China [13]. Cotton cv. *Jimian11,* the standard susceptibility control for the national identification of cotton varieties in China, was used in experiments to identify new resistant synthetic allotetraploids. Upland cotton TM-1 was the female plant source used for crossbreeding at the final experimental stage of this study.

Interspecies hybridisation

Distant hybridisation was carried out in Sanya, China, in the winter of 2015. The female flowers were removed from *G. herbaceum* L. (♀) and the stigmas covered with a wax tube to avoid cross-pollination during flowering the following day. The following day, pollen from the male *G. nelsonii* Fryx. parent was collected and used to pollinate the female parent between 9 a.m. and 12 noon. Flowers that have been pollinated were marked. More than a month later, the hybrid seeds were harvested from the female parent plant and planted in a nutrient bowl. On April 20, 2016, when the seedlings were two weeks old, they were transplanted into a field at Sanya, China [13,14].

Clonal propagation and colchicine treatment

Only very few hybrid seeds were obtained by interspecies hybridisation, and the F_1 hybrid was sterile. To provide sufficient experimental materials for chromosomal doubling, the F_1 hybrid generation was cloned by grafting and preserved in Sanya, China on July 15, 2016. Chromosome doubling experiments were conducted under natural field conditions, on selected original or cloned plants with a height of 50 cm. Approximately 2 mm was removed from the apical growth point of these plants while retaining the apical tender branches. Colchicine (0.3%) was dipped into the cotton balls, which were wrapped around the top of the tender branches, and covered with plastic bags to prevent evaporation. After 6 hours of treatment, the plastic bags and cotton balls were removed. Five days later, the leaves were sprayed with a solution of potassium dihydrogen phosphate (at a concentration of 1,000 times), once every 7 days, until new shoots grew. Throughout the experiment, side branches that did not receive both treatments were removed [13]. On October 15, 2016, the treated plants were transferred to seedling pouches to grow under natural field conditions in Sanya, China.

Morphological analyses

The morphological characteristics of parents, hybrids, and tetraploid plants were analysed and recorded as previously described [14]. Information about the stems and leaves, plant height, and plant type during the vegetative growth period was recorded. The shape and colour of the flowers, calyxes, and bracts, the colour of the filaments, anthers and pollen, and the features of the flower base, the shape and colour of fruits in boll stage, and the shape and colour of fibres in cotton boll production stage were also recorded. A Canon digital camera (EOS 70D) set at RF 35mm F1.8 MACRO was used to photograph the various tissues shapes of cotton. Adobe Photoshop 7.0 software from Adobe company was used to edit the morphological images of cotton.

Polymorphism identification by the SSR molecular marker

Plant genomic DNA from the F_1 hybrid, tetraploid S_1 and their parents was extracted according to a previously published method with minor modifications **[**14**]**. Plant DNA was extracted from young leaves near the growth point and genomic DNA was

quantitatively and qualitatively analysed by agarose gel electrophoresis. Six SSR primers were selected for PCR amplification (Table 4), which was performed in a DNA Mastercycler (Eppendorf). The PCR reaction components and optimised reaction conditions were based on previous literature [13]. PCR products were resuspended in double distilled water and analysed by agarose gel electrophoresis. The gels were photographed using a Canon digital camera (EOS 70D) and imaged edited in Adobe Photoshop 7.0 software.

Ploidy identification by flow cytometry

Selected young leaves that are 1 cm long and 1 cm wide were cut into small pieces with a razor blade and mixed with 1 ml of Galbraith buffer (45 mM $MgCl_2$, 30 mM Sodium Citrate, 20 mM 3-(N-morpholino) propanesulfonic acid [MOPS], 0.1% (v/v) Triton X, pH=7 [16]) before filtering through a 50 micron nylon mesh. The filtrate was centrifuged at 1,000 xg for 5 min to pellet the cells. Propidium iodide solution (200 ml of a 50 mg/l solution) was added to the cells, which were incubated in the dark for 20 min [39]. DNA content was analysed by flow cytometry (Sysmex-Partec CyFlow® Cube 8) with excitation set at 488 nm. Diploid *G. herbaceum* L. was used as the external reference standard (code: 2C). Samples suspected to be diploid (*G. herbaceum* L. × *G. nelsonii* Fryx. F_1, code: 2C) and those suspected to be tetraploid (*G. herbaceum* L. × *G. nelsonii* Fryx. S_1, code: 4C) were mixed prior to testing. One diploid peak indicated that both tested plants were diploid; whereas the presence of two peaks at a 1:2 ratio indicated the presence of a diploid and a tetraploid plant. Adobe Photoshop 7.0 software was used to edit and process the images detected by flow cytometry.

Microscopic observation of chromosome numbers

When the hybrid began to germinate and the root tip grew to 2 cm long, approximately 5 mm of the tip was cut off between at 9:00 am to 10:00 am, and the apex was immersed into a saturated solution of p-dichlorobenzene at room temperature for 6 h. The treated materials was rinsed twice with distilled water and immediately fixed in freshly prepared Carnoy's fluid (ethanol: acetic acid at 3:1 v/v [13]). After 24 h, the root tip was rinsed with distilled water and cut into pieces in 1 M of hydrochloride. Two root tips were dispensed in each 1.5 ml centrifuge tube and

dissociated at 60°C in a water bath for 10-15 min. The separated root tips were then stained with Carbol fuchsin solution on the slide, and identified by microscopy. Micrographs of cells and chromosomes were collected using German Leica biological microscope DM2500 and camera system MC170. Adobe Photoshop 7.0 software was used to edit the pictures of cells and chromosomes.

Identification of disease resistance

The Dahlia Vd080 strain that causes cotton fusarium blight was obtained from the Cotton Research Institute of the Chinese Academy of Agricultural Sciences. The pathogen strain was frozen in 25% glycerin (v/v) at -80°C for long-term preservation, and the strain was activated on potato dextrose agar (PDA) culture medium before inoculation. The pathogen was cultured in PDA at 28°C for 7 days to obtain the strain culture solution (conidia solution) according to previous reports[18]. The final concentration was adjusted to 10^6 conidia/ml with sterile distilled water. The S_1 allotetraploid was tested using upland Cotton *Jimian11*, which was susceptible to *Verticillium dahliae* Kleb. as controls. Each variety was divided into three treatment groups of thirty plants per group, with six bowls for each . Commercial vermiculite and sun-dried river sand were used as substrates to prepare bottomless paper bowls. Plantlets at the five-leaf stage were supplied, and the roots were inoculated with a spore suspension (10^6 conidia/ml) to induce cotton verticillium wilt. The cotton seedlings were planted under controlled greenhouse conditions to observe the pathogenesis of verticillium wilt. Disease was classified and indexed according to a previously published ranking system as follows[19]: Rank zero: healthy; Rank 1: disease occurred on one or two cotyledons; Rank 2: disease occurred on one true leaf, but terminal bud was healthy; Rank 3: disease occurred on more than two pieces of true leaves, but the terminal bud remained healthy; Rank 4: the entire plant was morbid and/or dead. The incidence and disease index were calculated based on the survey results. All cotton varieties were photographed with a Canon digital camera (EOS 70D) [20]. Microscopic observation and image acquisition of pathogenic bacteria spores and hyphae were carried out using a biological microscope (Leica DM2500 with an MC170 camera system) and edited in Adobe Photoshop 7.0 software.

Crossbreeding experiments

In the winter of 2017, the hybridisation of the upland cotton TM-1 (genome AADD) and the newly synthesised allotetraploid S_1 ($A_1A_1C_4C_4$) was carried out in Sanya, China. The flowers of upland cotton TM-1 were removed and the stigmas covered with a wax tube to avoid cross-pollination by pollen from other plants. Between 9 a.m. to 12 noon. the following day, allotetraploid S_1 plants were was selected to pollinate the female upland cotton TM-1 and marked. After at least one month, hybrid seeds could be obtained from upland cotton TM-1. The seeds were planted in a nutrient bowl and transplanted to the field two weeks later, on April 20, 2017. All cotton varieties were photographed with a Canon digital camera (EOS 70D) and edited in Adobe Photoshop 7.0 software.

Results

Morphological analysis of the diploid F_1 hybrid and the synthetic S_1 tetraploid

Production of the F_1 hybrid. The breeding of the F_1 hybrid was initiated from artificial hybrid bolls. One boll per plant containing three seeds was obtained. After sowing, all three hybrid plants germinated and developed. The appearance and morphology of the three F_1 hybrids were similar, with broad leaves. On average, each boll from the hybrid plants contained one to three seeds. All three F_1 hybrid plants were infertile at later growth stages and did not bear fruit. This phenomenon was not unexpected and was consistent with the law of distant hybrid infertility, as these plants were the offspring of interspecies hybridisation. Plants presumed to be true hybrids were propagated by grafting. More than 100 plants were grafted to prevent the loss of the interspecies hybrid.

Production of the S_1 tetraploid. We crossed *G. herbaceum* L. (♀) (Fig. 2A)with *G. nelsonii* Fryx. (♂)(Fig. 2B) to produce an interspecies F_1 hybrid.The stem apices of grafted plants F_1 were treated with 0.3% colchicine for 6 h during the squaring stage. Sixty-one days later, we were surprised to find that one of the grafted plants had produced a large pink flower with abundant yellow pollen (Fig. 1A), which we designated S_1. Five days after the flowers died, an ovate boll grew, ripened, and split, to produce four seeds with brown fibres (Fig. 1B, 1C).The S_1 tetraploid was produced from seeds of the fertile F_1 plant(Fig. 2C) described above. Four plump seeds were sown, one of which germinated successfully and grew into an adult plant (Fig. 2D).

This S_1 plant was shorter than the F_1 parental plant, with thicker leaves, petals, and calyxes compared to its F_1 parent. Importantly, this S_1 tetraploid was fertile and produced a large number of bolls. Therefore, it is likely that the chromosomal content of the newly grown S_1 tetraploid had successfully doubled. In contrast, the F_1 plant produced only one boll, and only once. These observations suggest that successful doubling of the cotton chromosome did not happen very often and that the diploid F_1 might have been successfully transformed into a new derivative by colchicine treatment. The genome of the new synthetic tetraploid S_1 was 2n = 4x = 52, $A_1A_1C_4C_4$.

Plant shape and growth, and flowering. After the plants had developed into adults, they were surveyed. *G. herbaceum* L., F_1, and S_1 had lower branches that were longer than the upper branches, giving the plants a tower-like appearance (Fig. 2A, 2C, 2D). In contrast *G*. nelsonii Fryx. had upper, middle, and lower branches of approximately the same length; these plants looked like long canisters (Fig. 2B). Similar to *G. herbaceum* L., both F_1 and S_1 exhibited prolific growth with extensive monopodial branching (Fig. 2). Flower buds of the F_1 plant appeared 40 days after planting (Fig. 3c). The *G. herbaceum* L, F_1, and S_1 flowered all year round (Fig. 3A, 3C, 3D), but the flowering time of *G. nelsonii* Fryx. was limited, from November in the first year to June in the second year (Fig. 3B). The S_1 flowers had corollas of different shapes, colours, and sizes (Supplementary Figure 1A-H). The shapes were cylindrical, funnel, bell, and semi-closed or closed; sizes were considered big, medium, and small, corresponding to 40, 25, and 15 mm in length, respectively; and the colours included light red, pink, and creamy white (Supplementary Figure 1A-H). The closed pollination in the S_1 generation was inherited from the male parents, because closed pollination often occurred when male parents were at low temperature, whereas it did not occur in the female parents (Supplementary Figure 1H).

Bolls and fibres. The diploid F_1 could not bear bolls because it was sterile. In contrast, the S_1 tetraploid was fertile and showed characteristics of precocious puberty; for example, the S_1 tetraploid that was sown in the field took 4 days to germinate, which was 2 days earlier than either parent. Sowing to flowering was completed in 40 days in the S_1 tetraploid, which was 5 days and 3 days earlier than that in the female and male parental plants, respectively. Subsequently, flowering to cotton boll opening in the S_1 occurred in 35 days, again, 5 days and 3 days earlier than that in the female and

male parental plants, respectively. Therefore, the S_1 tetraploid had a growth cycle of 99 days. The S_1 tetraploid had strong fruit production capacity (Supplementary Figure 2), producing up to 50 bolls per plant after 4 months of growth in the field (Supplementary Figure 2). Cotton bolls of various shapes, including pear-shaped, lemon-shaped, pebbly-shaped, and irregular shapes, were produced (Fig.4C). Some of these shapes were never produced by either parent; in particular, a pear-shaped boll with a sunken navel on its surface (Fig. 4C). These boll characteristics were significantly different from the female parents from which the fruit tips were raised. Additionally, the fruit surface of the S_1 tetraploid was covered with short fuzzy hair, a trait inherited from the male parent (Fig. 4C). The boll of the S_1 tetraploid contained three or four ventricles. In Sanya, China, the S_1 tetraploid bloomed and bore fruit all year round and was highly fertile. In summary, the S_1 tetraploid had strong boll-forming abilities, indicating that the plant has potential for high yield, which is an important agronomic factor that improves the yield of upland cotton.

The S_1 tetraploid produced open and unimpeded cotton bolls, containing up to 16 fertile seeds per boll. Its fibre colour was mainly inherited from the male parent including light brown, brown, and light green, whereas fibres of the female parents were white (Fig.4D,E,F). In the male parent plants, fibres grew upright (Fig. 4E) and were shaped like upright iron needles. In contrast, fibres of female parents grew in a spiral (Fig. 4D) and were shaped like fixed telephone lines. In contrast, the fibres of the S_1 tetraploid showed wavy growth and were shaped like human curls, representing an intermediate phenotype of both parents (Fig. 4F). In all species, hairs grew in the direction of the seed tip. The cotton bolls of the S_1 tetraploid were also diverse and included four-chambered bolls with brown fibres and three-chambered bolls with light green and brown fibres (Supplementary Figure 3A,B). The average fibre length of the S_1 tetraploid was approximately 13 mm, which was longer than those produced by male parents but shorter than those produced in female parents (Supplementary Figure 3C).

Comparisons of the biological traits of F_1 and S_1 with their parental plants. The biological characteristics of the parents, F_1 and S_1 are detailed in Tables 1,2. In the fields of Sanya, China, the female *G. herbaceum* L. parent grows for up to 3 years. After the first year of growth, the plant reaches approximately 80 cm in height (Fig.

2A), and the main stem reaches approximately 1.5 cm in diameter. *G. herbaceum* L. is tower-shaped with short and compact branches. It bears bell-shaped flowers with yellow corollas, pink basal patches, pink filaments, yellowish anthers, yellow pollen, a cup-shaped calyx, heart-shaped bracts that grow separately rather than together, and is broad-leaved. The female *G. herbaceum* L. produces smooth conical bolls that are green and red in colour (Fig. 4A). There is a distinct conical bulge at the top of the boll, and the bolls are 3-chambered with grey-white spiral cotton fibres. The male *G. nelsonii* Fryx. parent also grows for 3 years. After the first year of growth, the plant reaches approximately 200 cm in height (Fig. 2B), and the main stem grows to approximately 2 cm in diameter. *G. nelsonii* Fryx. is cylindrical with short and compact branches, bears tubular-shaped flowers with pale pink corollas, purple basal spots, red filaments and anthers, milky pollen, a wavy calyx, sword-shaped bracts that are detached rather than united, and has ovoid leaf blades. The male *G. nelsonii* Fryx. parent plant produces oval, green cotton bolls with fine hair on the surface. There are no distinctive features on the boll tips (Fig. 4B), and the bolls are 3-chambered with dark brown upright fibres (Fig. 4E).

After the first year of growth, the diploid F_1 plant reached 300 cm in height (Fig.2C) and was taller than either parent plant. Its main stem diameter grew to 3.0 cm, also wider than either parent. It had longer, loosely spaced branches, but was infertile (Tables 1, 2). Most of the biological characteristics of the F_1 plants were inherited from the female *G. herbaceum* L. parent, including a tower-like appearance, trumpet flowers, green main stems, leaves, and petioles, yellow anthers, and pollen. The leaf length and width, detached bracts, and column length were also similar to the female parent. A few traits were inherited from the male *G.nelsonii* Fryx. parent, including pink corollas, light pink filaments, and medium-sized spots on the petal base. Some traits were inherited from both parents and of an intermediate phenotype; these included a serrated calyx, sword-shaped bolls, red spots on the petal base, lobated leaves, and teeth-like bracts. Some features of the F_1 hybrid were not observed in either parent, including production of taller plants, larger main stems, more fuzzy stems and leaves, long and looser branches, and lobate leaf lobes. Some leaves of the F_1 were broad-leaved, oval, and semi-circular in shape, displaying leaf characteristics of both parents.

After successfully doubling of the growth points of the diploid F_1 hybrids with colchicine, the plants obtained were designated the S_1 tetraploid generation. The S_1 plants had restored fertility and could produce bolls. After the first year of growth, S_1 plants reached 150 cm in height (Fig. 2D), which was an average of both parents. Its main stem diameter reached 2.5 cm, which was larger than either parent, and it produced longer, more loosely spaced branches. S_1 plants showed some biological traits that were distinct from the F_1 hybrid. They were shorter, and their leaves were thicker and greener. The leaves were broad-leaved, ovoid or semi-circular in shape, and the flowers were trumpet-, funnel-shaped, cylindrical, closed, or half closed. The colours of the corollas were pink, light pink, milky white, red, and white, simultaneously displaying characteristics of both parents and the F_1 parents. Some biological characteristics of the S_1 generation were inherited from the female *G. herbaceum* L. parent, and were similar to the F_1 hybrid, namely, tower-like plants, green main stems and petioles, yellow anthers, and pollen. A few traits were inherited from the male *G. nelsonii* Fryx. parent, namely, serrated calyx and green stems and bolls. Some traits were intermediate of both parents, namely plant height, leaf length and width, triangle dentate bracts, ensiform bracts, and light green and brown fibres. Some features of the S_1 generation showed new variations beyond their parental phenotypes, such as boll shape, wavy fibres, the colour and size of the spot on the petal base, fuzzier stems and leaves, longer and looser branches, lobate leaf lobes, and column length. In particular, the bolls of S_1 were pear-shaped, with sunken navels, oval, irregular, or round. The column length was long, medium, or short. The size of the petal base spot was medium or small, and it produced some branches but had no basal plaques.

Polymorphism detection in diploid F_1 and tetraploid S_1 using simple sequence repeat (SSR) molecular markers

To better understand the authenticity of hybrids and their molecular differences compared to their parents, SSR analysis was performed using six SSR primer pairs (Table 4) and all yielded microsatellite products. Among these products, the bands amplified by primers NAU1157, NAU1355, NAU1052, JESPR156, BNL1679, and NAU3093 in the F_1 and S_1 hybrids showed complementary contributions from both parents (Fig. 5A). In addition to bands that were inherited from the parents, novel

bands in the F_1 and S_1 hybrid plants were amplified by primers NAU1052 and NAU3093 (Fig.5Ac,f), indicating that mutations and chromosomal recombination had occurred between the parental genomes during hybridisation. The other three primers did not amplify new bands (Fig.5Aa,b,d,e). In summary, both F_1 and S_1 were confirmed as true hybrids by field morphological observation and by SSR molecular marker identification.

Validation of the synthetic allotetraploid S_1 by flow cytometry and microscopy

The flow cytometry test results showed that the peak threshold value of the female of *G. herbaceum* L (External standard reference) was at approximately 200 channels (Fig. 5B), and the peaks of the mixed diploid F_1 and tetraploid S_1 were at approximately 200 and 400 channels respectively (Fig. 5C). The results showed that the F_1 was diploid, which was the same as the *G. herbaceum* L. reference, and that S_1 was tetraploid.

By microscopic observation, 52 chromosomes were counted in the root tips of S_1 cells in the middle stage of mitosis (Fig. 5D, black arrow), indicating that the F_1 was diploid (i.e. had 26 chromosomes; Fig. 5E, black arrow) and had successfully doubled into tetraploids after colchicine treatment.

Preliminary identification of resistance to *Verticillium dahliae* Kleb. in S_1 tetraploids

Cotton seedlings were inoculated with the fungal pathogen (*Verticillium dahliae* Kleb.) (Fig. 6A, black arrow) in the greenhouse. The susceptible control, upland cotton *Jimian-11*, had a large number of yellow leaves 7 days after inoculation, and a large number of leaves fell off the plant 25 days after inoculation, followed by death (Fig. 6B, red arrow). In contrast, the S_1 tetraploid and its parents that had been inoculated with the same pathogen (Fig. 6C,D,E) showed no yellowing and shedding of leaves. No dead seedlings were found throughout the experimental growth period that lasted 60 days. Therefore, according to the previously published “disease classification” and “disease index” system [41]the disease level of the control group was ranked level 4. Both S_1 and its parents had a disease ranking level of 0, which mean that the plants

were resistant to the disease. Disease resistance of the S_1 tetraploid was inherited from the male parent.

Crossbreeding of the S_1 allotetraploid

The production of F_1-1 and F_1-2 hybrids. To transfer favourable resistance genes to upland cotton and to determine whether disease resistance of the S_1 (♂) could be preserved upon breeding, we hybridised it with upland cotton TM-1 (♀). The female bolls of TM-1 produced by natural pollination were not deformed (Fig. 7A), but most of the hybrid bolls were malformed, and the bolls were not full (Fig. 7B,C,D), producing only a small amount of seeds. On average, only two to five seeds were obtained per boll. Interestingly, after a strict hybridisation, two seeds that were obtained from a hybrid boll grew into different types of plants in the later stage, including one with red flowers and another one with white flowers (designated F_1-1 and F_1-2, respectively, Fig. 8C, 9D).

Biological traits. The biological traits of F_1-1 and F_1-2 and the parental plants are summarised in Tables 3. The female cotton TM-1 parent was tower-like with longer and looser branches (Fig. 8A) and bore bell-shaped flowers with milky corollas, milky filaments, milky anthers and pollen, without basal patches, a cup-shaped calyx, heart-shaped bracts of united growth, and was broad-leaved (Fig. 8A). Some male S_1 plants were tower-like, with shorter and more compact branches (Fig. 8B) and had bell-shaped flowers with pink corollas, milky filaments, yellow anthers and pollens, red basal patches, a serrated calyx, sword-shaped bracts that grew separately rather than together, and heart-shaped or broad leaves (Fig. 8B).

The F_1-1 and F_1-2 shared some characteristics but not all (Tables 3; Fig. 9C, 9D). The shared traits included tower-like plants, broad leaves, trumpet flowers, looser branches, and green stems and petioles that simultaneously display characteristics of both parents. However, F_1-1 was more similar to the male parent and F_1-2 was more similar to the female parent. The biological characteristics of the F_1-1 that were inherited from the male parent included fuzzy stems and leaves, a serrated calyx, shorter branches, pink corollas, red petal base spots, yellow anthers and pollen, and bracteoles that grew separately. The biological characteristics of F_1-2 that were inherited from the female TM-1 parent included longer branches, stems, and leaves

that were not covered with fuzz, a wavy calyx, heart-shaped bract of united growth, white and red corollas, no petal base spots, and milky anthers and pollen.

Specific and major differences between F_1-1 and F_1-2 were found in the flower colours, bolls, and plant fuzziness. F_1-1 had pink flowers, which were similar to the parental S_1 (Fig. 9C), whereas F_1-2 had white flowers, similar to the female TM-1 parent, although its pollen grains were larger (Fig. 9D). F_1-1 only bore small numbers of cotton bolls in the wild after artificial pollination, and there were no seeds and no fibres in the bolls. The stem of the F_1-1 was fuzzy during the seedling stage; in larger seedlings, fuzz covered the stems and leaves. The surfaces of the F_1-1 bolls were also marked by a small amount of fuzz, a trait that is inherited from the male S_1 (Fig. 9F, 9G). In contrast, the stem of F_1-2 was not fuzzy throughout the seedling stages. F_1-2 also produced non-fuzzy cotton bolls in the wild, a trait inherited from the female TM-1 parent (Fig. 9E,H).

To summarise, the two F_1 hybrids from the same parent showed significant morphological differences, indicating that complex genetic recombination events had occurred during distant hybridisation of two different species. This is an advantage of distant hybridisation, which produces hybrid progeny that provide abundant intermediate materials for cotton breeding, and also expanded the resources available for cotton genetic research.

It should be noted that we have only preliminarily assessed the authenticity of the F_1 generation obtained from crosses between TM-1 and S_1 through morphological characterisation. Further studies are required to confirm they are true hybrids using cytology and molecular genetics techniques.

Discussion

Wild cotton varieties are a great treasury of improved cultivars [21]. There are at least 30 varieties of wild cotton, widely distributed in the Americas, Australia, Africa, and Asia that have undergone long-term natural selection to accumulate rich genetic diversity conferring adaptability to adverse conditions and resistance to environmental onslaughts and diseases. The cultivated varieties present today have also undergone a long period of artificial selection, to significantly improve yields and

fibre quality compared to wild varieties. However, even after long-term intensive farming and artificial selection, some favourable resistant phenotypes have not developed in cultivated varieties, and were even were excluded by excessive selection. Therefore, despite the continuous improvements in yields and fibre quality, their disease resistance had weakened. Resistance breeding programmes are now being conducted to transfer the disease resistance genes from wild cotton to cultivated cotton, and to re-diversify the genetic makeup of cultivated cotton.

The superior qualities of cotton fibre, including its length, strength, fineness as well as disease resistance of wild cotton has been successfully transferred to cultivated cotton [22-24]. However, diploid wild cotton and tetraploid cultivated cotton are biologically isolated. While increased genetic diversity could be achieved from distant hybridisation, they are often genetically incompatible, forming a barrier to full utilisation of wild diploid cotton varieties [25-28]. In the present study, we successfully obtained an infertile interspecies F_1 hybrid through distant hybridisation, overcoming the genetic incompatibility barriers between two distant species. We further doubled the chromosomal content of this F_1 hybrid with colchicine treatment to overcome the significant challenge of interspecies hybrid sterility, to successfully synthesise a new tetraploid cotton species, S_1 ($A_1A_1C_4C_4$).

Hybrid separation still occurred in the subsequent S_1 generations. We found that the offspring of S_1 gradually exhibited growth decline in the field. The S_2 generation could still grow normally, blossom, and bear fruit, but the S_3 and S_4 generations had limited growth and flowered less. Plants of the S_5 generation had severely restricted growth and could not blossom, indicating that S_1 was a genetically unstable germplasm that carried the risk of natural separation. The genetic instability of S_1 severely hindered the utilisation of new cotton germplasm for breeding. We were unable to resolve this issue in the present study; thus, the control of hybrid separation for successful establishment of stable genetic strains needs to be explored in future research.

The S_1 generation had high potential for breeding due to its boll-forming properties, diversity of fibre colours, and fibre quality. Boll formation is the key intrinsic factor that determines cotton yield. After 4 months of growth in the field, the number of bolls per S_1 plant reached 50 bolls per plant, an important characteristic for high yield

breeding applications. We also found that a variety of coloured fibres developed in S_1 cotton bolls, including brown fibres in a 4-chambered boll and light green and brown cotton fibres in a 3-chambered boll (Supplementary Figure 3A,B). These observations indicated that the fibre characteristics of S_1 were recessive and dynamic. Notably, the fibre Micronaire reading (A measure of air permeability of compressed cotton fibres) of the S_1 fibres was 9.6, determined using the Macron Airflow Meter in the fibre-testing centre of the Cotton Research Institute of Chinese Academy of Agricultural Sciences. This reading was far above the parental range and may be the highest value reported to date. The S_1 fibres were very thick and provided us with the raw materials for making non-woven fabrics. These traits are also of great value in breeding programmes.

Molecular markers are specific pieces of DNA that reflect differences in the genomes of individuals or populations [29]. Since the discovery that microsatellite markers are uniformly distributed on cotton chromosomes, SSR markers have been used to monitor the infiltration of heterologous chromosome fragments in the offspring of interspecies hybrids [30,31]. In the present study, SSR detection revealed the presence of new specific bands in hybrids (Fig. 5Ac,f) that were not detected in the parents, indicating that new combinations or mutations of chromosomes are present in the interspecies progeny (Genomic A and Genomic C). Our results also support the hypothesis that distant hybridisation is an important driver of new mutations and species in plant evolution [32,33].

There is potential for genes from the new synthetic allotetraploid to fuse with favourable genes from *G. herbaceum* L. and *G. nelsonii* Fryx., including genes that encode good fibre quality, or resistance to diseases, pests, and drought. This will allow their transfer them to upland cotton through hybridisation, to further improve the resistance and fibre quality of upland cotton [34]. We tested this possibility by performing preliminary disease-resistance testing of the new S_1 species in the greenhouse, and found that the new species presented high disease resistance to the fungal pathogen *Verticillium dahliae* Kleb. When the new species were transplanted to cultivated land where disease occurs all year round, we confirmed that the new species also showed high disease resistance to wilt verticillium in the field. This

important agronomic character provided a new and important resource for cotton disease resistance breeding programmes.

When we examined whether the newly synthesised allotetraploid S_1 could be applied in cotton breeding by hybridisation with upland cotton TM-1, we found two types of plants in the F_1 hybrid offspring, namely F_1-1 with red flowers and F_1-2 with white flowers. F_1-1 plants died upon onset of anthesis, indicating that this plant type could not be applied in crossbreeding. F_1-2 plants grew normally until flowering and fruiting, but their seeds could not germinate normally, indicating that this type had the potential for breeding applications but additional research is required to overcome key difficulties in promoting seed germination each generation. This phenomenon of unsuccessful germination of F_1-2 seeds and root tip wilting had previously been observed during the early generation of the amphiploid *G. arboretum* L. hybridised with *G .bickii* Prokh. [35]. The variation in hybrid phenotypes may be related to gene overlap, new genetic variation, chromosome rearrangement, or a variety of other factors [36-39].

Thus far, we have only performed preliminary characterisations of the newly synthesised allotetraploid cotton ($A_1A_1C_4C_4$). We propose to perform more in-depth and comprehensive research to extract valuable information from this new cotton tetraploid, so as to provide a more accurate theoretical basis for cotton breeding and genetic evolution.

References

1. El-Zik,KM.Integrated control of Verticillium wilt of cotton. Phytopathology. 1985;6,1025e1032.
2. Carpenter,CW.The *Verticillium* wilt problem.Phytopathology. 1914;4,393.
3. Wang,K.,Wendel,J.&Hua,J.Designations for individual genomes and chromosomes in Gossypium.J.Cotton.Res. 2018;1,3;10.1186/s42397-018-0002-1 .
4. Fryxell,P.A revised taxonomic interpretation of *Gossypium* L.(Malvaceae).Rheedea.1992;2(2),108-65.
5. Endrizzi,J.E.,Phillips,L.L.A hybrid between *Gossypium arboreum* L.and *G.raimondii* Ulb.Can.J.Genet Cytol.1960;2(4),311-319.
6. Muramoto H.Hexaploid cotton: some plant and fiber properties.Crop Sci. 1969;9(1),27-9.
7. Li B,Zhang B,Zhang X,Niu Y.Studies on the Hybrid between *G. arboreum* L and *G.bickii* Prokh.Acta Genetica Sinica. 1987;14(2),121-6.
8. Sun,Y.et al.Production and characterization of somatic hybrids between upland cotton (*Gossypium hirsutum*) and wild cotton (*G.klotzschianum* Anderss) via electrofusion.Theor.Appl.Genet. 2004;109(3),472-479.
9. Yu,X.et al.Characteristics of fertile somatic hybrids of *G.hirsutum* L.and *G.trilobum* generated via protoplast fusion.Theor.Appl.Genet. 2012;125(7),1503-1516.
10. Newaskar,G.S.,Chimote,V.P.,Mehetre,S.S.,Jadhav,A.S. Interspecific hybridization in *Gossypium* L.:characterization of progenies with different ploidy-confirmed multigenomic backgrounds.Plant Breed. 2013;132(2),211-216.
11. Zhang,X.et al.Morphological,cytological and molecular analyses of a synthetic hexaploid derived from an interspecific hybrid between *Gossypium hirsutum* and *Gossypium anomalum*.The Crop Journal. 2014;2(5),272-7.
12. Liu,Q.et al.A New Synthetic Allotetraploid (A1A1G2G2) between *Gossypium herbaceum* and *G. australe*:Bridging for Simultaneously Transferring Favorable Genes from These Two Diploid Species into Upland Cotton.PLoS One. 2015;10(4), e0123209;10.1371/journal.pone.0123209.
13. Wu,Y.,Chen,D.,Zhu,S.,Zhang,L.,Li,L.A New Sythetic Hybrid(A1D5) between *Gossypium herbaceum* and *G. raimondii* and Its Morphological, Cytogenetic, Molecular Characterization. PLoS One. 2017;12(2),e0169833;10.1371/journal.pone.016983 .
14. Chen,D.,Wu,Y.,Zhang,X.,Li,F.Analysis of [*Gossypium capitis-viridis*×(*G.hirsutum*× *G.australe*)^2]Trispecific Hybrid and Selected Characteristics.PLoS One. 2015;10(6),e0127023;10.1371/journal.pone.0127023.
15. Paterson,A.H.,Brubaker,C.L.,Wendel,J.F.,A rapid method for extraction of cotton(*Gossypium* spp.)genomic DNA suitable for RFLP or PCR analysis.Plant.Mol.Biol.Rep. 1993;11(2),122-127.
16. Galbraith,D.W.,et al.Rapid flow cytometric analysis of the cell cycle in intact plant tissues.Science. 1983;220,1049-1051.
17. Zhang,X.H.,et al.Interspecific hybridization, polyploidization and backcross of *Brassica oleracea var. alboglabra* with *B. rapa var.purpurea* morphologically recapitulate the evolution of Brassica vegetables.Sci. Rep. 2016;10.1038/srep18618.

18. Yin,X.M.,et al. Characterisation of Early Events in Banana Root Infected with the GFP-tagged *Fusarium oxysporum* f. sp. *cubense*. Acta Hortic. 2011;(897),371-376.
19. Ping,M.A.,HUANG,H.C.,LI,S.Z.,TANG,W.H.A new inoculation method for verticillium wilt on cotton and its application in evaluating pathogenesis and host resistance.Acta Phytopathol. Sin. 2004;34(6),356-541.
20. PENG,S.,et al.Study on a new rapid inoculation method for Verticillium wilt and fusarium wilt of cotton.J.Cotton Sci. 2008;20(3),174-178.
21. Endrizzi,J.,Turcotte,E.,Kohel,R.Genetics,cytology and evolution of *Gossypium*.Adv.Genet. 1985;23,271-375.
22. Campbell,B.,Williams,V.,Park,W.Using molecular markers and field performance data to characterize the Pee Dee cotton germplasm resources.Euphytica. 2009;169,285-301.
23. Culp,T.,Harrell,D.Breeding methods for improving yield and fiber quality of upland cotton (*Gossypium hirsutum* L.).Crop Science. 1973;13,686-689().
24. Qian,S.et al.Studies on the hybrid of *G.hirsutum* L.and *G.anomalum* Wawr.& Peyr.and application in breeding.Sci.Agric.Sinica. 1992;25,44-51.
25. Oakes,A.J.A Sterility in certain *Gossypium* hybrids.I. Prefertilization phenomena.Can.J.Genet Cytol. 1966;8(4),818-829.
26. Sacks,E.J.Ovule rescue efficiency of *Gossypium hirsutum*× *G. arboreum* progeny from fieldgrown fruit is affected by media composition and antimicrobial compounds.PCTOC. 2008;93,15-20.
27. Ganesh,R.S.,Hari,R.S.,Thiruvengadam,V.,Kannan,B.J.Prefertilization barriers to interspecific hybridization involving *Gossypium hirsutum* and four diploid wild species.Plant Breed. 2008;127,295-300.
28. Li,B.L.,Zhang,B.J.,Zhang,X.R.,Niu,Y.Z.Studies on the Hybrid between *G.arboreum* L and *G.bickii* Prokh.Acta Genetica Sinica. 1987;14,121-126.
29. Benbouza,H.,et al.Introgression of the low-gossypol seed & high-gossypol plant trait in upland cotton:Analysis of [(*Gossypium hirsutum*×*G. raimondii*)×*G.sturtianum*] trispecific hybrid and selected derivatives using mapped SSRs.MOL BREEDING. 2010;25(2),273-86.
30. Siu,L.,Saha,S.,Stelly,D.,Burr,B.,Cantrell,R.Chromosomal assignment of microsatellite loci in cotton.J. Hered. 2000;91(4),326-32.
31. Barton,N.H.,The role of hybridization in evolution.Mol.Ecol. 10(3),551-68(2001).
32. Jiao,Y.,et al.Ancestral polyploidy in seed plants and angiosperms. Nature. 2011;473(7345),97-100.
33. Soltis,P.S.,Soltis,D.E.The role of genetic and genomic attributes in the success of polyploids.Proc Natl Acad Sci. 2000;97(13),7051-7057.
34. Chen,Y.,et al. A new synthetic amphiploid (AADDAA) between *Gossypium hirsutum* and *G. arboreum* lays the foundation for transferring resistances to Verticillium and drought.PLoSOne. 2015;10(6),e0128981;10.1371/journal.pone.0128981.
35. Kim,N.Z.,et al.Altered circadian rhythms regulate growth vigour in hybrids and allopolyploids.Nature. 2009;457(7227),327-331.
36. Kashkush,K.,Feldman,M.,Levy,A.A.Gene loss,silencing and activation in a newly synthesized wheat allotetraploid.Genetics. 2002;160(4),1651-9.
37. Rieseberg,L.H.Evolution:replacing genes and traits through hybridization.Curr. Biol. 2009;19(3),119-22.

38. Rowe,H.C.,Rieseberg,L.H.Genome-scale transcriptional analyses of first-generation interspecific sunflower hybrids reveals broad regulatory compatibility.BMC Genomics. 2013;14,342.
39. Shaked,H.,Kashkush,K.,Ozkan,H.,Feldman,M.,Levy,A.A.Sequence elimination and cytosine methylation are rapid and reproducible responses of the genome to wide hybridization and allopolyploidy in wheat.Plant Cell. 2001;13(8),1749-59.

Acknowledgements

This research work was financially supported by the National Natural Science Foundation of China (No. 31704178) and Central Public-interest Scientific institution Basal Research Fund for Chinese Academy of Tropical Agricultural Sciences(No.1630092017004).

We are grateful to Dr Kunbo Wang and Dr Fang Liu of the Cotton Research Institute of Chinese Academy of Agricultural Sciences for kindly providing us pollen of *G. nelsonii* Fryx. to use at the National Wild Cotton Nursery, Sanya, China.

Author Contributions

X.M.Y. conceived this work, D.C. and X.M.Y. performed the experiments, R.L.Z. wrote the manuscript, B.Q.X., L.X.W., and Y.G. interpreted the data and figures, and S.S. and Y.D.H. reviewed the manuscript.

Additional information

Competing financial interests: The authors declare no competing financial interest.

Figure Legends

Figure 1. Growth and formation of the synthetic tetraploid, S_1. (A) Tetraploid plants with a large pink flower accompanied by yellow pollen. (B) The growth process of the oval boll. (C) Four seeds with brown fibres.

Figure 2. Pictures of parent and hybrid plants. (A) The female *G. herbaceum* L. parent had a tower shape with shorter and more compact branches. (B) The male *G. nelsonii* Fryx. was cylindrical with short and compact branches. (C) The diploid F_1 hybrid was tower-shaped, with longer branches and looser spacing but were infertile. (D) The tetraploid, S_1, were tower-shaped with long fruit branches and loose spacing. They were fertile.

Figure 3. Pictures of leaves and flowers. (A) *G. herbaceum* L. bore bell-shaped flowers with yellow corollas, pink basal patches, pink filaments, yellowish anthers, yellow pollen, cup-shaped calyxes, heart-shaped bracts that grow separately rather than together, and were broad-leaved. (B) *G. nelsonii* Fryx. bore tubular-shaped flowers with pale pink corollas, purple basal spots, red filaments and anthers, milky pollen, wavy calyxes, lanceolate bracts that were detached rather than united, and ovoid leaf blades. (C) The F_1 hybrid had bell-shaped flowers with alternating red and white corollas, red basal patches, pink filaments, yellow anthers and pollen, serrated calyxes, sword-shaped bracts that grow separately rather than together, and had broad-leaved, ovoid, and subcircular leaves. (D) The S_1 had trumpet and funnel-shaped flowers with alternating red and white corollas, red basal patches, light pink filaments, yellow anthers and pollen, serrated and wavy calyxes, triangle dentate and ensiform bracts that grew separately rather than together, and had broad-leaved, ovoid, and subcircular leaves.

Figure 4. Pictures of cotton bolls and fibres. (A) *G. herbaceum* L. had conical, smooth green and red bolls. The bulge at the top of the boll was distinctly cone-shaped. (B) *G. nelsonii* Fryx. had oval, green cotton bolls with fine hairs on the surface. The boll tip was not obvious. (C) Green pear-shaped boll of S_1 covered with downy hairs. (D) *G. herbaceum* L. had 3-chambered bolls with grey-white spiral cotton fibres. (E) *G. nelsonii* Fryx. had 3-chambered bolls with dark brown upright

fibres. (F) The S_1 had 3-chambered bolls with brown fibres, and the seed hairs grew upright.

Figure 5. Validation of S_1 by SSR polymorphisms,flow cytometry and microscopy. (A) Microsatellite loci identified by SSR primers including NAU1157(a),NAU1355(b),NAU1052(c),JESPR156(d),BNL1679(e),NAU3093(f).the novel bands produced in F_1 and S_1 are indicated by red and blue arrows, (1, *G. herbaceum* L.;2, *G. nelsonii* Fryx.;3, the F_1;4, the S_1).(B)Distribution peak of *G. herbaceum* L.at approximately 200 channels.(C)Distribution peak of the tetraploid S_1 at approximately 400 channels . Fluorescence intensities of propidium-iodide stained genomic DNA. (D)The 26 chromosomes in F_1 hybrid.(E)The 52 chromosomes in S_1 tetraploid.The root tip cells at the end of mitosis(red arrows) and the chromosomes(black arrows) stained with Carbol fuchsin solution under 40× microscope.

Figure 6. Identification of resistance to *Verticillium dahliae* Kleb. (A) The pathogen of the candida conidia is indicated with the black arrow. (B) Disease-susceptible upland cotton *Jimian11*, with disease symptoms of yellowing, necrosis, and leaf abscission indicated by red arrows. (C) The resistant S_1.(D) resistant *G. nelsonii* Fryx. (E) resistant *G. herbaceum* L.

Figure 7. Cotton bolls from cotton TM-1 and from the F_1 hybrid of TM-1 × S_1 tetraploid. (A) The normal conical boll of TM-1 shown by the red arrow. (B) The abnormally conical boll of TM-1 × S_1 F_1 plants on the right and on the left(C), indicated by yellow arrows. (D) The deformed hybrid boll of F_1 plants indicated by yellow arrows.

Figure 8. Cotton TM-1, the S_1 tetraploid and F_1-1, F_1-2 plants. (A) Cotton TM-1 was tower-like with longer and looser branches. (B) S_1 was tower-like with shorter and more compact branches. (C) Hybrid F_1-1 was tower-like with shorter and looser branches and pink flowers (red arrow). (D) Hybrid F_1-2 was tower-like with longer and looser branches and had white flowers (white arrow).

Figure 9. Pictures of leaves, flowers, and bolls of cotton TM-1, the S_1 tetraploid, and of F_1-1 and F_1-2 plants. (A) Cotton TM-1 bore bell-shaped flowers with milky

corollas, milky filaments, milky anthers and pollen, without basal patches, wavy calyxes, heart-shaped bracts grown together, and were broad-leaved. (B) S_1 had bell-shaped flowers with pink corollas, milky filaments, yellow anthers and pollen, red basal patches, serrated calyxes, sword-shaped bracts grown separately rather than together, and had heart-shaped or broad leaves. (C) Hybrid F_1-1 had bell-shaped flowers with pink corollas, milky filaments, yellow anthers and pollen, red basal patches, serrated calyxes, heart-shaped bracts grown separately rather than together, and broad leaves. (D) Hybrid F_1-2 bore bell-shaped flowers which were milky white in the middle with a light red corolla border, milky filaments, milky anthers and pollen, without basal patches, wavy calyxes, heart-shaped bracts grown together, and broad leaves. (E) A normal conical boll of cotton TM-1. (F) A green pear-shaped boll of S_1 with a sunken navel. (G) A downy green boll of F_1-1 that was deformed, indicated by the red arrow. (H) A normal conical boll of F_1-2.

Table 1. Comparison of shape and colours of plant parts in F_1 and S_1

Shape/ colours	*G. herbaceum* L. (♀)	*G. nelsonii* Fryx. (♂)	Diploid F_1	Tetraploid S_1
Plant	Tower	Cylindrical	Tower	Tower
Leaf	Broad-leaved	Ovoid	Broad-leaved, ovoid, subcircular	Broad-leaved, ovoid, subcircular
Flower	Trumpet	Funnel-shaped	Trumpet	Trumpet, Funnel-shaped, cylindrical, closed, half closed
Calyx	Cup-shaped	Wavy	Serration	Serrated, Wavy
Bract	Heart-shaped	Lanceolate	Sword-shaped	Triangle Dentate, ensiform
Boll	Conical	Oval	/	Pear-shaped, sunken navel, oval, irregular round boll
Fibre	Spiral	Upright	/	Wavy
Stem	Green	Brown	Green	Green
Leaf	Green	Grey green	Green	Grey green
Corolla	Yellow	Light pink	Pink	Pink, Light pink, Milky white, Red and white
Petal base spot	Pink	Purple	Red	Red, Purple, Rose, None
Filaments	White	Pink	Light pink	Light pink
Anther	Yellow	Red	Light yellow	Yellow
Pollen	Yellow	Pink	Yellow	Yellow
Boll	Red and green	Green	/	Green
Fibre	Grey-white	Dark brown	/	Light green, Brown

Table 2. Comparison of quantifiable parameters in F_1 and S_1

Quantifiable parameters	*G. herbaceum* L. (♀)	*G. nelsonii* Fryx. (♂)	Diploid F_1	Tetraploid S_1
Plant height (cm)	80	200	300	150
Main stem diameter (cm)	1.5	2	3.0	2.5
Leaf length (cm)	4~6	5~9	4~6	4~8
Leaf width (cm)	4~6	4~6	4~6	4~7
Leaf lobation	3~5	0~1	0~3	0~3
Bract tooth	5~9	1	4~6	4~6
Locule	3	3	/	3~4
Seeds per locule	1-6	1-5	/	1~5
Bracteole	Joint	No joint	No joint	No joint, Jointed
Stem fuzz	Less	Medium	More	More
Leaf fuzz	No	Medium	More	More
Leaf Lobes	Parted	No	Lobate	Lobate
Column length	Medium	Long	Medium	Medium, Long, short
The size of Petal base spot	Small	Medium	Medium	Medium, Small, None
Boll tip protuberance degree	Medium	Weak	/	None, Weak
Branch length	Short	Short	Long	Long
Branch openness	Compact	Compact	Looser	Looser

Table 3. Comparison ofshape and colours of plant parts in F_1-1 and F_1-2

Shape/colours	TM-1 (♀)	A selected S_1 (♂)	Hybrid F_1-1	Hybrid F_1-2
Plant	Tower	Tower	Tower	Tower
Leaf	Broad-leaved	Broad-leaved, Ovoid, Heart-shaped	Broad-leaved	Broad-leaved
Flower	Trumpet	Trumpet	Trumpet	Trumpet
Calyx	Cup-shaped	Serration	Wavy	Cup-shaped
Bract	Heart-shaped	Sword-shaped	Heart-shaped	Heart-shaped
Boll	Conical	Pear-shaped	Deformed Conical	Deformed conical
Branch length	Longer	Shorter	Shorter	Longer
Branch openness	Looser	Compact	Looser	Looser
Bracteole	Joint	No joint	No joint	Jointed
Stem	Red	Red	Green	Green
Leaf	Green	Green	Green	Green
Corolla	Milky	Pink	Pink	White and red
Petal base spot	None	Red	Red	None
Filaments	Milky	Milky	Milky	Milky
Anther	Milky	Yellow	Yellow	Milky
Pollen	Milky	Yellow	Yellow	Milky

Table 4. SSR polymorphism primer sequences

Primer number	Forward sequence (5'-3')	Reverse sequence (5'-3')
NAU1157	GAGTTTGGTTCTGGGTTGAG	GATCCTTTTCATCTCCTCCA
NAU1355	ATCTGTTTACGCCACTCTCC	CCAGCCTTTGACATTTTTCT
NAU1052	CGCAGATAAAGGATGGATTT	AGAGCTGGAGGACATAACAAA
JESPR156	GCCTTCAATCAATTCATACG	GAAGGAGAAAGCAACGAATTAG
BNL1679	AATTGAGTGATACTAGCATTTC AGC	AAAGGGATTTGCTGGCAGTA
NAU3093	GTCTTGAACCGGAACTTGAT	TCCTGTTGAACACCAAAGTG

The primer sequences were obtained from http://www.cottonmarker.org/cgi-bin/cmd_search_marker.cgi

Figures:

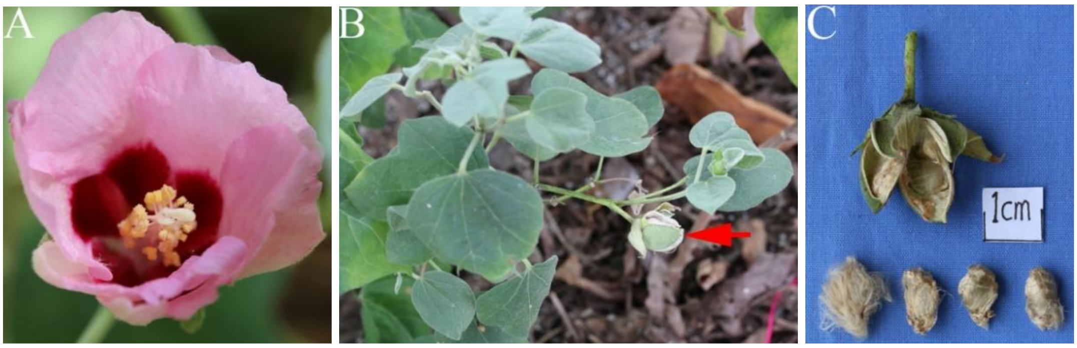

Figure 1

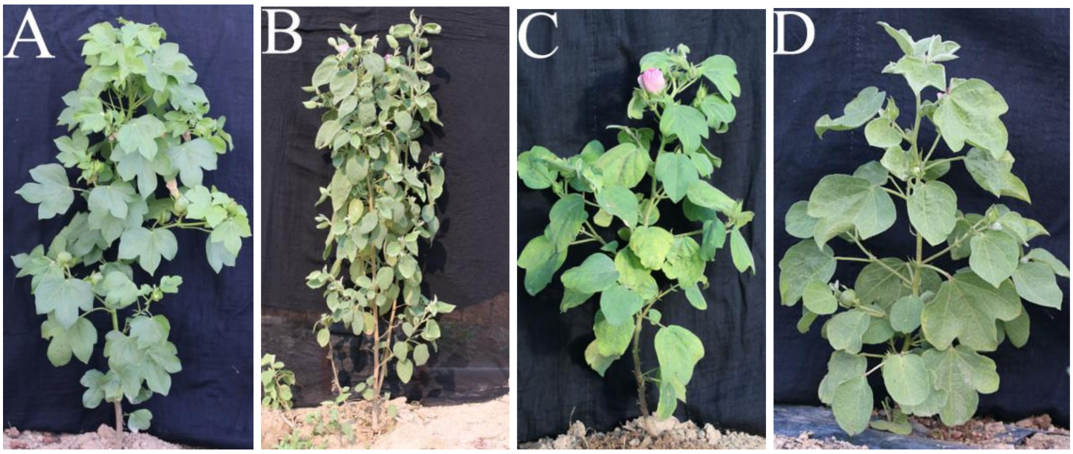

Figure 2

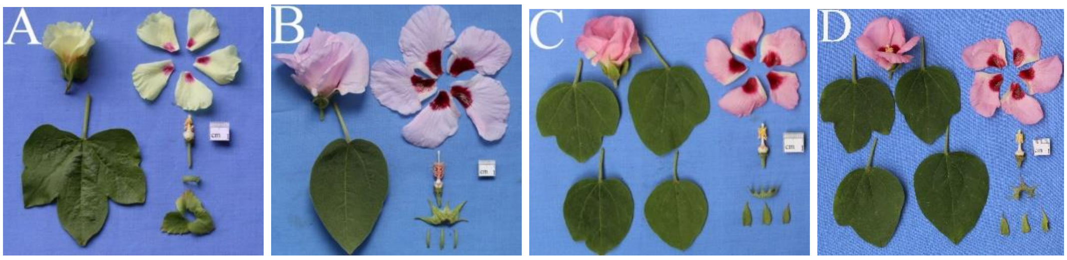

Figure 3

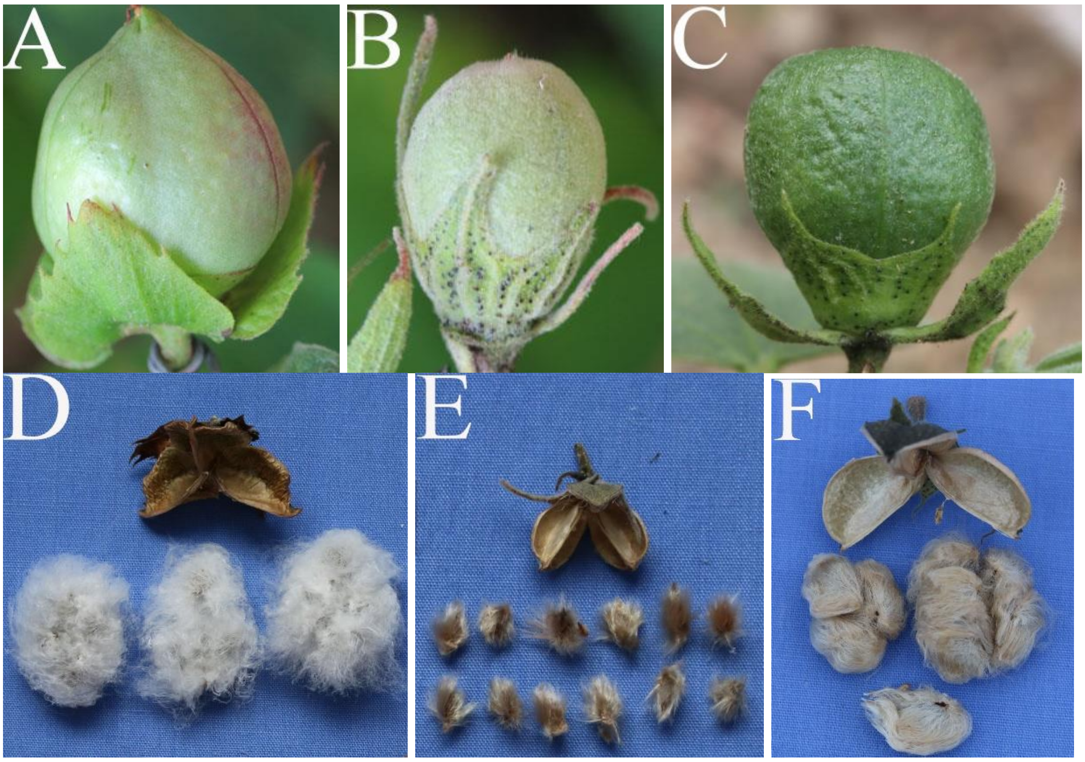

Figure 4

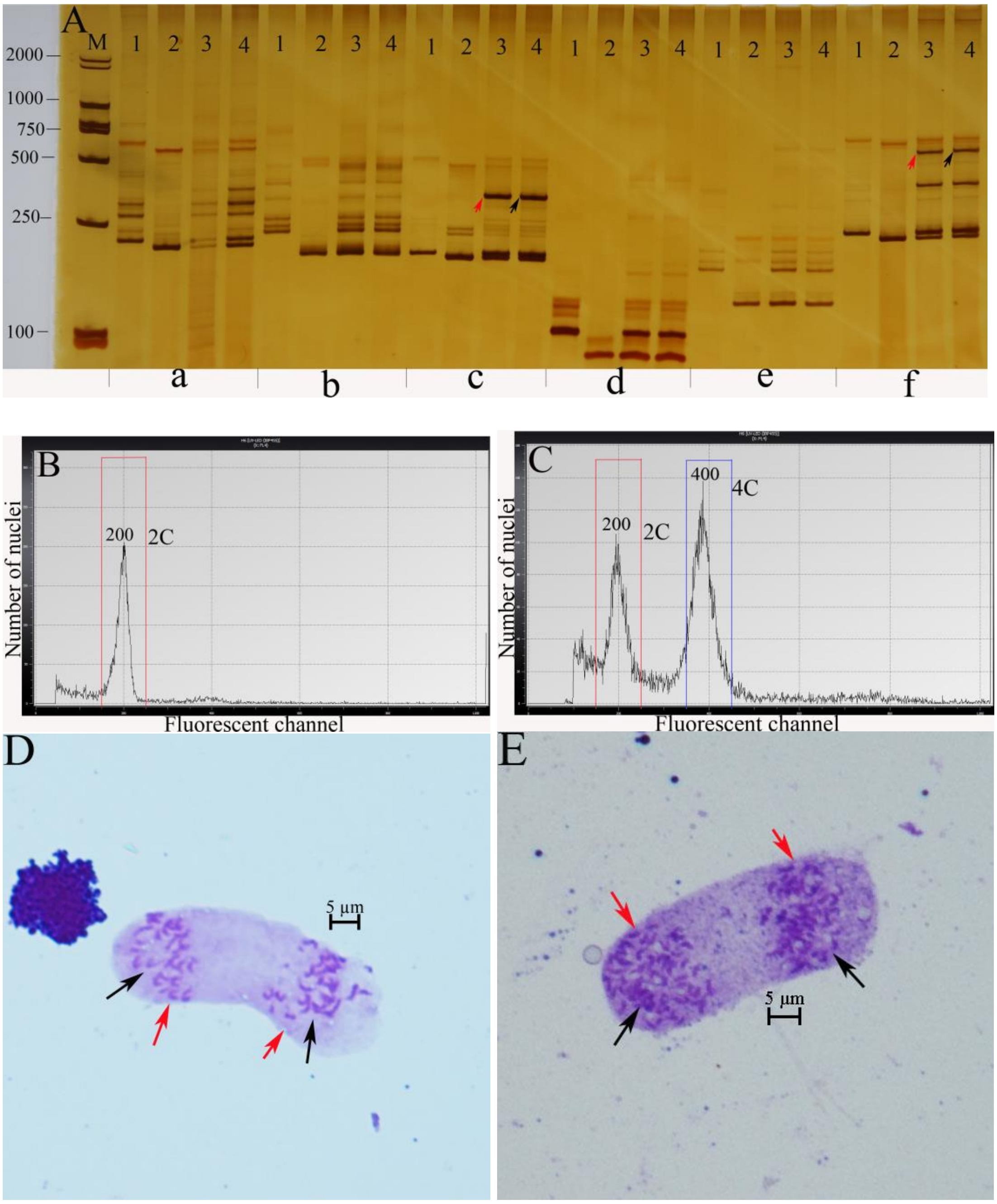
A
M
2000
1000
750
500
250
100
1 2 3 4
a
b
c
d
e
f
B
200
2C
Number of nuclei
Fluorescent channel
C
200
2C
400
4C
Number of nuclei
Fluorescent channel
D
5 µm
E
5 µm

Figure 5

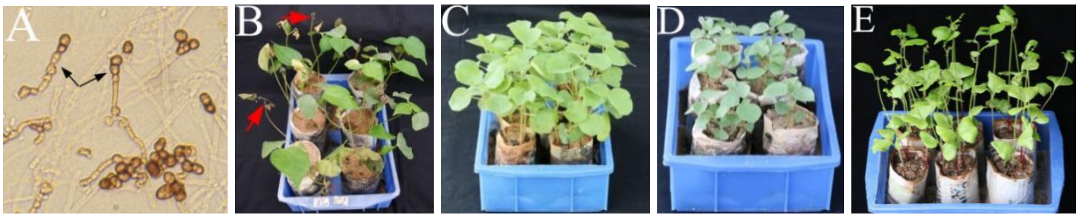

Figure 6

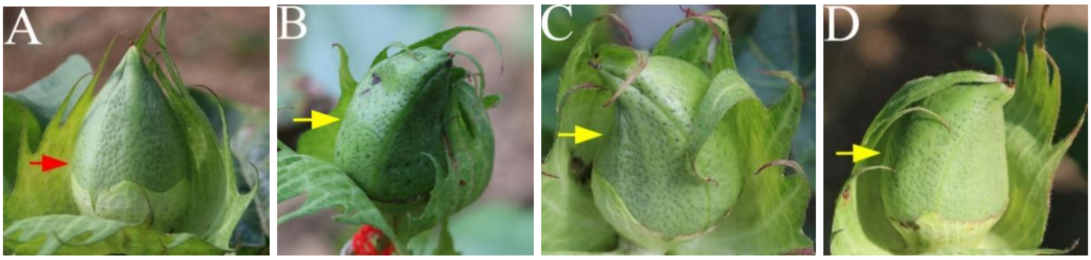

Figure 7

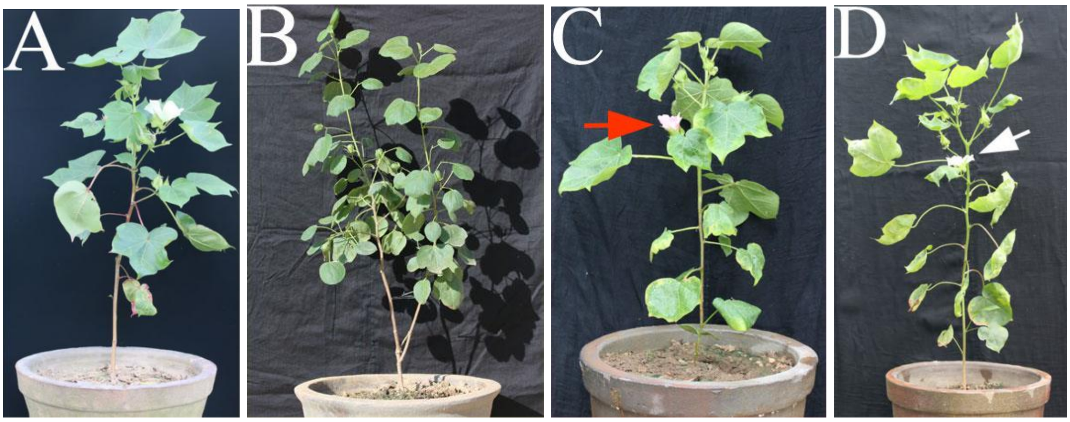

Figure 8

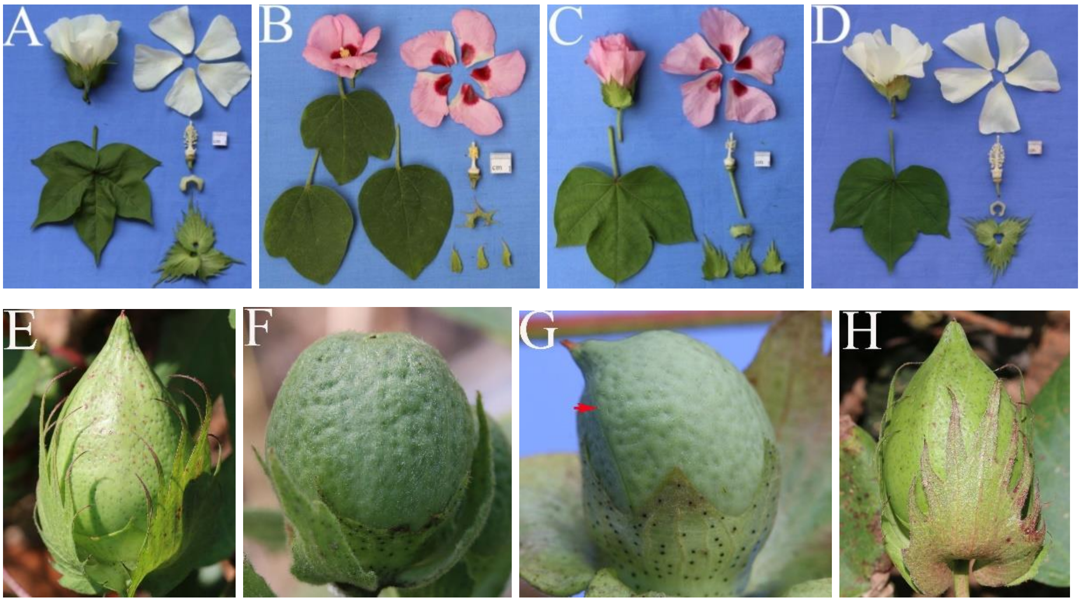

Figure 9

Morphological and genetic description of a novel synthetic allotetraploid of *Gossypium herbaceum* L. and *Gossypium nelsonii* Fryx

Di Chen, Bingqiang Xu, Shun Song, Yingdui He, Lixia Wang, Yu Ge, Rulin Zhan*, and Xiaomin Yin*

Supplementary Information

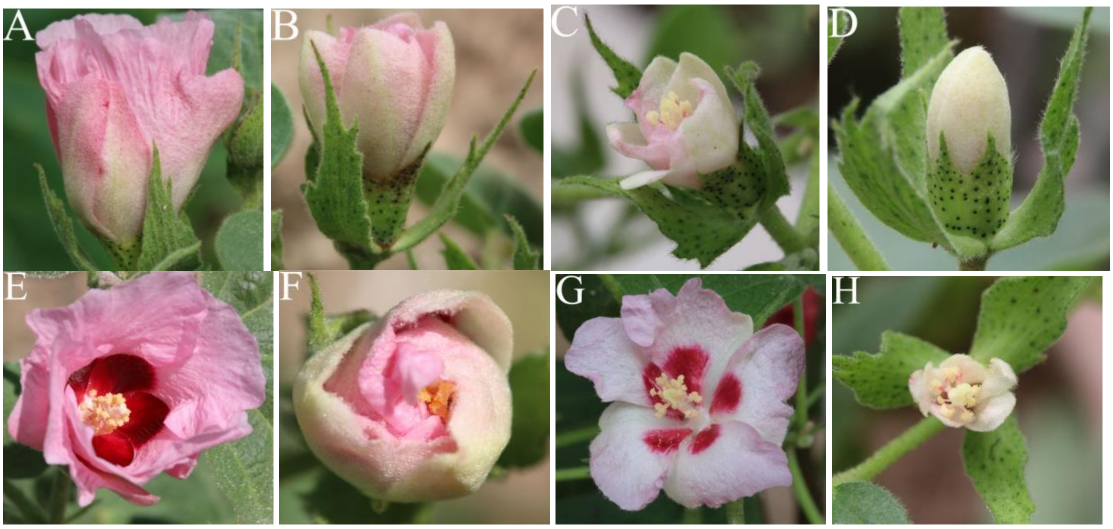

Supplementary Figure 1. Flowers of the tetraploid, S_1. (A) A pink cylindrical flower with pink corolla. (B) A funnel-shaped flower with light-pink corolla. (C) A funnel-shaped flower with white and pink alternating corolla. (D) A white closed flower. (E) A bell-shaped flower with red corolla and a clear rose-coloured spot on the petal base. (F) A semi-closed flower with pink corolla. (G) A bell-shaped flower with pale pink corolla. (H) A small flower with white corolla and no spot on the petal base.

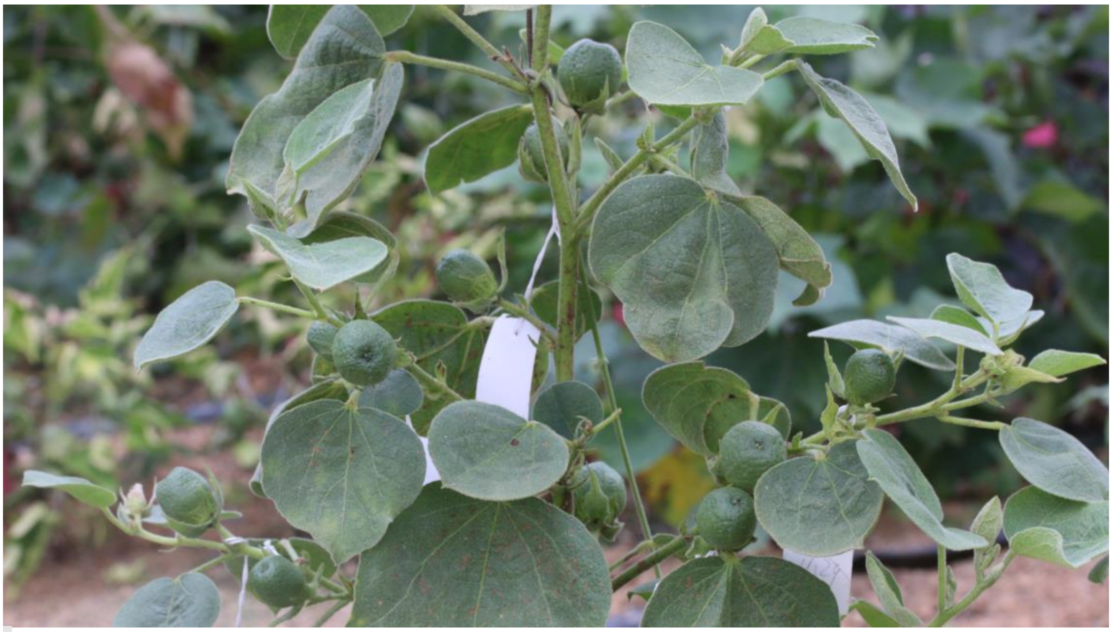

Supplementary Figure 2. Fruits of the tetraploid, S_1. Multiple bolls of S_1 on multiple fruit branches with the sunken navel of the boll tip showing an ergonomic shape with strong bolls.

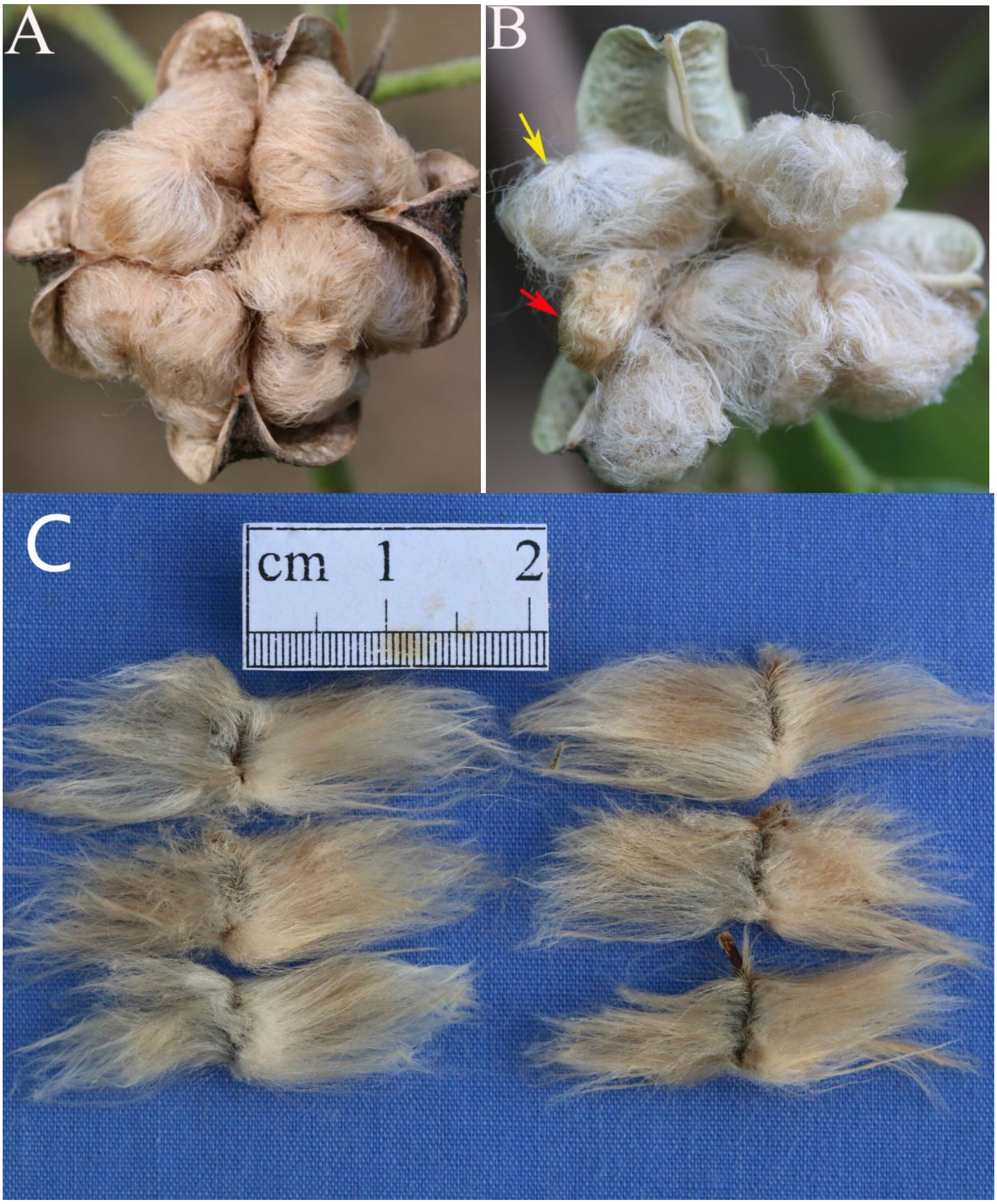

Supplementary Figure 3. Fibre colours of the tetraploid, S_1. (A) A four-chambered boll with brown fibres. (B) A three-chambered boll with light green and brown cotton fibres. (C) The brown cotton fibres of S_1 with an average fibre length of 13 mm.

Click here to access/download;Figure;Figure 2.docx

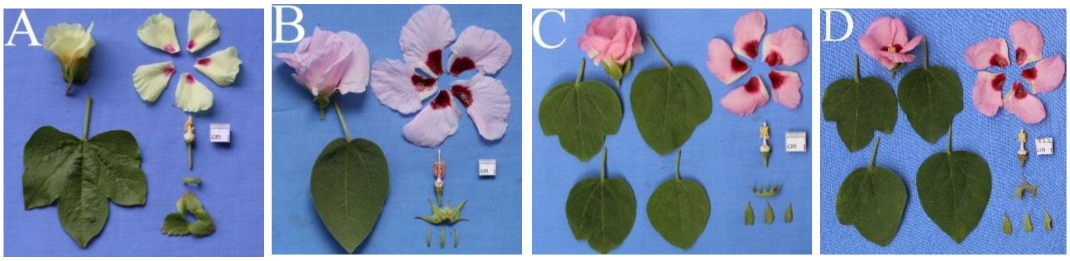

Figure 2

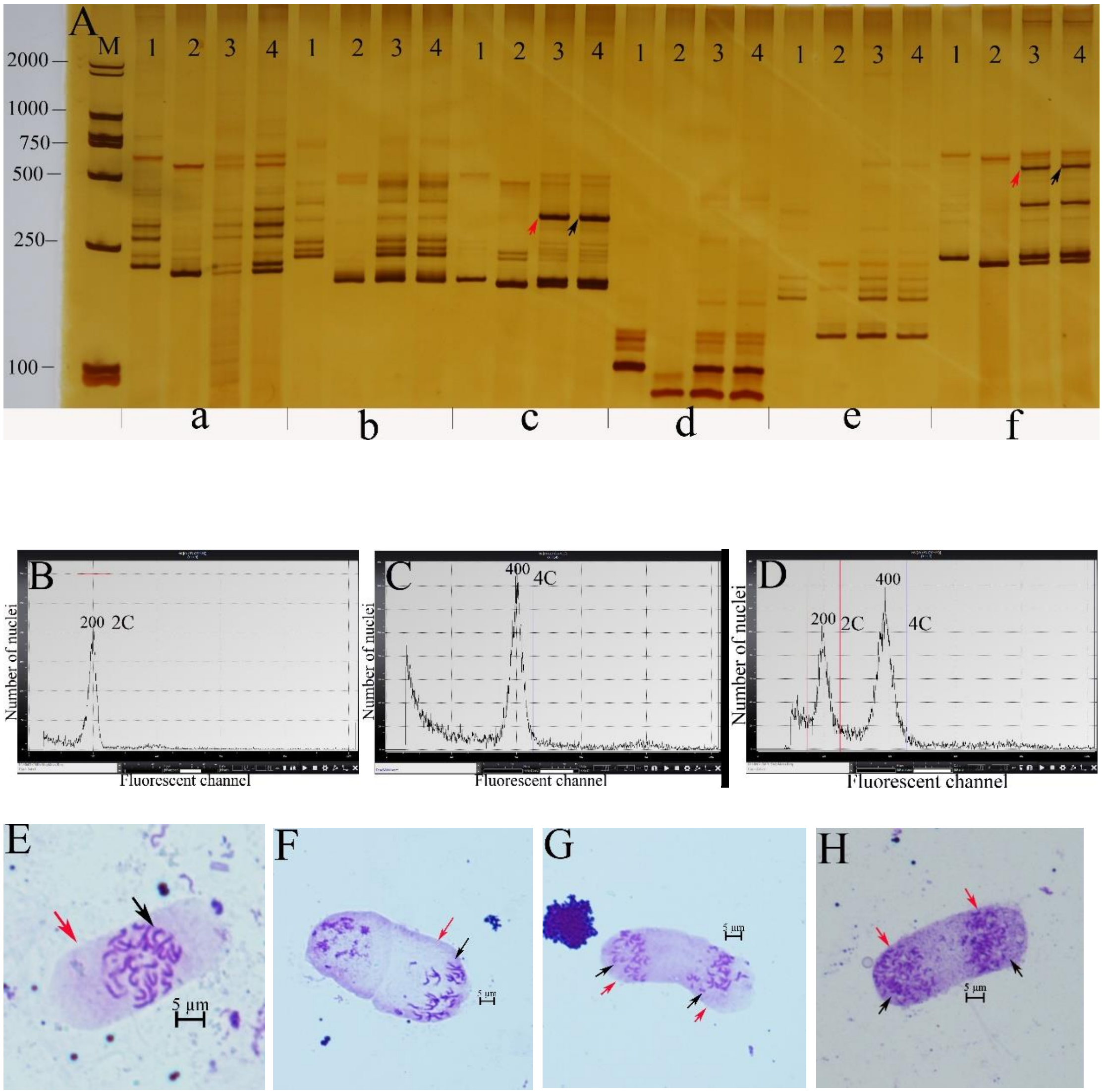
A
M
2000
1000
750
500
250
100
1 2 3 4
a
b
c
d
e
f
B
C
D
200 2C
400 4C
200 2C
400
4C
Number of nuclei
Fluorescent channel
E
F
G
H
5 μm

Figure 3

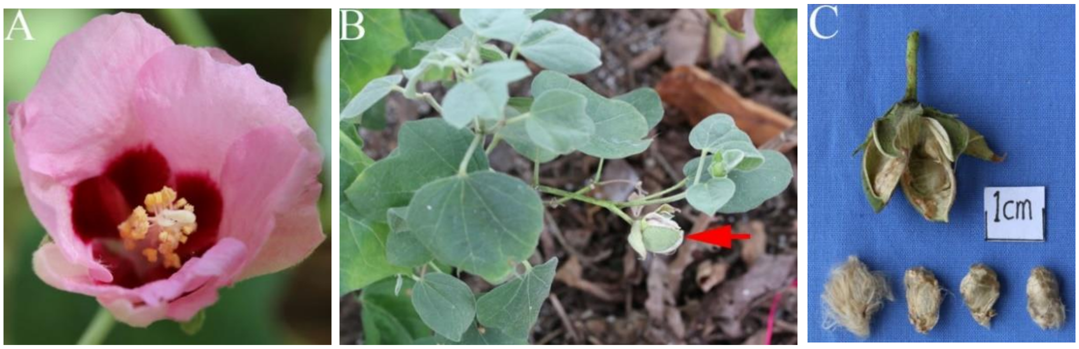

Figure 4

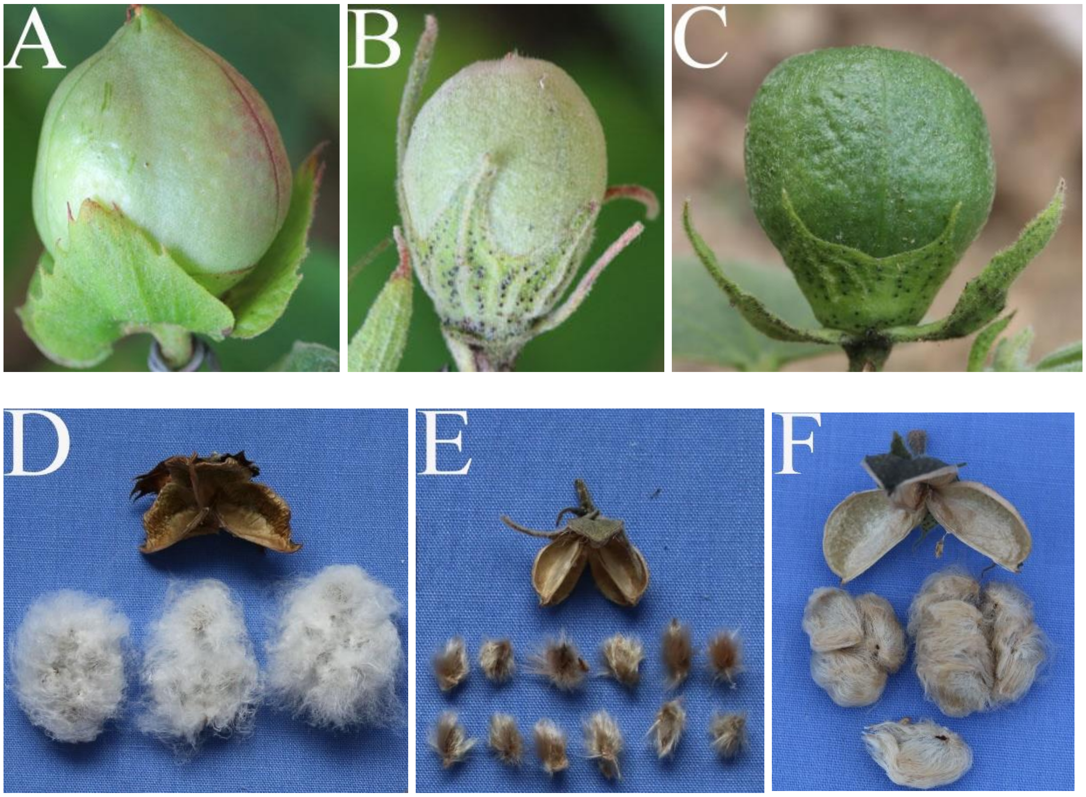

Figure 5

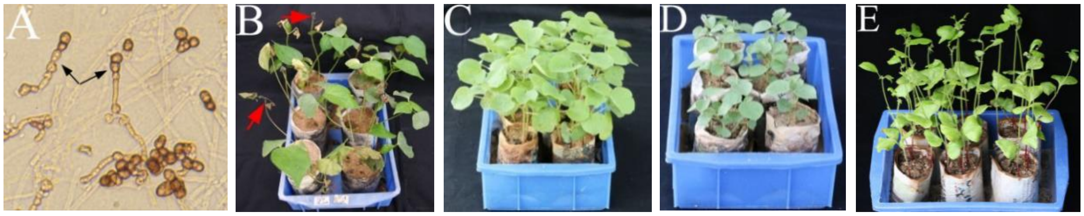

Figure 6

Click here to access/download;Figure;Figure 7.docx

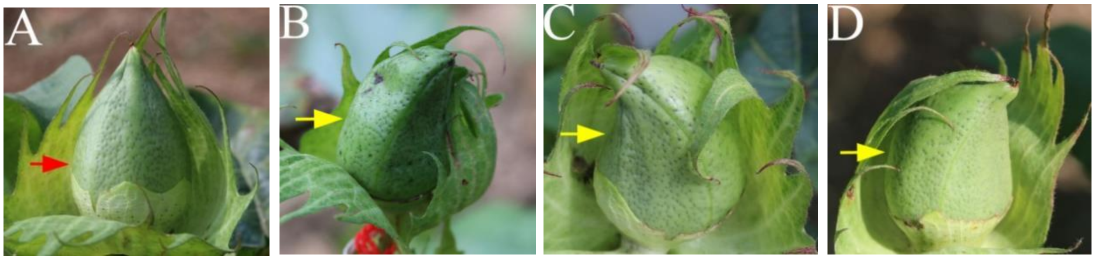

Figure 7

 Click here to access/download;Figure;Figure 8.docx

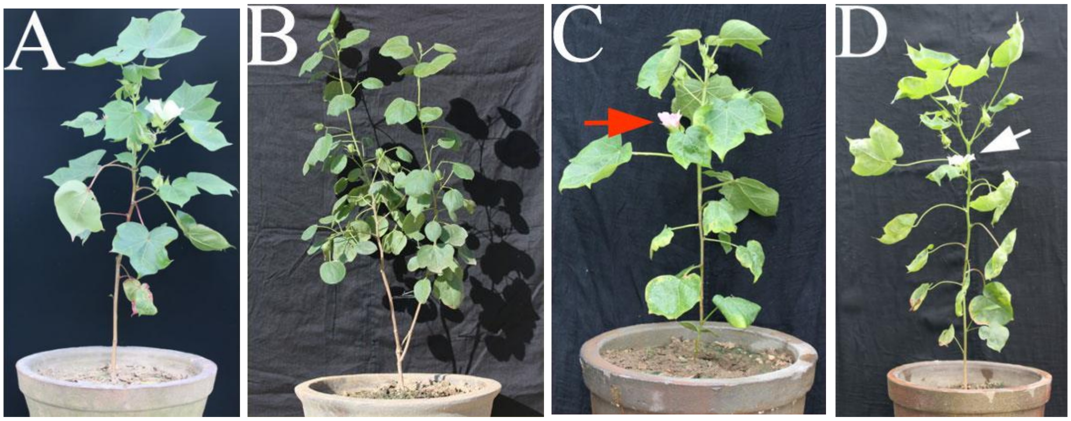

Figure 8

Figure 9

Click here to access/download;Figure;Figure 9.docx

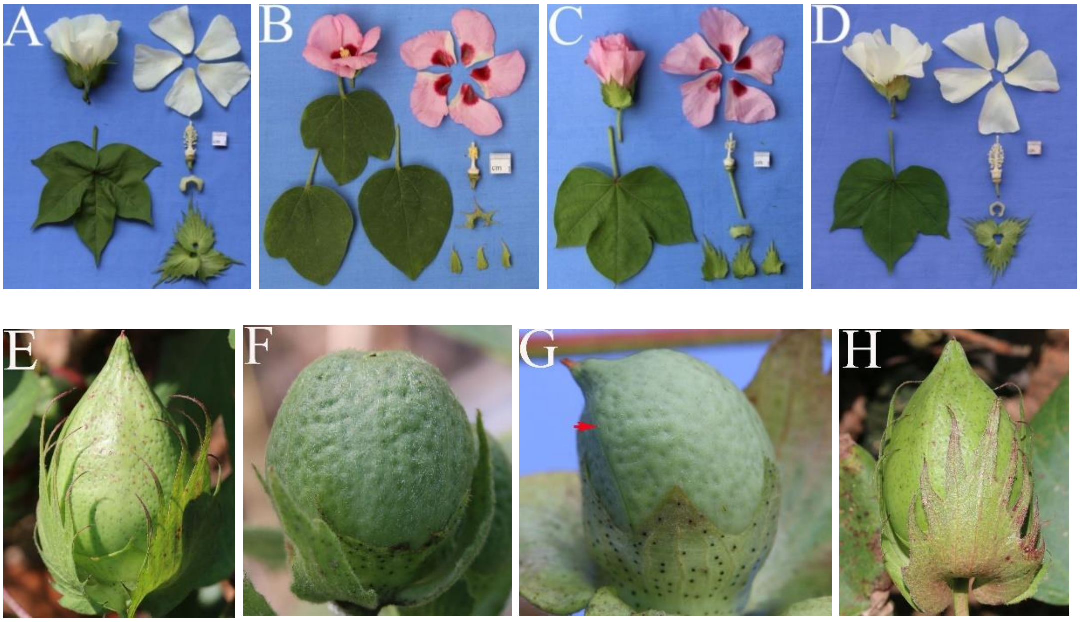

Figure 9

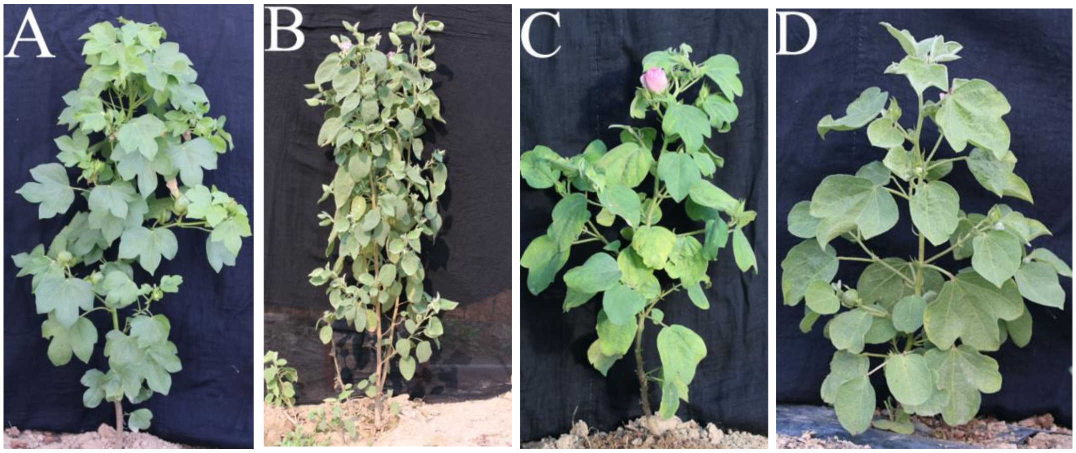

Figure 1

Click here to access/download;Figure;S1 Fig.docx

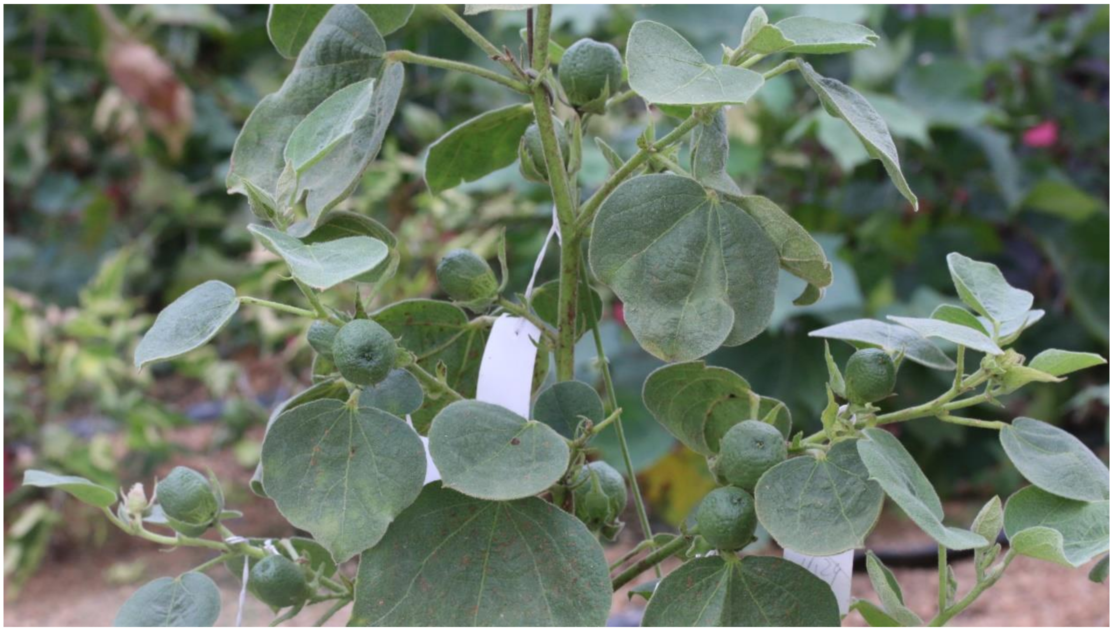

S1 Fig.

Click here to access/download;Figure;S3 Fig.docx

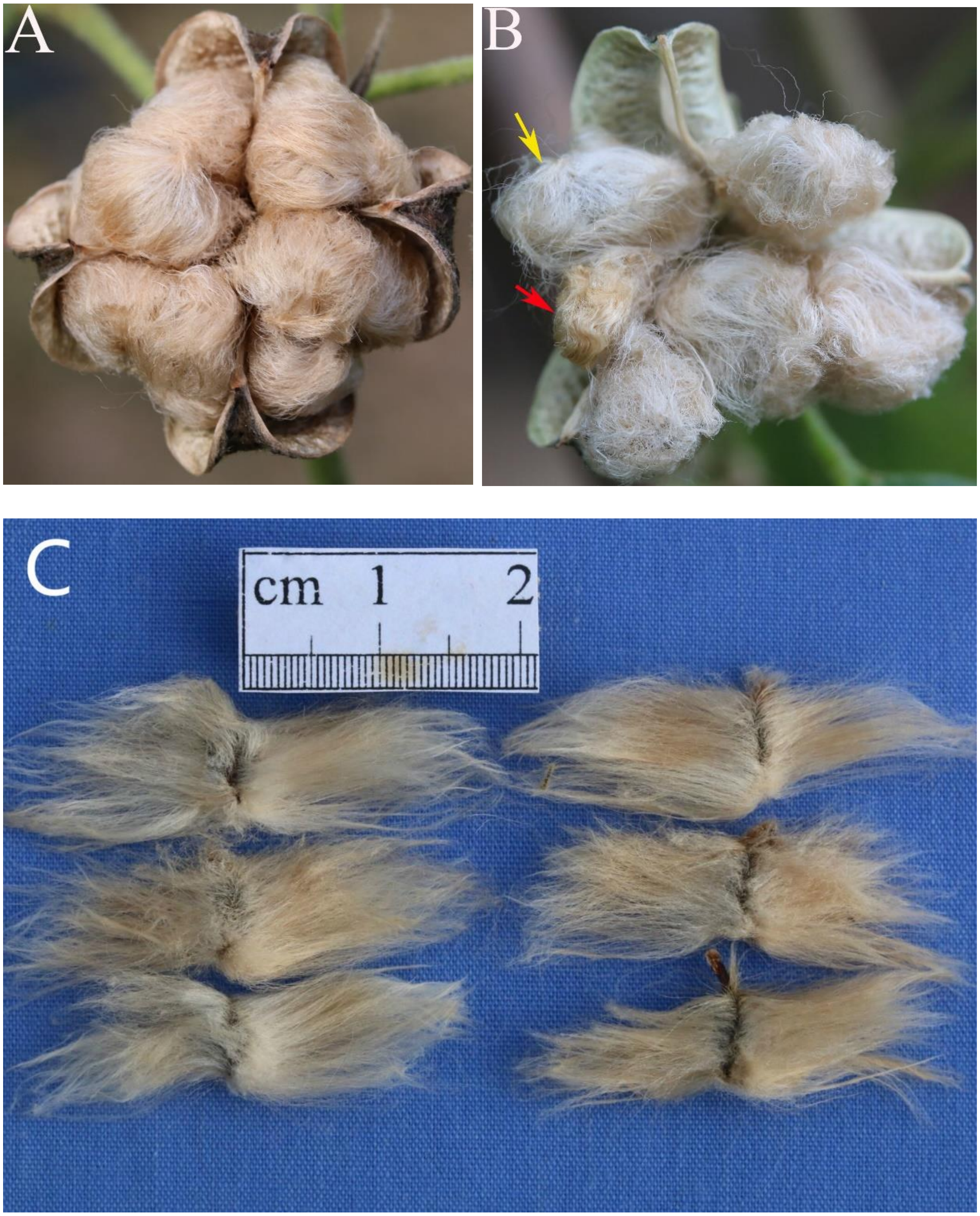

S3 Fig

 Click here to access/download;Figure;S2 Fig.docx

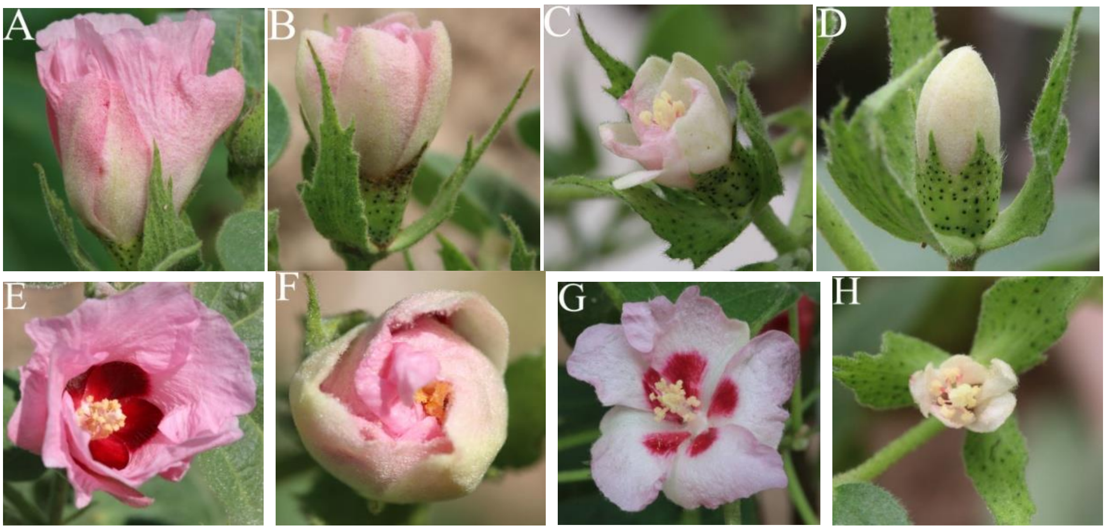

S2 Fig.

The review certificate by Editage

Click here to access/download
Supporting Information
renamed_07ace.pdf

Morphological and genetic description of a novel synthetic allotetraploid of *Gossypium herbaceum* L. and *Gossypium nelsonii* Fryx.

Xiaomin Yin[#], Rulin Zhan[#], Shun Song, Yingdui He, Lixia Wang, Yu Ge, Di Chen[*]

Haikou Experimental Station, Chinese Academy of Tropical Agricultural Sciences (CATAS), Haikou, China

*Corresponding author

E-mail: chendi209@163.com (DC)

Keywords: genetic evolution, *Gossypium*, interspecific hybridization, synthetic allotetraploid

Abstract

Gossypium herbaceum L. is a cultivated, diploid cotton species (2n = 2x = 26, A_1A_1) with superior resistance to drought, sap-feeding insects, and cotton leaf curl virus. *Gossypium nelsonii* Fryx. is a wild, diploid cotton species (2n = 2x = 26, G_3G_3) that produces strong fibers and is resistant to drought, aphids, and Verticillium wilt and can delay pigment gland formation. These cotton species were used to synthesize an allotetraploid cotton species for use as a foundation for the future transfer of beneficial genes to cultivated cotton. Here, we used *G. herbaceum* (♀) and *G. nelsonii* (♂) for interspecific hybridizations to obtain F_1 generation. The apical growth points were immersed in colchicine to induce chromosome doubling. Tetraploid S_1 cotton bolls were then obtained from the diploid F_1 plants to successfully synthesize tetraploid cotton plants. Morphological markers, detecting DNA polymorphism,following ploidy analysis and microscopic observation were used to identify the tetraploid S_1 plants, and then self-pollination was conducted. The S_1 plants showed good growth without significant segregation, indicating that the compatibility among them had been maintained. The S_2 plants showed obvious segregation, indicating some incompatibility. The S_2 seedlings that were cultivated in nutrient pots were inoculated with Verticillium wilt, but they did not develop the disease and had an immune phenotype. The S_1 interspecific hybrid S_1 plants were hybridized with cultivated *G. hirsutum* L. to measure the compatibility of the S_1 plants and *G. hirsutum* L. and to determine if they could be used for cotton species improvements. The results showed that these plants had some compatibility and that fertile hybrid progeny could be obtained. The newly synthesized allotetraploid S_1 line will have important application value in polyploidy evolution studies and could act as

a bridge to transfer beneficial genes from two diploid parental cotton species to *G. hirsutum* L.

Introduction

The *Gossypium* genus includes 54 species, of which 4 are cultivated cotton species (2 diploid and 2 tetraploid) and 50 are wild cotton species [1,2]. Approximately 90 % commercially produced cotton is cultivated from *G. hirsutum* L. Studies have shown that the existing cultivated varieties have undergone long-term, artificial selection and that their yield and fiber qualities, in particular, have been largely developed, making it difficult to compare them with wild species. However, this long-term, intensive cultivation and artificial selection has resulted in the reduced development of certain resistance traits that are lost during unreasonable selection. Consequently, as the high-yielding quality of cultivated species continuously increases, abiotic and biotic resistance usually does not increase and can even be weakened [3,4]. To increase the resistance in *G. hirsutum* L., the existing genetic basis of cotton should be expanded to adapt to biotic and abiotic stresses. It is generally believed that wild cotton species contain a large treasure trove of traits that could be used to improve cultivated species [5]. There are more than 50 wild cotton species, which are widely distributed in unique ecological conditions in the Americas, Oceania, Africa, and Asia. These species have undergone long-term, natural selection, developed many adaptations, and are resistant to many types of adverse factors, such as salinity, heat, drought, insects, and disease. At the same time, there are many mutant varieties, all of which contribute to cottons rich genetic foundation [6,7].

G. nelsonii Fryx. is a wild diploid cotton species that originates from Australia and has many economically valuable characteristics, such as delayed pigment gland

formation. This is beneficial for producing seeds with low gossypol levels. In addition, it is a potential source of human foodstuff and animal feed; is resistant to blight, Verticillium wilt, aphids, and mites; and is tolerant of drought, high temperatures, and abiotic stresses. Furthermore, its brown, straight, extensible, and high-strength fibers are a valuable agronomic trait. *G. herbaceum* L. is a cultivated diploid cotton species that is produced in western China and neighboring Russia country . This species is highly resistant to drought, sap-feeding insects (such as leafhoppers, whiteflies, thrips, and aphids), and cotton leaf curl virus [8,9]. If these characteristics can be transferred to tetraploid *G. hirsutum* L. (2n = 4x = 52, AADD), the resulting species will be extremely valuable.

We summarize a pathway for transferring genes from two cotton species into *G. hirsutum* L.. Specifically, two different diploid species undergo artificial interspecific hybridization so that they can rapidly and mutually recognize and overcome their biological segregations to form the diploid hybrid F_1. Following that, artificial ploidy doubling of F_1 hybrids is carried out to obtain a cotton species that has the same chromosome number as *G. hirsutum* L., i.e. it is a new tetraploid cotton species. The new tetraploid cotton is then hybridized with the tetraploid *G. hirsutum* L. to achieve efficient gene transfer. This process shortens the time needed to introduce essential genes from two species and is currently the most direct and easiest method that could potentially be utilized with wild cotton. In the last few decades, we have successfully produced many interspecific hybrids, including *G. arboretum* × *G. raimondii* UI [10], *G. hirsutum* × *G. sturtianum* [11], *G. arboreum* × *G. bickii* [12], *G. hirsutum* × *G. klotzschianum* [13], *G. hirsutum* × *G. trilobum* [14], *G. arboreum* × *G. anomalum* [15], *G. hirsutum* × *G. anomalum* [16], and *G. herbaceum* × *G. austral* [17]. However,

to the best of our knowledge, there has been no report of using *G. herbaceum* and *G. nelsonii* to successfully synthesize an allotetraploid ($A_1A_1G_3G_3$).

Owing to the biological isolation between the species, it is difficult to directly hybridize diploid *G. herbaceum* and *G. nelsonii* with tetraploid *G. hirsutum*. Therefore, we ~~employed~~ homoploid hybridization to provide a pathway to simultaneously transfer i~~deal~~ genes from these two diploid cotton species into *G. hirsutum* using *G. herbaceum* as the female parent and *G. nelsonii* as the male parent to generate the interspecific hybrid F_1 (2n = A_1G_3 = 26). Colchicine treatments were used to double the chromosome number, and form a new tetraploid cotton (code: S_1) (2n = 4x = 52, $A_1A_1G_3G_3$). The birth of the new S_1 cotton tetraploid not only enriches the ~~genetic foundation of cotton~~ but also provides a valuable intermediate material for cotton hybridization and breeding. This is a successful example of using wild cotton species for distant hybridization and has important significance for promoting scientific research on cotton ~~plants.~~

Materials and methods

Plant material~~s~~

The diploid *G. herbaceum* cotton species that has been cultivated in China, is also known as Hongxingcaomian (2n = 2x = 26, A_1A_1), and is a line that has not undergone any generations of inbreeding. *G. nelsonii* is a diploid cotton species from Australia (2n = 2x = 26, G_3G_3) and the two *G. hirsutum* L. lines ~~were~~ cotton cv. *Jimian11* and TM-1. These lines mentioned above were obtained from The China National Wild Cotton Nursery in Sanya, China. The *Verticillium dahliae* strain used

for disease resistance identification was the Dahlia Vd080 strain and it was provided by the Institute of Cotton Research, Chinese Academy of Agricultural Sciences.

Interspecific hybridization

Cotton interspecific hybridization was carried out in Sanya (18°25′34″N, 109°28′25″E), Hainan, China in 2015 [18]. When the two parental species flowered at the same time, ~~healthy~~ *G. herbaceum* were selected as the female parents. One day before flowering, all stamens in the flowers from the female parents were completely removed. Wax tubes were used to cover the stigmas and non-woven bags were used to cover all the floral buds, entirely, to prevent contamination by external pollen. The afternoon before flowering, ropes were used to tie the apex of the petals in the flowers from the male parent (*G. nelsonii*), and non-woven bags were used to cover the floral buds entirely, to prevent contamination by external pollen. From 9 am to 12 pm on the day of flowering, pollen from the male parent was used to pollinate the stigmata of the female parents for hybridization. After pollination was completed, wax tubes were used to cover the stigmas, non-woven bags were used to cover the entire floral buds, and labels were hung. At 5 pm on the following day, when the hybridized flowers had withered, the non-woven bags were removed to promote normal growth in the hybridized bolls. After the cotton bolls had matured and underwent dehiscence, the seeds were removed and sown in nutrient pots containing sterile soil. After 1-2 true leaves had sprouted, the plants were transplanted into fields and normal fertilizer and water management was carried out. The morphological characteristics of the plants were observed, including the type of plant, stems, leaves, flowers, bolls,etc.~~We further~~ speculated as to whether ~~the plant was a true interspecific hybrid F_1.~~ A Canon digital camera (EOS 70D) equipped with an F1.8 lens with a macro of RF 35 mm was

used to photograph the morphological characteristics at the various sites. The Photoshop CS5 software (Adobe Inc) was used to edit all acquired images.

No permits were required for the described study, which complied with all relevant regulations.

SSR molecular marker validation of the F_1 hybrids

We randomly selected 568 pairs of SSRs based on our cotton genetic map [19] to screen for parental polymorphisms. The polymorphic primers obtained were used to validate the authenticity of the interspecific F_1 hybrids. These SSR primer sequences can be found at http://www.cottonmarker.org. A programmable gradient thermal cycler (Bio-Rad T100) was used for SSR-PCR amplification[20]. Electrophoresis and silver staining of the PCR products were carried out according to methods previously reported [21]. A Canon digital camera (EOS 70D) equipped with a F1.8 lens with a macro of RF 35 mm was used to photograph the electrophoresis bands. The Photoshop CS5 software (Adobe Inc) was used to edit all images.

F_1 hybrid chromosome doubling

In 2016-2018, the F_1 hybrids that were validated by the SSR molecular markers were grafted for storage and breeding. At 25 ℃, the apical growth points of these F_1 hybrids were immersed in 0.30 % (w/v) colchicine for 6 hours. After immersion, they were placed in pots in the greenhouse that were not exposed to light and incubated until new growth points or floral buds sprouted. When the length of the new growth points reached 5 cm, they were removed and grafted for storage and breeding and further validation of the chromosome doubling results was carried out [22]. Newly sprouted floral buds could grow until the afternoon before flowering. Then a rope was

used to tie the tip of the petal for self-pollination and non-woven bags were used to cover the entire floral bud to prevent outcrossing. After the cotton bolls matured and underwent dehiscence, the seeds were removed and sown in nutrient pots. After 1-2 true leaves sprouted, the plants were transplanted into fields. The morphological characteristics of the plants were observed, including the type of plant, stems, leaves, flowers, bolls, and other major morphological traits and further validation of the chromosome doubling results was carried out. A Canon digital camera (EOS 70D) equipped with a F1.8 lens with a macro of RF 35 mm was used to photograph the morphological characteristics in various sites. The Photoshop CS5 software (Adobe Inc) was used to edit all images.

Ploidy analysis

Diploid *G. herbaceum* L. and the TM-1 tetraploid *G. hirsutum* L. lines were used as controls and flow cytometry was used to examine the differences in the chromosome numbers between the F_1 hybrid and its resultant tetraploid S_1 progeny. Preparation of the sample cell suspensions, specific staining, and loading were carried out according to the description of Galbraith et al. [23]. The Sysmex-Partec CyFlow Cube8 flow cytometer was used to determine the ploidy in the nuclear DNA content. The CellQuest Pro software was used to analyze the peak values in the channel.

~~Root tip chromosome observations~~

G. herbaceum and *G. nelsonii* were used as controls and microscopic observations of the number of root tip chromosomes in the F_1 hybrid and its resultant tetraploid S_1 progeny were carried out, to observe the differences in the chromosome

number between the two. Finally, the F_1 hybrid chromosome doubling result was determined. Root tip fixation, preservation, dissection, squash preparation, and microscopy were carried out according to the methods of Wu et al. [24]. The biological microscope (MODEl ECLIPSE Ni-E microscope and photography system, Nikon) was used to observe the root tip chromosome number and acquire photographs. The Photoshop CS5 software (Adobe Inc) was used to edit all images.

~~Determination of~~ disease resistance in the tetraploid S_2

The *Verticillium dahliae* strain (code: Dahlia Vd080 strain) was inoculated indoors onto tetraploid S_2 seedlings growing in nutrient pots for preliminary determination of disease resistance. cv. *Jimian11* was used as an infection control group. *G. herbaceum* and *G. nelsonii* were used as disease resistance controls and the S_2 were the determination targets. In the experiment, there were 2 treatments (fungal suspension and sterile water for inoculation) and triplicates. Every replicate contained 540 plants.

Inoculations of the seedlings in the nutrient pots were carried out according to a previously reported method [25]. Seeds were sown in cylindrical paper pots (height 8 cm and diameter 5 cm) with no bottom and filled with sterile medium. When the first true leaf had grown until it was level, 10^7 conidia/ml of suspension was directly injected into the plastic tray with a diameter of 8 cm. ~~In contrast, in the control tray, sterile water was injected.~~ The paper pot and seedlings were placed upright in the trays so that the root systems adsorbed the suspension. The seedlings were incubated until disease onset. Examination of the disease symptoms was carried out according to the method previously reported by Peng et al. [26]. Changes in leaf color and degree

of wilting were used as a basis for preliminary observations of disease resistance in the experimental materials.

Tetraploid S_1 breeding application test

In the winter of 2017, hybridization was carried out in Sanya, Hainan province, China, using the tetraploid S_1 as the male parent (♂) and the TM-1 *G. hirsutum* L. line (♀) as the female parent. The hybridization method used was similar to the hybridization method previously described for *G. herbaceum* (♀) and *G. nelsonii* (♂) [18]. Investigate and record the boll-forming rate and effective boll-forming rate of hybrids.After the hybridized cotton bolls matured and underwent dehiscence, seeds were removed and sowed in nutrient pots containing sterile soil. After 1-2 true leaves sprouted, the plants were transplanted into fields. The morphological characteristics of the plants were observed, including type of plant, stems, leaves, flowers, bolls, and other major morphological traits. According to the morphological characteristics, the authenticity of the hybrids were preliminarily speculated and the fertility of the tetraploid S_1 hybrids was examined. A Canon digital camera (EOS 70D) equipped with a F1.8 lens with a macro of RF 35 mm was used to photograph the morphological characteristics at the various sites. The Photoshop CS5 software (Adobe Inc) was used to edit all images.

Results

F_1 hybrid generation and trait observations

In 2016, hybridization was conducted using *G. herbaceum* as the female parent and *G. nelsonii* as the male parent. A total of 235 flowers were pollinated and 205

formed bolls. However, most bolls did not form seeds and only 3 effective bolls were finally obtained. Each of these bolls contain 1 seed. In 2017, the 3 seeds were sown in a pot and all germinated after 5 days. After the first true leaves were level, the seedlings were transplanted to the field. Floral buds appeared after 43-45 days and flowering occurred after 68-70 days. In Sanya, China, the 3 F_1 plants showed exuberant growth, were tall, produced large numbers of flowers throughout the year, and could produce pollen. However, the pollen produced was non-viable and did not produce bolls. The samples have been preserved, but are still infertile. This highlights the difficulty in obtaining F_1 hybrids from interspecific hybridizations between *G. herbaceum* L. and *G. nelsonii* Fryx., and fertility restoration was also difficult.

According to the 14-item investigation data analysis (Table 1), the morphological traits of the F_1 plants showed classical hybrid characteristics(Figs. 1,2). Morphological comparison with parental plants showed that 5 items were intermediate between the parents including leaf shape and color, number of lobes, bract shape, calyx teeth shape, and number of bract teeth(Fig. 2A-C), 2 items were greater than the parents including plant height(Fig. 1A-C) and corolla color(Fig. 2A-C), 2 item was similar to the female parent including anther and pollen color((Fig. 2A,C)), and 5 items were similar to the male parent including stem and leaf trichomes, flowering habit, bract extension, flower opening, and extrafloral nectary(Fig. 2B,C).

Table 1. Comparison of morphological traits between *G. herbaceum* L. × *G. nelsonii* Fryx. F_1 and its parents

Character	*G. herbaceum* L.	*G. nelsonii* Fryx.	F_1
Plant height (cm)	80	200	300
Stem/branch characteristics	Less trichomes	More trichomes	More trichomes
Shape and color of leaf	Broad-leaved, Green	Ovoid, gray green	Broad-leaved, ovoid, subcircular, Green
No. of lobes	3–5	0–1	0–3
Bract size (length × width, cm)	1.1×1.3	1.2×0.2	1.1×0.5
Bract shape, No. of teeth	Heart-shaped, 4–6	Lanceolate, 1	Sword-shaped, 1–3
Bract jointing	Joint	No joint	No joint
Floral nectary	Intra-floral	Extrafloral	Extrafloral
Calyx	Cup-shaped	Wavy	Serration
Bract tooth	5–9	1	4–6
Corolla shape and color, corolla length (cm)	Yellow trumpet, around 2.1	Light pink, funnel-shaped, around 3.5	Pink trumpet, around 2.5
Color and size of spot	Rose	Purple	Red
Filaments colors	Pink interspersed with milky white	Rose	Light pink
Anther colors	Yellow	Rose	Light yellow
Pollen colors	Yellow	Milky white	Yellow
Stigma colors	Milky white	Light green	Milky white
Boll	Conical	Oval	/
Fiber	Spiral	Upright	/

Figure 1. Pictures of parent and hybrid plants. (A) The female *G. herbaceum* L. parent had a tower shape with shorter and more compact branches. (B) The male *G.*

nelsonii Fryx. was cylindrical with short and compact branches. (C) The diploid F_1 hybrid was tower-shaped, with longer branches and looser spacing but were infertile. (D) The tetraploid, S_1, were tower-shaped with long fruit branches and loose spacing. They were fertile.

Figure 2. Pictures of leaves and flowers. (A) *G. herbaceum* L. bore bell-shaped flowers with yellow corollas, rose basal patches, pink and white alternating filaments, yellowish anthers, yellow pollen, cup-shaped calyxes, heart-shaped bracts grown together separately rather than together, and were broad-leaved. (B) *G. nelsonii* Fryx. bore tubular-shaped flowers with light pink corollas, purple basal spots, rose filaments and anthers, milky pollen, wavy calyxes, lanceolate bracts that were detached rather than united, and ovoid leaf blades. (C) The F_1 hybrid had bell-shaped flowers with pink corollas, red basal patches, light pink filaments, light yellow anthers and pollen, serrated calyxes, sword-shaped bracts that grow separately rather than together, and had broad-leaved, ovoid, and subcircular leaves. (D) The S_1 had trumpet and funnel-shaped flowers with alternating red and white corollas, red basal patches, light pink filaments, yellow anthers and pollen, serrated and wavy calyxes, triangle dentate and ensiform bracts that grew separately rather than together, and had broad-leaved, ovoid, and subcircular leaves.

SSR molecular marker determination was carried out and 6 pairs of parental polymorphic primers (Table 2) were obtained from the screening. These 6 pairs of primers could be used to amplify the complementary bands from the parents in the F_1 hybrids (Fig. 3A), of which 2 primer pairs (NAU1052 and NAU3093) not only amplified bands that are characteristic to the parents but also characteristic bands of the hybrid itself (Fig. 3Ac,f), showing that the F_1 hybrids are true hybrids. Flow cytometry showed that the peak value of the control sample *G. herbaceum* was approximately 200 channels (Fig. 3B), the peak value of TM-1 was close to 400 channels (Fig. 3C), and the peak value of the F_1 hybrid was close to 200 channels (Fig. 3D), showing that the F_1 hybrid was a diploid. Root tip staining and microscopy

observations showed that the root tip chromosome number of the *G. herbaceum* and *G. nelsonii* control samples was 26 pairs (Fig. 3E,F), while the root tip chromosome number of the F_1 hybrid was also 26 pairs (Fig. 3G), which proved that the F_1 hybrid was a diploid.

Table 2. SSR polymorphism primer sequences

Primer number	Forward sequence (5'-3')	Reverse sequence (5'-3')
NAU1157	GAGTTTGGTTCTGGGTTGAG	GATCCTTTTCATCTCCTCCA
NAU1355	ATCTGTTTACGCCACTCTCC	CCAGCCTTTGACATTTTTCT
NAU1052	CGCAGATAAAGGATGGATTT	AGAGCTGGAGGACATAACAAA
JESPR156	GCCTTCAATCAATTCATACG	GAAGGAGAAAGCAACGAATTAG
BNL1679	AATTGAGTGATACTAGCATTTCAGC	AAAGGGATTTGCTGGCAGTA
NAU3093	GTCTTGAACCGGAACTTGAT	TCCTGTTGAACACCAAAGTG

Figure 3. Validation of S_1 by detecting DNA polymorphism, following ploidy analysis and microscopic observation. (A) Microsatellite loci identified by SSR primers including NAU1157 (a), NAU1355 (b), NAU1052 (c), JESPR156 (d), BNL1679 (e), NAU3093 (f). The novel bands produced in F_1 and S_1 are indicated by red and blue arrows, (1, *G. herbaceum* L.;2, *G. nelsonii* Fryx.;3, the F_1;4, the S_1). (B) Distribution peak of *G. herbaceum* L.at approximately 200 channels. (C) Distribution peak of TM-1 at approximately 400 channels. (D) Distribution peak of the diploid F_1 hybrid and the tetraploid S_1 at approximately 200 channels and 400 channels respectively. Fluorescence intensities of propidium-iodide stained genomic DNA. (E) The 26 chromosomes in *G. herbaceum* L. (F) The 26 chromosomes in *G. nelsonii* Fryx. (G) The 26 chromosomes in F_1 hybrid. (H) The 52 chromosomes in S_1 tetraploid.

The root tip cells (red arrows) and the chromosomes (black arrows) stained with Carbol fuchsin solution under 40× microscope.

Tetraploid S_1 generation and trait observations

After the apical growth point of the F_1 hybrid underwent chromosome doubling, the first large red flower grew in the apex of the plant after 27 days (Fig. 4A). The flower was full of pollen and a boll was formed through self-pollination (Fig. 4B). The boll contained 4 seeds (Fig. 4C). In 2018, the 4 seeds were sown in a pot and all germinated after 5 days. After the first true leaves were level, the seedlings were transplanted to the field. Floral buds appeared after 45-50 days and flowering occurred after 70-75 days. Boll opening occurred after 107-115 days. In Sanya, China, the 4 S_1 plants grew normally, with medium plant height and flowering that occurs throughout the year. The pollen formed was viable and could form a lot of bolls. The samples that were preserved were fertile. Chromosome doubling of the F_1 hybrids restored their fertility. We hypothesized that the 4 S_1 plants were all tetraploid hybrids and were assigned the code S_1(2n = 4x = 52, $A_1A_1G_3G_3$). The differences in the morphological characteristics between the 4 S_1 plants were not great. Most plants developed large red flowers, with radiating purple patches, and significant separation was not observed. The leaves were large and tough, and the plants were tall, and shrub-like(Fig. 1D). The plants could produce many flowers, release pollen, and fruit normally. The fruits formed were small bolls containing small seeds, and short, brown, and high-strength fibers (Fig. 5F). In particular, the S_1 plants retained some characteristics of *G. nelsonii* Fryx. For example, at low temperatures, the buds on the lower branches were extremely small (0.5 cm length), milky white in color, and did

not open until wilting, and had closed pollination. In contrast, flowers in the middle and top branches were large (4.4 cm length), opened normally, and had open pollination. The red flowers contained large purple patches, the bracts were opened, and the plants contain a lot of glands, but not the seed embryo.

Figure 4. Growth and formation of the synthetic tetraploid, S_1. (A) Tetraploid plants with a large pink flower accompanied by yellow pollen. (B) The growth process of the oval boll. (C) Four seeds with brown fibers.

Figure 5. Pictures of cotton bolls and fibers. (A) *G. herbaceum* L. had conical, smooth green and red bolls. The bulge at the top of the boll was distinctly cone-shaped. (B) *G. nelsonii* Fryx. had oval, green cotton bolls with fine hairs on the surface. The boll tip was not obvious. (C) Green pear-shaped boll of S_1 covered with downy hairs. (D) *G. herbaceum* L. had 3-chambered bolls with gray-white spiral cotton fibers. (E) *G. nelsonii* Fryx. had 3-chambered bolls with dark brown upright fibers. (F) The S_1 had 3-chambered bolls with brown fibers, and the seed hairs grew upright.

The differences in the morphological characteristics between the S_1 plants and F_1 plants is that S_1 plants were shorter(Tables 2), had larger and thicker leaves, produced viable pollen, could form bolls, seeds, and long fibers, with a boll number of 50 per plant (S1 Fig.). The samples were still fertile after preservation. Fertility was restored after the diploid F_1 hybrids underwent chromosome doubling. However, other S_1 traits were not different from the F_1 plants, including leaf shape and color, number of lobes, bract shape, calyx teeth shape, number of bract teeth, anther and pollen color, stem and leaf trichomes, bract extension, and extrafloral nectary(Fig. 2C,D).

Table 3. ***G. herbaceum*** **L. ×** ***G. nelsonii*** **Fryx. S_1, S_2 ~~morphology and fertility comparison~~**

Generation	Plant height (cm)	Colors of leaf	Leaf size (length×width, cm)	No. of lobes	No. of nectary on the back of leaf blade	Corolla color	Corolla length (cm)	Color and size of spot	Anther and Filaments colors	Bract size (length×width, cm)	Bract shape, No. of teeth (n)	Flower nectary	Boll shape, no. of boll loculi	Boll size (length× width, cm)	Sterility
S_1	150	Dark green	5.1×5.4	0–3	1 on primary vein	Pink,	Around 2.6	Red	Yellow, Light pink	1.2×0.6	Sword-shaped, 1–3	Extrafloral	Pear-shaped, 4	Generally, 3×2.5	A lot of bolls formed
S_2	75–170	Light green, Dark green	5.7×6.2–10.9×13.6	0–3	2, 3 at the primary vein and lateral vein	Pink, Light pink, Milky white, Red flower with white border	2.4–4.4	Red, Purple, Rose, None	Yellow, Light pink, Pink interspersed with milky white	1.3×0.7–1.6×1.1	Triangle, Dentate, Ensiform, 2–4	None, extrafloral, intra-floral	Pear-shaped, sunken navel, oval, irregular round boll, 3–4	2.7×2.3–3.2×2.6	Only 32 out of 145 plants had 1–7 bolls while no bolls were found in other plants

S_1 plants are similar to the F_1 plants as their morphological traits showed classical hybrid characteristics (Figs. 1,2). According to the 25-item investigation data analysis(Table 3), 10 items were intermediate between the parents including leaf shape and color(Fig. 2A-D), number of lobes, bract shape, calyx teeth shape, number of bract teeth, corolla size, number of anthers, boll diameter(Fig. 5A-C),fiber color, and fiber length, 2 items were greater than the parents including number of boll loculi and fiber tensile strength(Fig. 5D-F), 2 items were similar to the female parent including anther and pollen color(Fig. 2A,D), and 6 items were similar to the male parent including stem and leaf trichomes(Fig. 1B,D), flowering habit, bract extension, bract size(Fig. 2B,D), flower opening, and extrafloral nectary. In addition, maternal traits were dominant for the anthers, stamens, and stigmata(Fig. 2A,D), while paternal traits were dominant in the stem and leaf trichomes, and the boll shape and the boll trichomes (Fig. 5E,F).

SSR molecular marker determination showed that the 6 pairs of parental polymorphic primers(Fig. 3A) not only amplified complementary bands of the parents but also characteristic bands of the hybrid itself (Fig. 3Ac,f). This shows that the S_1 is also a true hybrid like the F_1. At the same time, this shows that the external genes were not introduced during the chromosome doubling of the F_1 to obtain the S_1. Flow cytometry showed that the peak value of the control sample *G. herbaceum* was close to 200 channels (Fig. 3B), the peak value of the TM-1 was close to 400 channels (Fig. 3C), and the peak value of the S_1 hybrid was close to 400 channels (Fig. 3D), showing that the S_1 hybrid was a tetraploid. Root tip staining and microscopy observations showed that the root tip chromosome number of the *G. herbaceum* and *G. nelsonii* control samples was 26 pairs (Fig. 3E,F) while the root tip chromosome number of the S_1 hybrid was 52 pairs (Fig. 3G), which proved that the S_1 hybrid was a tetraploid.

Examination of tetraploid S_2 segregation

The S_2 plants were completely different from the S_1, and showed abnormal and intense segregation. For example, for plant height, the shortest plant was 75 cm, which is shorter than the *G. herbaceum* L. female parent, while most plants were tall and thick shrubs, with the tallest plant having a height of 170 cm. Corolla color started to show segregation (S2 Fig.) as not only red corollas were produced but pink, red flowers with white borders, and milky white were seen but yellow flowers of the female parent were not seen(Fig. 2A). The leaves were generally large, and the longest leaf length being 8 cm and leaf width being 11 cm. Nectaries at the back of the leaves were also unique, as they were different from normal leaves that had one nectary at the base of the primary vein, but both primary and lateral veins contained nectaries. Some leaves had 2 while other leaves have 3 nectaries(Table ~~3). This multiple nectary trait in the leaves~~ is rarely seen in the *Gossypium* genus.

Observations also showed that the S_2 morphology was associated with fertility. Plants that resembled a pagoda like the female parent (*G. herbaceum* L.) produced more bolls and seeds, with 10-20 seeds per boll. In contrast, plants that resembled the male parent (*G. nelsonii* Fryx.) were inclined to have a higher ratio of sterility and few fertile plants that produced fewer bolls, with each boll contain only a few seeds (1-5).

Intense segregation also occurred for fertility. Some plants grew slowly and were weaker, flowered later, and had fewer flowers, but could normally disperse pollen and fruit, but the fruits were small and not upright. Other plants showed exuberant growth, flowered earlier, and produced more flowers and fruits, and the fruits were large and

upright. Overall, the fibers produced were brown in color, but some bolls contained fibers with two different colors, namely brown and light green fibers (S3 Fig). We found that a variety of colored fibers developed in the S_2 cotton bolls, including brown fibers in a 4-chambered boll and light green and brown cotton fibers in a 3-chambered boll (S3 Fig. A,B). Notably, the fiber Micronaire reading of the S_2 fibers was 9.6, determined using the Macron Airflow Meter in the fiber-testing center of the Cotton Research Institute of Chinese Academy of Agricultural Sciences. This reading was far above the parental range and may be the highest value reported to date. The S_2 fibers were very thick and provided us with the raw materials for making non-woven fabrics(S3 Fig. C). These observations indicated that the fiber characteristics of S_2 were recessive and dynamic.

The above results indicate that the S_2 generation underwent intense segregation in both morphological characteristics and fertility. This phenomenon exists in varying degrees in other interspecific hybrids that we generated and is common in segregated generations. However, it was particularly prominent here in the S_2 generation of this hybrid. Table 3 compares the observation data of the S_2 and S_1 plants.

Table 4. TM-1×(*G. herbaceum* L. × *G. nelsonii* Fryx.S_1) hybridization results and fertility examination

No. of flowers pollinated	Effective boll number	Boll-forming rate (%)	No. of normal embryos	Number of three-way F_1 plants	Number of three-way F_1 low-fertility plants
278	16	5.75	9	7	2

Determination of disease resistance in the tetraploid S_2

For each plant, there were 108 nutrient pots, and each pot contained 5 seedlings. After the first true leaf was level, the plants were inoculated with a fixed concentration of *Verticillium dahliae* Kleb. suspension (Fig. 6A). After 20 days the percentage of infected plants in the control group was 80 %. Results showed that when the inoculation was carried out indoors, the seedlings developed disease at the cotyledon stage. There were 456 plants from the susceptible strain cv. *Jimian11* that developed disease, with an incidence of 84.44 % and disease index of 33 (Fig. 6B). The incidence and disease indices of the S_2, *G. herbaceum* and *G. nelsonii* were all 0 and they were considered to be immune (Fig. 6C-E). No disease occurred in any of the plants inoculated with sterile water. From this, it was preliminarily determined that the tetraploid S_2 was germplasm material with potential disease resistance. In future study plans, we will examine and validate the disease resistance in the S_2 in specific plots in the field, to provide reliable intermediate materials for the cultivation of disease resistant *G. hirsutum* L.

Figure 6. Identification of resistance to *Verticillium dahliae* Kleb. (A) The pathogen of the candida conidia is indicated with the black arrow. (B) Disease-susceptible upland cotton *Jimian11*, with disease symptoms of yellowing, necrosis, and leaf abscission indicated by red arrows. (C) The resistant S_1.(D) Resistant *G. nelsonii* Fryx. (E) Resistant *G. herbaceum* L.

Tetraploid S_1 breeding application test

From an analysis of the effective boll number and boll-forming rate (Table 4), the tetraploid S_1 showed some compatibility for hybridization with the TM-1 *G.*

hirsutum L. plants. In 2018, the S_1 was hybridized with the TM-1 *G. hirsutum* L. plants and 16 effective bolls were obtained, but they were small and deformed (Fig. 7A-D). These grew into seven three-way F_1 plants, of which only 2 grew normally and were fertile (Fig. 8A-D). The remaining 5 plants did not grow normally (some grew to the flowering stage and growth stopped, some died, some were infertile, and some had low fertility). This is an inevitable reason from three-way species crossing between TM-1× (*G. herbaceum* L. × *G. nelsonii* Fryx.S_1). Hence, the 5 three-way F_1 hybrids cannot be used for selective breeding.

Figure 7. Cotton bolls from cotton TM-1 and from the F_1 hybrid of TM-1 × S_1 tetraploid. (A) The normal conical boll of TM-1 shown by the red arrow. (B) The abnormally conical boll of TM-1 × S_1 F_1 plants on the right and on the left(C), indicated by yellow arrows. (D) The deformed hybrid boll of F_1 plants indicated by yellow arrows.

Figure 8. Cotton TM-1, the S_1 tetraploid and F_1-1, F_1-2 plants. (A) Cotton TM-1 was tower-like with longer and looser branches. (B) S_1 was tower-like with shorter and more compact branches. (C) Hybrid F_1-1 was tower-like with shorter and looser branches and pink flowers (red arrow). (D) Hybrid F_1-2 was tower-like with longer and looser branches and had white flowers (white arrow).

The ~~two three-way F_1~~ plants described above, that grew normally and were fertile, were transplanted to fields and named F_1-1 and F_1-2 (Fig. 8C,D). Field morphological observations found that the F_1-1 plant retained significant wild cotton characteristics, which were mostly paternal traits(Fig. 9B,C). The morphology of the F_1-2 was an intermediate *G. hirsutum* L. and had mostly inherited material traits(Fig.

9A,D). The significant differences between the two plants was the corolla color, presence of spots, bract characteristics, trichomes, and boll fertility. The F_1-1 plant had a pink-colored corolla and red spots, bracts that were separate and not jointed(Fig. 9C）, and the stems, branches, leaves, and bolls had a large number of trichomes. The boll number was low and the bolls were deformed(Fig. 9G）. The seed embryos were small and not fertile. The F_1-2 plant had a milky white corolla, no spots were present, and bracts that were jointed(Fig. 9D）. The surface of the stems, branches, leaves, and bolls were smooth and there were no trichomes. The bolls developed into a funnel shape and the seed embryos were large and fertile(Fig. 9H）.

Figure 9. Pictures of leaves, flowers, and bolls of cotton TM-1, the S_1 tetraploid, and of F_1-1 and F_1-2 plants. (A) Cotton TM-1 bore bell-shaped flowers with milky corollas, milky filaments, milky anthers and pollen, without basal patches, wavy calyxes, heart-shaped bracts grown together, and were broad-leaved. (B) S_1 had bell-shaped flowers with pink corollas, milky filaments, yellow anthers and pollen, red basal patches, serrated calyxes, sword-shaped bracts grown separately rather than together, and had heart-shaped or broad leaves. (C) Hybrid F_1-1 had bell-shaped flowers with pink corollas, milky filaments, yellow anthers and pollen, red basal patches, serrated calyxes, heart-shaped bracts grown separately rather than together, and broad leaves. (D) Hybrid F_1-2 bore bell-shaped flowers which were milky white in the middle with a light red corolla border, milky filaments, milky anthers and pollen, without basal patches, wavy calyxes, heart-shaped bracts grown together, and broad leaves. (E) A normal conical boll of cotton TM-1. (F) A green pear-shaped boll of S_1 with a sunken navel. (G) A downy green boll of F_1-1 that was deformed, indicated by the red arrow. (H) A normal conical boll of F_1-2.

The tetraploid S_1 had some potential for breeding applications, but there was severe segregation in its progeny and its fertility was decreased, which affects further breeding applications. Notably, as a limitation, we have only preliminarily assessed the authenticity of the F_1 generation obtained from the crosses between the TM-1 and S_1 through morphological characterization. Further studies are required to confirm that they are true hybrids using cytology and molecular genetics techniques.

Discussion

Wild cotton varieties provide a great treasury of traits that could be utilized to improve commercial cultivars [27]. There are at least 30 varieties of wild cotton, widely distributed in the Americas, Australia, Africa, and Asia that have undergone long-term natural selection to accumulate rich genetic diversity conferring adaptability to adverse conditions and resistance to environmental onslaughts and diseases. The cultivated varieties present today have also undergone a long period of artificial selection compared with wild varieties to significantly improve yields and fiber quality. However, even after long-term intensive farming and artificial selection, some favorable resistant phenotypes have not developed in cultivated varieties and were even excluded in some situations by excessive selection. Therefore, despite the continuous improvements in yields and fiber quality, the disease resistance of commercial cotton cultivars has weakened. Resistance breeding programs are now being conducted to transfer the disease resistance genes from wild cotton to cultivated cotton, and to re-diversify the genetic makeup of the cultivated cotton.

The superior qualities of cotton fiber, including its length, strength, fineness as well as the disease resistance of wild cotton, has been successfully transferred to

cultivated cotton [28,29]. However, diploid wild cotton and tetraploid cultivated cotton are biologically isolated. While increased genetic diversity could be achieved from distant hybridization, they are often genetically incompatible, forming a barrier to the full utilization of wild diploid cotton varieties [30-32]. In the present study, we successfully obtained an infertile interspecific F_1 hybrid through distant hybridization, overcoming the genetic incompatibility barriers between two distant species. We further doubled the chromosomal content of this F_1 hybrid with colchicine treatments to overcome the significant challenge of interspecific hybrid sterility, to successfully synthesize a new tetraploid cotton species S_1.

We found that the S_1 progeny in the field showed a phenomenon of gradual decline in growth. The morphological traits of the S_1 plants were generally consistent, with normal growth, flowering, and fruiting. However, the S_2 plants showed violent segregation and resulted in different types of plant shapes and corolla shapes, and huge differences in the flowering and fruiting characteristics. The growth of many plants in the S_3 to S_5 generations were suppressed and only a few plants could flower. In severe cases, the plants could not flower and only a small number of plants could bear fruit. This shows that the S_1 was genetically unstable germplasm and there is still a risk of natural segregation. The genetic instability of the S_1 severely impedes its use in breeding. This problem will prevail until the new germplasm obtained from hybridization cannot be used to achieve the aim of hybridization. Therefore, in future studies, there is a need to control segregation in hybrids and establish new genetic isolates. The genetic instability of the S_1 severely hindered the utilization of the new cotton germplasm for breeding. We were unable to resolve this issue in the present study; thus, the control of hybrid separation for the successful establishment of stable genetic strains needs to be explored in future research. It should be mentioned that the

S_2 was used as an experimental material as it was a population with genetic segregation and with a rich genetic diversity. It is also an ideal population for the screening of disease resistant varieties in conventional hybridization and breeding. Furthermore, the number of S_1 plants obtained was low and does not facilitate the execution of this experiment.

Molecular markers are specific pieces of DNA that reflect differences in the genomes of individuals or populations [33]. Since the discovery that microsatellite markers are uniformly distributed on cotton chromosomes, SSR markers have been used to monitor the infiltration of heterologous chromosome fragments in the offspring of interspecific hybrids [34,35]. In the present study, SSR detection revealed the presence of new specific bands in the hybrids (Fig. 3Ac,f) that were not detected in the parents, indicating that new combinations or mutations of chromosomes are present in the interspecific progeny (Genomic A and Genomic C). Our results also support the hypothesis that distant hybridization is an important driver of new mutations and species in plant evolution [36,37].

There is potential for genes from the new synthetic allotetraploids to fuse with favorable genes from *G. herbaceum* L. and *G. nelsonii* Fryx., including genes that encode good fiber quality, or resistance to diseases, pests, and drought. This will allow their transfer to upland cotton through hybridization, to further improve the resistance and fiber quality of the upland cotton [22]. We tested this possibility by performing preliminary disease resistance testing of the new S_2 species in the greenhouse. We found that the new species presented high disease resistance to the fungal pathogen *Verticillium dahliae* Kleb. This important agronomic characteristic

provided a new and important resource for cotton disease resistance breeding programs.

When we examined whether the newly synthesized allotetraploid S_1 could be applied in cotton breeding via hybridization with upland cotton TM-1, we found two types of plants in the F_1 hybrid offspring, namely F_1-1 with red flowers and F_1-2 with white flowers. The F_1-1 plants died upon the onset of anthesis, indicating that this plant type could not be utilized in crossbreeding. The F_1-2 plants grew normally until flowering and fruiting, but their seeds could not germinate normally, indicating that this type had the potential for breeding applications, but additional research is required to overcome the key difficulties in promoting seed germination in each generation. This phenomenon of unsuccessful germination of F_1-2 seeds and root tip wilting had previously been observed during the early generations of the amphiploid *G. arboretum* L. hybridized with *G. bickii* Prokh. [38]. The variation in hybrid phenotypes may be related to gene overlap, new genetic variation, chromosome rearrangement, or a variety of other factors [39-41].

The tetraploid S_1 is a new germplasm obtained by chromosome doubling of the diploid F_1 hybrid and is a polyploidy event in cotton. This not only restores fertility to sterile diploid F_1 hybrids and the tetraploid S_1 generation obtained showed segregation, which increases the phenotypic mutations in the species, enabling breeders to select for required traits. After the polyploid was generated, its stress resistance not only did not decrease but biotic stress resistance was maintained. For example, the preliminary examination showed that the disease resistance of the tetraploid S_2 was 1 grade better than the female parent (*G. herbaceum*) and the same level as the male parent (*G. nelsonii*), and its disease resistance inheritance was closer

to the male parent. In addition, the fiber quality of the tetraploid S_2 exceeded that of both parents. The S_2 generation had high potential for breeding owing to its boll-forming properties, diversity of fiber colors, and fiber quality. These traits are also of great value for breeding programs.From this we can see that diploid F_1 hybrid polyploidy has important significance for cotton evolution, as the genome structure changed and the species genetic diversity enriched as well as the species adaptability and fiber quality were improved.

Thus far, we have only performed preliminary characterizations of the newly synthesized allotetraploid cotton S_1.We have proposed to perform more in-depth and comprehensive research to extract the valuable information from this new cotton tetraploid, so as to provide a more accurate theoretical basis for cotton breeding and genetic evolution.

Acknowledgements

We are grateful to Dr Kunbo Wang and Dr Fang Liu of the Cotton Research Institute of Chinese Academy of Agricultural Sciences for kindly providing us pollen of *G. nelsonii* Fryx. to use at the National Wild Cotton Nursery, Sanya, China.

We would like to thank Editage (www.editage.cn) for English language editing.

References

1. P.A. Fryxell.A revised taxonomic interpretation of *Gossypium* L.(Malvaceae).Rheedea.1992;2(2):108-165.
2. Wang Kunbo,Wendel Jonathan F.,Hua Jinping.Designations for individual genomes and chromos[illegible]es in Gossypium.J.Cotton.Res. 2018;1:3.doi:10.1186/s42397-018-0002-1
3. Wendel,Jonathan F.,Brubaker,Curt L.,Percival, A. Edward.Genetic diversity in *Gossypium hirsutum* and the origin of upland cotton. American Journal of Botany.1992;79: 1291–1310.
4. M.J. Iqbal, O.U.K. Reddy, K.M. El-Zik & A.E. Pepper.A genetic bottleneck in the 'evolution under domestication'of upland cotton *Gossypium hirsutum* L. examined using DNA fingerprinting. Theoretical and Applied Genetics.2001;103: 547–554.
5. Campbell B, Saha S, Percy R, Frelichowski J, Jenkins J, Park W, et al.Status of the global cotton germplasm resources. Crop Science. 2010;50: 1161–1179.
6. David D. Fang, Lori L. Hinze, Richard G. Percy, Ping Li, Dewayne Deng & Gregory Thyssen. A microsatellite-based genome-wide analysis of genetic diversity and linkage disequilibrium in Upland cotton (*Gossypium hirsutum L.*) cultivars from major cotton-growing countries. Euphytica.2013;191: 391–401.
7. Jonathan F Wendel, Richard C Cronn.Polyploidy and the evolutionary history of cotton.Advances in Agronomy.2003.78,139-186.
8. Hutchinson JB, Silow RA, Stephens SG.The evolution of *Gossypium* and the differentiation of the cultivated cottons. The Quarterly Review of Biology.1949;24(2):143-144.
9. Satya Narayan Jena,Anukool Srivastava,Krishan Mohan Rai,Alok Ranjan,Sunil K. Singh,Tarannum Nisar,et al. Development and characterization of genomic and expressed SSRs for levant cotton (*Gossypium herbaceum* L.). Theoretical and Applied Genetics. 2012;124: 565–576.
10. J.E. Endrizzi, L.L. Phillips.A hybrid between *Gossypium arboreum* L.and *G.raimondii* Ulb.Can.J.Genet.Cytol.1960;2(4),311-319.
11. H. Muramoto. Hexaploid cotton:some plant and fiber properties.Crop Sci. 1969;9(1),27-29.
12. Li Binglin，Zhang Xinrun.Studies on the Hybrid between *G. arboreum* L and *G.bickii* Prokh.Acta Genetica Sinica. 1987;14(2),121-126.
13. Yuqiang Sun, Xianlong Zhang, Yichun Nie, Xiaoping Guo, Shuangxia Jin & Shaoguang Liang.Production and characterization of somatic hybrids between

upland cotton (*Gossypium hirsutum*) and wild cotton (*G.klotzschianum* Anderss) via electrofusion.Theor.Appl.Genet. 2004;109(3),472-479.

14. X.S.Yu, B.J.Chu, R.E.Liu, J.Sun, Joseph Jones Brian, H.Z.Wang,et al.Characteristics of fertile somatic hybrids of *G.hirsutum* L.and *G.trilobum* generated via protoplast fusion.Theor.Appl.Genet. 2012;125(7),1503-1516.
15. Ganesh S. Newaskar Vivek P. Chimote Subhash S. Mehetre Ashok S. Jadhav. Interspecific hybridization in *Gossypium* L.:characterization of progenies with different ploidy-confirmed multigenomic backgrounds.Plant Breed. 2013;132(2),211-216.
16. Xia Zhang,Caijiao Zhai,Linchi He,Qi Guo,Xianggui Zhang,Peng Xu.et al.Morphological,cytological and molecular analyses of a synthetic hexaploid derived from an interspecific hybrid between *Gossypium hirsutum* and *Gossypium anomalum*.The Crop Journal. 2014;2(5),272-277.
17. Liu Q, Chen Y, Chen Y, Wang Y, Chen J, Zhang T. et al.A New Synthetic Allotetraploid ($A_1A_1G_2G_2$) between *Gossypium herbaceum* and *G. australe*:Bridging for Simultaneously Transferring Favorable Genes from These Two Diploid Species into Upland Cotton.PLoS One. 2015;10(4), e0123209. doi: 10.1371/journal.pone.0123209.
18. Mavromatis, Athanasios G. Pankou, Chrysanthi I. Vlachostergios, Dimitrios N. Xynias, Ioannis N. Roupakias, Dimitrios G. Hybridization between cotton and Malvaceae species as a tool for production of partial interspecific aneuploid cotto n plants. Euphytica.2018;214(10). doi:10.1007/s10681-018-2257-5.
19. Guo W,Cai C, Wang C,Han Z,Song X, Wang K,et al. A microsatellite-based, gen e-rich linkage map reveals genome structure, function and evolution in *Gossypiu m*. Genetics.2007;176:527–541. Doi:10.1534/genetics.107.070375
20. J. Zhang, W. Guo & T. Zhang.Molecular linkage map of allotetraploid cotton (*Go ssypium hirsutum* L.× *Gossypium barbadense* L.) with a haploid population. Theo retical and Applied Genetics. 2002;105:1166–1174. Doi:10.1007/s00122-002-110 0-4
21. Jun Zhang. Fast screening of microsatellite markers in cotton with PAGE/silver st aining. Cotton Science Sinica.2000;12(5): 267–269.
22. Di Chen,Yuxiang Wu,Xiling Zhang,Fuguang Li. Analysis of [*Gossypium capitis-viridis*×(*G.hirsutum*× *G.australe*)^2]Trispecific Hybrid and Selected Characteristic s.PLoS One. 2015;10(6),e0127023.doi:10.1371/journal.pone.0127023.

23. D W Galbraith,K R Harkins,J M Maddox,N M Ayres,D P Sharma,E Firoozabady. Rapid flow cytometric analysis of the cell cycle in intact plant tissues. Science (N ew York, N.Y.). 1983;220(4601),1049-1051.
24. Wu Yuxiang,Yang Fuhong,Zhao Xiaoming, Wude Yang. Identification of Tetrap loid Mutants of Platycodon grandiflorus by Colchicine Induction. Caryologia.201 1;64(3), 343-349.
25. Ma Ping, Huang H C, Li Shezeng, Tang Wenhua. A new inoculation method for verticillium wilt on cotton and its application in evaluating pathogenesis and host resistance.Acta Phytopathol.Sin. 2004;34(6),356-541.
26. Peng S,Lu XL,Gao F,Li GY, Li H.Study on a new rapid inoculation method for V erticillium wilt and fusarium wilt of cotton.J.Cotton Sci.2008;20(3),174-178.
27. J.E.Endrizzi,E.L.Turcotte,R.J.Kohel.Genetics,cytology,and evolution of *Gossypium*.Adv.Genet. 1985;23,271-375.
28. T W Culp,D C Harrell.Breeding methods for improving yield and fiber quality of upland cotton (*Gossypium hirsutum* L.).Crop Science. 1973;13,686-689.
29. Qian Siying,Huang Junqi,Peng Yuejin,Zhou Baoliang,Ying Miaocheng,Shen Duanzhuang.et al.Studies on the hybrid of *G.hirsutum* L.and *G.anomalum* Wawr.& Peyr.and application in breeding.Sci.Agric.Sinica. 1992;25,44-51.
30. Oakes,A.J.A Sterility in certain *Gossypium* hybrids.I. Prefertilization phenomena.Can.J.Genet Cytol. 1966;8(4),818-829.
31. Erik J.Sacks.Ovule rescue efficiency of *Gossypium hirsutum*× *G. arboreum* progeny from fieldgrown fruit is affected by media composition and antimicrobial compounds.Plant Cell Tissue & Organ Culture.2008;93(1),15-20.
32. S.Ganesh Ram,S.Hari Ramakrishnan,V.Thiruvengadam,J.R.Kannan Bapu.Prefertilization barriers to interspecific hybridization involving *Gossypium hirsutum* and four diploid wild species.Plant Breed. 2008;127(3),295-300.
33. H.Benbouza, J.M.Lacape, J.M.Jacquemin,B.Courtois,F.B.H.Diouf,D.Sarr,et al.Introgression of the low-gossypol seed & high-gossypol plant trait in upland cotton:Analysis of [(*Gossypium hirsutum*×*G. raimondii*)×*G.sturtianum*] trispecific hybrid and selected derivatives using mapped SSRs.Molecular Breeding.2010;25(2),273-86.
34. L Siu,S Saha,D Stelly,B Burr,RG Cantrell.Chromosomal assignment of microsatellite loci in cotton.Journal of Heredity.2000;91(4),326-32.

35. N H Barton.The role of hybridization in evolution.Molecular ecology. 2001.10(3),551-568.
36. Yuannian Jiao,Norman J. Wickett,Saravanaraj Ayyampalayam,André S. Chanderbali,Lena Landherr,Paula E. Ralph,et al.Ancestral polyploidy in seed plants and angiosperms. Nature. 2011;473(7345),97-100.
37. Soltis,P.S.,Soltis,D.E.The role of genetic and genomic attributes in the success of polyploids.Proc Natl Acad Sci. 2000;97(13),7051-7057.
38. Zhongfu Ni, Eun-Deok Kim, Misook Ha, Erika Lackey, Jianxin Liu, Yirong Zhang,et al.Altered circadian rhythms regulate growth vigour in hybrids and allopolyploids.Nature. 2009;457,327-331.
39. Kashkush K,Feldman M,Levy AA.Gene loss,silencing and activation in a newly synthesized wheat allotetraploid.Genetics.2002;160(4),1651-1659.
40. Loren H.Rieseberg.Evolution:replacing genes and traits through hybridization.Curr. Biol. 2009;19(3),119-122.
41. H Shaked,K Kashkush,H Ozkan,M Feldman,A A Levy.Sequence elimination and cytosine methylation are rapid and reproducible responses of the genome to wide hybridization and allopolyploidy in wheat.Plant Cell. 2001;13(8),1749-1759.

Supporting information

S1 Fig. Fruits of the tetraploid S_1. Multiple bolls of S_1 on multiple fruit branches with the sunken navel of the boll tip showing an ergonomic shape with strong bolls.

S2 Fig. Flowers of the tetraploid S_2. (A) A pink, cylindrical flower with pink corolla. (B) A funnel-shaped flower with light pink corolla. (C) A funnel-shaped flower with white and pink alternating corolla. (D) A white, closed flower. (E) A bell-shaped flower with red corolla and a clear rose-colored spot on the petal base. (F) A semi-closed flower with pink corolla. (G) A bell-shaped flower with pale pink corolla. (H) A small flower with white corolla and no spot on the petal base.

S3 Fig. Fiber colors of the tetraploid S_2. (A) A four-chambered boll with brown fibers. (B) A three-chambered boll with light green and brown cotton fibers. (C) The brown cotton fibers of S_1 with an average fiber length of 13 mm.

Figure Legends

Figures:

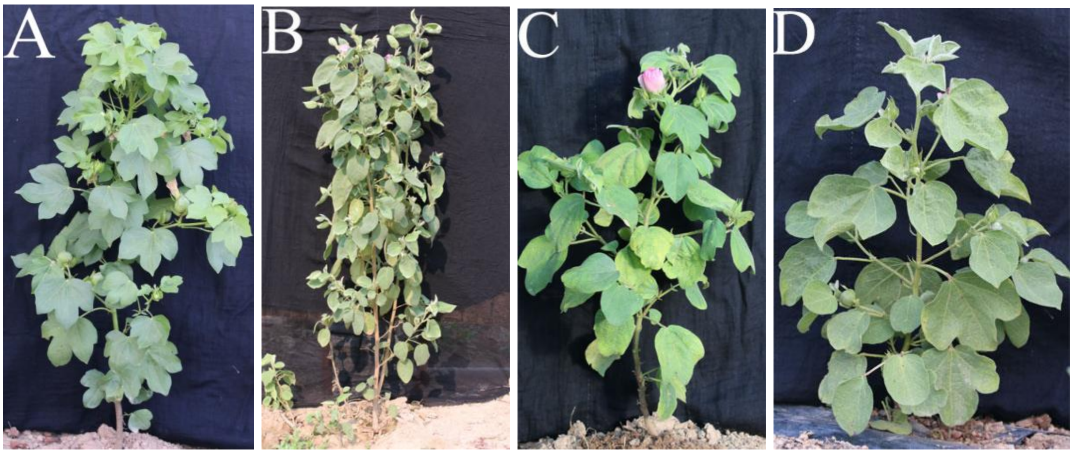

Figure 1

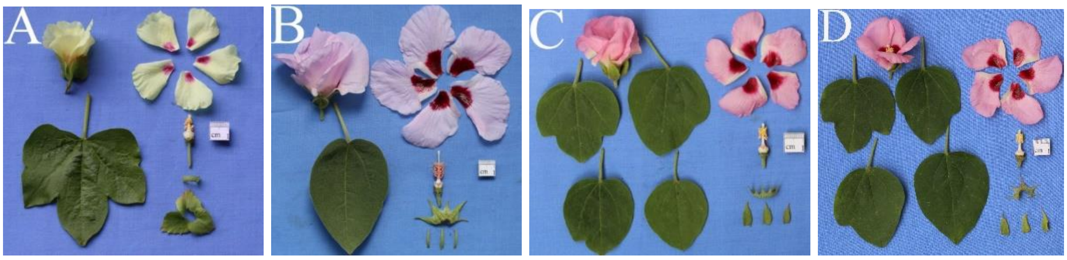

Figure 2

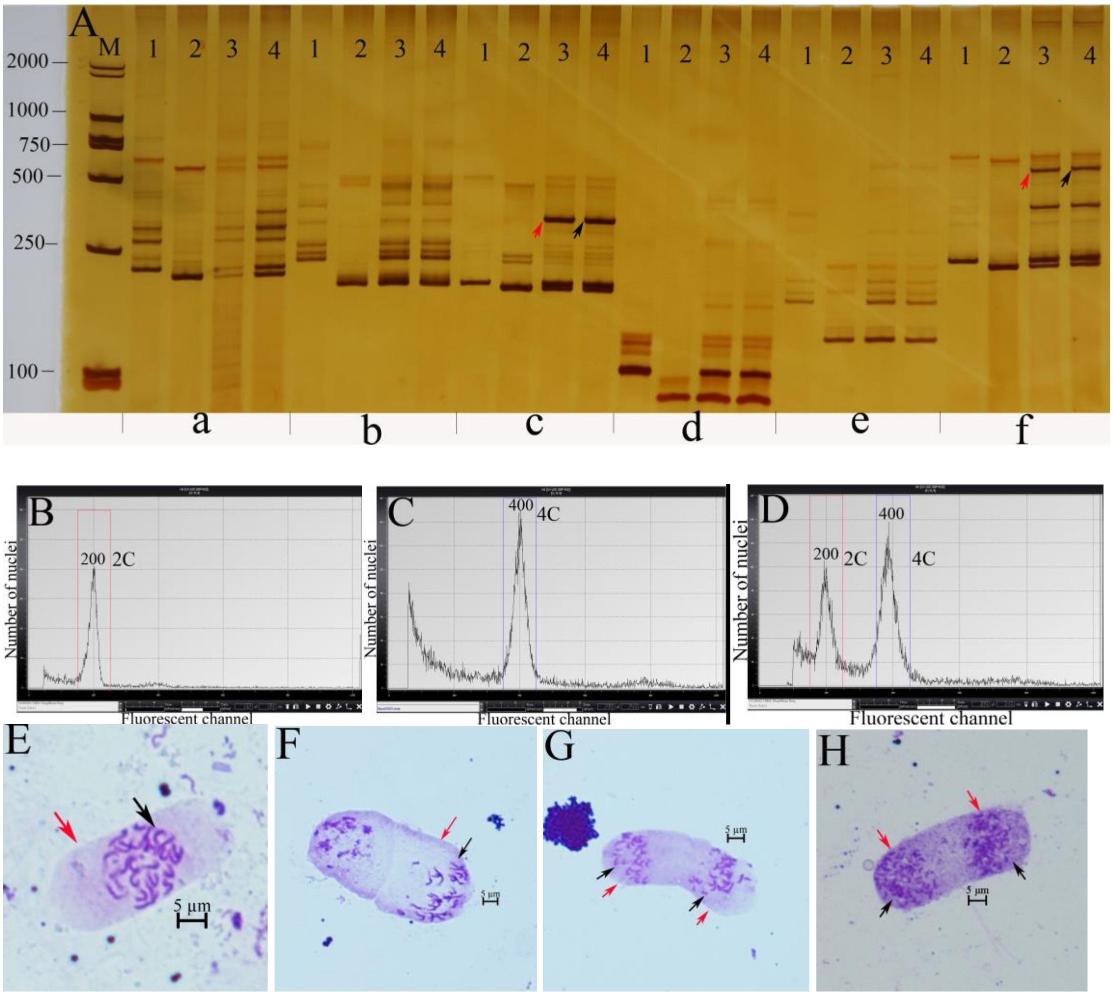

Figure 3

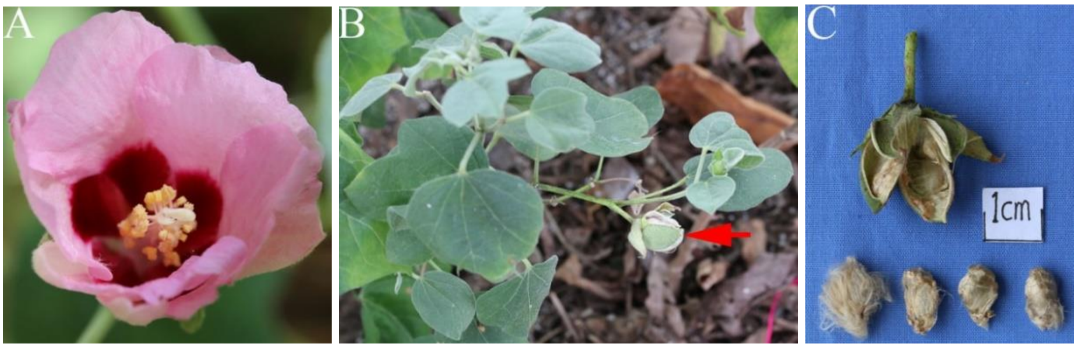

Figure 4

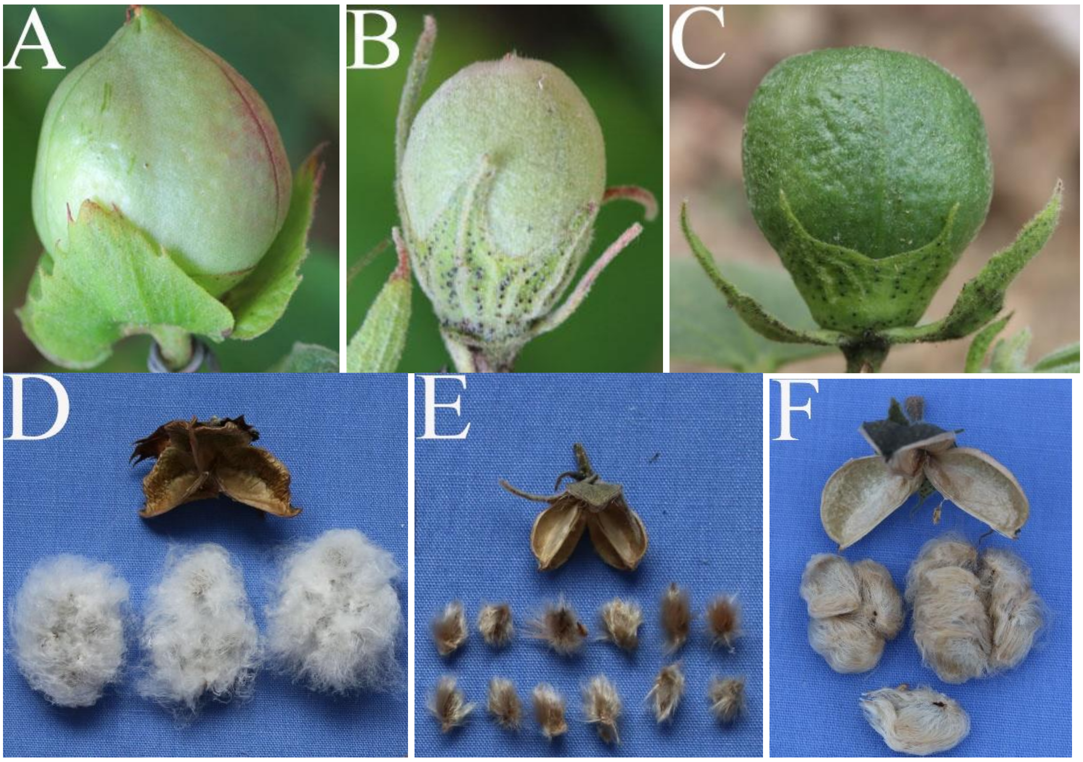

Figure 5

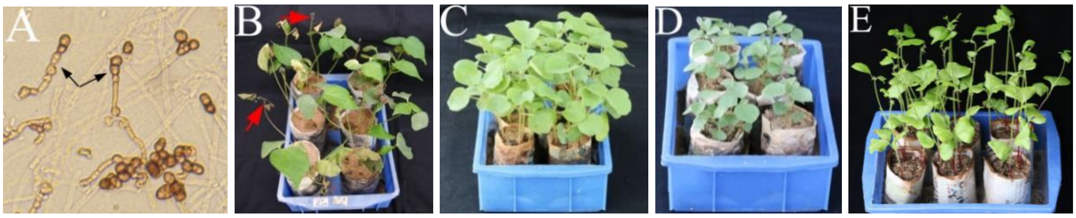

Figure 6

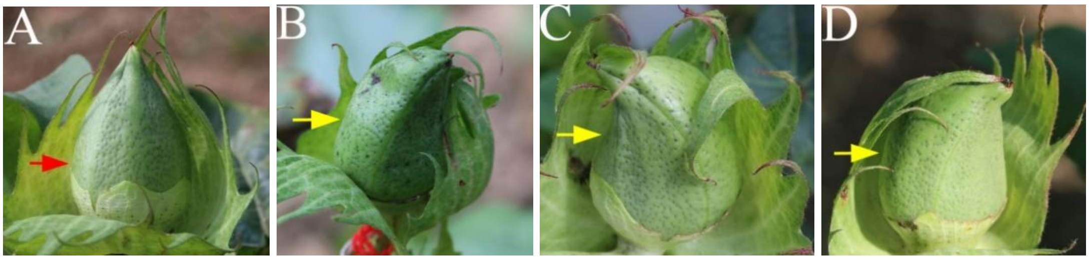

Figure 7

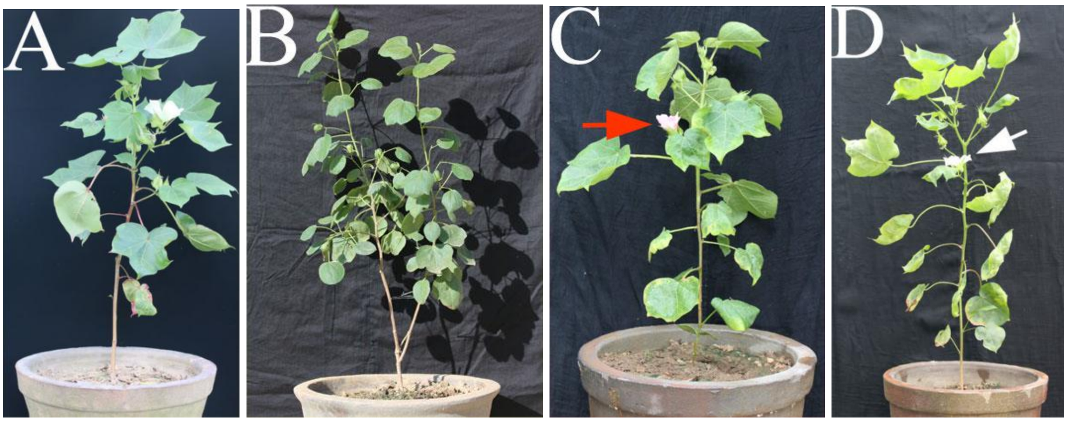

Figure 8

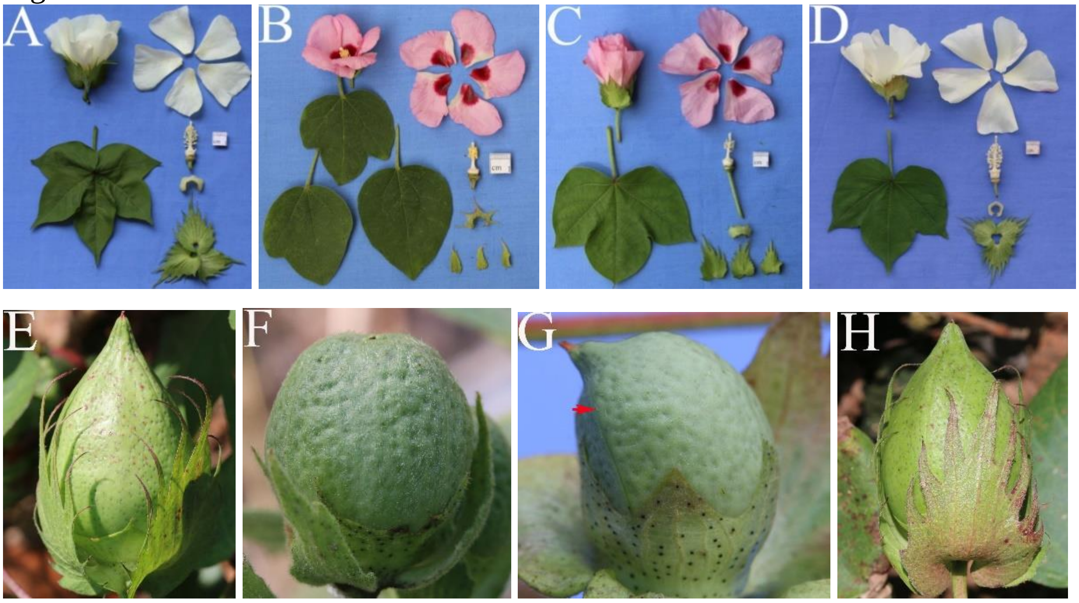

Figure 9

Supporting information

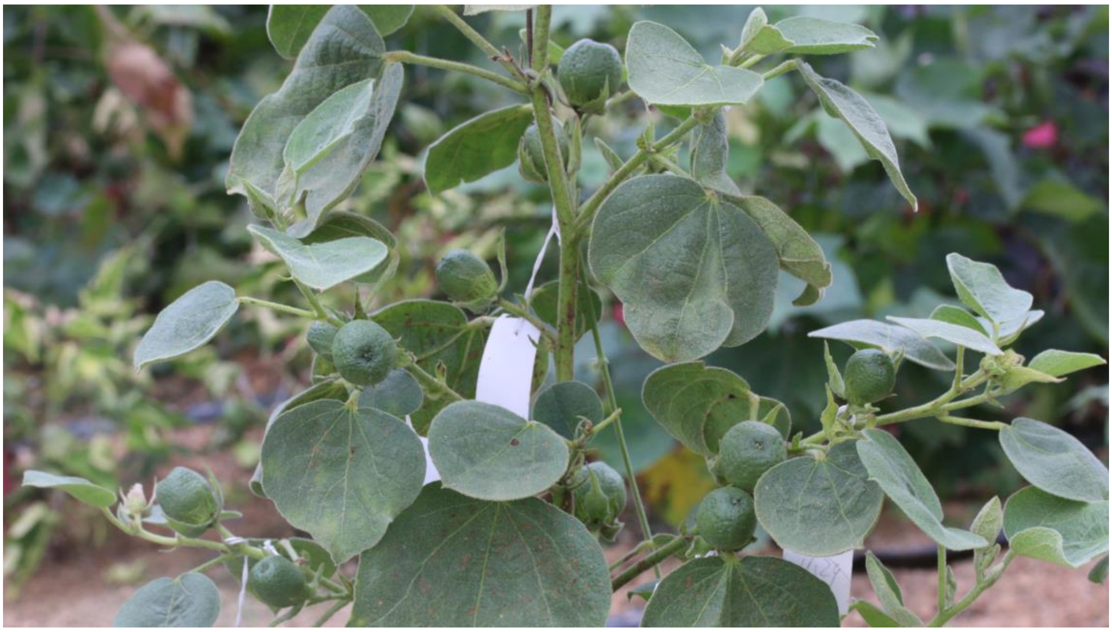

Supporting Figure 1

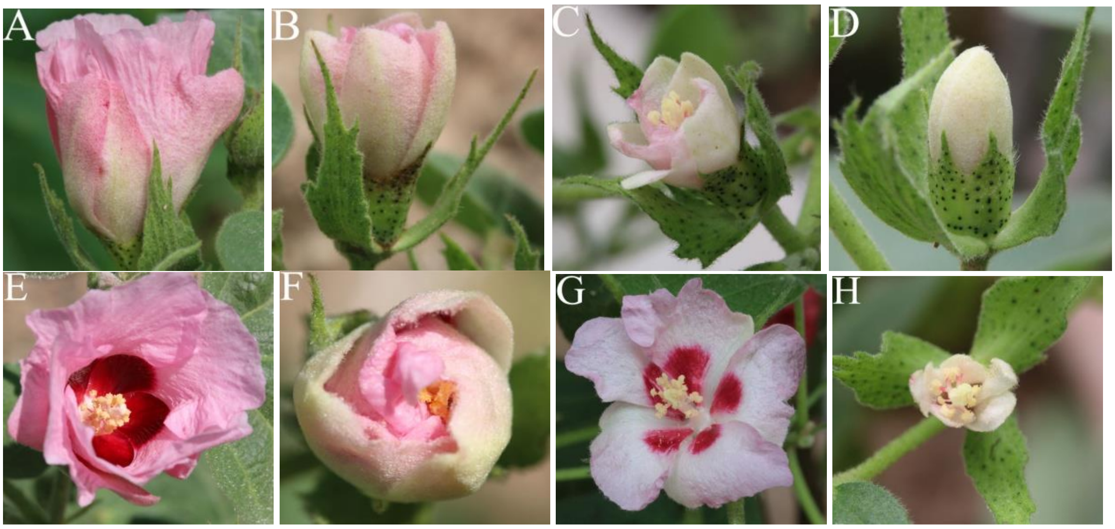

Supporting Figure 2

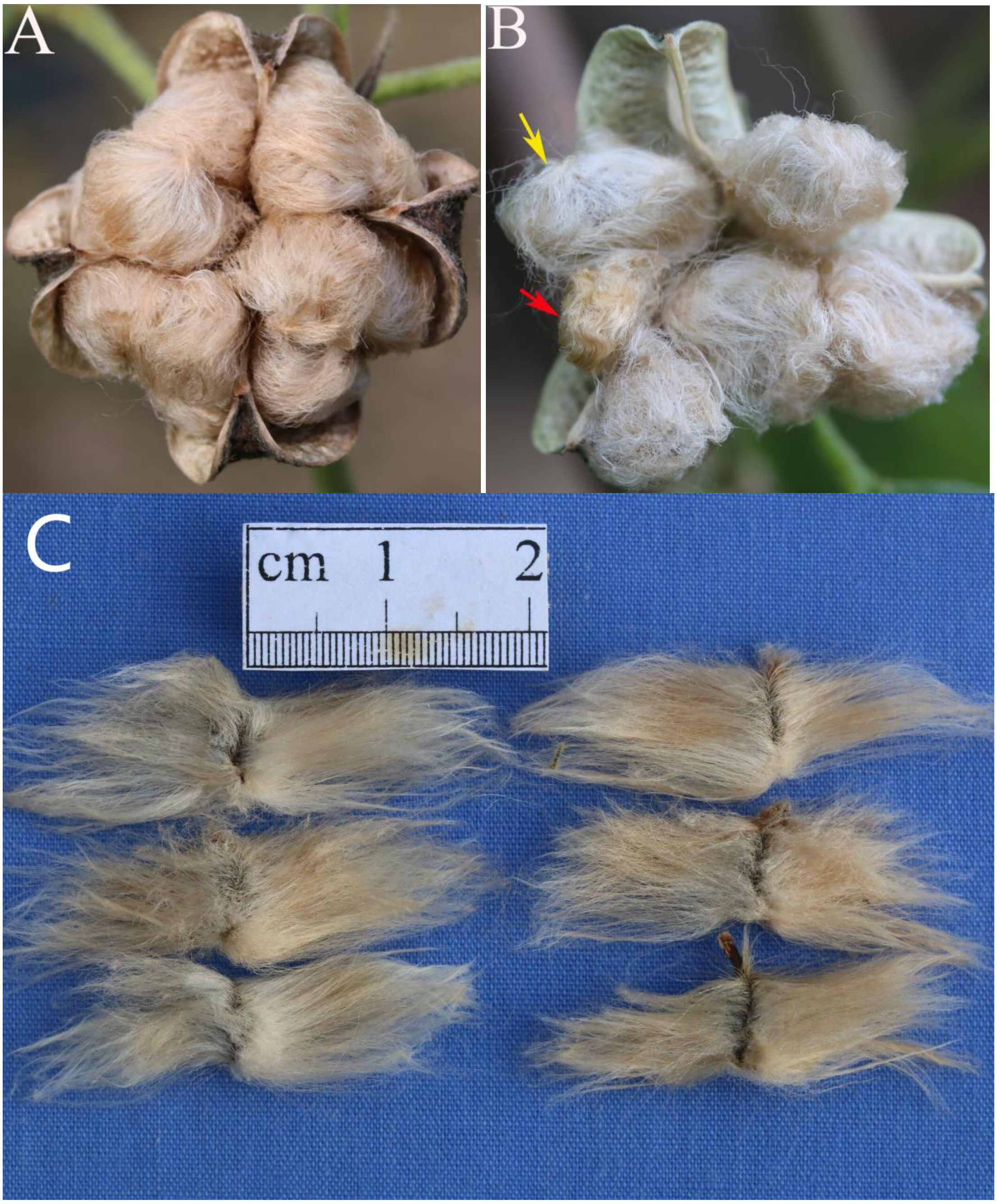

Supporting Figure 3

A cover letter describing your response to our editorial requests

Dear Editor,

First of all, I would like to thank the editorial staff for their preliminary approval of our article. At the same time, I would also like to thank the review experts for their careful review and valuable Suggestions.

We will respond to the questions raised as soon as possible, and will answer them truthfully one by one,and try our best to revise and write the article as standardized as possible.

I uploaded the original photo of the article picture, the processing format of this article pictures,If you have any questions, please reply immediately and we will respond as soon as possible.

lastly, we hope this article could be accepted by dear editing department and published as soon as possible.
Sincerely

Di Chen
2020-05-23

Reviewer Comments:

5. Review Comments to the Author

Please use the space provided to explain your answers to the questions above. You may also include additional comments for the author, including concerns about dual publication, research ethics, or publication ethics. (Please upload your review as an attachment if it exceeds 20,000 characters)

Reviewer #1:

The manuscript “Morphological and genetic description of a novel synthetic allotetraploid of *Gossypium herbaceum* L. and *Gossypium nelsonii* Fryx” created a new germplasm of cotton through remote hybridisation for a resistant source of wild cotton. *G. herbaceum* L. crossed with *G. nelsonii* Fryx. to produce an interspecies F1 hybrid cotton. Colchicine was used to double its chromosomal content, leading to the successful generation of a synthetic S1 tetraploid. The newly created S1 tetraploid showed morphological variations in its flowers, cotton bolls, and fibres and was partially resistant to *Verticillium dahliae* Kleb . The S1 tetraploid was hybridised with upland cotton to produce two types of cotton with different characteristics. This newly synthesised tetraploid cotton species is a valuable resource for cotton resistance breeding and for the enrichment of cotton genetic resources. The results are informative, but there are some major concerns need to be addressed.

1. The description of phenotypic morphological analysis of the diploid F_1 hybrid and the synthetic S_1 tetraploid is poorly organized. The author's investigation of morphological in materials and methods has not been described in detail. Even with reference to the methods of previous reference, no specific survey data and criteria are given. In the survey results, the key traits such as the germinating stage and the flowering stage are unclear. Moreover, wild cotton is mostly perennial wild material, and its germinating stage and flowering stage belongs to agronomic traits. It is not suitable for investigation and comparative analysis of wild materials.

__Answer：We have added the key traits such as germination and flowering of new germplasm cotton.The F_1 hybrid were sown in a pot and all germinated after 5 days. After the first true leaves were level, the seedlings were transplanted to the field. Floral buds appeared after 43-45 days and flowering occurred after 68-70 days. The S_1 were sown in a pot and all germinated after 5 days. After the first true leaves were level, the seedlings were transplanted to the field. Floral buds appeared after 45-50 days and flowering occurred after 70-75 days. Boll opening occurred after 107-115 days.

The new cotton germplasm was created by using wild cotton through distant hybridization and is the descendant of wild cotton.When investigating and analyzing it, it involves genetic problems.To explain the genetic problems of new germplasm, the source needs to be traced back. Therefore, it is necessary to list wild cotton parents as a reference in the study in order to analyze and explain.The reviewer mentioned that wild cotton is mostly perennial wild material, this statement is correct. In fact, all cotton is a perennial plant in tropical regions, including wild cotton and cultivated cotton (such as *G. hirsutum* L. *G. barbadense* L.*G. herbaceum* L.*G. arboreum* L.). The morphological traits of various cotton species grown in the same environment difference.

2. Statistical analysis of leaf shape, plant height, boll shape and other traits is not appropriate. Because the leaf shape will change according to the different growth stages of the plant, the plant height will vary greatly due to changes in the growth environment, and the boll shape will also vary in different positions of the plant. The number of bolls per plant is also strongly related to nutritional status and planting density, so the author's description of these traits seems meaningless.

__Answer：Plant morphology is a characteristic expression of a species, mainly including plant morphology traits such as plant type, stem, leaf, flower and cotton boll.Regarding the color of the floral organs of the plant, the presence or absence of the floral basal spot, the characteristics of the bracts, the amount of plant hairs, and the boll fertility is a trait that can be stably inherited and does not change with the external environment.In a limited time, these descriptions of the new germplasm we created, please dear editors and review experts to understand.We describe morphology and only want to show the new cotton germplasm as much as possible. If we talk about its significance, the birth event of new cotton germplasm is meaningful in itself.

3. When statistically analysis the differences in phenotypic traits, it is necessary to distinguish between qualitative and quantitative traits. For F_1, in the case of a limited single plant, it cannot be compared with a tetraploid with a larger number of offspring. As for the results of the investigation against Verticillium wilt, as it is a quantitative

trait, it is recommended to compare and analyze the resistance differences of different isolated plants of S_1.

__Answer：Due to the low number of diploid F_1 hybrids and tetraploid S_1 plants, these two materials cannot be used for cotton Verticillium wilt resistance experiments.In fact, in this study, we used the descendant material S_2 of S_1 to carry out disease resistance identification of seedlings in indoor nutrition bowls（Fig.5）, because S_2 material is not only a large number, but also an ideal separation group with rich morphological diversity .In the next step, we will plant S_2 in a specific field nursery, and analyze and compare the differences in disease resistance of different S_2 isolated plants.

4. For the identification and analysis of F_1 true or false hybrids, when doing cytological analysis, the chromosomes of both parents and the chromosome of F_1 should be observed.

__Answer： We have shown the cytological observations of both parents, F_1 and S_1 in the article.

5. When conducting a flow cytometer test, both parents, F_1, S_1, and the TM-1 materials, should be tested and investigated to increase the scientificity and credibility of the test.

__Answer： We have shown the results of flow cytometer test of female parent, F_1, S_1 and TM-1 in this article.

6. At present, according to the taxonomic standards of the cotton genus, it has been recognized that Gossypium nelsonii Fryxell has been classified as the G3 species. So the newly synthesized double cotton germplasm should be A1A1G3G3.

__Answer： We have changed to $A_1A_1G_3G_3$ in the article.

7. According to the cotton genus classification and distribution research, 53-54 cotton species have been discovered and named, instead of 55.

__Answer： We have changed to 54 kinds in the article.

Reviewer #2:

The manuscript has potential for publication, i the authors have achieved good results, the discussion needs to be improved, with mention to other works, in the discussion it is necessary to mention other results found in the difference of ploidy, the use of colchicine, as well as resistance to *Verticillium dahliae*, which is one of the main objectives, however, the work has all merit for publication.

__Answer：We have added relevant content to the discussion of the article. The tetraploid S_1 is a new germplasm obtained by chromosome doubling of the diploid F_1 hybrid and is a polyploidy event in cotton. This not only restores fertility to sterile diploid F_1 hybrids and the tetraploid S_1 generation obtained showed segregation, which increases the phenotypic mutations in the species, enabling breeders to select for required traits. After the polyploid was generated, its stress resistance not only did not decrease but biotic stress resistance was maintained. For example, the preliminary examination showed that the disease resistance of the tetraploid S_2 was 1 grade better than the female parent (*G. herbaceum*) and the same level as the male parent (*G. nelsonii*), and its disease resistance inheritance was closer to the male parent. In addition, the fiber quality of the tetraploid S_2 exceeded that of both parents. From this we can see that diploid F_1 hybrid polyploidy has important significance for cotton evolution, as the genome structure changed and the species genetic diversity enriched as well as the species adaptability and fiber quality were improved.

Reviewer #3:

WHAT THE AUTHORS NEED TO DO FOR IMPROVE THEIR PAPER• I THEORIZED THAT YOU MUST FOLLOW A LANGUGE EDITING FIRSTLY AND AFTERWARDS WE COULD CAPABLE TO FOLLOW DETAILED CORRECTIONS AND SUGGESTIONS.• I AM SORRY BUT THERE MAINY TERMS AND SENTENCES THAT MUST BE REWRITE USING BETTER TERMINOLOGY.ALL THE SENTENCES, TERMS AND WORDS UNDERLINED INTO THE TEXT BY YELLOW COLOUR SHOULD BE CHANGED.• PLEASE FOLLOW A LANGUAGE EDITIND FROM A PERSON WHO HAS ENGLISH AS NATIVE LANGUAGE BECAUSE YOU LOOSE MUCH OF THE VALUE OF

YOUR WORK .

PLEASE SEE CAREFULLY OUR ADVICES INTO THE FILE COMMENTS & SUGGESTIONS (1)

__Answer：We have made changes to the questions raised by the review experts.
