# Supplementary material for: Morphological description of a novel synthetic allotetraploid(A1A1G3G3) of Gossypium herbaceum L.and G.nelsonii Fryx. suitable for disease-resistant breeding applications
Source: PLoS One. 2020 Dec 3;15(12):e0242620. doi: 10.1371/journal.pone.0242620 (PMC7714114; doi:10.1371/journal.pone.0242620)
Supplement: Attachment — Submitted filename: PONE-D-20-02970_R3.pdf [file pone.0242620.s009.pdf]

PLOS ONE

Morphological description of a novel synthetic allotetraploid (A1A1G3G3) of Gossypium herbaceum L. and G. nelsonii Fryx. suitable for disease-resistant breeding applications

--Manuscript Draft--

**Manuscript Number:**	PONE-D-20-02970R2
**Article Type:**	Research Article
**Full Title:**	Morphological description of a novel synthetic allotetraploid (A1A1G3G3) of Gossypium herbaceum L. and G. nelsonii Fryx. suitable for disease-resistant breeding applications
**Short Title:**	Morphological description of a novel synthetic allotetraploid (A1A1G3G3) suitable for breeding applications
**Corresponding Author:**	Di Chen<br>Chinese Academy of Tropical Agricultural Sciences<br>Haikou, CHINA
**Keywords:**	genetic evolution, Gossypium, interspecific hybridization, synthetic allotetraploid
**Abstract:**	Wild varieties of Gossypium are a source of traits for improving commercial cultivars. Previous reports show that Gossypium herbaceum L. and Gossypium nelsonii Fryx. have better disease resistance characteristics than commercial cotton varieties. However, chromosome ploidy and biological isolation make it difficult to hybridize diploid species with the tetraploid Gossypium hirsutum . We developed a new allotetraploid cotton species (A1A1G3G3) using a process of distant hybridization within wild cotton species to create new germplasms. G. herbaceum (♀) and G. nelsonii (♂) were used for interspecific hybridizations to obtain F1 generation. The apical regions of the F1 plants were treated with colchicine to induce chromosome doubling and new interspecific hybrid F1 and tetraploid S1 populations were confirmed as true hybrids via morphological analysis, molecular marker analysis, flow cytometry, and cytological identification. The S1 population was crossed with a TM-1 line and fertile hybrid offspring were obtained. The S2 descendants of S1 demonstrated disease resistance potential. The results show that the S1 line can be the parent material for hybridization with G. hirsutum L. to produce pathogen-resistant cotton hybrids. The new S1 allotetraploid contributes to the enrichment of Gossypium germplasm resources and is expected to be valuable in polyploidy evolutionary studies.
**Order of Authors:**	Xiaomin Yin
	Rulin Zhan
	Yingdui He
	Shun Song
	Lixia Wang
	Yu Ge
	Di Chen
**Response to Reviewers:**	The concerns raised by the reviewers have been addressed in our revised manuscript. We carefully checked the language of the manuscript according to the comments of Reviewer 3 and the sticky notes in the pdf file to ensure a high level of readability in English. Please see below for our point-by-point response.
**Additional Information:**	
**Question**	**Response**
**Financial Disclosure**<br><br>Enter a financial disclosure statement that	YES - Specify the role(s) played.This research work was financially supported by the National Natural Science Foundation of China (No. 31704178) and Central Public-interest Scientific institution Basal Research Fund for Chinese Academy of Tropical

Powered by Editorial Manager® and ProduXion Manager® from Aries Systems Corporation

describes the sources of funding for the work included in this submission. Review the submission guidelines for detailed requirements. View published research articles from *PLOS ONE* for specific examples.<br><br>This statement is required for submission and **will appear in the published article** if the submission is accepted. Please make sure it is accurate.<br><br>**Unfunded studies**<br>Enter: *The author(s) received no specific funding for this work.*<br><br>**Funded studies**<br>Enter a statement with the following details:<br>• Initials of the authors who received each award<br>• Grant numbers awarded to each author<br>• The full name of each funder<br>• URL of each funder website<br>• Did the sponsors or funders play any role in the study design, data collection and analysis, decision to publish, or preparation of the manuscript?<br>• **NO** - Include this sentence at the end of your statement: *The funders had no role in study design, data collection and analysis, decision to publish, or preparation of the manuscript.*<br>• **YES** - Specify the role(s) played.<br><br>* typeset	Agricultural Sciences(No.1630092017004).
**Competing Interests**<br><br>Use the instructions below to enter a competing interest statement for this submission. On behalf of all authors, disclose any competing interests that could be perceived to bias this work—acknowledging all financial support and any other relevant financial or non-financial competing interests.<br><br>This statement **will appear in the published article** if the submission is accepted. Please make sure it is accurate. View published research articles from *PLOS ONE* for specific examples.	The authors have declared that no competing interests exist.

Powered by Editorial Manager® and ProduXion Manager® from Aries Systems Corporation

**NO authors have competing interests**<br><br>Enter: *The authors have declared that no competing interests exist.*<br><br>**Authors with competing interests**<br><br>Enter competing interest details beginning with this statement:<br><br>*I have read the journal's policy and the authors of this manuscript have the following competing interests: [insert competing interests here]*<br><br>* typeset	
**Ethics Statement**<br><br>Enter an ethics statement for this submission. This statement is required if the study involved:<br><br>• Human participants<br>• Human specimens or tissue<br>• Vertebrate animals or cephalopods<br>• Vertebrate embryos or tissues<br>• Field research<br><br>Write "N/A" if the submission does not require an ethics statement.<br><br>General guidance is provided below. Consult the submission guidelines for detailed instructions. **Make sure that all information entered here is included in the Methods section of the manuscript.**	No permits were required for the described study, which complied with all relevant regulations.

Powered by Editorial Manager® and ProduXion Manager® from Aries Systems Corporation

**Format for specific study types**<br><br>**Human Subject Research (involving human participants and/or tissue)**<br>• Give the name of the institutional review board or ethics committee that approved the study<br>• Include the approval number and/or a statement indicating approval of this research<br>• Indicate the form of consent obtained (written/oral) or the reason that consent was not obtained (e.g. the data were analyzed anonymously)<br><br>**Animal Research (involving vertebrate animals, embryos or tissues)**<br>• Provide the name of the Institutional Animal Care and Use Committee (IACUC) or other relevant ethics board that reviewed the study protocol, and indicate whether they approved this research or granted a formal waiver of ethical approval<br>• Include an approval number if one was obtained<br>• If the study involved *non-human primates*, add *additional details* about animal welfare and steps taken to ameliorate suffering<br>• If anesthesia, euthanasia, or any kind of animal sacrifice is part of the study, include briefly which substances and/or methods were applied<br><br>**Field Research**<br><br>Include the following details if this study involves the collection of plant, animal, or other materials from a natural setting:<br>• Field permit number<br>• Name of the institution or relevant body that granted permission	
**Data Availability**<br><br>Authors are required to make all data underlying the findings described fully available, without restriction, and from the time of publication. PLOS allows rare exceptions to address legal and ethical concerns. See the PLOS Data Policy and FAQ for detailed information.	Yes - all data are fully available without restriction

A Data Availability Statement describing where the data can be found is required at submission. Your answers to this question constitute the Data Availability Statement and **will be published in the article**, if accepted.<br><br>**Important:** Stating 'data available on request from the author' is not sufficient. If your data are only available upon request, select 'No' for the first question and explain your exceptional situation in the text box.<br><br>Do the authors confirm that all data underlying the findings described in their manuscript are fully available without restriction?	
**Describe where the data may be found in full sentences. If you are copying our sample text, replace any instances of XXX with the appropriate details.**<br><br>• If the data are **held or will be held in a public repository**, include URLs, accession numbers or DOIs. If this information will only be available after acceptance, indicate this by ticking the box below. For example: *All XXX files are available from the XXX database (accession number(s) XXX, XXX.*).<br>• If the data are all contained **within the manuscript and/or Supporting Information files**, enter the following: *All relevant data are within the manuscript and its Supporting Information files.*<br>• If neither of these applies but you are able to provide **details of access elsewhere**, with or without limitations, please do so. For example:<br><br>*Data cannot be shared publicly because of [XXX]. Data are available from the XXX Institutional Data Access / Ethics Committee (contact via XXX) for researchers who meet the criteria for access to confidential data.*<br><br>*The data underlying the results presented in the study are available from (include the name of the third party*	All relevant data are within the manuscript and its Supporting Information files.

and contact information or URL).<br>• This text is appropriate if the data are owned by a third party and authors do not have permission to share the data.<br><br>* typeset	
Additional data availability information:	

Powered by Editorial Manager® and ProduXion Manager® from Aries Systems Corporation

Manuscript
Click here to access/download;Manuscript;Revised Manuscript.pdf

Morphological description of a novel synthetic allotetraploid($A_1A_1G_3G_3$) of *Gossypium herbaceum* L. and *G. nelsonii* Fryx. suitable for disease-resistant breeding applications

Xiaomin Yin[#], Rulin Zhan[#],Yingdui He, Shun Song, Lixia Wang, Yu Ge, Di Chen[*]

Haikou Experimental Station, Chinese Academy of Tropical Agricultural Sciences (CATAS), Haikou, China

*Corresponding author

E-mail: chendi209@163.com (DC)

Keywords: genetic evolution, *Gossypium*, interspecific hybridization, synthetic allotetraploid

Funding sources

This research work was financially supported by the National Natural Science Foundation of China (No. 31701478) and Central Public-interest Scientific institution Basal Research Fund for Chinese Academy of Tropical Agricultural Sciences(No.1630092017004).

Abstract

Wild varieties of *Gossypium* are a source of traits for improving commercial cultivars. Previous reports show that *Gossypium herbaceum* L. and *Gossypium nelsonii* Fryx. have better disease resistance characteristics than commercial cotton varieties. However, chromosome ploidy and biological isolation make it difficult to hybridize diploid species with the tetraploid *Gossypium hirsutum* L. We developed a new allotetraploid cotton species ($A_1A_1G_3G_3$) using a process of distant hybridization within wild cotton species to create new germplasms. *G. herbaceum* (♀) and *G. nelsonii* (♂) were used for interspecific hybridizations to obtain the F_1 generation. The apical regions of the F_1 plants were treated with colchicine to induce chromosome doubling and new interspecific hybrid F_1 and tetraploid S_1 populations were confirmed as true hybrids via morphological analysis, molecular marker analysis, flow cytometry, and cytological identification. The S_1 population was crossed with a TM-1 line and fertile hybrid offspring were obtained. ~~The S_2 descendants of S_1~~ demonstrated disease resistance potential. The results show that the S_1 line can be the parent material for hybridization with *G. hirsutum* L. to produce pathogen-resistant cotton hybrids. The new S_1 allotetraploid contributes to the enrichment of *Gossypium* germplasm resources and is expected to be valuable in polyploidy evolutionary studies.

Introduction

The *Gossypium* genus includes 54 species, of which 50 are wild cotton species and four are cultivated cotton species made up of two diploids and two tetraploids [1, 2]. Approximately 90 % of commercially produced cotton is cultivated from *G.*

hirsutum L. However, as the high-yielding quality of cultivated species continuously increases, abiotic and biotic resistance usually does not increase and can even be weakened [3,4]. To increase the resistance in *G. hirsutum* L., the existing genetic basis of cultivated cotton should be expanded to adapt to biotic and abiotic stresses. It is generally believed that wild cotton species contain a large treasure trove of traits that could be used to improve cultivated species [5]. The wild cotton species are widely distributed in various ecological conditions in the Americas, Oceania, Africa, and Asia. These species have undergone long-term natural selection, and are resistant to many types of adverse factors, such as disease, insects, drought, heat, and salinity. At the same time, there are many mutant varieties, all of which contribute to cotton's rich genetic foundation [6,7].

G. herbaceum L. is a diploid cotton species cultivated in Western China and Russia. This species is highly resistant to drought, cotton leaf curl virus and sap-feeding insects such as leafhoppers, whiteflies, thrips, and aphids [8, 9]. *G. nelsonii* Fryx. is a wild diploid cotton species that originated in Australia and has many economically valuable characteristics, such as delayed pigment gland formation. This is beneficial for producing seeds with low gossypol levels. In addition, it is resistant to blight, Verticillium wilt, aphids, and mites; and is tolerant to abiotic stress conditions such as high temperatures and drought. Furthermore, its brown, straight, extensible, and high-strength fibers are valuable agronomical traits. If these characteristics can be transferred to tetraploid *G. hirsutum* L. (2n = 4x = 52, AADD), then the resulting species will be extremely valuable.

Owing to the biological isolation between the species, it is difficult to directly hybridize diploid cotton species with tetraploid *G. hirsutum*. We are proposing a new pathway for transferring genes from two diploid cotton species into tetraploid *G.*

hirsutum. Specifically, two different diploid species undergo interspecific hybridization so that they can recognize and overcome their biological segregations to form the diploid hybrid F_1. Following that, artificial ploidy doubling of the apical regions in F_1 hybrids is carried out to obtain a tetraploid cotton species that has the same chromosome number as *G. hirsutum* L. The new tetraploid cotton is then hybridized with the tetraploid *G. hirsutum* to achieve efficient gene transfer. This process shortens the time needed to introduce essential genes from two species and is currently the most direct and easiest method that has the potential to be utilized with wild cotton. In the last few decades, studies on interspecific hybrids have been reported, including *G. arboretum* × *G. raimondii*[10], *G. hirsutum* × *G. sturtianum* [11], *G. arboreum* × *G. bickii* [12], *G. hirsutum* × *G. klotzschianum* [13], *G. hirsutum* × *G. trilobum* [14], *G. arboreum* × *G. anomalum* [15], *G. hirsutum* × *G. anomalum* [16], *G. herbaceum* × *G. australe* [17], *G.hirsutum* × *G.australe* [18], *G.hirsutum* and *G. arboretum* [19], and *G. capitis-viridis* × (*G.hirsutum* × *G.australe*)^2 [20]. To the best of our knowledge, there has been no report of using *G. herbaceum* and *G. nelsonii* to successfully synthesize an allotetraploid ($A_1A_1G_3G_3$).

In this work, we applied homoploid hybridization to provide a pathway to simultaneously transfer genes from these two diploid cotton species into *G. hirsutum* using *G. herbaceum* as the female parent and *G. nelsonii* as the male parent to successfully generate the interspecific hybrid F_1 ($2n = A_1G_3 = 26$). Colchicine treatments were used to double the chromosome numberand form a new tetraploid cotton genotype (code: S_1) ($2n = 4x = 52$, $A_1A_1G_3G_3$). The birth of the new S_1 cotton tetraploid not only enriches the genetic foundation of cotton but also provides a valuable intermediate material for cotton hybridization and breeding. This is a

successful example of using wild cotton species for distant hybridization and has important significance for promoting scientific research on cotton genetics.

Materials and methods

Plant material

The diploid *G. herbaceum* cotton species that has been cultivated in China is also known as Hongxingcaomian (2n = 2x = 26, A_1A_1), and is a line that has not been subjected to inbreeding. *G. nelsonii* is a diploid cotton species from Australia (2n = 2x = 26, G_3G_3), and the two *G. hirsutum* lines , cv. *Jimian11* (a commercial variety from China) and the control variety TM-1 (a prototype for molecular mapping in cotton) were used in our experiments. The lines mentioned above were obtained from The China National Wild Cotton Nursery in Sanya, China. The *Verticillium dahliae* strain Dahlia Vd080, used for disease resistance identification, was provided by the Institute of Cotton Research, Chinese Academy of Agricultural Sciences.

Interspecific hybridization

Interspecific hybridization of the cotton was carried out in Sanya (18°25′34″N, 109°28′25″E), Hainan, China in 2015 [21]. When the two parental species flowered at the same time, *G. herbaceum* plants were selected as the female parents. One day before flowering, all stamens in the flowers from the female parents were completely removed. Wax tubes were used to cover the stigmas and non-woven bags were used to cover all of the floral buds. The afternoon before flowering, cotton thread was used to tie the apices of the petals in the flowers of the male parents (*G. nelsonii*), and non-woven bags were used to cover the floral buds entirely. From 9 am to 12 pm on the

day of flowering, pollen from the male parents was used to pollinate the stigmas of the female parents for hybridization. After pollination was completed, wax tubes were used to cover the stigmas, non-woven bags were used to cover the entire floral buds, and labels were hung. After the cotton bolls had matured, the seeds were removed and sown in nutrient pots containing sterile soil. After one or two true leaves had sprouted, the plants were transplanted into fields and normal fertilizer and water management was carried out. The morphological characteristics of the plants were observed, including the type of plant, stems, leaves, flowers and bolls. The morphological characteristics of each plant part at different biological stages were photographed using a digital camera, and the photographs were edited by using Photoshop software.

SSR molecular marker analysis in F_1 hybrids

We randomly selected 568 pairs of SSRs based on our cotton genetic map [22] to screen for parental polymorphisms. The polymorphic primers obtained were used to validate the authenticity of the interspecific F_1 hybrids. These SSR primer sequences can be found at http://www.cottonmarker.org. A programmable gradient thermal cycler (Bio-Rad T100) was used for SSR-PCR amplification[23]. Electrophoresis and silver staining of the PCR products were carried out according to previously reported methods [24]. The bands generated by electrophoresis were photographed and edited by Photoshop software.

Chromosome duplication in the F_1 interspecific hybrid

In 2016–2018, the F_1 hybrids that were validated by the SSR molecular markers were grafted. The temperature was kept at 25 ℃ and the apical growth points of the F_1 hybrids were immersed in 0.30 % (w/v) colchicine for 6 h. After immersion, they

were placed in pots in the greenhouse that were not exposed to light and incubated until new growth points or floral buds sprouted. When the length of the new meristematic apices reached 5 cm, they were removed and grafted for storage and further validation of the chromosome doubling results [20]. Newly sprouted floral buds could grow until the afternoon before flowering. Then, a cotton thread was used to tie the tip of the petal for self-pollination and non-woven bags were used to cover the entire floral bud to prevent outcrossing. After the cotton bolls matured and underwent dehiscence, the seeds were removed and sown in nutrient pots. After one or two true leaves sprouted, the plants were transplanted into fields. The morphological characteristics of the plants were observed, including the plant shape, stems, leaves, flowers, and bolls. The resulting tetraploid plant was named S_1, and chromosome counting and flowcytometric analysis were performed for validation. The morphological characteristics of each plant part were photographed using a camera, and the photographs were edited by using Photoshop software.

Ploidy analysis

In order to detect whether the S_1 chromosome had been doubled, flow cytometry was used to examine the differences in the chromosome numbers between the F_1 hybrid and S_1, simultaneously diploid *G. herbaceum* L. and the tetraploid TM-1 lines were used as controls. The operational steps were divided into preparation of the sample cell suspensions, specific staining of DNA, and loading. Specific methods were carried out according to the description of Galbraith [25]. A Sysmex-Partec CyFlow Cube8 flow cytometer was used to determine the nuclear DNA content. CellQuest Pro software was used to analyze the results. The experimental data are displayed in a

single-parameter histogram. The ordinate represents the number of nuclei (count), and the abscissa represents the relative intensity of the fluorescent channel.

Karyotype analysis

Microscopic observations of the number of root tip chromosomes in the F_1 hybrid and its resultant tetraploid S_1 progenies were carried out to observe the differences in the chromosome number between the two genotypes, and *G. herbaceum* and *G. nelsonii* were used as controls. Finally, karyotype analysis of the F_1 hybrid was performed for determination of chromosome doubling. Root tip fixation, preservation, dissection, squash preparation, and microscopy were carried out according to previously reported methods [26]. A Nikon Eclipse Ni-E biological microscope and camera system was used to observe, count, and photograph the chromosomes in the root-tip cells. Photoshop software was used to edit the images.

Validation of disease resistance in the tetraploid S_2

S_2 descendants of S_1 were chosen as the target of disease resistance identification. The tetraploid S_2 seedlings, growing in nutrient pots, were inoculated indoors with the *Verticillium dahliae* strain (code: Dahlia Vd080 strain) for preliminary determination of disease resistance. The strain cv. *Jimian11* was used as an infection control group; *G. herbaceum* and *G. nelsonii* were used as disease resistance controls. In the experiment, there were two treatments (fungal suspension or sterile water for inoculation) performed in triplicate. Each replicate of the experiment contained 540 plants.

Inoculations of the seedlings in the nutrient pots were carried out according to a previously reported method [27]. Seeds were sown in cylindrical paper pots (height 8

cm and diameter 5 cm) with no bottom and filled with sterile medium. When the first true leaf had grown until it was level, 10^7 conidia/ml of suspension was directly injected into the 8-cm-dimeter plastic tray. Only sterile water was used for inoculation in the control group. The paper pot and seedlings were placed upright in the trays so that the root systems adsorbed the suspension liquid. Examination of the disease symptoms was carried out according to previously reported methods [19]. Changes in leaf color and degree of wilting were used as a basis for preliminary observations of disease resistance in the experimental materials.

Hybridization between the S_1 interspecific hybrid and *G. hirsutum* (TM-1 cultivar)

In the winter of 2017, hybridization was carried out in Sanya, Hainan province, China, using the tetraploid S_1 as the male parent (♂) and the TM-1 line (♀) as the female parent. The hybridization method used was similar to the previously described for *G. herbaceum* (♀) and *G. nelsonii* (♂) [21], and the boll-forming rate and effective boll-forming rate of hybrids were determined. After the hybridized cotton bolls matured and underwent dehiscence, seeds were removed and sowed in nutrient pots containing sterile soil. After one or two true leaves sprouted, the plants were transplanted into fields. The morphological characteristics of the plants were observed, including plant shape, stems, leaves, flowers and bolls. Based on the morphological characteristics, the authenticity of the hybrids was preliminarily assessed and the fertility of the tetraploid S_1 hybrids was examine[illegible] The morphological characteristics of each plant part were photographed, and the images were edited with Photoshop software.

Image acquisition and editing

A Canon EOS 70D digital camera with an RF 35 mm F1.8 Macro Lens was used to photograph the morphology of plant parts and electrophoresis bands of the PCR products. The images were edited with Adobe Photoshop CS5 software.

Results

F_1 hybrid generation and trait observations

In 2016, hybridization was conducted using *G. herbaceum* as the female parent and *G. nelsonii* as the male parent. A total of 235 flowers were pollinated, 205 of which formed bolls. However, most bolls did not form seeds and only three effective bolls were finally obtained; each containing one seed. In 2017, the three seeds were sown in a pot and all germinated after 5 days. After the first true leaves were level, the seedlings were transplanted to the field. Floral buds appeared after 43–45 days and flowering occurred after 68–70 days. In Sanya, China, the three F_1 plants showed exuberant growth, were tall, produced large numbers of flowers throughout the year, and could produce pollen. However, the pollen produced was non-viable and did not produce bolls. The samples have been preserved, but are still infertile. This highlights the difficulty in obtaining F_1 hybrids from interspecific hybridizations between *G. herbaceum* L. and *G. nelsonii* Fryx..

The morphological traits of the F_1 plants showed classical hybrid characteristics(Figs. 1) according to the 14–item investigation data analysis (Table 1). Morphological comparison with parental plants showed that two items were greater than the parents: plant height (Fig. 1a–c) and corolla color (Fig. 1e–g). Five items were intermediate between the parents: leaf shape and color, number of lobes, bract shape, calyx teeth shape, and number of bract teeth (Fig. 1e–g). Two items were

similar to the female parent, anther and pollen color (Fig. 1e, 1g). Five items were similar to the male parent: stem and leaf trichomes, flowering habit, bract extension, flower opening, and extrafloral nectary (Fig. 1f, 1g).

Table 1. Comparison of morphological traits between *G. herbaceum* L. × *G. nelsonii* Fryx. F_1 hybrid and its parental genotypes

Character	*G. herbaceum* L.	*G. nelsonii* Fryx.	F_1
Plant height (cm)	80	200	300
Stem/branch characteristics	Less trichomes	More trichomes	More trichomes
Shape and color of leaf	Broad-leaved, Green	Ovoid, gray green	Broad-leaved, ovoid, subcircular, Green
No. of lobes	3–5	0–1	0–3
Bract size (length × width, cm)	1.1×1.3	1.2×0.2	1.1×0.5
Bract shape, No. of teeth	Heart-shaped, 4–6	Lanceolate, 1	Sword-shaped, 1–3
Bract jointing	Joint	No joint	No joint
Floral nectary	Intra-floral	Extrafloral	Extrafloral
Calyx	Cup-shaped	Wavy	Serration
Bract tooth	5–9	1	4–6
Corolla shape and color	Yellow trumpet, around	Light pink, funnel-shaped,	Pink trumpet, around 2.5
Corolla length (cm)	2.1	around 3.5	
Color and size of spot	Rose	Purple	Red
Filaments colors	Pink interspersed with milky white	Rose	Light pink
Anther colors	Yellow	Rose	Light yellow
Pollen colors	Yellow	Milky white	Yellow
Stigma colors	Milky white	Light green	Milky white
Boll	Conical	Oval	/
Fiber	Spiral	Upright	/

Figure 1. Pictures of plants, leaves , flowers, corollas, basal patches, filaments, anthers, pollen, calyxes, and bracts. *G. herbaceum* (a, e), *G. nelsonii* (b, f), the F_1 (c, g), the S_1 (d, h).

SSR molecular marker determination was carried out and six pairs of parental polymorphic primers (Table 2) were obtained from the screening. These six pairs of primers could be used to amplify the complementary bands from the parents in the F_1 hybrids (Fig. 2a), of which two primer pairs (NAU1052 and NAU3093) amplified not only the bands that are characteristic of the parents but also characteristic the bands of the hybrid itself (Fig. 2a/iii, 3a/vi) , showing that the F_1 hybrids are true hybrids. Flow cytometry showed that the peak value of the control sample of *G. herbaceum* was approximately 200 channels (Fig. 2b); the peak value of TM-1 was close to 400 channels (Fig. 2c); and the peak value of the F_1 hybrid was close to 200 channels (Fig. 2d), indicating that the F_1 hybrid was a diploid. Root tip staining and microscopy observations showed that the root tip chromosome number of both the *G. herbaceum* and *G. nelsonii* control samples (Fig. 2e, 2f), and the F_1 hybrid (Fig. 2g) was also 26 pairs, indicating that the F_1 hybrid was a diploid.

Table 2. SSR polymorphism primer sequences

Primer number	Forward sequence (5′-3′)	Reverse sequence (5′-3′)
NAU1157	GAGTTTGGTTCTGGGTTGAG	GATCCTTTTCATCTCCTCCA
NAU1355	ATCTGTTTACGCCACTCTCC	CCAGCCTTTGACATTTTTCT
NAU1052	CGCAGATAAAGGATGGATTT	AGAGCTGGAGGACATAACAAA
JESPR156	GCCTTCAATCAATTCATACG	GAAGGAGAAAGCAACGAATTAG
BNL1679	AATTGAGTGATACTAGCATTTCAGC	AAAGGGATTTGCTGGCAGTA
NAU3093	GTCTTGAACCGGAACTTGAT	TCCTGTTGAACACCAAAGTG

Figure 2. Validation of F_1 and S_1 by detecting DNA polymorphism, ploidy analysis, and the number of chromosomes. (a) Microsatellite loci identified by SSR primers including (i) NAU1157, (ii) NAU1355, (iii) NAU1052, (iv)J ESPR156, (v) BNL1679, and (vi) NAU3093. The novel bands produced in F_1 and S_1 are indicated by red and blue arrows (1, *G. herbaceum*; 2, *G. nelsonii*; 3, the F_1; 4, the S_1). (b) *G. herbaceum* at 200 channels. (c) TM-1 at 400 channels, respectively. (d) F_1 and S_1 at 200 channels and 400 channels respectively. (e) 26 of *G. herbaceum*. (f) 26 of *G. nelsonii*. (g) 26 of the F_1. (h) 52 of the S_1. The root tip cells (red arrows) and the chromosomes (black arrows) stained with Carbol fuchsin solution under 40× microscope.

Tetraploid S_1 generation and trait observations

After the apical growth point of the F_1 hybrid underwent chromosome doubling, the first large red flower grew in the apex of the plant after 27 days (S1 Fig. a). The flower was full of pollen and a boll containing four seeds was formed through self-pollination (S1 Fig. b, c). In 2018, the four seeds were sown in a pot and all

germinated after 5 days. After the first true leaves were level, the seedlings were transplanted to the field. Floral buds appeared after 45-50 days and flowering occurred after 70–75 days. Boll opening occurred after 107–115 days. In Sanya, the four S_1 plants grew normally, with medium plant height and flowering that occurred throughout the year. The pollen formed was viable and many bolls were formed. The samples that were preserved were fertile. Chromosome doubling of the F_1 hybrids restored their fertility. We hypothesized that the four S_1 plants were all tetraploid hybrids and were assigned the code S_1 ($2n = 4x = 52$, $A_1A_1G_3G_3$). The differences in the morphological characteristics between the four S_1 plants were not large. Most plants developed large red flowers, with radiating purple patches, and significant separation was not observed. The leaves were large and tough, and the plants were tall and shrub-like (Fig. 1d). The plants produced many flowers, released pollen, and produced fruit normally. The fruits formed were small bolls containing small seeds, and short, brown, high-strength fibers (Fig. 3f). In particular, the S_1 plants retained some characteristics of *G. nelsonii* Fryx. For example, at low temperatures, the buds on the lower branches were extremely small (0.50 cm length), milky white in color, did not open until wilting, and had closed pollination. In contrast, flowers in the middle and top branches were large (4.40 cm length), opened normally, and had open pollination. The red flowers contained large purple patches, the bracts were opened, and the plants contained a lot of glands, but not the seed embryo.

The differences in the morphological characteristics between the S_1 plants and F_1 plants is that S_1 plants were shorter (Tables 1, 3), had larger and thicker leaves, produced viable pollen, could form bolls, seeds, and long fibers, and had 50 bolls per plant (S2 Fig. a) after 4 months of growth in the field. The samples were still fertile after preservation. Fertility was restored after the diploid F_1 hybrids underwent

chromosome doubling. However, other S_1 traits were not different from the F_1 plants, including leaf shape and color, number of lobes, bract shape, calyx teeth shape, number of bract teeth, anther and pollen color, stem and leaf trichomes, bract extension, and extrafloral nectary(Fig. 1g, 1h).

Table 3. Comparison between S_1 and S_2 (*G. herbaceum* × *G. nelsonii*) generations based on morphological and fertility traits

Generation	Plant height (cm)	Colors of leaf	Leaf size (length×width, cm)	No. of lobes	No. of nectaries on the back of leaf blade	Corolla color	Corolla length (cm)	Color and size of spot	Anther and Filaments colors	Bract size (length×width, cm)	Bract shape, No. of teeth (n)	Flower nectary	Boll shape, No. of boll loculi	Boll size (length× width, cm)	Sterility
S_1	150.00	Dark green	5.10×5.40	0–3	1 on primary vein	Pink,	Around 2.60	Red	Yellow, Light pink	1.20×0.60	Sword-shaped, 1–3	Extrafloral	Pear-shaped, 4	Generally, 3.00×2.50	Many bolls formed
S_2	75.00–170.00	Light green, Dark green	5.70×6.20–10.90×13.60	0–3	2, 3 at the primary vein and lateral vein	Pink, Light pink, Milky white, Red flower with white border	2.40–4.40	Red, Purple, Rose, None	Yellow, Light pink, Pink interspersed with milky white	1.30×0.70–1.60×1.10	Triangle, Dentate, Ensiform, 2–4	None, extrafloral, intra-floral	Pear-shaped, sunken navel, oval, irregular round boll, 3–4	2.70×2.30–3.20×2.60	Only 32 out of 145 plants had 1–7 bolls while no bolls were found in other plants

We determined that the S_1 plants were similar to the F_1 plants as their morphological traits showed classical hybrid characteristics (Fig. 1). According to the 25-item investigation data analysis (Table 3), 10 items(Fig. 1e–h) were intermediate between the parents including leaf shape and color, number of lobes, bract shape, calyx teeth shape, number of bract teeth, corolla size, number of anthers, boll diameter (Fig. 3a–c), fiber color, and fiber length. Two items were greater than the parents, including the number of boll loculi and fiber tensile strength (Fig. 3d–3f). Two items were similar to the female parent, including anther and pollen color (Fig. 1e, 1h), and six items were similar to the male parent including stem and leaf trichomes (Fig. 1b, 1d), flowering habit, bract extension, bract size (Fig. 1f, 1h), flower opening, and extrafloral nectary. In addition, maternal traits expressed as dominant for the anthers, stamens, and stigmata's characteristics (Fig. 1e, 1h), whereas paternal traits were expressed in the stem and leaf trichomes, boll shape, and trichomes (Fig. 3e, 3f).

Figure 3. Pictures of cotton bolls and fibers. (a) *G. herbaceum* had conical, smooth green and red bolls. (b) *G. nelsonii* had oval, green cotton bolls. (c) Green pear-shaped boll of S_1. (d) *G. herbaceum* had 3-chambered bolls with gray-white spiral cotton fibers. (e) *G. nelsonii* had 3-chambered bolls with dark brown upright fibers. (f) The S_1 had 3-chambered bolls with brown fibers.

SSR molecular marker determination showed that the six pairs of parental polymorphic primers(Fig. 2a) amplified the complementary bands of the parents, as well as the characteristic bands of the hybrid itself (Fig. 2a/iii, 3a/vi). This indicated that S_1 is also a true hybrid. At the same time, this shows that the external genes were not introduced during the chromosome doubling of the F_1 to obtain the S_1. Flow

cytometry showed that the peak value of the control sample of *G. herbaceum* was close to 200 channels (Fig. 2b), the peak value of the TM-1 was close to 400 channels (Fig. 2c), and the peak value of the S_1 hybrid was close to 400 channels (Fig. 2d), showing that the S_1 hybrid was a tetraploid. Root tip staining and microscopy observations showed that the root tip chromosome number of the *G. herbaceum* and *G. nelsonii* control samples was 26 pairs (Fig. 2e, 2f), whereas the root tip chromosome number of the S_1 hybrid was 52 pairs (Fig. 2h). This proved that the S_1 hybrid was a tetraploid.

Examination of tetraploid S_2 segregation

The S_2 plants were completely different from the S_1 plants, and showed abnormal and intense segregation. Due to the complex genetic basis of interspecific hybrids, the segregation of F_2 populations was diverse, from male parent to female parent types, including a series of intermediate types, as well as new types of plants that are not available in parents. In the F_2 generation population, 10 representative plants were selected as observation objects, and morphological indexes were compared in the S_2 descendants of S_1 with their parental plants (Table 5). For example, the shortest plant was 75.00 cm tall, which was shorter than the female parent *G. herbaceum* L., whereas most shrubs were tall and thick with the tallest plant being 170.00 cm. Corolla color began to segregate as plants came to have not only red corollas, but also pink, red with white borders, and milky white flowers (Fig. 4a–4h) . However, yellow flowers were not observed on the female parent (Fig. 1e). The leaves were generally large, with the longest leaf being 9.90 cm and the widest leaves being 11.00 cm. Nectaries at the back of the leaves were also unique, as they were different from normal leaves that had one nectary at the base of the primary vein, but both primary

and lateral veins contained nectaries. Some leaves had two, whereas other leaves had three nectaries (Table 3), which are traits rarely observed in the *Gossypium* genus.

Figure 4. Flowers of the tetraploid S_2. (a) Pink, cylindrical flower with pink corolla, (b) funnel-shaped flower with light pink corolla, (c) funnel-shaped flower with white and pink alternating corolla, (d) white, closed flower, (e) bell-shaped flower with red corolla and a clear rose-colored spot on the petal base, (f) semi-closed flower with pink corolla, (g) bell-shaped flower with pale pink corolla. (h) small flower with white corolla and no spot on the petal base.

Observations also showed that the S_2 morphology was associated with fertility. Plants that resembled a pagoda like the female parent produced more bolls and seeds, with 5–10 seeds per boll. In contrast, plants that resembled the male parent were inclined to have a higher ratio of sterility and few fertile plants that produced fewer bolls, with each boll containing only a few seeds (1–5).

Fertility was also found to be highly segregated, with some weaker plants growing slowly and flowering later with fewer flowers. They were still normally able to disperse pollen and fruit, even though the fruits were small and not upright. Other plants grew more readily, flowering earlier with more flowers and fruits, and the fruits were large and upright. Most fibers produced were brown, but some bolls contained fibers that were both brown and light green (S2 Fig. b, d).

The above results indicate that the S_2 generation underwent intense segregation in both morphological characteristics and fertility. This phenomenon exists in varying degrees in other interspecific hybrids that we generated and is common in segregated generations. However, it was particularly prominent in the S_2 generation of this hybrid.

Table 3 compares the observation data of the S_2 and S_1 plants. It should be mentioned that the S_2 was used as an experimental material because it was a population with genetic segregation and with a rich genetic diversity. It is also an ideal population for the screening of disease-resistant varieties using conventional hybridization and breeding methods.

Table 4. Comparison of quantifiable parameters in the S_2 descendants of S_1 with their parental plants

Plants	Plant height (cm)	Internode of stem (cm)	Vegetative branches	Fruiting branches	Leaf size (length×width, cm)	Bract size (length×width, cm)	Boll size (length×width, cm)	Corolla length (cm)	Stigma length (cm)	Length of fiber (mm)
S_2-1	156.68	5.18	5	13	8.26×9.76	1.58×1.10	3.11×2.56	4.35	1.14	12.86
S_2-2	170.00	5.21	7	15	9.23×10.81	1.60×1.10	3.26×2.65	4.46	1.15	11.12
S_2-3	146.23	5.06	5	14	9.51×10.72	1.50×0.98	2.89×2.67	4.14	1.22	10.46
S_2-4	138.17	4.89	6	15	8.62×9.92	1.30×0.89	3.05×2.38	3.95	1.21	12.98
S_2-5	129.28	5.07	5	12	9.81×10.61	1.25×0.76	2.97×2.56	4.28	1.36	10.56
S_2-6	89.86	4.86	4	9	6.33×7.50	1.38×0.72	2.84×2.80	2.51	1.28	9.78
S_2-7	70.00	3.16	4	7	5.71×6.20	1.3×0.72	2.78×2.36	2.46	0.95	8.23
S_2-8	98.56	3.38	4	8	8.21×9.80	1.35×0.72	2.75×2.58	2.67	0.89	7.46
S_2-9	158.46	4.78	5	42	8.63×10.20	1.35×0.68	3.08×2.59	4.39	1.16	10.95
S_2-10	169.76	4.86	5	15	9.90×11.00	1.59×1.02	3.19×2.98	4.34	1.35	10.96
G.herbaceum	80.00	3.45	4	7	5.21×6.18	1.10×1.32	3.56×2.87	2.18	0.85	17.70
G.nelsonii	200.00	4.96	7	15	12.86×13.98	1.2×0.25	2.31×2.06	3.53	1.48	5.60

Determination of disease resistance in the tetraploid S_2

For each plant, there were 108 nutrient pots, and each pot contained five seedlings. After the first true leaf was level, the plants were inoculated with a fixed concentration of *Verticillium dahliae* Kleb. suspension (Fig. 5a). After 20 days the percentage of infected plants in the control group was 80 %. Results (Table 6) showed that when the inoculation was carried out indoors, the seedlings developed disease at the cotyledon stage. There were 456 plants from the susceptible strain cv. *Jimian11* that developed disease, with an incidence of 84.44 % and disease index of 44.00 (Fig. 5b). The resistant grades in S_2 descendants of S_1 were considered to be immune or highly resistant (Fig. 5c). The incidence and disease indexes of *G. nelsonii* were zero, and the resistance was classified as immune (Fig. 5d). The disease index of *G. herbaceum* was 1.68 (Fig. 5e). No disease occurred in any of the plants inoculated with sterile water. From this, it was preliminarily determined that the tetraploid S_2 was germplasm material with potential disease resistance. In future studies, we will examine and validate the disease resistance in the S_2 in specific plots in the field, to provide reliable intermediate materials for the cultivation of disease-resistant *G. hirsutum* L.

Table 5. Identification of cotton Verticillium wilt resistance in the S_2 descendants of S_1 with their parental plants.

Cultivar	Disease index	Relative resistance index	Classification
Jimian11	44.00±3.62	22.00±1.81	S
S_2-1	0.00	0.00	I
S_2-2	0.00	0.00	I
S_2-3	0.28±0.68	0.14±0.34	HR
S_2-4	0.00	0.00	I
S_2-5	0.00	0.00	I
S_2-6	0.56±0.12	0.28±0.06	HR
S_2-7	0.00	0.00	I
S_2-8	0.00	0.00	I
S_2-9	0.16±2.86	0.8±1.43	HR
S_2-10	0.00	0.00	I
G. nelsonii Fryx.	0.00	0.00	I
G. herbaceum L.	1.68±1.64	0.84±0.82	HR

Figure 5. Identification of resistance to *Verticillium dahliae* Kleb. (a) The pathogen of the conidia is indicated with the black arrow. (b) Disease-susceptible upland cotton *Jimian11*, with yellowing, necrosis, and leaf abscission indicated by red arrows. (c) The resistant S_1. (d) Resistant *G. nelsonii*. (e) Resistant *G. herbaceum*.

Description of the S_1 synthetic tetraploid hybrid based on morphological and fertility traits

From an analysis of the effective boll number and boll-forming rate (Table 6), we determined that the tetraploid S_1 showed some compatibility for hybridization with the TM-1. In 2018, the S_1 was hybridized with the TM-1 plants and 16 effective bolls

were obtained, but they were small and deformed (S3 Fig. a–d). These grew into seven three-way F_1 plants, of which only two grew normally and were fertile (Fig. 6a–6d). The remaining five plants did not grow normally; some grew to the flowering stage and growth stopped, some died, some were infertile, and some had low fertility. This is an inevitable consequence of three-way species crossing between *G. hirsutum* L. × (*G. herbaceum* L. × *G. nelsonii* Fryx. S_1). Hence, the five three-way F_1 hybrids cannot be used for breeding.

Table 6. Hybridization characteristics of TM-1 × (*G. herbaceum* × *G. nelsonii* S_1)

No. of flowers pollinated	Effective boll number	Boll-forming rate (%)	No. of normal embryos	No. of three-way F_1 plants	No. of three-way F_1 low-fertility plants
278	16	5.75	9	7	2

The plants described above, that grew normally and were fertile, were transplanted to fields and named F_1-1 and F_1-2 (Fig. 6c, 6d). Field morphological observations found that the F_1-1 plant retained significant wild cotton characteristics, which were mostly paternal traits(Fig. 6f, 6g). The morphology of the F_1-2 was an intermediate type, having the most important traits from *G. hirsutum* L. (Fig. 6e, 6h). The significant differences between the two plants were the corolla color, presence of spots, bract characteristics, trichomes, and boll fertility. The F_1-1 plant had a pink-colored corolla and red spots, bracts that were separate and not jointed (Fig. 6g), and the stems, branches, leaves, and bolls had a large number of trichomes. The boll number was low, and the bolls were deformed (Fig. 6k). Furthermore, the embryos tested from the seeds were very small and infertile. The F_1-2 plant had a milky white corolla; no spots were present, and the bracts were jointed (Fig. 9h). The surfaces of the stems, branches, leaves, and bolls were smooth and there were no trichomes. The

bolls developed into a funnel shape and the seed embryos were large and fertile (Fig. 6l).

Figure 6. The plants, leaves, flowers, corollas, basal patches, filaments, anthers, pollen, calyxes, bracts, and bolls about TM-1, S_1 and their offprings. TM-1(a, e), S_1(b, f), F_1-1(c, g), F_1-2(d, h). (i) normal conical boll of cotton TM-1, (j) green pear-shaped boll of S_1, (k) downy green boll of F_1-1 that was deformed, indicated by the red arrow, and (l) normal conical boll of F_1-2.

The S_1 had some potential for breeding applications, but there was severe segregation in its progeny and its fertility was decreased. Notably, a limitation of our study is that we have only preliminarily assessed the authenticity of the F_1 generation obtained from the crosses between the TM-1 and S_1 through morphological characterization. Further studies using cytology and molecular genetics techniques are required to confirm that they are true hybrids.

Discussion

Wild cotton varieties have undergone long-term natural selection to accumulate rich genetic diversity conferring adaptability to adverse conditions and resistance to environmental onslaughts and diseases. They provide a great treasury of traits that could be utilized to improve commercial cultivars [5]. Despite continuous improvements in yield and fiber quality of commercial cotton cultivars, disease resistance has weakened. Resistance breeding programs are now being conducted to transfer the disease resistance genes from wild cotton to cultivated cotton, and to re-diversify the genetic makeup of cultivated cotton.

The superior quality of cotton fiber and the disease resistance of wild cotton have been successfully transferred to cultivated cotton [28, 29]; However, diploid wild cotton and tetraploid cultivated cotton are biologically isolated. They are often genetically incompatible, forming a barrier to the full utilization of wild diploid cotton varieties [30–32]. We have already obtained an interspecific hybrid F_1 of *G. herbaceum* and *G. raimondii*. The F_1 plants are sterile in the field, leading to various methods being attempted to double their chromosome but without success [21]. In the present study, we successfully obtained an infertile interspecific F_1 hybrid and managed to overcome the genetic incompatibility barriers between two distantly related species. Furthermore, we doubled the chromosomal content of this F_1 hybrid through colchicine immersion, overcoming the considerable challenges of interspecific hybrid sterility, to successfully synthesize a new S_1 tetraploid cotton species.

Our results support the hypothesis that distant hybridization is an important driver of new mutations and species in plant evolution [33, 34]. Since the discovery that microsatellite markers are uniformly distributed on cotton chromosomes, SSR markers have been used to monitor the infiltration of heterologous chromosome fragments in the offspring of interspecific hybrids [35, 36]. In the present study, SSR detection revealed the presence of new specific bands in the hybrids that were not detected in the parents, indicating that new combinations or mutations of chromosomes are present in the interspecific progeny (Genomic A and genomic C). This result might be due to chromosomal rearrangements in this newly formed hybrid [37] which was consistent with those reported in previous studies on trispecific hybrids [20]. There is potential for genes from the new synthetic allotetraploids to fuse with favorable genes from *G. herbaceum* and *G. nelsonii*, including genes that

encode good fiber quality, or resistance to diseases. The number of bolls per S_1 plant reached 50 (S2 Fig. a), an important characteristic for high yield breeding applications. We found that a variety of colored fibers developed in S_2 cotton bolls, including brown fibers in a 4-chambered boll and light green and brown cotton fibers in a 3-chambered boll (S2 Fig. b, d). The preliminary examination showed that the disease resistance of the tetraploid S_2 was better than *G. herbaceum* and the same level as *G. nelsonii.* Increased disease resistance also occurs in interspecific hybrids between *G. hirsutum* × *G. arboretum* [19]. This important agronomic characteristic provided a new and important resource for cotton disease resistance breeding programs.

At the same time, there is a need to control segregation in hybrids and establish new genetic isolates. We found that the S_1 progeny in the field showed a phenomenon of gradual decline in growth. The morphological traits of the S_1 plants were generally consistent, with normal growth, flowering, and fruiting. However, the S_2 plants showed dramatic segregation and resulted in different types of plant and corolla shapes, and huge differences in the flowering and fruiting characteristics. The growth of many plants in the S_3 to S_5 generations was suppressed and only a few plants could flower. In severe cases, the plants could not flower and only a small number of plants could bear fruit. This shows that S_1 was genetically unstable germplasm and there is still a risk of natural segregation. The decline in growth potential for hybrid offspring is a common phenomenon in cotton distant hybridization[7,13,15].

To determine the breeding potential of the S_1 population, the hybridization of S_1 with the TM-1 upland cotton germline yielded two different types of hybrid F_1, namely F_1-1 with red flowers and F_1-2 with white flowers, indicating that the different genotypes of parents lead to the diversity and complexity of their hybrid offspring in

the process of genetic recombination. The F_1-1 plants died at the onset of anthesis, indicating that this plant type could not be utilized in crossbreeding. The sterility of F_1-1 hybrid plants was consistent with the results of interspecific hybrids of *G. hirsutum* L. and *G. tomentosum* Nutt.[38], which may be caused by the incoordination of physiological processes in the hybrid offspring. The F_1-2 plants grew normally until flowering and fruiting, but their seeds could not germinate normally. This phenomenon of unsuccessful germination of F_1-2 seeds and root tip wilting had previously been observed during the early generations of the amphiploid *G. arboretum* L. hybridized with *G. bickii* Prokh.[12]. Additional research is required to overcome the key difficulties in promoting seed germination in each generation. The variation in hybrid phenotypes may be related to gene overlap, new genetic variation, chromosome rearrangement, or a variety of other factors [39–40].

The tetraploid S_1 is a new germplasm obtained by chromosome doubling of the diploid F_1 hybrid and is a polyploidy event in cotton. This restored fertility to sterile diploid F_1 hybrids, and the tetraploid S_1 generation obtained showed segregation, which increases the phenotypic mutations in the species, enabling breeders to select for required traits. After the polyploid was generated, not only did its stress resistance not decrease, but biotic stress resistance was maintained. In addition, the fiber quality of the tetraploid S_2 exceeded that of both parents. The S_2 generation had high potential for breeding owing to its boll-forming properties, diversity of fiber colors, and fiber quality. These traits are also of great value for breeding programs. From this we can see that diploid F_1 hybrid polyploidy has important significance for cotton evolution; as the genome structure changed, the species genetic diversity was enriched, and species adaptability and fiber quality was improved. Thus far, we have only performed preliminary characterizations of the newly synthesized allotetraploid cotton

S_1.We have proposed performing more in-depth and comprehensive research to extract the valuable information from this new cotton tetraploid S_1, so as to provide a more accurate theoretical basis for cotton breeding and genetic evolution.

Acknowledgements

We are grateful to Dr Kunbo Wang and Dr Fang Liu of the Cotton Research Institute of Chinese Academy of Agricultural Sciences for kindly providing us with the pollen of *G. nelsonii* Fryx. to use at the National Wild Cotton Nursery, Sanya, China.

References

1. Fryxell PA. A revised taxonomic interpretation of *Gossypium* L.(Malvaceae). Rheedea. 1992; 2(2):108-165.
2. Wang KB, Wendel JF, Hua JP. Designations for individual genomes and chromosomes in Gossypium. J Cotton Res. 2018; 1:3. https://doi.org/10.1186/s42397-018-0002-1.
3. Wendel JF, Brubaker CL, Edward PA. Genetic diversity in *Gossypium hirsutum* and the origin of upland cotton. American Journal of Botany. 1992; 79:1291-1310. https://doi.org/10.1002/j.1537-2197.1992.tb13734.x
4. Iqbal MJ, Reddy OUK, El-Zik KM & Pepper AE. A genetic bottleneck in the 'evolution under domestication' of upland cotton *Gossypium hirsutum* L. examined using DNA fingerprinting. Theoretical and Applied Genetics. 2001; 103:547–554. https://doi.org/10.1007/PL00002908
5. Campbell B, Saha S, Percy R, Frelichowski J, Jenkins J, Park W, et al. Status of the global cotton germplasm resources. Crop Science. 2010; 50:1161-1179. https://doi.org/10.2135/cropsci2009.09.0551er

6. David DF, Hinze LL, Percy R G, Li P, Deng D & Thyssen G. A microsatellite-based genome-wide analysis of genetic diversity and linkage disequilibrium in Upland cotton (*Gossypium hirsutum* L.) cultivars from major cotton-growing countries. Euphytica. 2013; 191:391-401. https://doi.org/10.1007/s10681-013-0886-2
7. Wendel JF, Cronn RC. Polyploidy and the evolutionary history of cotton. Advances in Agronomy. 2003; 78:139-186. https://doi.org/10.1016/S0065-2113(02)78004-8
8. Hutchinson JB, Silow RA, Stephens SG. The evolution of *Gossypium* and the differentiation of the cultivated cottons. The Quarterly Review of Biology. 1949; 24(2):143-144.
9. Satya Narayan Jena, Anukool Srivastava, Krishan Mohan Rai, Alok Ranjan, Sunil K Singh, Tarannum Nisar, et al. Development and characterization of genomic and expressed SSRs for levant cotton (*Gossypium herbaceum* L.). Theoretical and Applied Genetics. 2012; 124:565-576. https://doi.org/10.1007/s00122-011-1729-y
10. Endrizzi JE, Phillips LL. A hybrid between *Gossypium arboreum* L. and *G.raimondii* Ulb. Can J Genet Cytol. 1960; 2(4):311-319. https://doi.org/10.1139/g60-032
11. Muramoto H. Hexaploid cotton: some plant and fiber properties. Crop Sci. 1969; 9(1):27-29. https://doi.org/10.2135/cropsci1969.0011183X000900010009x
12. Li Bl, Zhang XR. Studies on the Hybrid between *G. arboreum* L. and *G.bickii* Prokh.. Acta Genetica Sinica. 1987; 14(2):121-126.
13. YQ Sun, XL Zhang, YC Nie, XP Guo, SX Jin & SG Liang. Production and characterization of somatic hybrids between upland cotton (*Gossypium hirsutum*)

and wild cotton (*G.klotzschianum* Anderss) via electrofusion. Theoretical and Applied Genetics. 2004; 109(3):472-479. https://doi.org/10.1007/s00122-004-1663-3

14. Yu XS, Chu BJ, Liu RE, Sun J, Joseph Jones Brian, Wang HZ, et al. Characteristics of fertile somatic hybrids of *G.hirsutum* L. and *G. trilobum* generated via protoplast fusion. Theoretical and Applied Genetics. 2012; 125(7):1503-1516. https://doi.org/ 10.1007/s00122-012-1929-0
15. Newaskar GS, Chimote VP, Mehetre SS, JadhavSS . Interspecific hybridization in *Gossypium* L.: characterization of progenies with different ploidy-confirmed multigenomic backgrounds. Plant Breed. 2013; 132(2):211-216. https://doi.org/10.1111/pbr.12031
16. Zhang X, Zhai CJ, He LC, Guo Q, Zhang XG, Xu P, et al. Morphological, cytological and molecular analyses of a synthetic hexaploid derived from an interspecific hybrid between *Gossypium hirsutum* and *Gossypium anomalum*. The Crop Journal. 2014; 2(5):272-277. https://doi.org/ 10.1016/j.cj.2014.06.009
17. Liu Q, Chen Y, Chen Y, Wang Y, Chen J, Zhang T. et al. A new synthetic allotetraploid ($A_1A_1G_2G_2$) between *Gossypium herbaceum* and *G. australe*: bridging for simultaneously transferring favorable genes from these two diploid species into upland cotton. PLoS One.2015;10(4):e0123209. https://doi.org/10.1371/journal.pone.0123209
18. Chen Y, Wang YY, Wang K, Zhu XF, Guo WZ, Zhang TZ, et al. Construction of a complete set of alien chromosome addition lines from *Gossypium australe* in *Gossypium hirsutum*: morphological, cytological, and genotypic characterization. Theoretical and Applied Genetics. 2014; 127:1105–1121. https://doi.org/10.1007/s00122-014-2283-1

19. Chen Y, Wang YY, Zhao T, Yang JW, Feng SL, Nazeer W, et al. A new synthetic amphiploid (AADDAA) between *G. hirsutum* and *G.arboreum* lays the foundation for transferring resistances to verticillium and drought. PLoS One. 2015; 10(6):e0128981. https://doi.org/10.1371/journal.pone.0128981

20. Chen D, Wu YX, Zhang XL, Li FG. Analysis of [*Gossypium capitis-viridis* × (*G.hirsutum* × *G.australe*)^2] trispecific hybrid and selected characteristics. PLoS One. 2015; 10(6):e0127023. https://doi.org/10.1371/journal.pone.0127023

21. Wu YX, Chen D, Zhu SJ, Zhang LF, Li LJ. A new sythetic hybrid (A_1D_5) between *Gossypium herbaceum* and *G. raimondii* and its morphological, cytogenetic, molecular characterization. PLoS One. 2017; 12(2): e0169833. https://doi.org/10.1371/journal.pone.0169833

22. Guo W, Cai C, Wang C, Han Z, Song X, Wang K, et al. A microsatellite-based, gene-rich linkage map reveals genome structure, function and evolution in *Gossypium*. Genetics. 2007; 176:527–541. https://doi.org/10.1534/genetics.107.070375

23. Zhang J, Guo W & Zhang T. Molecular linkage map of allotetraploid cotton (*Gossypium hirsutum* L. × *Gossypium barbadense* L.) with a haploid population. *Theore*tical and Applied Genetics. 2002; 105:1166–1174. https://doi.org/ 10.1007/s00122-002-1100-4

24. Zhang J. Fast screening of microsatellite markers in cotton with PAGE/silver staining. Cotton Science Sinica. 2000; 12(5):267–269.

25. Galbraith DW, Harkins KR, Maddox JM, Ayres NM, Sharma DP, Firoozabady E. Rapid flow cytometric analysis of the cell cycle in intact plant tissues. Science (New York). 1983; 220(4601):1049-1051. https://doi.org/10.1126/science.220.4601.1049

26. Wu YX, Yang FH, Zhao XM, Yang WD. Identification of tetraploid mutants of platycodon grandiflorus by colchicine induction. Caryologia. 2011; 64(3):343-349. https://doi.org/10.1080/00087114.2011.10589801

27. Baytar, Asena Akkose Erdogan, Oktay Frary, Anne Frary, Amy Doganlar, Sami. Molecular diversity and identification of alleles for Verticillium wilt resistance in elite cotton (*Gossypium hirsutum* L.) germplasm. Euphytica. 2017; 213:31. https://doi.org/10.1007/s10681-016-1787-y

28. Culp TW, Harrell DC. Breeding methods for improving yield and fiber quality of upland cotton (*Gossypium hirsutum* L.). Crop Science. 1973; 13:686-689. https://doi.org/10.2135/cropsci1973.0011183X001300060030x

29. Qian SY, Huang JQ, Peng YY, Zhou BL, Ying MC, Shen DZ, et al. Studies on the hybrid of *G.hirsutum* L.and *G.anomalum* Wawr. & Peyr. and application in breeding. Sci Agric Sinica. 1992; 25:44-51. https://doi.org/

30. Oakes AJ. A Sterility in certain *Gossypium* hybrids. I. Prefertilization phenomena. Can J Genet Cytol. 1966; 8(4):818-829. https://doi.org/10.1139/g66-099

31. Sacks EJ. Ovule rescue efficiency of *Gossypium hirsutum* × *G. arboreum* progeny from-field grown fruit is affected by media composition and antimicrobial compounds. Plant Cell Tissue & Organ Culture. 2008; 93(1):15-20. https://doi.org/10.1007/s11240-007-9316-2

32. Ram SG, Ramakrishnan SH, Thiruvengadam V, Kannan Bapu JR. Prefertilization barriers to interspecific hybridization involving *Gossypium hirsutum* and four diploid wild species. Plant Breed. 2008; 127(3):295-300. https://doi.org/10.1111/j.1439-0523.2007.01453.x

33. Soltis PS, Soltis DE. The role of genetic and genomic attributes in the success of polyploids. Proc Natl Acad Sci. 2000; 97(13):7051-7057. https://doi.org/10.1073/pnas.97.13.7051
34. Barton NH. The role of hybridization in evolution. Molecular ecology. 2001; 10(3):551-568. https://doi.org/10.1046/j.1365-294x.2001.01216.x
35. Siu L, Saha S, Stelly D, Burr B, Cantrell RG. Chromosomal assignment of microsatellite loci in cotton. Journal of Heredity. 2000; 91(4):326-32. https://doi.org/10.1016/0167-4781(90)90071-9
36. Rieseberg LH, Sinervo B, Linder CR, Ungerer MC, Arias DM. Role of gene interactions in hybrid speciation: evidence from ancient and experimental hybrids. Science. 1996; 272:741-745. https://doi.org/10.1126/science.272.5262.741
37. Kashkush K, Feldman M, Levy AA. Gene loss, silencing and activation in a newly synthesized wheat allotetraploid. Genetics. 2002;160(4):1651-1659. https://doi.org/10.1017/S0016672301005511
38. Guo WZ，Peng ST. Studlies on the morphogenetics and cytogenetics of interspecific hybrids of *G. hirsutum L.* (4x) and *G. tomentosum* Nutt. (4x). Acta Gossypii Sinica. 1997; 9(1):21-24.
39. Loren H. Rieseberg. Evolution: replacing genes and traits through hybridization. Curr Biol. 2009; 19(3):119-122. https://doi.org/10.1016/j.cub.2008.12.016
40. Shaked H, Kashkush K, Ozkan H, Feldman M, Levy A A. Sequence elimination and cytosine methylation are rapid and reproducible responses of the genome to wide hybridization and allopolyploidy in wheat. Plant Cell. 2001; 13(8):1749-1759. https://doi.org/ 10.1105/tpc.010083

Supporting Information

S1 Fig. Growth and formation of the tetraploid S_1. (a) Tetraploid plants with a large pink flower accompanied by yellow pollen. (b) The growth process of the oval boll. (c) Four seeds with brown fibers.

S2 Fig. Fruits and Fibers of the S_1. (a) Multiple bolls of S_1 on multiple fruit branches with the sunken navel of the boll tip showing an ergonomic shape with strong bolls. (b) Four-chambered boll with brown fibers. (c) The brown fibers with fiber length of 13 mm. (d) Three-chambered boll with light green and brown cotton fibers.

S3 Fig. Cotton bolls of the TM-1 and F_1 hybrid (TM-1 × S_1). (a)The normal conical boll of TM-1 shown by the red arrow. The abnormally conical boll of F_1 on the right (b) and on the left (c). (d) The deformed hybrid boll of F_1 indicated by yellow arrows.

Figure 1

Click here to access/download;Figure;Figure 1.tif

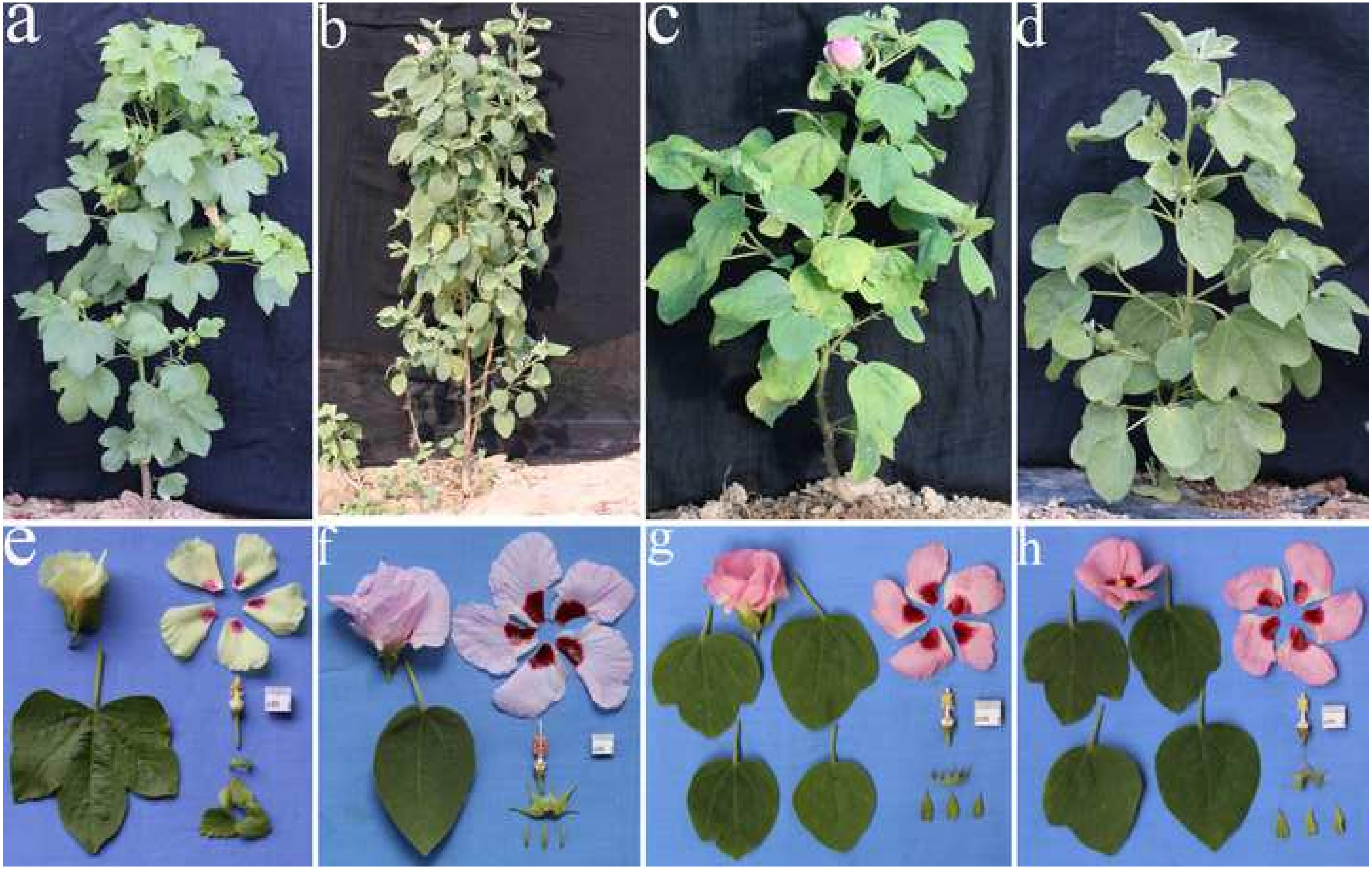

Figure 2

Click here to access/download;Figure;Figure 2.tif

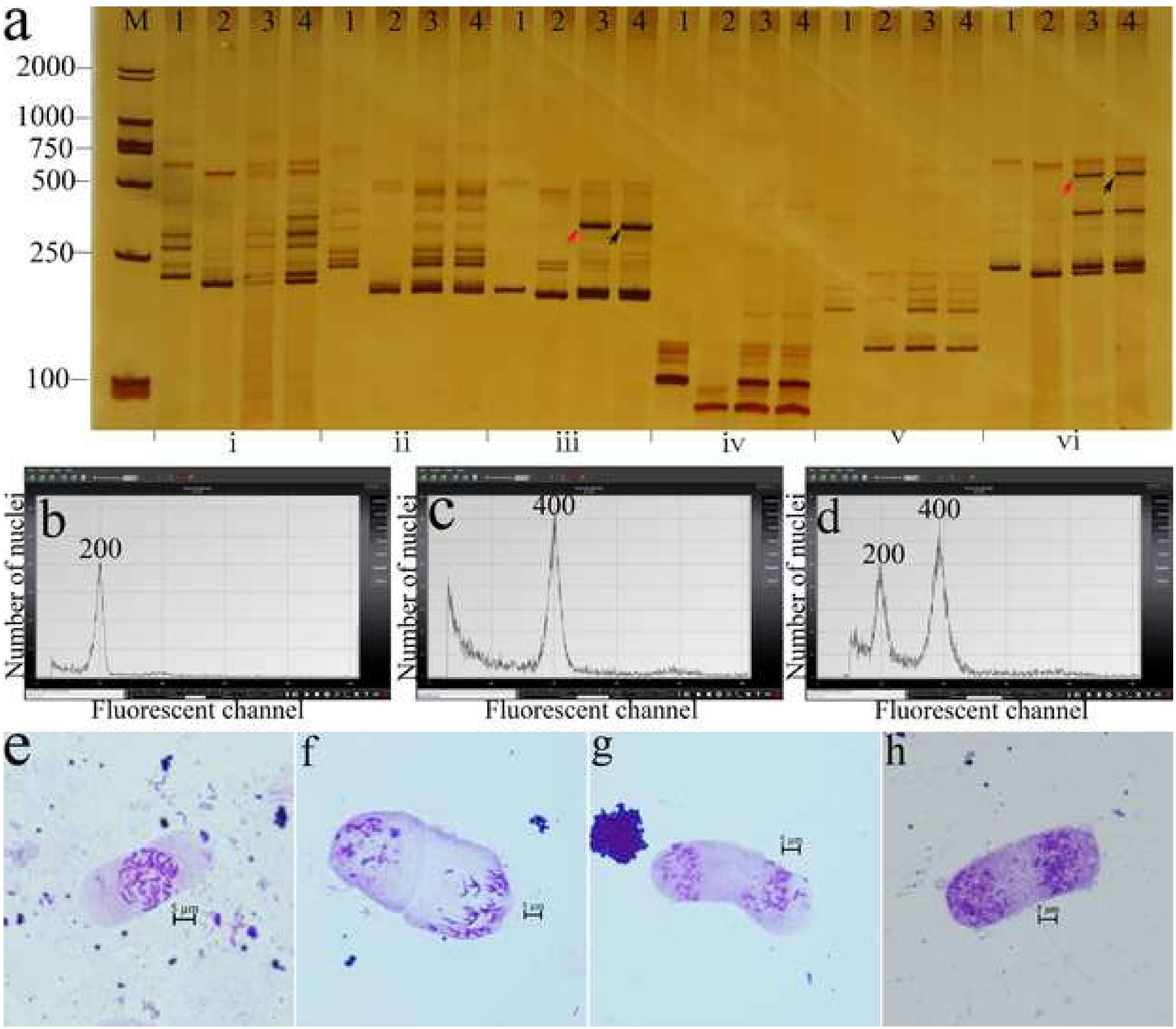

Figure 3

Click here to access/download;Figure;Figure 3.tif

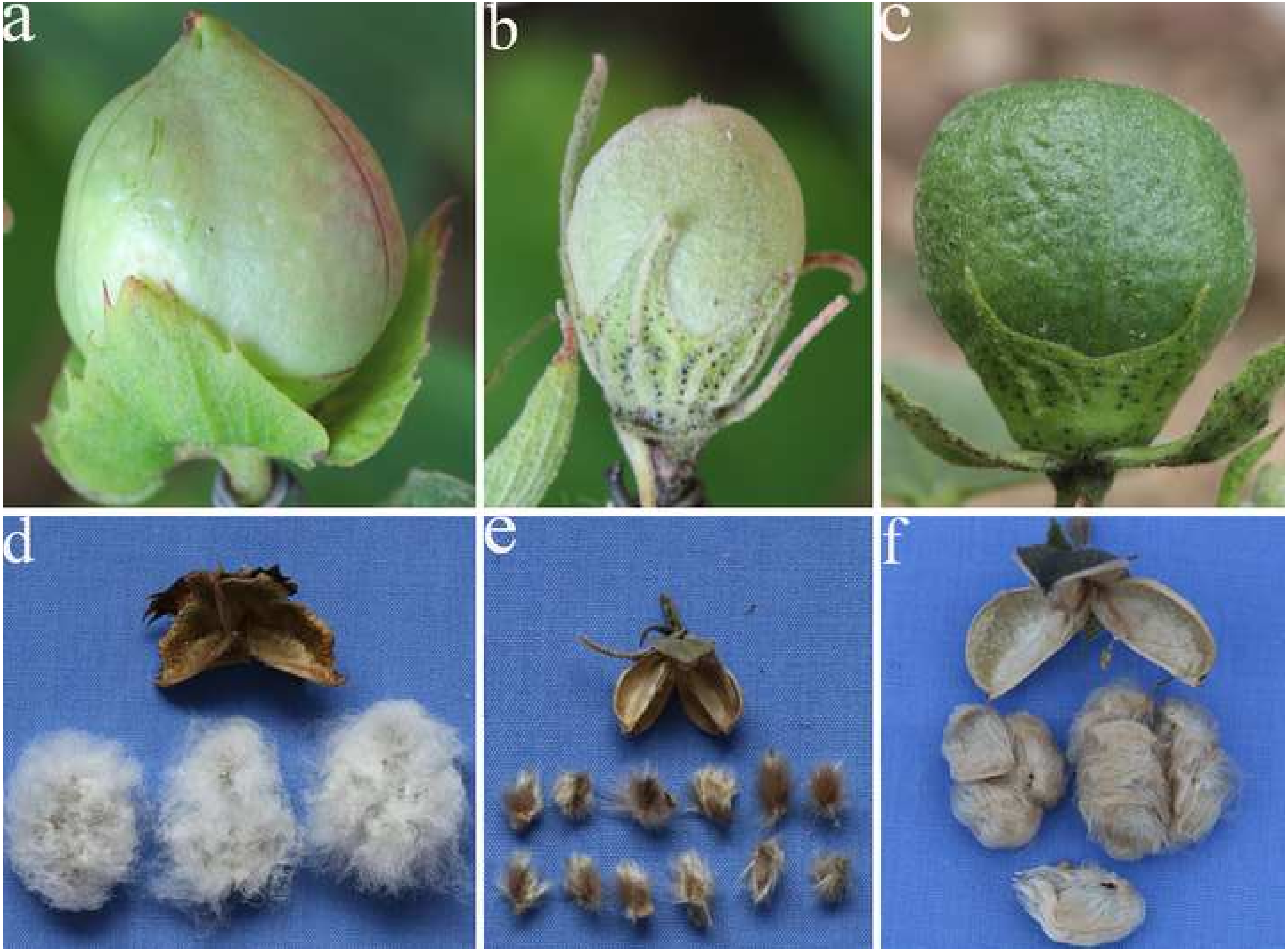

Figure 4

Click here to access/download;Figure;Figure 4.tif

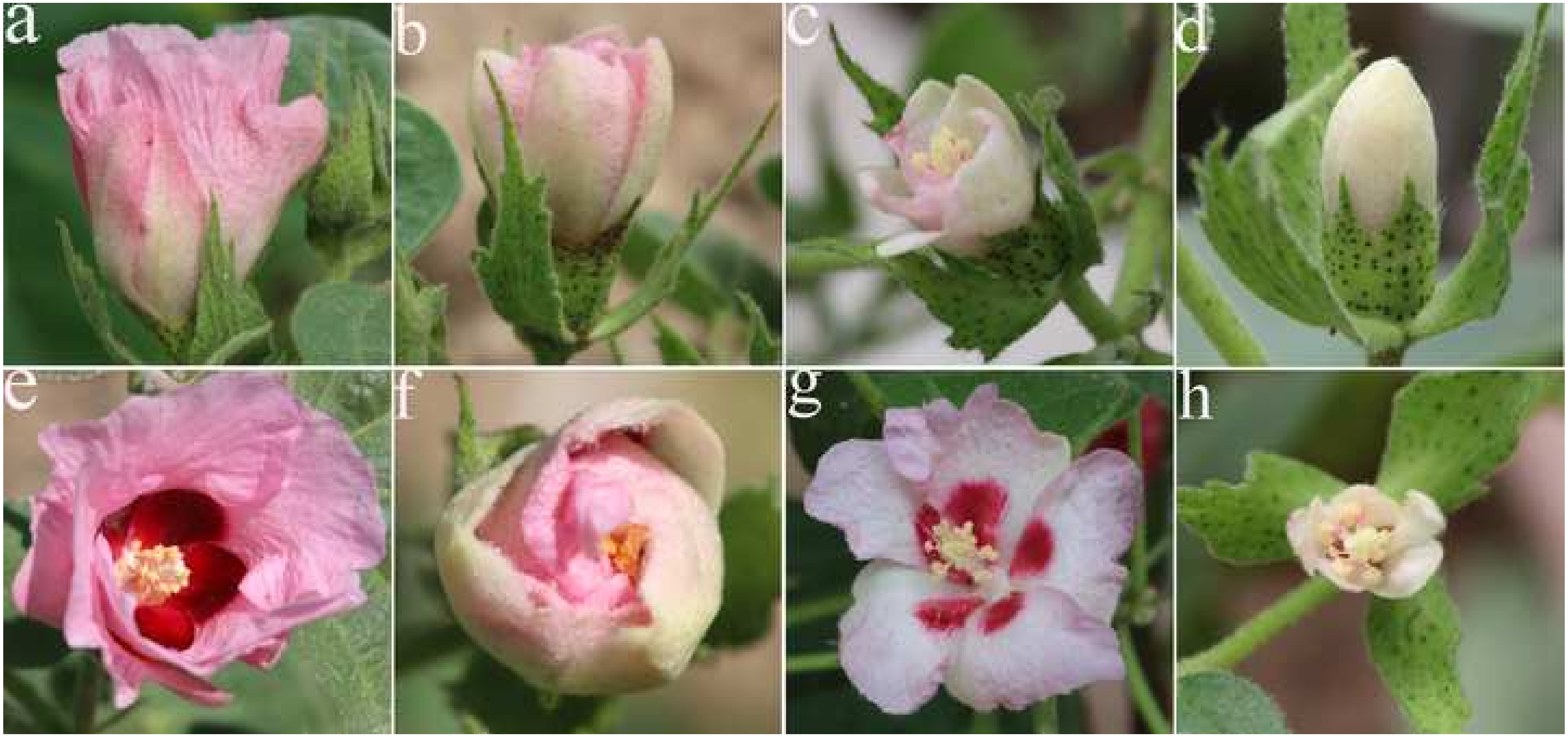

Figure 5

Click here to access/download;Figure;Figure 5.tif

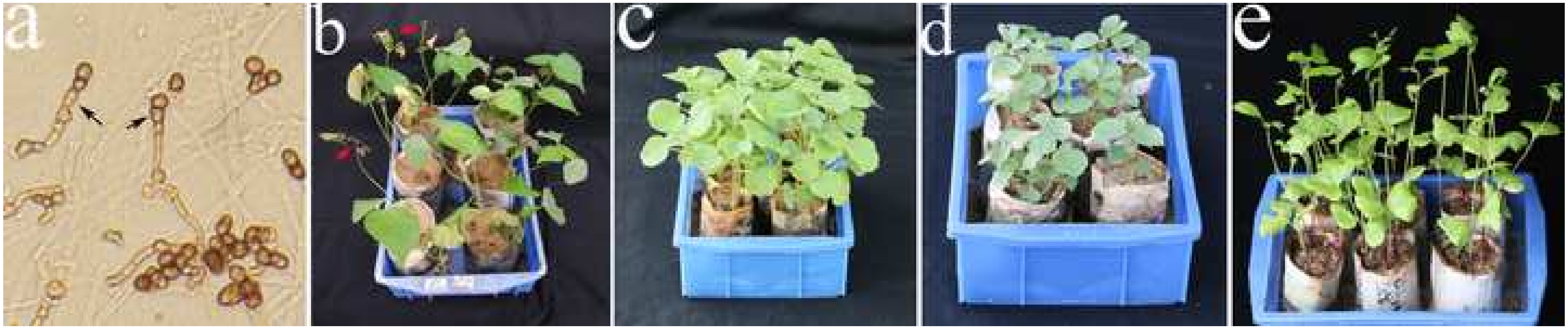

Figure 6

Click here to access/download;Figure;Figure 6.tif

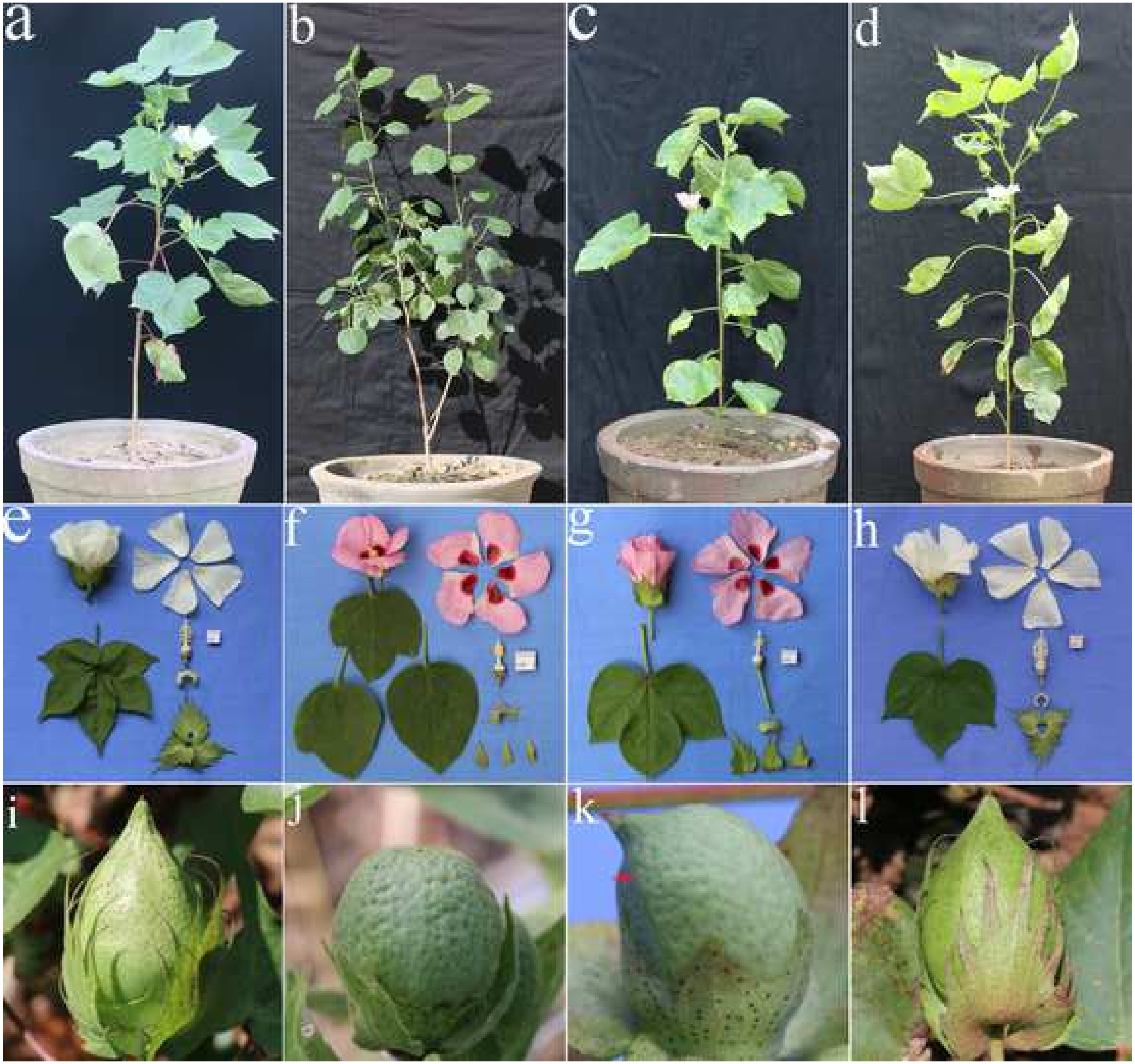

Click here to access/download
Supporting Information - Compressed/ZIP File Archive
Suppoting Information.zip

Morphological ~~and genetic~~ description of a novel synthetic allotetraploid(A$_1$A$_1$G$_3$G$_3$) of *Gossypium herbaceum* L. and *G~~ossypium~~*. *nelsonii* Fryx. suitable for disease-resistant breeding applications

Xiaomin Yin#, Rulin Zhan#,Yingdui He, Shun Song, Lixia Wang, Yu Ge, Di Chen*

Haikou Experimental Station, Chinese Academy of Tropical Agricultural Sciences (CATAS), Haikou, China

*Corresponding author

E-mail: chendi209@163.com (DC)

Keywords: genetic evolution, *Gossypium*, interspecific hybridization, synthetic allotetraploid

Funding sources

This research work was financially supported by the National Natural Science Foundation of China (No. 31701478) and Central Public-interest Scientific institution Basal Research Fund for Chinese Academy of Tropical Agricultural Sciences(No.1630092017004).

Formatted

Formatted: Font: Bold, Font color: Auto

Formatted: Font color: Auto

Formatted: Left

Formatted: Left, Don't add space between paragraphs of the same style

Formatted: Font color: Auto

Formatted: Font color: Auto

Formatted: Font color: Auto

Formatted: Left

Formatted: Left

Formatted: Adjust space between Asian text and numbers

Formatted: Left, Indent: First line: 0 ch

Formatted: Left

Abstract

Gossypium herbaceum L. is a cultivated, diploid cotton species (2n = 2x = 26, A_1A_1) with superior resistance to drought, sap-feeding insects, and cotton leaf curl virus. *Gossypium nelsonii* Fryx. is a wild, diploid cotton species (2n = 2x = 26, G_3G_3) that produces strong fibers and is resistant to drought, aphids, and Verticillium wilt and can delay pigment gland formation.

Wild cotton varieties of *Gossypium* are a great resource of traits that couldfor improvinge commercial cultivars. Previous reports and field surveys show that *Gossypium herbaceum* L. and *Gossypium nelsonii* Fryx. have better disease resistance characteristics than commercial cotton varieties. However, chromosome ploidy and biological isolation make it difficult to directly hybridize diploid cotton species with the tetraploid *Gossypium hirsutum* L. These cotton species were used to synthesize an allotetraploid cotton species for use as a foundation for the future transfer of beneficial genes to cultivated cotton. Here,We successfully developed a new allotetraploid cotton species ($A_1A_1G_3G_3$) using an extended process of distant hybridization within wild cotton species to create new germplasms. wwe used *G. herbaceum* (♀) and *G. nelsonii* (♂) were used for interspecific hybridizations to obtain the F_1 generation. The apical regions of the F_1 plants growth points were treated with immersed in colchicine to induce chromosome doubling and new interspecific hybrid F_1 and tetraploid S_1 populations were confirmed as true hybrids via. Tetraploid S_1 cotton bolls were then obtained from the diploid F_1 plants to successfully synthesize tetraploid cotton plants. Morphological morphological analysis, molecular marker analysis, flow cytometry, and cytological identification. To determine the breeding potential of tThe S_1 population, we was crossed it with a TM-1 line and obtained fertile hybrid offspring were obtained., indicating that the S_1

Formatted: Font color: Auto

Formatted: Left, Indent: First line: 0 ch

Formatted: Font color: Auto

Formatted: Left

Formatted: Font color: Auto

Formatted: Font color: Auto

Formatted: Font color: Auto

Formatted: Font color: Auto

Formatted: Font color: Auto

Formatted: Font color: Auto

line was cross-compatible with TM-1. The S_2 descendants of S_1 were resistant to Verticillium wilt, demonstratinged that the S_1 and S_2 lines had disease resistance potential. Therefore, results show that the S_1 line can be used as the parent material for hybridization with *G. hirsutum* L. to produce pathogen-resistant cotton hybrids. The new S_1 allotetraploid contributes to the enrichment of *Gossypium* germplasm resources and is expected to have importantbe valuableue in polyploidy evolutionary studiess, as well as act as a bridge to transfer beneficial genes from two diploid parental cotton species to *G. hirsutum* L. Moreover, this study describes important techniques for investigating and understanding wild cotton resources.,

markers, detecting DNA polymorphism,following ploidy analysis and microscopic observation were used to identify the tetraploid S_1 plants, and then self-pollination was conducted. The S_1 plants showed good growth without significant segregation, indicating that the compatibility among them had been maintained. The S_2 plants showed obvious segregation, indicating some incompatibility. The S_2 seedlings that were cultivated in nutrient pots were inoculated with Verticillium wilt, but they did not develop the disease and had an immune phenotype. The S_1 interspecific hybrid S_1 plants were hybridized with cultivated *G. hirsutum* L. to measure the compatibility of the S_1 plants and *G. hirsutum* L. and to determine if they could be used for cotton species improvements. The results showed that these plants had some compatibility and that fertile hybrid progeny could be obtained. The newly synthesized allotetraploid S_1 line will have important application value in polyploidy evolution studies and could act as a bridge to transfer beneficial genes from two diploid parental cotton species to *G. hirsutum* L.

Introduction

Formatted: Font color: Auto

Formatted: Font color: Auto

Formatted: Font color: Auto

Formatted: Font color: Auto

Formatted: Font color: Auto

Formatted: Font color: Auto

Formatted: Left, Indent: First line: 0 ch

Formatted: Left

The *Gossypium* genus includes 54 species, of which 50 are wild cotton species and ~~4~~ four are cultivated cotton species ~~including~~made up of ~~(2~~two diploid~~s~~ and ~~2~~ two tetraploid~~s) and 50 are wild cotton species~~ [1, 2]. Approximately 90 % of commercially produced cotton is cultivated from *G. hirsutum* L. ~~Studies have shown that the existing cultivated varieties have undergone long-term, artificial selection and that their yield and fiber qualities, in particular, have been largely developed, making it difficult to compare them with wild species.~~ However, as ~~this long-term, intensive cultivation and artificial selection has resulted in the reduced development of certain resistance traits that are lost during unreasonable selection. Consequently, as~~ the high-yielding quality of cultivated species continuously increases, abiotic and biotic resistance usually does not increase and can even be weakened [3,4]. To increase the resistance in *G. hirsutum* L., the existing genetic basis of cultivated cotton should be expanded to adapt to biotic and abiotic stresses. It is generally believed that wild cotton species contain a large treasure trove of traits that could be used to improve cultivated species [5]. –The~~re are more than 50~~ wild cotton species~~, which~~ are widely distributed in ~~unique~~ various ecological conditions in the Americas, Oceania, Africa, and Asia. These species have undergone long-term~~,~~ natural selection, ~~developed many adaptations,~~ and are resistant to many types of adverse factors, such as disease,~~,~~ insects, drought, heat, and salinity~~, heat, drought, insects, and disease~~. At the same time, there are many mutant varieties, all of which contribute to cotton's rich genetic foundation [6,7].

G. herbaceum L. is a –diploid cotton species cultivated in Western China and Russia. This species is highly resistant to drought, cotton leaf curl virus and sap-feeding insects such as leafhoppers, whiteflies, thrips, and aphids [8, 9]. *G. nelsonii* Fryx. is a wild diploid cotton species that ~~originates~~ originated ~~from~~ in Australia and

Formatted: Font color: Auto
Formatted: Font color: Auto
Formatted: Font color: Auto
Formatted: Font color: Auto
Formatted: Font color: Auto
Formatted: Font color: Auto
Formatted: Font color: Auto
Formatted: Font color: Auto
Formatted: Font color: Auto
Formatted: Font color: Auto
Formatted: Font color: Auto
Formatted: Font color: Auto
Formatted: Font color: Auto
Formatted: Font color: Auto
Formatted: Font color: Auto
Formatted: Font color: Auto

has many economically valuable characteristics, such as delayed pigment gland formation. This is beneficial for producing seeds with low gossypol levels. ~~This is beneficial for producing seeds with low gossypol levels.~~ In addition, it ~~is a potential source of human foodstuff and animal feed;~~ is resistant to blight, Verticillium wilt, aphids, and mites; and is tolerant to abiotic stress conditions such as ~~of drought,~~ high temperatures and drought~~,~~ . ~~and abiotic stresses~~~~.~~ Furthermore, its brown, straight, extensible, and high-strength fibers are ~~a~~ valuable agronomical traits. ~~*G. herbaceum* L. is a cultivated diploid cotton species that is produced in western China and neighboring Russia country . This species is highly resistant to drought, sap-feeding insects (such as leafhoppers, whiteflies, thrips, and aphids), and cotton leaf curl virus [8,9].~~ If these characteristics can be transferred to tetraploid *G. hirsutum* L. (2n = 4x = 52, AADD), then the resulting species will be extremely valuable.

Owing to the biological isolation between the species, it is difficult to directly hybridize diploid cotton species with tetraploid *G. hirsutum*. We are propos~~e~~ing~~summarize~~ a new pathway for transferring genes from two diploid cotton species into tetraploid *G. hirsutum*~~ L.~~. Specifically, two different diploid species undergo ~~artificial~~ interspecific hybridization so that they can ~~rapidly and mutually~~ recognize and overcome their biological segregations to form the diploid hybrid F_1. Following that, artificial ploidy doubling of the apical regions in F_1 hybrids is carried out to obtain a tetraploid cotton species that has the same chromosome number as *G. hirsutum* L.~~, i.e. it is a new tetraploid cotton species.~~ The new tetraploid cotton is then hybridized with the tetraploid *G. hirsutum* ~~L.~~ to achieve efficient gene transfer. This process shortens the time needed to introduce essential genes from two species and is currently the most direct and easiest method that ~~could~~ has the potential to~~ly~~ be utilized with wild cotton. In the last few decades, studies on interspecific hybrids have

Formatted: Font color: Auto

Formatted: Font color: Auto

Formatted: Font color: Auto

Formatted: Font color: Auto

Formatted: Font color: Auto

Formatted: Font color: Auto

Formatted: Font color: Auto

been reported~~we have successfully produced many interspecific hybrids~~, including *G. arboretum* × *G. raimondii* ~~UI~~ [10], *G. hirsutum* × *G. sturtianum* [11], *G. arboreum* × *G. bickii* [12], *G. hirsutum* × *G. klotzschianum* [13], *G. hirsutum* × *G. trilobum* [14], *G. arboreum* × *G. anomalum* [15], *G. hirsutum* × *G. anomalum* [16], ~~and~~ *G. herbaceum* × *G. australe* [17~~].~~], *G.hirsutum* × *G.australe* [18], *G.hirsutum* and *G. arboretum* [19], and *G. capitis-viridis* × (*G.hirsutum* × *G.australe*)^2 [20]. To the best of our knowledge~~However~~, ~~to the best of our knowledge,~~ there has been no report of using *G. herbaceum* and *G. nelsonii* to successfully synthesize an allotetraploid ($A_1A_1G_3G_3$).

~~Owing to the biological isolation between the species, it is difficult to directly hybridize diploid *G. herbaceum* and *G. nelsonii* with tetraploid *G. hirsutum*.~~ ~~Therefore,~~ In this work, we applied~~employed~~ homoploid hybridization to provide a pathway to simultaneously transfer ~~ideal~~ genes from these two diploid cotton species into *G. hirsutum* using *G. herbaceum* as the female parent and *G. nelsonii* as the male parent to successfully generate the interspecific hybrid F_1 (2n = A_1G_3 = 26). Colchicine treatments were used to double the chromosome number~~,~~ and form a new tetraploid cotton genotype (code: S_1) (2n = 4x = 52, $A_1A_1G_3G_3$). –The birth of the new S_1 cotton tetraploid not only enriches the genetic foundation of cotton but also provides a valuable intermediate material for cotton hybridization and breeding. This is a successful example of using wild cotton species for distant hybridization and has important significance for promoting scientific research on cotton genetics~~plants~~.

Materials and methods

Plant material~~s~~

Formatted: Font color: Auto
Formatted: Font color: Auto
Formatted: Font color: Auto, Not Highlight
Formatted: Font: Italic, Font color: Auto
Formatted: Font color: Auto
Formatted: Font: Italic, Font color: Auto
Formatted: Font color: Auto
Formatted: Font color: Auto, Not Highlight
Formatted: Font color: Auto
Formatted: Font: Italic, Font color: Auto
Formatted: Font color: Auto
Formatted: Font: Italic, Font color: Auto
Formatted: Font color: Auto
Formatted: Font: Italic, Font color: Auto
Formatted: Font color: Auto
Formatted: Font color: Auto, Superscript
Formatted: Font color: Auto
Formatted: Font color: Auto, Not Highlight
Formatted: Font color: Auto
Formatted: Font color: Auto
Formatted: Font color: Auto

The diploid *G. herbaceum* cotton species that has been cultivated in China~~,~~ is also kno~~o~~wn as Hongxingcaomian (2n = 2x = 26, A_1A_1), and is a line that has not been subjected ~~undergone any generations of~~to inbreeding. *G. nelsonii* is a diploid cotton species from Australia (2n = 2x = 26, G_3G_3)~~-~~, and the two *G. hirsutum* ~~L.~~ lines (, cv. *Jimian11* ~~–~~(a commercial variety from China) ~~were cotton cv. *Jimian11*~~ and the control variety TM-1 (a prototype for molecular mapping in cotton) were used in our experiments. The~~se~~ lines mentioned above –were obtained from The China National Wild Cotton Nursery in Sanya, China. The *Verticillium dahliae* strain Dahlia Vd080~~-~~, used for disease resistance ~~identification~~ identification, ~~was the Dahlia Vd080 strain and it~~ was provided by the Institute of Cotton Research, Chinese Academy of Agricultural Sciences.

Interspecific hybridization

~~Cotton i~~Interspecific hybridization of the cotton was carried out in Sanya (18°25′34″N, 109°28′25″E), Hainan, China in 2015 [~~18~~21]. When the two parental species flowered at the same time, ~~healthy~~ *G. herbaceum* plants were selected as the female parents. One day before flowering, all stamens in the flowers from the female parents were completely removed. Wax tubes were used to cover the stigmas and non-woven bags were used to cover all of the floral buds~~, entirely, to prevent contamination by external pollen~~. The afternoon before flowering, cotton thread~~ropes~~ ~~were~~ was used to tie the ~~apex~~ apices of the petals in the flowers ~~from~~ of the male parents (*G. nelsonii*), and non-woven bags were used to cover the floral buds entirely~~, to prevent contamination by external pollen~~. From 9 am to 12 pm on the day of flowering, pollen from the male parents was used to pollinate the stigma~~ta~~s of the female parents for hybridization. After pollination was completed, wax tubes were used to cover the

- **Formatted:** Font color: Auto
- **Formatted:** Font color: Auto
- **Formatted:** Font color: Auto
- **Formatted:** Font color: Auto
- **Formatted:** Font color: Auto
- **Formatted:** Font color: Auto
- **Formatted:** Font color: Auto
- **Formatted:** Font color: Auto
- **Formatted:** Font color: Auto
- **Formatted:** Font color: Auto
- **Formatted:** Font color: Auto
- **Formatted:** Font color: Auto
- **Formatted:** Font color: Auto
- **Formatted:** Font color: Auto
- **Formatted:** Font color: Auto
- **Formatted:** Font color: Auto
- **Formatted:** Font color: Auto
- **Formatted:** Font color: Auto

stigmas, non-woven bags were used to cover the entire floral buds, and labels were hung.~~ At 5 pm on the following day, when the hybridized flowers had withered, the non-woven bags were removed to promote normal growth in the hybridized bolls.~~ After the cotton bolls had matured~~ and underwent dehiscence~~, the seeds were removed and sown in nutrient pots containing sterile soil. After one or two~~1–2~~ true leaves had sprouted, the plants were transplanted into fields and normal fertilizer and water management was carried out. The morphological characteristics of the plants were observed, including the type of plant, stems, leaves, flowers~~,~~, and bolls~~, etc~~. The morphological characteristics of each plant part at different biological stages were photographed using a digital camera, and the photographs were edited by using Photoshop software.~~We further speculated as to whether the plant was a true interspecific hybrid F_1. A Canon digital camera (EOS 70D) equipped with an F1.8 lens with a macro of RF 35 mm was used to photograph the morphological characteristics at the various sites. The Photoshop CS5 software (Adobe Inc) was used to edit all acquired images.~~

~~No permits were required for the described study, which complied with all relevant regulations.~~

Formatted: Font color: Auto

SSR molecular marker analysis in F_1 hybrids~~validation of the F_1 hybrids~~

We randomly selected 568 pairs of SSRs based on our cotton genetic map [~~19~~22] to screen for parental polymorphisms. The polymorphic primers obtained were used to validate the authenticity of the interspecific F_1 hybrids. These SSR primer sequences can be found at http://www.cottonmarker.org. A programmable gradient thermal

Formatted: Font color: Auto

cycler (Bio-Rad T100) was used for SSR-PCR amplification[~~20~~23]. Electrophoresis and silver staining of the PCR products were carried out according to previously reported methods ~~previously reported~~ [~~21~~24]. The bands generated by electrophoresis were photographed ~~using a camera~~ and edited by Photoshop software.

~~A Canon digital camera (EOS 70D) equipped with a F1.8 lens with a macro of RF 35 mm was used to photograph the electrophoresis bands. The Photoshop CS5 software (Adobe Inc) was used to edit all images.~~

~~F_1 hybrid c~~Chromosome duplication in the F_1 interspecific hybrid~~doubling~~

In 2016~~-~~–2018, the F_1 hybrids that were validated by the SSR molecular markers were grafted ~~for storage and breeding~~. The temperature was kept at ~~At~~ 25 °C~~℃,~~ and the apical growth points of the~~se~~ F_1 hybrids were immersed in 0.30 % (w/v) colchicine for 6 h~~ours~~. After immersion, they were placed in pots in the greenhouse that were not exposed to light and incubated until new growth points or floral buds sprouted. When the length of the new meristematic ap~~e~~icesxes~~growth points~~ reached 5 cm, they were removed and grafted for storage ~~and breeding~~ and further validation of the chromosome doubling results ~~was carried out~~ [~~22~~20]. Newly sprouted floral buds could grow until the afternoon before flowering. ~~Then~~ Then, a ~~rope~~ cotton thread was used to tie the tip of the petal for self-pollination and non-woven bags were used to cover the entire floral bud to prevent outcrossing. After the cotton bolls matured and underwent dehiscence, the seeds were removed and sown in nutrient pots. After one or two~~1–2~~ true leaves sprouted, the plants were transplanted into fields. The morphological characteristics of the plants were observed, including the ~~type of~~ plant

Formatted: Font color: Auto

Formatted: Font color: Auto

Formatted: Font color: Auto

Commented [A1]: Range is denoted with an en dash rather than a hyphen. I have checked that the correct punctuation has been used throughout the manuscript.

Formatted: Indent: First line: 0 ch

Formatted: Font color: Auto

Formatted: Font color: Auto

Formatted: Font color: Auto

Formatted: Font color: Auto

Formatted: Font color: Auto

Formatted: Font color: Auto

Formatted: Font color: Auto

shape, stems, leaves, flowers, and bolls~~,~~. The ~~obtained~~ resulting –tetraploid plant was named S_1, and ~~though~~ chromosome counting and flowcytometric analysis were performed for validation. The morphological characteristics of each plant part were photographed using a camera, and the photographs were edited by using Photoshop software. ~~and other major morphological traits and further validation of the chromosome doubling results was carried out. A Canon digital camera (EOS 70D) equipped with a F1.8 lens with a macro of RF 35 mm was used to photograph the morphological characteristics in various sites. The Photoshop CS5 software (Adobe Inc) was used to edit all images.~~

Ploidy analysis

In order to detect whether the S_1 chromosome ha~~s~~d been doubled, flow cytometry was used to examine the differences in the chromosome numbers between the F_1 hybrid and S_1, simultaneously ~~D~~diploid *G. herbaceum* L. and the tetraploid TM-1 ~~tetraploid *G. hirsutum* L.~~ lines were used as controls ~~and flow cytometry was used to examine the differences in the chromosome numbers between the F_1 hybrid and its resultant tetraploid S_1 progeny~~. The operational steps ~~–are~~were divided into p~~P~~reparation of the sample cell suspensions, specific staining of DNA, and loading~~.~~, Specific methods were carried out according to the description of Galbraith ~~et al.~~ [~~23~~25]. ~~The~~ A Sysmex-Partec CyFlow Cube8 flow cytometer was used to determine ~~the ploidy in~~ the nuclear DNA content. ~~The~~ CellQuest Pro software was used to analyze the ~~peak values in the channel~~results. The experimental data are displayed in a single-parameter histogram~~,~~. ~~t~~The ordinate represents the number of nuclei (count), and the abscissa represents the relative intensity of the fluorescent channel.

Formatted: Font color: Auto
Formatted: Font color: Auto
Formatted: Font color: Auto
Formatted: Indent: First line: 0 ch
Formatted: Font color: Auto
Formatted: Font color: Auto
Formatted: Font color: Auto
Formatted: Font color: Auto
Formatted
Formatted: Font color: Auto
Formatted: Font color: Auto
Formatted: Font color: Auto
Formatted: Font color: Auto
Formatted: Font color: Auto
Formatted: Font color: Auto
Formatted: Font color: Auto
Field Code Changed
Formatted: Font color: Auto
Formatted: Font color: Auto
Formatted: Font color: Auto
Formatted: Font color: Auto

Karyotype analysis

Root tip chromosome observations

G. herbaceum and *G. nelsonii* were used as controls and mMicroscopic observations of the number of root tip chromosomes in the F_1 hybrid and its resultant tetraploid S_1 progenyies were carried out, to observe the differences in the chromosome number between the two genotypes, and *G. herbaceum* and *G. nelsonii* were used as controls. Finally, karyotype analysis of the F_1 hybrid was performed for determination of chromosome doublingchromosome doubling result was determined. Root tip fixation, preservation, dissection, squash preparation, and microscopy were carried out according to previouslythe reported methods of Wu et al. [2426]. A Nikon Eclipse Ni-E biological microscope and camera system was used to observe, count, and photograph the chromosomes in the root-tip cells. Photoshop software was used to edit the images.

The biological microscope (MODEl ECLIPSE Ni-E microscope and photography system, Nikon) was used to observe the root tip chromosome number and acquire photographs. The Photoshop CS5 software (Adobe Inc) was used to edit all images.

Validation Determination of disease resistance in the tetraploid S_2

Take S_2 descendants of S_1 were chosen as the target of disease resistance identification,. The tetraploid S_2 seedlings, growing in nutrient pots, were inoculated indoors with the TThe *Verticillium dahliae* strain (code: Dahlia Vd080 strain) was inoculated indoors onto tetraploid S_2 seedlings growing in nutrient pots for preliminary determination of disease resistance. The strain cv. *Jimian11* was used as

Formatted: Left

Formatted: Left, Indent: First line: 6 ch

Formatted

Formatted: Font color: Auto

Formatted: Font color: Auto

Formatted: Font color: Auto

Formatted: Font color: Auto

Formatted: Left

Formatted: Indent: First line: 0 ch, Space Before: Auto, After: Auto

Formatted: Indent: First line: 0 ch

Formatted: Font color: Auto

Formatted: Font color: Auto

Formatted: Font color: Auto

Formatted: Font color: Auto

an infection control group~~.~~; *G. herbaceum* and *G. nelsonii* were used as disease resistance controls~~ and the S_2 were the determination targets~~. In the experiment, there were ~~2~~ two treatments (fungal suspension ~~and~~ or sterile water for inoculation ~~respectivly~~) performed in ~~and~~ triplicate~~s~~. Each replicate of the experiment contained 540 plants.~~Every replicate contained 540 plants.~~

Inoculations of the seedlings in the nutrient pots were carried out according to a previously reported method [~~25~~27]. Seeds were sown in cylindrical paper pots (height 8 cm and diameter 5 cm) with no bottom and filled with sterile medium. When the first true leaf had grown until it was level, 10^7 conidia/ml of suspension was directly injected into the 8-cm-dimeter plastic tray~~ with a diameter of 8 cm~~. ~~In contrast,~~ Only sterile water ~~in the control tray, sterile wate~~was ~~r~~used for inoculation in the~~as~~ control groups~~.~~ ~~was injected.~~ The paper pot and seedlings were placed upright in the trays so that the root systems adsorbed the suspension liquid.~~ The seedlings were incubated until disease onset.~~ Examination of the disease symptoms was carried out according to ~~the~~ previously reported methods ~~ previously reported by Peng et al.~~ [~~26~~19]. Changes in leaf color and degree of wilting were used as a basis for preliminary observations of disease resistance in the experimental materials.

Hybridization between the S_1 interspecific hybrid and *G. hirsutum* (TM~~-~~-1 cultivar)

~~Tetraploid S_1 breeding application test~~

In the winter of 2017, hybridization was carried out in Sanya, Hainan province, China, using the tetraploid S_1 as the male parent (♂) and the TM-1 ~~*G. hirsutum* L.~~ line (♀) as the female parent. The hybridization method used was similar to the ~~hybridization~~

Formatted: Font color: Auto
Formatted: Font color: Auto
Formatted: Font color: Auto
Formatted: Font color: Auto
Formatted: Font: 12 pt, (Asian) Chinese (PRC), (Other) English (United States)
Formatted: Font color: Auto
Formatted: Font: 12 pt, (Asian) Chinese (PRC), (Other) English (United States)
Formatted: Font color: Auto
Formatted: Font color: Auto
Formatted: Font color: Auto
Formatted: Font color: Auto
Formatted: Font color: Auto
Formatted: Font color: Auto
Formatted: Font color: Auto
Formatted: Font color: Auto
Formatted: Font color: Auto
Formatted: Left
Formatted: Left
Formatted: Left, Line spacing: Double, Don't adjust space between Latin and Asian text, Don't adjust space between Asian text and numbers

~~method~~ previously described for *G. herbaceum* (♀) and *G. nelsonii* (♂) [~~18~~21]~~.~~ ~~Investigate~~ , , and ~~record~~ the boll-forming rate and effective boll-forming rate of hybrids ~~was~~ , were determined. After the hybridized cotton bolls matured and underwent dehiscence, seeds were removed and sowed in nutrient pots containing sterile soil. After ~~1–2~~one or two true leaves sprouted, the plants were transplanted into fields. The morphological characteristics of the plants were observed, including ~~type of~~ plant shape, stems, leaves, flowers, ~~–~~ and bolls~~, and other major morphological traits~~. ~~According to~~Based on the morphological characteristics, the authenticity of the hybrids ~~were~~ was preliminarily ~~speculated~~ assessed and the fertility of the tetraploid S_1 hybrids was examined.

The morphological characteristics of each plant part were photographed, and the images were edited with Photoshop software.

~~A Canon digital camera (EOS 70D) equipped with a F1.8 lens with a macro of RF 35 mm was used to photograph the morphological characteristics at the various sites. The Photoshop CS5 software (Adobe Inc) was used to edit all images.~~

Image acquisition and editing

A Canon EOS 70D digital camera with an RF 35 mm F1.8 Macro Lens was used to photograph the morphology of plant parts and electrophoresis bands of the PCR products. The images were edited with Adobe Photoshop CS5 software.

–

Results

F_1 hybrid generation and trait observations

Formatted: Font color: Auto

Formatted: Font color: Auto

Formatted: Font color: Auto

Formatted: Font color: Auto

Formatted: Font color: Auto

Formatted: Font color: Auto

Formatted: Font color: Auto

Formatted

Formatted: Left, Don't add space between paragraphs of the same style, Line spacing: Double, Don't adjust space between Latin and Asian text, Don't adjust space between Asian text and numbers

Formatted: Font color: Auto

Formatted: Font color: Auto

Formatted

Formatted: Left, Don't add space between paragraphs of the same style, Line spacing: Double, Don't adjust space between Latin and Asian text, Don't adjust space between Asian text and numbers

Formatted: Left

In 2016, hybridization was conducted using *G. herbaceum* as the female parent and *G. nelsonii* as the male parent. A total of 235 flowers were pollinated, ~~and~~ 205 of which formed bolls. However, most bolls did not form seeds and only ~~3~~ three effective bolls were finally obtained~~.~~; ~~Each~~ each ~~of these bolls~~ containing ~~1~~ one seed. In 2017, the ~~3~~ three seeds were sown in a pot and all germinated after 5 days. After the first true leaves were level, the seedlings were transplanted to the field. Floral buds appeared after 43–45 days and flowering occurred after 68–70 days. In Sanya, China, the ~~3~~ three F_1 plants showed exuberant growth, were tall, produced large numbers of flowers throughout the year, and could produce pollen. However, the pollen produced was non-viable and did not produce bolls. The samples have been preserved, but are still infertile. This highlights the difficulty in obtaining F_1 hybrids from interspecific hybridizations between *G. herbaceum* L. and *G. nelsonii* Fryx.~~, and fertility restoration was also difficult~~.

~~According to the 14 – item investigation data analysis (Table 1), the~~ The morphological traits of the F_1 plants showed classical hybrid characteristics(Figs. 1~~, and 2~~) according to the 14–item investigation data analysis (Table 1). Morphological comparison with parental plants showed that two items were greater than the parents: plant height (Fig. 1a–c) and corolla color (Fig. 1e–g). ~~5 f~~Five items were intermediate between the parents~~ including~~: leaf shape and color, number of lobes, bract shape, calyx teeth shape, and number of bract teeth (Fig. ~~2A~~1e ~~–Cg),~~). ~~2 two items were greater than the parents including plant height(Fig. 1A – C) and corolla color(Fig. 2A–2 C), 2 t~~Two items ~~was~~ were similar to the female parent, ~~including~~ anther and pollen color ~~(((~~Fig. ~~2A~~1e, ~~21Cg)),~~). ~~Fand 5 f~~five items were similar to the male parent ~~including~~ ~~,~~: stem and leaf trichomes, flowering habit, bract extension, flower opening, and extrafloral nectary (Fig. ~~2B~~1f, ~~2C~~1g).

Formatted: Left, Indent: First line: 0", Don't add space between paragraphs of the same style, Don't adjust space between Asian text and numbers

Formatted: Font color: Auto

Formatted: Font color: Auto

Formatted: Font color: Auto

Formatted: Font color: Auto

Formatted: Font color: Auto

Formatted: Font color: Auto

Formatted: Font color: Auto

Formatted: Font color: Auto

Formatted: Font color: Auto

Formatted: Font: Not Bold

Formatted: Left, Indent: First line: 2.5 ch

Formatted: Font color: Auto

Formatted: Font color: Auto

Formatted: Font color: Auto

Formatted: Font color: Auto

Formatted: Font color: Auto

Formatted: Font color: Auto

Formatted: Font color: Auto

Formatted: Font color: Auto

Formatted: Font color: Auto

Formatted: Font color: Auto

Formatted: Font color: Auto

Formatted: Font color: Auto

Table 1. Comparison of morphological traits between *G. herbaceum* L. × *G. nelsonii* Fryx. F_1 hybrid and its ~~parents~~parental genotypes

Character	*G. herbaceum* L.	*G. nelsonii* Fryx.	F_1
Plant height (cm)	80	200	300
Stem/branch characteristics	Less trichomes	More trichomes	More trichomes
Shape and color of leaf	Broad-leaved, Green	Ovoid, gray green	Broad-leaved, ovoid, subcircular, Green
No. of lobes	3–5	0–1	0–3
Bract size (length × width, cm)	1.1×1.3	1.2×0.2	1.1×0.5
Bract shape, No. of teeth	Heart-shaped, 4–6	Lanceolate, 1	Sword-shaped, 1–3
Bract jointing	Joint	No joint	No joint
Floral nectary	Intra-floral	Extrafloral	Extrafloral
Calyx	Cup-shaped	Wavy	Serration
Bract tooth	5–9	1	4–6
Corolla shape and color~~,~~ ~~corolla~~ Corolla length (cm)	Yellow trumpet, around 2.1	Light pink, funnel-shaped, around 3.5	Pink trumpet, around 2.5
Color and size of spot	Rose	Purple	Red
Filaments colors	Pink interspersed with milky white	Rose	Light pink
Anther colors	Yellow	Rose	Light yellow
Pollen colors	Yellow	Milky white	Yellow
Stigma colors	Milky white	Light green	Milky white
Boll	Conical	Oval	/
Fiber	Spiral	Upright	/

Figure 1. Pictures of parent and hybrid plants, leaves , flowers, corollas, basal patches, filaments, anthers, pollen, calyxes, and bracts.. (A) The female *G. herbaceum* L. female parent had a tower shape(a, e) with shorter and more compact branches., (B) The male *G. nelsonii* Fryx. was cylindrical(b, f) with short and compact branches., the (C) The diploid F_1 hybrid was tower_ shaped, with longer branches and looser spacing but were infertile(c, g.), (D) The tetraploid, the S_1, were tower-shaped with long fruit branches and loose spacing. ,They they were fertile(d, h).

Figure 2. Pictures of leaves and flowers, corollas, basal patches, filaments, anthers, pollen, calyxes, bracts. (A) *G. herbaceum* . (B) *G. nelsonii* Fryx. (C) The F_1 hybrid (D) The tetraploid S_1.. (A) *G. herbaceum* L. bore bell-shaped flowers with yellow corollas, rose basal patches, pink and white alternating filaments, yellowish anthers, yellow pollen, cup-shaped calyxes, heart-shaped bracts grown together separately rather than together, and were broad-leaved. (B) *G. nelsonii* Fryx. bore tubular-shaped flowers with light pink corollas, purple basal spots, rose filaments and anthers, milky pollen, wavy calyxes, lanceolate bracts that were detached rather than united, and ovoid leaf blades. (C) The F_1 hybrid had bell-shaped flowers with pink corollas, red basal patches, light pink filaments, light yellow anthers and pollen, serrated calyxes, sword-shaped bracts that grow separately rather than together, and had broad-leaved, ovoid, and subcircular leaves. (D) The S_1 had trumpet and funnel-shaped flowers with alternating red and white corollas, red basal patches, light pink filaments, yellow anthers and pollen, serrated and wavy calyxes, triangle dentate and ensiform bracts that grew separately rather than together, and had broad-leaved, ovoid, and subcircular leaves.

Formatted: Not Highlight
Formatted: Font color: Auto
Formatted: Not Highlight
Formatted: Not Highlight
Formatted: Not Highlight
Formatted: Not Highlight
Formatted: Not Highlight
Formatted: Not Highlight
Formatted: Not Highlight
Formatted: Not Highlight
Formatted: Font: Not Bold
Formatted: Font: 12 pt, Not Highlight
Formatted: Normal, Space Before: Auto, After: Auto, Don't adjust space between Asian text and numbers, Pattern: Clear
Formatted: English (United States)

SSR molecular marker determination was carried out and ~~6~~ six pairs of parental polymorphic primers (Table 2) were obtained from the screening. These ~~6~~ six pairs of primers could be used to amplify the complementary bands from the parents in the F_1 hybrids (Fig. ~~3A~~2a), of which ~~2~~ two primer pairs (NAU1052 and NAU3093) ~~not only~~ amplified not only the bands that are characteristic ~~to~~ of the parents but also characteristic the bands of the hybrid itself (Fig. 2a/iii, 3a/vi) ~~(Fig. 3Ac, 3Af)~~, showing that the F_1 hybrids are true hybrids. Flow cytometry showed that the peak value of the control sample of *G. herbaceum* was approximately 200 channels (Fig. ~~3B~~2b~~),~~); the peak value of TM-1 was close to 400 channels (Fig. ~~3C~~2c~~),~~); and the peak value of the F_1 hybrid was close to 200 channels (Fig. ~~3D~~2d), ~~showing~~ indicating that the F_1 hybrid was a diploid. Root tip staining and microscopy observations showed that the root tip chromosome number of both the *G. herbaceum* and *G. nelsonii* control samples ~~was 26 pairs~~ (Fig. ~~3E~~2e, ~~3~~2~~F~~f), ~~while the root tip chromosome number of~~ and the F_1 hybrid (Fig. ~~3~~2~~G~~g) was also 26 pairs ~~(Fig. 3G)~~, ~~which proved~~~~also~~ indicating that the F_1 hybrid was a diploid.

Formatted: Font color: Auto
Formatted: Font color: Auto
Formatted: Font color: Auto
Formatted
Formatted: Font: (Default) SimSun
Formatted
Formatted: Font: (Default) SimSun
Formatted: Font color: Auto
Formatted: Font color: Auto
Formatted: Font color: Auto

Table 2. SSR polymorphism primer sequences

Primer number	Forward sequence (5′–3′)	Reverse sequence (5′–3′)
NAU1157	GAGTTTGGTTCTGGGTTGAG	GATCCTTTTCATCTCCTCCA
NAU1355	ATCTGTTTACGCCACTCTCC	CCAGCCTTTGACATTTTTCT
NAU1052	CGCAGATAAAGGATGGATTT	AGAGCTGGAGGACATAACAAA
JESPR156	GCCTTCAATCAATTCATACG	GAAGGAGAAAGCAACGAATTAG
BNL1679	AATTGAGTGATACTAGCATTTCAGC	AAAGGGATTTGCTGGCAGTA
NAU3093	GTCTTGAACCGGAACTTGAT	TCCTGTTGAACACCAAAGTG

Figure ~~3~~2. Validation of F_1 and S_1 by detecting DNA polymorphism, ~~following~~ ploidy analysis, and the number of chromosomes~~microscopic observation~~. (~~A~~a) Microsatellite loci identified by SSR primers including (i) NAU1157 ~~(a)~~, ~~–~~(ii) NAU1355 ~~(b)~~, (iii) NAU1052 ~~(c)~~, ~~–~~ (iv) JESPR156 ~~(d)~~, (v) BNL1679 ~~(e)~~, and (vi) NAU3093 ~~(f)~~. The novel bands produced in F_1 and S_1 are indicated by red and blue arrows~~,~~ (1~~–~~, *G. herbaceum* ~~L.~~; 2~~–~~, *G. nelsonii* ~~Fryx.~~; 3~~–~~, the ~~the~~ F_1; 4~~–~~, the ~~the~~ S_1). (~~B~~b) ~~Distribution peak of~~ *G. herbaceum* ~~L.~~ at ~~approximately~~ 200 channels. (~~C~~c) ~~Distribution peak of~~ TM-1 at ~~approximately~~ 400 channels~~.~~, respectively. (~~D~~d) ~~Distribution peak of the diploid~~ F_1 ~~hybrid~~ and ~~the tetraploid~~ S_1 at ~~approximately~~ 200 channels and 400 channels respectively. ~~Fluorescence intensities of propidium iodide stained genomic DNA.~~ (~~E~~e) ~~The~~ 26 ~~chromosomes in~~of *G. herbaceum* ~~L~~. (~~F~~f) ~~The~~ 26 ~~chromosomes in~~of *G. nelsonii* ~~Fryx~~. (~~G~~g) ~~The~~ 26 ~~chromosomes in~~of the F_1 ~~hybrid~~. (~~H~~h) ~~The~~ 52 ~~chromosomes in~~ of the S_1 ~~tetraploid~~. The root tip cells (red arrows) and the chromosomes (black arrows) stained with Carbol fuchsin solution under 40× microscope.

Formatted: Left
Formatted: Font color: Auto
Formatted: Font color: Auto
Formatted: Font color: Auto
Formatted: Font color: Auto
Formatted: Left
Formatted: Left
Formatted: Left
Formatted: Left
Formatted: Left
Formatted: Left
Formatted: Left
Formatted: Font color: Auto
Formatted: Font color: Auto
Formatted: Font color: Auto
Formatted
Formatted: Font color: Auto
Formatted: Font color: Auto
Formatted: Font color: Auto
Formatted: Font color: Auto
Formatted: Font color: Auto
Formatted: Font color: Auto
Formatted: Font color: Auto
Formatted: Font color: Auto
Formatted: Font color: Auto
Formatted: Font color: Auto
Formatted: Font color: Auto
Formatted: Font color: Auto
Formatted: Font color: Auto
Formatted: Font color: Auto
Formatted: Font color: Auto
Formatted: Font color: Auto
Formatted: Font color: Auto
Formatted: Font color: Auto
Formatted: Font color: Auto
Formatted: Font color: Auto
Formatted: Font color: Auto
Formatted: Font color: Auto
Formatted: Font color: Auto
Formatted: Left, Indent: First line: 0 ch, Space Before: Auto, After: Auto, Don't adjust space between Asian text and numbers

Tetraploid S_1 generation and trait observations

After the apical growth point of the F_1 hybrid underwent chromosome doubling, the first large red flower grew in the apex of the plant after 27 days (S1 Fig. a)~~(Fig. 4A)~~. The flower was full of pollen and a boll containing four seeds was formed through self-pollination (S1 Fig. ~~4B~~b, c). ~~The boll contained four4 seeds (Fig. 4C).~~ In 2018, the ~~4~~ four seeds were sown in a pot and all germinated after 5 days. After the first true leaves were level, the seedlings were transplanted to the field. Floral buds appeared after 45-50 days and flowering occurred after 70–75 days. Boll opening occurred after 107–115 days. In Sanya, ~~China,~~ the ~~4~~ four S_1 plants grew normally, with medium plant height and flowering that ~~occurs~~ occurred throughout the year. The pollen formed was viable and ~~could form a lot of~~ many bolls were formed. The samples that were preserved were fertile. Chromosome doubling of the F_1 hybrids restored their fertility. We hypothesized that the ~~4~~ four S_1 plants were all tetraploid hybrids and were assigned the code S_1 (2n = 4x = 52, $A_1A_1G_3G_3$). The differences in the morphological characteristics between the ~~4~~ four S_1 plants were not ~~great~~large. Most plants developed large red flowers, with radiating purple patches, and significant separation was not observed. The leaves were large and tough, and the plants were tall~~,~~ and shrub-like (Fig. ~~1D~~1d). The plants ~~could~~ produced many flowers, released pollen, and produced fruit normally. The fruits formed were small bolls containing small seeds, and short, brown, ~~and~~ high-strength fibers (Fig. ~~5F~~3f). In particular, the S_1 plants retained some characteristics of *G. nelsonii* Fryx. For example, at low temperatures, the buds on the lower branches were extremely small (0.50 cm length), milky white in color, ~~and~~ did not open until wilting, and had closed pollination. In contrast, flowers in the middle and top branches were large (4.40 cm length), opened normally, and had open pollination. The red flowers contained large

Formatted: Left

Formatted: Left, Indent: First line: 0 ch

Formatted: Font color: Auto

Formatted: Font color: Auto, Not Highlight

Formatted: Font color: Auto

Formatted: Font color: Auto

Formatted: Font color: Auto

Formatted: Font color: Auto

Formatted: Font color: Auto

Formatted: Font color: Auto

Formatted: Font color: Auto

Formatted: Font color: Auto

Formatted: Font color: Auto

Formatted: Font color: Auto

Formatted: Font color: Auto

purple patches, the bracts were opened, and the plants contained a lot of glands, but not the seed embryo.

Figure 4. Growth and formation of the synthetic tetraploid, S_1. (A) Tetraploid plants with a large pink flower accompanied by yellow pollen. (B) The growth process of the oval boll. (C) Four seeds with brown fibers.

Figure 5. Pictures of cotton bolls and fibers. (A) *G. herbaceum* L. had conical, smooth green and red bolls. The bulge at the top of the boll was distinctly cone-shaped. (B) *G. nelsonii* Fryx. had oval, green cotton bolls with fine hairs on the surface. The boll tip was not obvious. (C) Green pear-shaped boll of S_1 covered with downy hairs. (D) *G. herbaceum* L. had 3-chambered bolls with gray-white spiral cotton fibers. (E) *G. nelsonii* Fryx. had 3-chambered bolls with dark brown upright fibers. (F) The S_1 had 3-chambered bolls with brown fibers, and the seed hairs grew upright.

The differences in the morphological characteristics between the S_1 plants and F_1 plants is that S_1 plants were shorter (Tables s 21, 3), had larger and thicker leaves, produced viable pollen, could form bolls, seeds, and long fibers, and had with a boll number of 50 bolls per plant (S1 S2 Fig. a) after 4 months of growth in the field. The samples were still fertile after preservation. Fertility was restored after the diploid F_1 hybrids underwent chromosome doubling. However, other S_1 traits were not different from the F_1 plants, including leaf shape and color, number of lobes, bract shape, calyx teeth shape, number of bract teeth, anther and pollen color, stem and leaf trichomes, bract extension, and extrafloral nectary(Fig. 2C1g, 21Dh).

Formatted: Font color: Auto

Formatted: Left

Formatted: Font color: Auto

Formatted: Left

Formatted: Font color: Auto

Formatted: Font color: Auto

Formatted: Font color: Auto

Formatted: Font color: Auto

Formatted: Font color: Auto

Formatted: Font: Times New Roman

Formatted: Font color: Auto

Table 3. Comparison between S_1 and –S_2 (*G. herbaceum* ~~L.~~ × *G. nelsonii*)~~Fryx. S_1, S_2~~ generations based on ~~morphology~~ morphological and fertility ~~comparison~~traits

Generation	Plant height (cm)	Colors of leaf	Leaf size (length×width, cm)	No. of lobes	No. of nectaries on the back of leaf blade	Corolla color	Corolla length (cm)	Color and size of spot	Anther and Filaments colors	Bract size (length×width, cm)	Bract shape, No. of teeth (n)	Flower nectary	Boll shape, ~~no~~No. of boll loculi	Boll size (length× width, cm)	Sterility
S_1	150.00	Dark green	5.10×5.40	0–3	1 on primary vein	Pink,	Around 2.60	Red	Yellow, Light pink	1.20×0.60	Sword-shaped, 1–3	Extrafloral	Pear-shaped, 4	Generally, 3.00×2.50	~~A lot of~~Many bolls formed
S_2	75.00–170.00	Light green, Dark green	5.70×6.20–10.90×13.60	0–3	2, 3 at the primary vein and lateral vein	Pink, Light pink, Milky white, Red flower with white border	2.40–4.40	Red, Purple, Rose, None	Yellow, Light pink, Pink interspersed with milky	1.30×0.70–1.60×1.10	Triangle, Dentate, Ensiform, 2–4	None, extrafloral, intra-floral	Pear-shaped, sunken navel, oval, irregular	2.70×2.30–3.20×2.60	Only 32 out of 145 plants had 1–7 bolls while no bolls were found in

	white	round boll, 3–4	other plants

We determined that the S_1 plants ~~are~~ were similar to the F_1 plants as their morphological traits showed classical hybrid characteristics (Fig~~s~~. 1~~, 2~~). According to the 25-item investigation data analysis (Table 3), 10 items(Fig. 1e–h) were intermediate between the parents including leaf shape and color~~(Fig. 2A–D)~~, number of lobes, bract shape, calyx teeth shape, number of bract teeth, corolla size, number of anthers, boll diameter (Fig. ~~5A~~3a–~~C~~c), fiber color, and fiber length~~,~~. ~~2~~ Two items were greater than the ~~parents~~ parents, including the number of boll loculi and fiber tensile strength (Fig. ~~5D~~3d–~~53 F~~f)~~,~~). ~~2~~ Two items were similar to the female ~~parent~~ parent, including anther and pollen color (Fig. ~~2A~~1e, ~~2~~1~~D~~h), and ~~6~~ six items were similar to the male parent including stem and leaf trichomes (Fig. ~~1B~~1b, ~~1D~~1d), flowering habit, bract extension, bract size (Fig. ~~2B~~1f, ~~2D~~1h), flower opening, and extrafloral nectary. In addition, maternal traits ~~were~~expressed as dominant for the anthers, stamens, and stigmata's characteristics (Fig. ~~2~~1~~A~~e, ~~2~~1~~D~~h)~~(Fig. 2A,D)~~, ~~while~~whereas paternal traits were ~~were dominant~~expressed in the stem and leaf trichomes, ~~and the~~ boll ~~shape~~ shape, and ~~the boll~~ trichomes (Fig. ~~5E~~3e, ~~5F~~3f).

Figure 3. Pictures of cotton bolls and fibers. (a) *G. herbaceum* had conical, smooth green and red bolls. (b) *G. nelsonii* had oval, green cotton bolls. (c) Green pear-shaped boll of S_1. (d) *G. herbaceum* had 3-chambered bolls with gray-white spiral cotton fibers. (e) *G. nelsonii* had 3-chambered bolls with dark brown upright fibers. (f) The S_1 had 3-chambered bolls with brown fibers.

SSR molecular marker determination showed that the ~~6~~six pairs of parental polymorphic primers(Fig. ~~3A~~2a) ~~not only~~ amplifi~~ied yinged~~ed the complementary bands of the ~~parents~~ parents, ~~but also~~ as well as the characteristic bands of the hybrid itself (Fig. ~~3Ac~~2a/iii, 3~~A~~a/~~v~~i~~f~~)~~.~~. This ~~which~~ indicated ~~. This shows~~ that~~ the~~ S_1 is also a true

Formatted: Font color: Auto
Formatted: Font color: Auto
Formatted: Font color: Auto
Formatted: Font color: Auto
Formatted: Font color: Auto
Formatted: Font color: Auto
Formatted: Font color: Auto
Formatted: Font color: Auto
Formatted: Font color: Auto
Formatted: Font color: Auto
Formatted: Font color: Auto
Formatted: Font color: Auto
Formatted: Font color: Auto
Formatted: Font color: Auto
Formatted: Font color: Auto
Formatted: Font color: Auto
Formatted: Font color: Auto
Formatted: Left, First line: 0 ch, Space Before: Auto, After: Auto, Don't adjust space between Asian text and numbers
Formatted: Left, Indent: First line: 2 ch
Formatted: Font color: Auto
Formatted: Font color: Auto
Formatted: Font color: Auto
Formatted
Formatted
Formatted
Formatted
Formatted
Formatted
Formatted: Font color: Auto

hybrid~~ like the F_1~~. At the same time, this shows that the external genes were not introduced during the chromosome doubling of the F_1 to obtain the S_1. Flow cytometry showed that the peak value of the control sample of *G. herbaceum* was close to 200 channels (Fig. ~~3B~~2b), the peak value of the TM-1 was close to 400 channels (Fig. ~~3C~~2c), and the peak value of the S_1 hybrid was close to 400 channels (Fig. ~~3D~~2d), showing that the S_1 hybrid was a tetraploid. Root tip staining and microscopy observations showed that the root tip chromosome number of the *G. herbaceum* and *G. nelsonii* control samples was 26 pairs (Fig. ~~3E~~2e, ~~3~~2~~F~~f)~~,~~ ~~while~~whereas the root tip chromosome number of the S_1 hybrid was 52 pairs (Fig. ~~3G~~2h~~),~~). ~~which~~ This proved that the S_1 hybrid was a tetraploid.

Examination of tetraploid S_2 segregation

The S_2 plants were completely different from the S_1 plants, and showed abnormal and intense segregation. Due to the complex genetic basis of interspecific hybrids, the segregation of F_2 populations was diverse, from male parent to female parent types, including a series of intermediate types, as well as new types of plants that are not available in parents. In the F_2 generation population, 10 representative plants were selected as observation objects, and morphological indexes were compared in the S_2 descendants of S_1 with their parental plants (Table 5). For example, ~~for plant height,~~ the shortest plant was 75.00 cm tall, which ~~is~~ was shorter than the –female parent *G. herbaceum* L.~~ female parent~~, ~~while~~whereas most ~~shrubs~~plants were tall and thick ~~shrubs,~~ with the tallest plant ~~having a height of~~being 170.00 cm. Corolla color ~~started~~began to ~~show~~ segregat~~ion~~e ~~(S2 Fig.)~~ as plants came to have not only red ~~corollas~~ corollas, ~~were produced~~ but– also pink, red ~~flowers~~ with white borders, and milky white flowers (Fig. 4a–4h) ~~–were produced~~. ~~seen but~~However~~-~~, yellow flowers

Formatted: Font color: Auto

Formatted: Font color: Auto

Formatted: Font color: Auto

Formatted: Font color: Auto

Formatted: Font color: Auto

Formatted: Left

Formatted: Font color: Auto

Formatted: Left, Indent: First line: 0 ch

Formatted: Font color: Auto

Formatted: Font color: Auto

Formatted: Font color: Auto, Subscript

Formatted: Font color: Auto

Formatted: Font color: Auto, Subscript

Formatted: Font color: Auto

Formatted: Font color: Auto

Formatted: Font color: Auto

Formatted: Font color: Auto

Formatted: Font color: Auto

Formatted: Font color: Auto

Formatted: Font color: Auto

Formatted: Font color: Auto

~~of the female parent~~ were not ~~seen~~observed on the female parent (Fig. ~~2A~~1e). The leaves were generally large, ~~and~~with the longest leaf ~~length~~ being ~~8~~ 9.90 cm and ~~leaf width being~~ the widest leaves being 11.00 cm. Nectaries at the back of the leaves were also unique, as they were different from normal leaves that had one nectary at the base of the primary vein, but both primary and lateral veins contained nectaries. Some leaves had ~~2~~two~~-~~, ~~while~~whereas other leaves ~~have~~ had ~~3~~three nectaries (Table ~~3~~3), which are traits rarely observed~~. This multiple nectary trait in the leaves is rarely seen~~ in the *Gossypium* genus.

Figure 4. Flowers of the tetraploid S$_2$. (a) Pink, cylindrical flower with pink corolla, (b) funnel-shaped flower with light pink corolla, (c) funnel-shaped flower with white and pink alternating corolla, (d) white, closed flower, (e) bell-shaped flower with red corolla and a clear rose-colored spot on the petal base, (f) semi-closed flower with pink corolla, (g) bell-shaped flower with pale pink corolla. (h) small flower with white corolla and no spot on the petal base.

Observations also showed that the S$_2$ morphology was associated with fertility. Plants that resembled a pagoda like the female parent ~~(*G. herbaceum* L.)~~ produced more bolls and seeds, with 5–10 seeds per boll. In contrast, plants that resembled the male parent ~~(*G. nelsonii* Fryx.)~~ were inclined to have a higher ratio of sterility and few fertile plants that produced fewer bolls, with each boll containing only a few seeds (1–5).

Fertility was also found to be highly segregated, with some weaker plants growing slowly and flowering later with fewer flowers~~,~~. ~~but~~ They were still normally ~~being~~ able to disperse pollen and fruit, even though the fruits were small and not upright. Other plants grew more readily, flowering earlier with more flowers and fruits, and

Formatted: Font color: Auto

Formatted: Font color: Auto

Formatted: Font color: Auto

Formatted: Font color: Auto

Formatted: Font color: Auto

Formatted: Font color: Auto

Formatted: Font color: Auto

Formatted: Font color: Auto

Formatted: Font color: Auto

Formatted: Font color: Auto, Not Highlight

Formatted: Left, First line: 0 ch, Space Before: Auto, After: Auto, Don't allow hanging punctuation, Don't adjust space between Asian text and numbers

Formatted: Not Highlight

Formatted: Left

Formatted: Font color: Auto

Formatted: Font color: Auto

Formatted: Font color: Auto

Formatted: Font color: Auto

Formatted: Font color: Auto

the fruits were large and upright. Most fibers produced fibers were brown, but some bolls contained fibers that were both brown and light green (S32 Fig. Ab, Bd). Intense segregation also occurred for fertility. Some plants grew slowly and were weaker, flowered later, and had fewer flowers, but could normally disperse pollen and fruit, but the fruits were small and not upright. Other plants showed exuberant growth, flowered earlier, and produced more flowers and fruits, and the fruits were large and upright. Overall, the fibers produced were brown in color, but some bolls contained fibers with two different colors, namely brown and light green fibers (S3 Fig). We found that a variety of colored fibers developed in the S_2 cotton bolls, including brown fibers in a 4-chambered boll and light green and brown cotton fibers in a 3-chambered boll (S3 Fig. A,B). Notably, the fiber Micronaire reading of the S_2 fibers was 9.6, determined using the Macron Airflow Meter in the fiber-testing center of the Cotton Research Institute of Chinese Academy of Agricultural Sciences. This reading was far above the parental range and may be the highest value reported to date. The S_2 fibers were very thick and provided us with the raw materials for making non-woven fabrics(S3 Fig. C). These observations indicated that the fiber characteristics of S_2 were recessive and dynamic.

The above results indicate that the S_2 generation underwent intense segregation in both morphological characteristics and fertility. This phenomenon exists in varying degrees in other interspecific hybrids that we generated and is common in segregated generations. However, it was particularly prominent here in the S_2 generation of this hybrid. Table 3 compares the observation data of the S_2 and S_1 plants. It should be mentioned that the S_2 was used as an experimental material asbecause it was a population with genetic segregation and with a rich genetic diversity. It is also an

Formatted: Font color: Auto

Formatted: Left

Formatted: Font color: Auto

Formatted: Font color: Auto

ideal population for the screening of disease-resistant varieties using conventional hybridization and breeding methods.

Formatted: Font color: Auto

Formatted: Font color: Auto

Table 4. Comparison of quantifiable parameters in the S_2 descendants of S_1 with their parental plants

Plants	Plant height (cm)	Internode of stem (cm)	Vegetative branches	Fruiting branches	Leaf size (length×width, cm)	Bract size (length×width, cm)	Boll size (length×width, cm)	Corolla length (cm)	Stigma length (cm)	Length of fiber (mm)
S_2-1	156.68	5.18	5	13	8.26×9.76	1.58×1.10	3.11×2.56	4.35	1.14	12.86
S_2-2	170.00	5.21	7	15	9.23×10.81	1.60×1.10	3.26×2.65	4.46	1.15	11.12
S_2-3	146.23	5.06	5	14	9.51×10.72	1.50×0.98	2.89×2.67	4.14	1.22	10.46
S_2-4	138.17	4.89	6	15	8.62×9.92	1.30×0.89	3.05×2.38	3.95	1.21	12.98
S_2-5	129.28	5.07	5	12	9.81×10.61	1.25×0.76	2.97×2.56	4.28	1.36	10.56
S_2-6	89.86	4.86	4	9	6.33×7.50	1.38×0.72	2.84×2.80	2.51	1.28	9.78
S_2-7	70.00	3.16	4	7	5.71×6.20	1.3×0.72	2.78×2.36	2.46	0.95	8.23
S_2-8	98.56	3.38	4	8	8.21×9.80	1.35×0.72	2.75×2.58	2.67	0.89	7.46
S_2-9	158.46	4.78	5	42	8.63×10.20	1.35×0.68	3.08×2.59	4.39	1.16	10.95
S_2-10	169.76	4.86	5	15	9.90×11.00	1.59×1.02	3.19×2.98	4.34	1.35	10.96
G.herbaceum	80.00	3.45	4	7	5.21×6.18	1.10×1.32	3.56×2.87	2.18	0.85	17.70
G.nelsonii	200.00	4.96	7	15	12.86×13.98	1.2×0.25	2.31×2.06	3.53	1.48	5.60

Determination of disease resistance in the tetraploid S_2

~~**Table 4. Hybridization characteristics of TM-1×(*G. herbaceum* L. × *G. nelsonii* Fryx.S₁) hybridization results and fertility examination**~~

~~No. of flowers pollinated~~	~~Effective boll number~~	~~Boll-forming rate (%)~~	~~No. of normal embryos~~	~~Number of three-way F_1 plants~~	~~Number of three-way F_1 low-fertility plants~~
~~278~~	~~16~~	~~5.75~~	~~9~~	~~7~~	~~2~~

~~**Determination of disease resistance in the tetraploid S_2**~~

For each plant, there were 108 nutrient pots, and each pot contained ~~5~~ five seedlings. After the first true leaf was level, the plants were inoculated with a fixed concentration of *Verticillium dahliae* Kleb. suspension (Fig. ~~6A~~5a). After 20 days the percentage of infected plants in the control group was 80 %. Results (Table 6) showed that when the inoculation was carried out indoors, the seedlings developed disease at the cotyledon stage. There were 456 plants from the susceptible strain cv. *Jimian11* that developed disease, with an incidence of 84.44 % and disease index of ~~33~~ 44.00 (Fig. ~~6B~~5b). The resistant grades in S_2 descendants of S_1 were considered to be immune or highly resistant (Fig. 5c). The incidence and disease indexes of *G. nelsonii* were zero, and the resistance was classified as immune (Fig. 5d). The disease index of *G. herbaceum* was 1.68 (Fig. 5e). ~~The incidence and disease indices of the S_2, *G. herbaceum* and *G. nelsonii* were all 0 and they were considered to be immune (Fig. 6C–6 E).~~ No disease occurred in any of the plants inoculated with sterile water. From

- **Formatted:** Numbering: Continuous
- **Formatted:** Font: 16 pt, Bold
- **Formatted:** Font: Not Bold
- **Formatted** ...
- **Formatted:** Font: Times New Roman, 9 pt
- **Formatted:** Left
- **Formatted:** Font: Times New Roman, 9 pt
- **Formatted:** Left
- **Formatted:** Left, Indent: First line: 0 ch
- **Formatted:** Left
- **Formatted** ...
- **Formatted:** Font color: Auto
- **Formatted:** Font color: Auto
- **Formatted:** Font: Not Bold, Font color: Auto
- **Formatted:** Font color: Auto
- **Formatted:** Font color: Auto
- **Formatted:** Font: Not Bold, Font color: Auto
- **Formatted:** Font color: Auto
- **Formatted:** Font: Not Bold, Font color: Auto
- **Formatted:** Font color: Auto, Not Highlight
- **Formatted:** Font color: Auto
- **Formatted:** Font color: Auto, Not Highlight
- **Formatted:** Font color: Auto
- **Formatted:** Font color: Auto, Not Highlight
- **Formatted:** Font color: Auto, Not Highlight
- **Formatted:** Font color: Auto
- **Formatted:** Font color: Auto, Not Highlight
- **Formatted:** Font color: Auto
- **Formatted:** Font color: Auto, Not Highlight
- **Formatted:** Font color: Auto
- **Formatted:** Font color: Auto, Not Highlight
- **Formatted** ...
- **Formatted:** Font color: Auto
- **Formatted:** Font color: Auto, Not Highlight
- **Formatted:** Font color: Auto
- **Formatted:** Font color: Auto, Not Highlight
- **Formatted:** Font color: Auto
- **Formatted:** Font color: Auto, Not Highlight
- **Formatted:** Font color: Auto, Not Highlight
- **Formatted:** Font color: Auto
- **Formatted:** Font color: Auto, Not Highlight
- **Formatted:** Font color: Auto
- **Formatted:** Font color: Auto, Not Highlight
- **Formatted:** Font color: Auto, Not Highlight

this, it was preliminarily determined that the tetraploid S_2 was germplasm material with potential disease resistance. In future stud~~y plans~~ies, we will examine and validate the disease resistance in the S_2 in specific plots in the field, to provide reliable intermediate materials for the cultivation of ~~disease~~ disease-resistant *G. hirsutum* L.

Table 5. Identification of cotton Verticillium wilt resistance in the S_2 descendants of S_1 with their parental plants.

Cultivar	Disease index	Relative resistance index	Classification
Jimian11	44.00±3.62	22.00±1.81	S
S_2-1	0.00	0.00	I
S_2-2	0.00	0.00	I
S_2-3	0.28±0.68	0.14±0.34	HR
S_2-4	0.00	0.00	I
S_2-5	0.00	0.00	I
S_2-6	0.56±0.12	0.28±0.06	HR
S_2-7	0.00	0.00	I
S_2-8	0.00	0.00	I
S_2-9	0.16±2.86	0.8±1.43	HR
S_2-10	0.00	0.00	I
G. nelsonii Fryx.	0.00	0.00	I
G. herbaceum L.	1.68±1.64	0.84±0.82	HR

Figure ~~6~~5. Identification of resistance to *Verticillium dahliae* Kleb.~~.~~ (~~A~~a) The pathogen of the ~~candida~~ conidia is indicated with the black arrow. (~~B~~b) Disease-susceptible upland cotton *Jimian11*, with yellowing, necrosis, and leaf abscission indicated by red arrows. (~~C~~c) The resistant S_1. (~~D~~d) Resistant *G. nelsonii*. (~~E~~e) Resistant *G. herbaceum*.

~~**Figure 6.** Identification of resistance to *Verticillium dahliae* Kleb. (A) The pathogen of the candida conidia is indicated with the black arrow. (B) Disease-susceptible upland cotton *Jimian11*, with disease symptoms of yellowing, necrosis, and leaf abscission indicated by red arrows. (C) The resistant S_1. (D) Resistant *G. nelsonii* Fryx. (E) Resistant *G. herbaceum* L.~~

Description of the S_1 synthetic tetraploid hybrid based on morphological and fertility traits

~~Tetraploid S_1 breeding application test~~

From an analysis of the effective boll number and boll-forming rate (Table ~~4~~6), we determined that the tetraploid S_1 showed some compatibility for hybridization with the TM-1~~ *G. hirsutum* L. plants~~. In 2018, the S_1 was hybridized with the TM-1~~ *G. hirsutum* L.~~ plants and 16 effective bolls were obtained, but they were small and deformed (S3 Fig. ~~7A~~a ~~7 D~~d). These grew into seven three-way F_1 plants, of which only ~~2~~ two grew normally and were fertile (Fig. ~~8A~~6a ~~86 D~~d). The remaining ~~5~~ five plants did not grow normally~~ (~~; some grew to the flowering stage and growth stopped, some died, some were infertile, and some had low fertility~~)~~. This is an inevitable ~~reason~~consequence ~~from~~ of three-way species crossing between *G.*~~TM-1~~ *hirsutum* L.

Formatted: First line: 0 ch, Space Before: Auto, After: Auto
Formatted: Font color: Auto
Formatted: Font color: Auto
Formatted: Left
Formatted: Font color: Auto
Formatted: Font color: Auto
Formatted: Font color: Auto
Formatted: Font color: Auto
Formatted: Font color: Auto
Formatted: Font color: Auto
Formatted: Font color: Auto
Formatted: Font color: Auto
Formatted: Font: Italic, Font color: Auto
Formatted: Font color: Auto

× (*G. herbaceum* L. × *G. nelsonii* Fryx., S_1). Hence, the 5 five three-way F_1 hybrids cannot be used for selective breeding.

Table 6. Hybridization characteristics of TM-1× (*G. herbaceum* × *G. nelsonii* S_1)

No. of flowers pollinated	Effective boll number	Boll-forming rate (%)	No. of normal embryos	No. of three-way F_1 plants	No. of three-way F_1 low-fertility plants
278	16	5.75	9	7	2

Figure 7. Cotton bolls from cottonof TM-1 and from the F_1 hybrid of from TM-1 × S_1 tetraploid. (A) The normal conical boll of TM-1 shown by the red arrow. (B) The abnormally conical boll of TM-1 × S_1 F_1 plants on the right(B) and on the left(C), indicated by yellow arrows. (D) The deformed hybrid boll of F_1 plants indicated by yellow arrows.

Figure 8. Cotton plants of TM-1, the S_1 tetraploid and, F_1-1, F_1-2 plants. (A) Cotton TM-1 was tTower-shaped TM-1like with longer and looser branches. (B) S_1 was towerTower-shaped S_1 .like with shorter and more compact branches. (C) Hybrid F_1-1 was tower-like with shorter and looser branches and pink flowers (shown by red arrow). (D) Hybrid F_1-2 was tower-like with longer and looser branches and had white flowers shown by (white arrow).

The plants described above, that grew normally and were fertile, were transplanted to fields and named F_1-1 and F_1-2 (Fig. 86Cc, 86Dd).The two three-way F_1 plants described above, that grew normally and were fertile, were transplanted to fields and named F_1-1 and F_1-2 (Fig. 8C,D). Field morphological observations found that the F_1-

Formatted: Font color: Auto

Formatted: Font color: Auto

Formatted: Font color: Auto

Formatted: Left, Indent: First line: 0 ch

Formatted: Font color: Auto

1 plant retained significant wild cotton characteristics, which were mostly paternal traits(Fig. ~~9B~~6f, ~~96C~~g). The morphology of the F_1-2 was an intermediate type, having the most important traits from *G. hirsutum* L. (Fig. ~~96A~~e, ~~96D~~h)~~intermediate *G. hirsutum* L. and had mostly inherited material traits(Fig. 9A,D)~~. The significant differences between the two plants ~~was~~ were the corolla color, presence of spots, bract characteristics, trichomes, and boll fertility. The F_1-1 plant had a pink-colored corolla and red spots, bracts that were separate and not jointed (Fig. ~~9C~~6g)~~)~~, and the stems, branches, leaves, and bolls had a large number of trichomes. The boll number was ~~low~~ low, and the bolls were deformed (Fig. ~~9G~~6k)~~)~~. Furthermore, ~~T~~the ~~seed~~ embryos tested from the seeds were very small and ~~not~~ infertile. The F_1-2 plant had a milky white corolla~~,~~; no spots were present, and the bracts ~~that~~ were jointed (Fig. ~~9D~~9h)~~)~~. The surfaces of the stems, branches, leaves, and bolls were smooth and there were no trichomes. The bolls developed into a funnel shape and the seed embryos were large and fertile (Fig. ~~9H~~6l)~~)~~.

Figure 6. The plants, leaves, flowers, corollas, basal patches, filaments, anthers, pollen, calyxes, bracts, and bolls about TM-1, S_1 and their offprings. TM-1(a, e), S_1(b, f), F_1-1(c, g), F_1-2(d, h). (i) normal conical boll of cotton TM-1, (j) green pear-shaped boll of S_1, (k) downy green boll of F_1-1 that was deformed, indicated by the red arrow, and (l) normal conical boll of F_1-2.

~~**Figure 9.** Pictures of leaves, flowers, ,corollas, basal patches,filaments,anthers,pollen,calyxes,bracts of cotton TM-1(A),S_1(B), F_1-1(C)and F_1-2(D) . and bolls of cotton TM-1, the S_1 tetraploid, and of F_1-1 and F_1-2 plants. (A)~~

Formatted: Font color: Auto

Formatted: Font color: Auto

Formatted: Font color: Auto

Formatted: Font color: Auto

Formatted: Font color: Auto

Formatted: Font color: Auto

Formatted: Font color: Auto

Formatted: Font color: Auto

Formatted: Font color: Auto

Formatted: Font color: Auto

Formatted: Font color: Auto

Formatted: Font color: Auto

Formatted: Font color: Auto

Formatted: Font color: Auto

Formatted: Left, Indent: First line: 0 ch, Space Before: Auto, After: Auto, Don't adjust space between Asian text and numbers

Formatted: Left

Cotton TM-1 bore bell-shaped flowers with milky corollas, milky filaments, milky anthers and pollen, without basal patches, wavy calyxes, heart-shaped bracts grown together, and were broad-leaved. (B) S_1 had bell-shaped flowers with pink corollas, milky filaments, yellow anthers and pollen, red basal patches, serrated calyxes, sword-shaped bracts grown separately rather than together, and had heart-shaped or broad leaves. (C) Hybrid F_1-1 had bell-shaped flowers with pink corollas, milky filaments, yellow anthers and pollen, red basal patches, serrated calyxes, heart-shaped bracts grown separately rather than together, and broad leaves. (D) Hybrid F_1-2 bore bell-shaped flowers which were milky white in the middle with a light red corolla border, milky filaments, milky anthers and pollen, without basal patches, wavy calyxes, heart-shaped bracts grown together, and broad leaves. (E) A normal conical boll of cotton TM-1. (F) A green pear-shaped boll of S_1 with a sunken navel. (G) A downy green boll of F_1-1 that was deformed, indicated by the red arrow. (H) A normal conical boll of F_1-2.

Formatted: Font color: Auto

The tetraploid S_1 had some potential for breeding applications, but there was severe segregation in its progeny and its fertility was decreased, which affects further breeding applications. Notably, as a limitation, we of our study is that we have only preliminarily assessed the authenticity of the F_1 generation obtained from the crosses between the TM-1 and S_1 through morphological characterization. Further studies using cytology and molecular genetics techniques are required to confirm that they are true hybrids using cytology and molecular genetics techniques.

Formatted: Font color: Auto

Formatted: Font color: Auto

Discussion

Wild cotton varieties have undergone long-term natural selection to accumulate rich genetic diversity conferring adaptability to adverse conditions and resistance to environmental onslaughts and –diseases. They provide a great treasury of traits that could be utilized to improve commercial cultivars [275],which. There are at least 30 varieties of wild cotton, widely distributed in the Americas, Australia, Africa, and Asia that have undergone long-term natural selection to accumulate rich genetic diversity conferring adaptability to adverse conditions and resistance to environmental onslaughts and diseases. The cultivated varieties present today have also undergone a long period of artificial selection compared with wild varieties to significantly improve yields and fiber quality. However, even after have undergone long-term intensive farming and artificialnatural selection to accumulate rich genetic diversity conferring adaptability to adverse conditions and resistance to environmental onslaughts and diseases., , some favorable resistant phenotypes have not developed in cultivated varieties and were even excluded in some situations by excessive selection. Therefore, despite Despite the continuous improvements in yields and fiber quality, the disease resistance of commercial cotton cultivars, the disease resistance has weakened. Resistance breeding programs are now being conducted to transfer the disease resistance genes from wild cotton to cultivated cotton, and to re-diversify the genetic makeup of the cultivated cotton.

Although tTThe superior qualities quality of cotton fiber, including its length, strength, fineness as well asand the disease resistance of wild cotton , hasve been successfully transferred to cultivated cotton [28278, 292829].]; However, diploid wild cotton and tetraploid cultivated cotton are biologically isolated. While increased genetic diversity could be achieved from distant hybridization, tThey are often genetically incompatible, forming a barrier to the full utilization of wild diploid cotton

Formatted: Font color: Auto

Formatted: Indent: First line: 0"

Formatted: Font color: Auto

Formatted: Font color: Auto

Formatted: Font color: Auto

Formatted: Font: 10.5 pt

Formatted: Font color: Auto

Formatted: Font color: Auto, Not Highlight

Formatted: Font color: Auto, Not Highlight

Formatted: Font color: Auto, Not Highlight

Formatted: Font color: Auto, Not Highlight

Formatted: Font color: Auto

Formatted: Font color: Auto, Not Highlight

Formatted: Font color: Auto

Formatted: Font color: Auto

varieties [~~30~~~~29~~30 ~~–~~3~~2~~2]. We have already obtained an interspecific hybrid F_1 of *G. herbaceum* and *G. raimondii*. The F_1 plants are sterile in the field, leading to various methods being attempted to double their chromosome but without success [~~24~~21]. In ~~the present~~~~this~~the present study, we successfully obtained an infertile interspecific F_1 hybrid ~~through distant hybridization,~~ and managed to ~~overcoming~~ overcome the genetic incompatibility barriers between two distantly related species. Furthermore, ~~We~~ we ~~further~~ doubled the chromosomal content of this F_1 hybrid ~~with~~ through colchicine ~~treatments to~~immersion, ~~overcome~~ overcoming –the ~~significant~~ considerable challenges of interspecific hybrid sterility, to successfully synthesize a new S_1 tetraploid cotton species ~~S_1~~.

~~We found that the S_1 progeny in the field showed a phenomenon of gradual decline in growth. The morphological traits of the S_1 plants were generally consistent, with normal growth, flowering, and fruiting. However, the S_2 plants showed violent segregation and resulted in different types of plant shapes and corolla shapes, and huge differences in the flowering and fruiting characteristics. The growth of many plants in the S_3 to S_5 generations were suppressed and only a few plants could flower. In severe cases, the plants could not flower and only a small number of plants could bear fruit. This shows that the S_1 was genetically unstable germplasm and there is still a risk of natural segregation. The genetic instability of the S_1 severely impedes its use in breeding. This problem will prevail until the new germplasm obtained from hybridization cannot be used to achieve the aim of hybridization. Therefore, in future studies, there is a need to control segregation in hybrids and establish new genetic isolates. The genetic instability of the S_1 severely hindered the utilization of the new cotton germplasm for breeding. We were unable to resolve this issue in the present study; thus, the control of hybrid separation for the successful establishment of stable~~

Formatted: Font color: Auto

Formatted: Font color: Auto

Formatted: Font color: Auto

Formatted: Font color: Auto

Formatted: Font color: Auto, Not Highlight

Formatted: Font color: Auto

Formatted: Font color: Auto

Formatted: Font color: Auto

Formatted: Font color: Auto

Formatted: Font: 10.5 pt

Formatted: Font color: Auto

genetic strains needs to be explored in future research. It should be mentioned that the S_2 was used as an experimental material as it was a population with genetic segregation and with a rich genetic diversity. It is also an ideal population for the screening of disease resistant varieties in conventional hybridization and breeding. Furthermore, the number of S_1 plants obtained was low and does not facilitate the execution of this experiment.

Our results support the hypothesis that distant hybridization is an important driver of new mutations and species in plant evolution [333, 344]. Molecular markers are specific pieces of DNA that reflect differences in the genomes of individuals or populations [33]. Since the discovery that microsatellite markers are uniformly distributed on cotton chromosomes, SSR markers have been used to monitor the infiltration of heterologous chromosome fragments in the offspring of interspecific hybrids [34355, 36,3536]. In the present study, SSR detection revealed the presence of new specific bands in the hybrids_ (Fig. 3Ac,f) that were not detected in the parents, indicating that new combinations or mutations of chromosomes are present in the interspecific progeny (Genomic A and Genomic genomic C). This result might be due to chromosomal rearrangements in this newly formed hybrid [3737], which was consistent with thethose reported in previous reportsstudies on trispecific hybrids [220]. Our results also support the hypothesis that distant hybridization is an important driver of new mutations and species in plant evolution [36,37].

There is potential for genes from the new synthetic allotetraploids to fuse with favorable genes from *G. herbaceum* L. and *G. nelsonii* Fryx., including genes that encode good fiber quality, or resistance to diseases., Such as pests, and drought. tThe number of bolls per S_1 plant reached 50 (S2 Fig. a) bolls, an important

Formatted: Space Before: Auto, After: Auto

Formatted: Font color: Auto

Formatted: Font: Times New Roman

Formatted: Font color: Auto

Formatted: Font: Times New Roman

Formatted: Font color: Auto

Formatted: Font color: Auto

Formatted: Font color: Auto

Formatted: Font color: Auto, Not Highlight

Formatted: Font color: Auto

Formatted: Font color: Auto

Formatted: Font color: Auto

Formatted: Font color: Auto

Formatted: Font color: Auto

Formatted: Not Highlight

Formatted: Font color: Auto

Formatted: Font color: Auto

Formatted: Font color: Auto

Formatted: Indent: First line: 0.42", Space Before: Auto, After: Auto

Formatted: Font: Times New Roman

Formatted: Font color: Auto

Formatted: Font color: Auto

Formatted: Font color: Auto

Formatted: Font color: Auto

Formatted: Font color: Auto

characteristic for high yield breeding applications~~(S1 Fig.)~~. We found that a variety of colo~~u~~red fib~~re~~ers developed in S_2 cotton bolls, including brown fib~~re~~ers in a 4-chambered boll and light green and brown cotton fib~~re~~ers in a 3-chambered boll (S~~3~~2 Fig. ~~A~~b, ~~B~~d). The preliminary examination showed that the disease resistance of the tetraploid S_2 was better than *G. herbaceum* and the same level as *G. nelsonii*.~~-~~ Increased disease resistance also occurs in interspecific hybrids between *G. hirsutum* × *G. ~~arboreum~~arboretum* [~~38~~19]. This important agronomic characteristic provided a new and important resource for cotton disease resistance breeding programs.

~~This will allow their transfer to upland cotton through hybridization, to further improve the resistance and fiber quality of the upland cotton [22]. We tested this possibility by performing preliminary disease resistance testing of the new S_2 species in the greenhouse. We found that the new species presented high disease resistance to the fungal pathogen *Verticillium dahliae* Kleb. This important agronomic characteristic provided a new and important resource for cotton disease resistance breeding programs.~~

At the same time, there is a need to control segregation in hybrids and establish new genetic isolates. We found that the S_1 progeny in the field showed a phenomenon of gradual decline in growth. The morphological traits of the S_1 plants were generally consistent, with normal growth, flowering, and fruiting. However, the S_2 plants showed ~~violent~~dramatic segregation and resulted in different types of plant and corolla shapes, and huge differences in the flowering and fruiting characteristics. The growth of many plants in the S_3 to S_5 generations w~~ere~~as suppressed and only a few plants could flower. In severe cases, the plants could not flower and only a small number of plants could bear fruit. This shows that ~~the~~ S_1 was genetically unstable

Formatted: Font color: Auto

Formatted: Font color: Auto

Formatted: Font color: Auto

Formatted: Font: Times New Roman

Formatted: Font color: Auto

Formatted: Font color: Auto

Formatted: Font color: Auto

Formatted: Font color: Auto

Formatted: Not Highlight

Formatted: Font color: Auto

Formatted: Not Highlight

Formatted: Indent: First line: 0", Line spacing: 1.5 lines

Formatted: Font: Times New Roman

Formatted: Indent: First line: 0.42", Space Before: Auto, After: Auto

Formatted: Font color: Auto

Formatted: Font color: Auto

germplasm and there is still a risk of natural segregation. The decline ~~of~~in ~~the~~ growth potential ~~of~~for hybrid offspring is a common phenomenon in cotton distant hybridization[7,13,15].

To determine the breeding potential of the S_1 population, the hybridization of S_1 with the TM-1 upland cotton germline yielded two different types of hybrid F_1, ~~When we examined whether the newly synthesized allotetraploid S_1 could be applied in cotton breeding via hybridization with upland cotton TM-1, we found two types of plants in the F_1 hybrid offspring,~~ namely F_1-1 with red flowers and F_1-2 with white flowers, ~~. The F_1-1 plants died upon the onset of anthesis,~~ indicating that the different genotypes of parents lead to the diversity and complexity of their hybrid offspring in the process of genetic recombination. The F_1-1 plants died ~~upon~~at the onset of anthesis, indicating that this plant type could not be utilized in crossbreeding. The sterility of F_1-1 hybrid plants was consistent with the results of interspecific hybrids of *G. hirsutum* L. and *G. tomentosum* Nutt.[38], which may be caused by the incoordination of physiological processes in the hybrid offspring. ~~.~~The F_1-2 plants grew normally until flowering and fruiting, but their seeds could not germinate normally~~, . indicating that this type had the potential for breeding applications, but additional research is required to overcome the key difficulties in promoting seed germination in each generation.~~ This phenomenon of unsuccessful germination of F_1-2 seeds and root tip wilting had previously been observed during the early generations of the amphiploid *G. arboretum* L. ~~–~~hybridized with *G. bickii* Prokh. [~~38~~~~39~~12]. Additional research is required to overcome the key difficulties in promoting seed germination in each generation. The variation in hybrid phenotypes may be related to gene overlap, new genetic variation, chromosome rearrangement, or a variety of other factors [~~39~~~~40~~39–~~41~~~~42~~40].

Formatted: Font color: Auto
Formatted: Not Highlight
Formatted: Font color: Auto
Formatted: Font: SimSun
Formatted: Font: Times New Roman
Formatted: Font color: Auto
Formatted: Font: Times New Roman
Formatted: Font color: Auto
Formatted: Font color: Auto
Formatted: Font color: Auto, Subscript
Formatted: Font color: Auto
Formatted: Font color: Auto
Formatted: Font color: Auto
Formatted: Font color: Auto
Formatted: Font: Times New Roman
Formatted: Font color: Auto
Formatted: Font color: Auto
Formatted: Font color: Auto
Formatted: Font: 10.5 pt, Font color: Auto

The tetraploid S_1 is a new germplasm obtained by chromosome doubling of the diploid F_1 hybrid and is a polyploidy event in cotton. This not only restoresd fertility to sterile diploid F_1 hybrids, and the tetraploid S_1 generation obtained showed segregation, which increases the phenotypic mutations in the species, enabling breeders to select for required traits. After the polyploid was generated, not only did its stress resistance not only did not decrease decrease, but biotic stress resistance was maintained. For example, the preliminary examination showed that the disease resistance of the tetraploid S_2 was 1 grade better than the female parent (*G. herbaceum*) and the same level as the male parent (*G. nelsonii*), and its disease resistance inheritance was closer to the male parent. In addition, the fiber quality of the tetraploid S_2 exceeded that of both parents. The S_2 generation had high potential for breeding owing to its boll-forming properties, diversity of fiber colors, and fiber quality. These traits are also of great value for breeding programs. From this we can see that diploid F_1 hybrid polyploidy has important significance for cotton evolution,; as the genome structure changed ,and, the species genetic diversity was enriched, and species adaptability enriched as well asand the species adaptability and fiber quality were was improved.

Thus far, we have only performed preliminary characterizations of the newly synthesized allotetraploid cotton S_1.We have proposed to performing more in-depth and comprehensive research to extract the valuable information from this new cotton tetraploid S_1, so as to provide a more accurate theoretical basis for cotton breeding and genetic evolution.

Formatted: Left, Indent: First line: 0 ch
Formatted: Font color: Auto
Formatted: Font color: Auto
Formatted: Font color: Auto
Formatted: Font color: Auto
Formatted: Font color: Auto
Formatted: Font color: Auto
Formatted: Font color: Auto
Formatted: Font color: Auto
Formatted: Font color: Auto
Formatted: Font color: Auto
Formatted: Font color: Auto
Formatted: Indent: First line: 0.42", Space Before: Auto, After: Auto
Formatted: Font color: Auto
Formatted: Font color: Auto
Formatted: Font: 10.5 pt
Formatted: Left

Acknowledgements

We are grateful to Dr Kunbo Wang and Dr Fang Liu of the Cotton Research Institute of Chinese Academy of Agricultural Sciences for kindly providing us with the pollen of *G. nelsonii* Fryx. to use at the National Wild Cotton Nursery, Sanya, China.

We would like to thank Editage (www.editage.cn) for English language editing.

Formatted: Font color: Auto

Formatted: Space Before: Auto, After: Auto, Line spacing: Double

Formatted: Left, Space Before: Auto, After: Auto, Don't adjust space between Asian text and numbers

Formatted: Space Before: Auto, After: Auto, Line spacing: Double

References

1. Fryxell P.A. ~~Fryxell~~. A revised taxonomic interpretation of *Gossypium* L.(Malvaceae). Rheedea. 1992; 2(2):108-165.
2. Wang K~~unbo~~B, Wendel J~~onathan F.~~, Hua J~~inping~~P. Designations for individual genomes and chromosomes in Gossypium. J Cotton Res. 2018; 1:3. https://doi.org/~~doi:~~10.1186/s42397-018-0002-1.
3. Wendel J~~onathan F.~~, Brubaker~~,~~ C~~urt~~ L., Edward P~~ercival,~~ A. ~~Edward.~~Genetic diversity in *Gossypium hirsutum* and the origin of upland cotton. American Journal of Botany. 1992; 79:1291–1310. https://doi.org/10.1002/j.1537-2197.1992.tb13734.x
4. Iqbal M.J. ~~Iqbal~~, Reddy O.U.K. ~~Reddy~~, El-Zik K.M. ~~El-Zik~~ & Pepper A.E. ~~Pepper~~. A genetic bottleneck in the 'evolution under domestication' of upland cotton *Gossypium hirsutum* L. examined using DNA fingerprinting. Theoretical and Applied Genetics. 2001; 103:547–554. https://doi.org/10.1007/PL00002908
5. Campbell B, Saha S, Percy R, Frelichowski J, Jenkins J, Park W, et al. Status of the global cotton germplasm resources. Crop Science. 2010; 50:1161–1179. https://doi.org/10.2135/cropsci2009.09.0551er
6. David D~~. Fang~~, Hinze L~~ori L. Hinze~~, Percy R~~ichard~~ G~~. Percy~~, Li P~~ing Li~~, Deng D~~ewayne Deng~~ & Thyssen G~~regory Thyssen~~. A microsatellite-based genome-wide analysis of genetic diversity and linkage disequilibrium in Upland cotton (*Gossypium hirsutum* L.) cultivars from major cotton-growing countries. Euphytica. 2013; 191:391–401. ~~6.~~ https://doi.org/10.1007/s10681-013-0886-2

Formatted: Left, Line spacing: Double, Numbered + Level: 1 + Numbering Style: 1, 2, 3, … + Start at: 1 + Alignment: Left + Aligned at: 0" + Indent at: 0.29"

Formatted: Font color: Auto

Formatted: Font color: Auto

Formatted: Font color: Auto

Formatted: Font color: Auto

Formatted: Font color: Auto

Formatted: Font: (Default) Times New Roman, 12 pt, Font color: Auto

Formatted: Font color: Auto

Formatted: Font: (Default) Times New Roman, 12 pt, Font color: Auto

Formatted: Font: (Default) Times New Roman, 12 pt, Font color: Auto

Formatted: Font: (Default) Times New Roman, 12 pt, Font color: Auto

Formatted: Font color: Auto

Formatted: Font: 12 pt

Formatted: Font: (Default) Times New Roman, 12 pt, Font color: Auto

Formatted: Font: Not Italic

Formatted: Font color: Auto

Formatted: Font color: Auto

Formatted: Font: (Default) Times New Roman, 12 pt, Font color: Auto

7. Wendel J~~onathan~~ F ~~Wendel~~, Cronn R~~ichard~~ C ~~Cronn~~. Polyploidy and the evolutionary history of cotton. Advances in Agronomy. 2003~~.~~; 78~~,~~:139–186.
~~7.~~ https://doi.org/10.1016/S0065-2113(02)78004-8

8. Hutchinson JB, Silow RA, Stephens SG. The evolution of *Gossypium* and the differentiation of the cultivated cottons. The Quarterly Review of Biology. 1949; 24(2):143–144.

9. Satya Narayan Jena, Anukool Srivastava, Krishan Mohan Rai, Alok Ranjan, Sunil K~~.~~ Singh, Tarannum Nisar, et al. Development and characterization of genomic and expressed SSRs for levant cotton (*Gossypium herbaceum* L.). Theoretical and Applied Genetics. –2012; 124:~~-~~565–576.
https://doi.org/10.1007/s00122-011-1729-y

10. Endrizzi J~~.~~E. ~~Endrizzi, ,~~ Phillips L~~.~~L. ~~Phillips~~. A hybrid between *Gossypium arboreum* L. and *G.raimondii* Ulb. Can J Genet Cytol. 1960; 2(4~~),~~):311–319.
https://doi.org/10.1139/g60-032

11. Muramoto H~~. Muramoto~~. Hexaploid cotton: some plant and fiber properties. Crop Sci. 1969; 9(1~~),~~):27–29.
https://doi.org/10.2135/cropsci1969.0011183X000900010009x

12. Li B~~inglin, –~~, Zhang X~~inrun~~R. Studies on the Hybrid between *G. arboreum* L. and *G.bickii* Prokh.. Acta Genetica Sinica. 1987; 14(2~~),~~):121–126.

13. ~~Yuqiang~~ YQ Sun, ~~Xianlong~~ XL Zhang, ~~Yichun~~ YC Nie, ~~Xiaoping~~ XP Guo, ~~Shuangxia~~ SX Jin & ~~Shaoguang~~ SG Liang. Production and characterization of somatic hybrids between upland cotton (*Gossypium hirsutum*) and wild cotton (*G.klotzschianum* Anderss) via electrofusion. Theoretical and Applied Genetics~~Theor.Appl.Genet.~~ –2004; 109(3~~),~~):472–479.
~~13.~~ https://doi.org/10.1007/s00122-004-1663-3

Formatted: Font color: Auto
Formatted: Font color: Auto
Formatted: Font: (Default) Times New Roman, 12 pt, Font color: Auto
Formatted: Font color: Auto
Formatted: Line spacing: Double, Numbered + Level: 1 + Numbering Style: 1, 2, 3, … + Start at: 1 + Alignment: Left + Aligned at: 0" + Indent at: 0.29"
Formatted: Font color: Auto
Formatted: Font color: Auto
Formatted: Font: (Default) Times New Roman, 12 pt, Font color: Auto
Formatted: Font color: Auto
Formatted: Font:
Formatted: Font color: Auto, Not Highlight
Formatted: Font color: Auto, Not Highlight
Formatted: Font color: Auto
Formatted: Font color: Auto
Formatted: Font color: Auto
Formatted: Font: (Default) Times New Roman, 12 pt, Font color: Auto
Formatted: Font color: Auto, Not Highlight
Formatted: Left, Line spacing: Double, Numbered + Level: 1 + Numbering Style: 1, 2, 3, … + Start at: 1 + Alignment: Left + Aligned at: 0" + Indent at: 0.29"
Formatted: Font color: Auto
Formatted: Font color: Auto
Formatted: Font: (Default) Times New Roman, 12 pt, Font color: Auto
Formatted: Font color: Auto
Formatted: Font color: Auto
Formatted: Font color: Auto
Formatted: Font: (Default) Times New Roman, 12 pt, Font color: Auto
Formatted: Font color: Auto
Formatted: Left, Indent: Left: 0.29", Line spacing: Double, No bullets or numbering
Formatted: Font: (Default) Times New Roman, 12 pt, Font color: Auto

14. Yu X.S.Yu., Chu –B.J.Chu, Liu R.E.Liu, Sun J.Sun, Joseph Jones Brian, Wang H.Z.,.Wang, et al. Characteristics of fertile somatic hybrids of *G.hirsutum* L. and *G. trilobum* generated via protoplast fusion. Theoretical and Applied GeneticsTheor.Appl.Genet. 2012; 125(7),):1503–-1516. https://doi.org/10.1007/s00122-012-1929-0

15. Newaskar GS, Chimote VP, Mehetre SS, JadhavSS Ganesh S. Newaskar Vivek P. Chimote Subhash S. Mehetre Ashok S. Jadhav. . Interspecific hybridization in *Gossypium* L.: characterization of progenies with different ploidy-confirmed multigenomic backgrounds. Plant Breed. –2013; 132(2),):211-216. https://doi.org/10.1111/pbr.12031

16. Zhang Xia Zhang, Zhai CaijiaoCJ Zhai, He LinchiLC He, Guo Qi Guo, Zhang XiangguiXG Zhang,, Xu Peng Xu., et al. Morphological, cytological and molecular analyses of a synthetic hexaploid derived from an interspecific hybrid between *Gossypium hirsutum* and *Gossypium anomalum*. The Crop Journal. 2014; 2(5),):272-277. https://doi.org/ 10.1016/j.cj.2014.06.009

17. Liu Q, Chen Y, Chen Y, Wang Y, Chen J, Zhang T. et al. A new synthetic allotetraploid ($A_1A_1G_2G_2$) between *Gossypium herbaceum* and *G. australe*: bridging for simultaneously transferring favorable genes from these two diploid species into upland cottonA New Synthetic Allotetraploid ($A_1A_1G_2G_2$) between *Gossypium herbaceum* and *G. australe*:Bridging for Simultaneously Transferring Favorable Genes from These Two Diploid Species into Upland Cotton. PLoS One.-2015;10(4),):e0123209.

doi: 10.1371/journal.pone.0123209https://doi.org/10.1371/journal.pone.0123209

Formatted: Left, Line spacing: Double, Numbered + Level: 1 + Numbering Style: 1, 2, 3, … + Start at: 1 + Alignment: Left + Aligned at: 0" + Indent at: 0.29"

Formatted: Font color: Auto

Formatted: Font color: Auto

Formatted: Font color: Auto

Formatted: Font color: Auto

Formatted: Font color: Auto

Formatted: Font color: Auto

Formatted: Font color: Auto

Formatted: Font color: Auto

Formatted: Font color: Auto

Formatted: Font: (Default) Times New Roman, 12 pt, Font color: Auto

Formatted: Font: 12 pt

Formatted: Font:

Formatted: Font color: Auto

Formatted: Font color: Auto

Formatted: Font color: Auto

Formatted: Font: (Default) Times New Roman, 12 pt, Font color: Auto

Formatted: Font: 12 pt

Formatted: Font color: Auto

Formatted: Left, Indent: Left: 0.29", Line spacing: Double, No bullets or numbering

Formatted: Font color: Auto

18. Chen Y, Wang YY, Wang K, Zhu XF, Guo WZ, Zhang TZ, et al. Construction of a complete set of alien chromosome addition lines from *Gossypium australe* in *Gossypium hirsutum*: morphological, cytological, and genotypic characterization. Theoretical and Applied Genetics. 2014; 127:1105–1121. https://doi.org/10.1007/s00122-014-2283-1

19. Chen Y, Wang YY, Zhao T, Yang JW, Feng SL, Nazeer W, et al. A new synthetic amphiploid (AADDAA) between *G. hirsutum* and *G.arboreum* lays the foundation for transferring resistances to verticillium and drought. PLoS One. 2015; 10(6):e0128981. https://doi.org/10.1371/journal.pone.0128981

20. Chen D, Wu YX, Zhang XL, Li FG. Analysis of [*Gossypium capitis-viridis*×(*G.hirsutum*× *G.australe*)^2] trispecific hybrid and selected characteristics. PLoS One. 2015; 10(6):e0127023. https://doi.org/10.1371/journal.pone.0127023

~~17.~~ .

21. Wu YX, Chen D, Zhu SJ, Zhang LF, Li LJ. A new sythetic hybrid (A_1D_5) between *Gossypium herbaceum* and *G. raimondii* and its morphological, cytogenetic, molecular characterization. PLoS One. 2017; 12(2): e0169833. https://doi.org/10.1371/journal.pone.0169833

~~18. Mavromatis, Athanasios G. Pankou, Chrysanthi I. Vlachostergios, Dimitrios N. Xynias, Ioannis N. Roupakias, Dimitrios G. Hybridization between cotton and Malvaceae species as a tool for production of partial interspecific aneuploid cotton plants. Euphytica.2018;214(10). doi:10.1007/s10681-018-2257-5.~~

22. Guo W, Cai C, Wang C, Han Z, Song X, Wang K, et al. A microsatellite-based, gene-rich linkage map reveals genome structure, function and evolution in

Formatted: Font color: Auto, Not Highlight
Formatted: Font color: Auto
Formatted: Font color: Auto, Not Highlight
Formatted: Font color: Auto
Formatted: Font color: Auto, Not Highlight
Formatted: Font color: Auto
Formatted: Font color: Auto, Not Highlight
Formatted: Font color: Auto
Formatted: Font color: Auto, Not Highlight
Formatted: Font color: Auto
Formatted: Font color: Auto, Not Highlight
Formatted: Font color: Auto
Formatted: Font color: Auto, Not Highlight
Formatted: Font color: Auto
Formatted: Font: (Default) Times New Roman, 12 pt, Font color: Auto, Pattern: Clear (White)
Formatted: Font color: Auto
Formatted: Font color: Auto
Formatted: Font: (Default) Times New Roman, 12 pt, Font color: Auto
Formatted: Font color: Auto, Not Highlight
Formatted: Font color: Auto
Formatted: Font: (Default) SimSun, Font color: Auto
Formatted: Font color: Auto
Formatted: Numbered + Level: 1 + Numbering Style: 1, 2, 3, … + Start at: 1 + Alignment: Left + Aligned at: 0" + Indent at: 0.29"
Formatted: Font color: Auto
Formatted: Font color: Auto
Formatted: Left, Line spacing: Double
Formatted: Font: (Default) SimSun
Formatted: Font color: Auto
Formatted: Left, Numbered + Level: 1 + Numbering Style: 1, 2, 3, … + Start at: 1 + Alignment: Left + Aligned at: 0" + Indent at: 0.29"
Formatted: Font color: Auto
Formatted: Font color: Auto
Formatted: Font color: Auto, Not Highlight
Formatted: Left, Line spacing: Double, Don't adjust space between Latin and Asian text, Don't adjust space between Asian text and numbers
Formatted: Font: Pattern: Clear (White)
Formatted: Line spacing: Double, Numbered + Level: 1 + Numbering Style: 1, 2, 3, … + Start at: 1 + Alignment: Left + Aligned at: 0" + Indent at: 0.29"

Gossypium. Genetics. 2007; 176:527–541. https://doi.org/10.1534/genetics.107.070375

~~19.~~ Zhang ~~Doi:10.1534/genetics.107.070375~~

23. J~~. Zhang~~, Guo W~~. Guo~~ & Zhang T~~. Zhang~~. Molecular linkage map of allotetraploid cotton (*Gossypium hirsutum* L.× *Gossypium barbadense* L.) with a haploid population. –*Theoret*ical and Applied Genetics. –2002; 105:1166–1174. https://doi.org/ 10.1007/s00122-002-1100-4

~~20. – *Doi:10,*1007/s00122-002-1100-4~~

~~21.~~24. Zhang J~~un Zhang~~. –Fast screening of microsatellite markers in cotton with PAGE/silver staining. –Cotton Science Sinica. 2000; 12(5):-267–269.

~~22. Di Chen,Yuxiang *Wu*,*Xiling* Zhang,Fuguang Li. Analysis of [*Gossypium capitis-viridis*×(*G.hirsutum*× *G.australe*)^2]Trispecific Hybrid and Selected Characteristics.PLoS One. 2015;10(6),e0127023.doi:10.1371/journal.pone.0127023.~~

~~23.~~25. Galbraith D-W~~-Galbraith~~, Harkins K-R~~-Harkins~~, Maddox J-M~~-Maddox~~, Ayres N-M~~-Ayres~~, Sharma D-P~~-Sharma~~, Firoozabady E~~-Firoozabady~~. Rapid flow cytometric analysis of the cell cycle in intact plant tissues. Science (New York~~,~~ ~~N.Y.~~). –1983; 220(4601~~),~~):1049-1051. https://doi.org/10.1126/science.220.4601.1049

~~24.~~26. Wu ~~Yuxiang~~YX, Yang ~~Fuhong~~FH, Zhao ~~Xiaoming~~XM, Yang ~~Wude~~ WD~~Yang~~. Identification of ~~Tetraploid~~ tetraploid ~~Mutants~~ mutants of ~~Platycodon~~ platycodon grandiflorus by ~~Colchicine~~ colchicine ~~Induction~~induction. Caryologia. 2011; 64(3~~),~~):343-349. https://doi.org/10.1080/00087114.2011.10589801

Formatted ...

Formatted: Line spacing: Double

Formatted: Font: Pattern: Clear (White)

Formatted: Line spacing: Double, Numbered + Level: 1 + Numbering Style: 1, 2, 3, … + Start at: 1 + Alignment: Left + Aligned at: 0" + Indent at: 0.29"

Formatted ...

Formatted: Font color: Auto

Formatted ...

Formatted: Indent: Left: 0.29", Line spacing: Double, No bullets or numbering

Formatted: Font: Italic, Pattern: Clear (White)

Formatted: Line spacing: Double

Formatted: Font: Pattern: Clear (White)

Formatted: Line spacing: Double, Numbered + Level: 1 + Numbering Style: 1, 2, 3, … + Start at: 1 + Alignment: Left + Aligned at: 0" + Indent at: 0.29"

Formatted ...

Formatted: Line spacing: Double

Formatted ...

Field Code Changed

Formatted: Line spacing: Double, Numbered + Level: 1 + Numbering Style: 1, 2, 3, … + Start at: 1 + Alignment: Left + Aligned at: 0" + Indent at: 0.29"

Formatted ...

25. Ma Ping, Huang H C, Li Shezeng, Tang Wenhua. A new inoculation method for verticillium wilt on cotton and its application in evaluating pathogenesis and host resistance.Acta Phytopathol.Sin. 2004;34(6),356-541.

27. Peng S,Lu XL,Gao F,Li GY, Li H.Study on a new rapid inoculation method for Verticillium wilt and fusarium wilt of cotton.J.Cotton Sci.2008;20(3),174-178.Baytar, Asena Akkose Erdogan, Oktay Frary, Anne Frary, Amy Doganlar, Sami. Molecular diversity and identification of alleles for Verticillium wilt resistance in elite cotton (*Gossypium hirsutum* L.) germplasm. Euphytica. 2017; 213:31. https://doi.org/10.1007/s10681-016-1787-y

26.

27. Culp J.E.Endrizzi,E.L.Turcotte,R.J.Kohel.Genetics,cytology,and evolution of *Gossypium*.Adv.Genet. 1985;23,271-375.

28. T-W Culp, Harrell D-C Harrell. Breeding methods for improving yield and fiber quality of upland cotton (*Gossypium hirsutum* L.). Crop Science. -1973; 13.:686-689. https://doi.org/10.2135/cropsci1973.0011183X001300060030x

29. Qian SiyingSY, Huang JunqiJQ, Peng YuejinYY, Zhou BaoliangBL, Ying MiaochengMC, Shen DuanzhuangDZ, .et al. Studies on the hybrid of *G.hirsutum* L.and *G.anomalum* Wawr. & Peyr. and application in breeding. Sci .Agric .Sinica. 1992; 25.:44-51. https://doi.org/

30. Oakes .A.J. A Sterility in certain *Gossypium* hybrids. I. -Prefertilization phenomena. Can .J .Genet Cytol. -1966; 8(4),):818-829. https://doi.org/10.1139/g66-099

31. Sacks Erik J.Sacks. Ovule rescue efficiency of *Gossypium hirsutum*× *G. arboreum* progeny from from-field grown fruit is affected by media composition

Formatted: Line spacing: Double

Formatted: Numbered + Level: 1 + Numbering Style: 1, 2, 3, … + Start at: 1 + Alignment: Left + Aligned at: 0" + Indent at: 0.29"

Formatted: Font color: Auto

Formatted: Font: Italic, Font color: Auto

Formatted: Font color: Auto

Formatted: Font: (Default) Times New Roman, 12 pt, Font color: Auto

Formatted: Font color: Auto

Formatted: Indent: Left: 0.3", Line spacing: Double, No bullets or numbering

Formatted: Font color: Auto

Formatted: Left, Line spacing: Double

Formatted: Left, Line spacing: Double, Numbered + Level: 1 + Numbering Style: 1, 2, 3, … + Start at: 1 + Alignment: Left + Aligned at: 0" + Indent at: 0.29"

Formatted: Font color: Auto

Formatted: Font color: Auto

Formatted: Font color: Auto

Formatted: Font color: Auto

Formatted: Font color: Auto

Formatted: Font color: Auto

Formatted: Font color: Auto

Formatted: Font: (Default) Times New Roman, 12 pt, Font color: Auto

Formatted: Font color: Auto

Formatted: Font color: Auto

Formatted: Font color: Auto

Formatted: Font color: Auto

Formatted: Font color: Auto

Formatted: Font color: Auto

Formatted: Font color: Auto

Formatted: Font: (Default) Times New Roman, 12 pt, Font color: Auto

Formatted: Font color: Auto

Formatted: Font color: Auto

Formatted: Font color: Auto, Not Highlight

Formatted: Font color: Auto

and antimicrobial compounds. Plant Cell Tissue & Organ Culture. 2008; 93(1),):15-20. https://doi.org/10.1007/s11240-007-9316-2

32. Ram S.Ganesh Ram, Ramakrishnan S.Hari Ramakrishnan, Thiruvengadam V.Thiruvengadam, Kannan Bapu J.R.Kannan Bapu. Prefertilization barriers to interspecific hybridization involving *Gossypium hirsutum* and four diploid wild species. Plant Breed. –2008; 127(3),):295-300. https://doi.org/10.1111/j.1439-0523.2007.01453.x

32.

33. Roupakias D.G , A.G. Mavromatis.Cotton Breeding in 21th Century In : Cotton: Technology for the 21th Century, International Cotton Advisory Committee, P.J. Wakelyn , M.R. Chaudhry Eds, 1st Edition , Washington, USA. 2010. pp.19-41.Soltis PS, Soltis DE. The role of genetic and genomic attributes in the success of polyploids. Proc Natl Acad Sci. 2000; 97(13):7051-7057. https://doi.org/10.1073/pnas.97.13.7051

34. Barton NH. The role of hybridization in evolution. Molecular ecology. 2001; 10(3):551-568. https://doi.org/10.1046/j.1365-294x.2001.01216.x

Yuannian Jiao,Norman J. Wickett,Saravanaraj Ayyampalayam,André S. Chanderbali,Lena Landherr,Paula E. Ralph,et al.Ancestral polyploidy in seed plants and angiosperms. Nature. 2011;473(7345),97-100.

Soltis,P.S.,Soltis,D.E.The role of genetic and genomic attributes in the success of polyploids.Proc Natl Acad Sci. 2000;97(13),7051-7057.

35. Siu L Siu, Saha S Saha, Stelly D Stelly, Burr B Burr, Cantrell RG Cantrell. Chromosomal assignment of microsatellite loci in cotton. Journal of Heredity. 2000; 91(4),:326-32. https://doi.org/10.1016/0167-4781(90)90071-9

Formatted: Font color: Auto

Formatted: Font color: Auto

Formatted: Font: (Default) Times New Roman, 12 pt, Font color: Auto

Formatted: Font color: Auto

Formatted: Font color: Auto

Formatted: Font: (Default) Times New Roman, 12 pt, Font color: Auto

Formatted: Left, Line spacing: Double, No bullets or numbering

Formatted: Not Highlight

Formatted: Left, Numbered + Level: 1 + Numbering Style: 1, 2, 3, … + Start at: 1 + Alignment: Left + Aligned at: 0" + Indent at: 0.29"

Formatted: Font color: Auto

Formatted: Font: (Default) Times New Roman, 12 pt, Font color: Auto

Formatted: Font color: Auto

Formatted: Font color: Auto, Not Highlight

Formatted: Font color: Auto, Not Highlight

Formatted: Font color: Auto

Formatted: Font color: Auto, Not Highlight

Formatted: Font color: Auto

Formatted: Font: (Default) Times New Roman, 12 pt, Font color: Auto

Formatted: Font color: Auto, Not Highlight

Formatted: Left, Line spacing: Double

Formatted: Left, Line spacing: Double, Numbered + Level: 1 + Numbering Style: 1, 2, 3, … + Start at: 1 + Alignment: Left + Aligned at: 0" + Indent at: 0.29"

Formatted: Font color: Auto

Formatted: Font color: Auto

Formatted: Font: (Default) Times New Roman, 12 pt, Font color: Auto

N H Barton.The role of hybridization in evolution.Molecular ecology. 2001.10(3),551-568.

33. H.Benbouza, J.M.Lacape, J.M.Jacquemin,B.Courtois,F.B.H.Diouf,D.Sarr,et al.Introgression of the low-gossypol seed & high-gossypol plant trait in upland cotton:Analysis of [(*Gossypium hirsutum*×*G. raimondii*)×*G.sturtianum*] trispecific hybrid and selected derivatives using mapped SSRs.Molecular Breeding.2010;25(2),273-86.

34. L Siu,S Saha,D Stelly,B Burr,RG Cantrell.Chromosomal assignment of microsatellite loci in cotton.Journal of Heredity.2000;91(4),326-32.

35. N H Barton.The role of hybridization in evolution.Molecular ecology. 2001.10(3),551-568.

36. Yuannian Jiao,Norman J. Wickett,Saravanaraj Ayyampalayam,André S. Chanderbali,Lena Landherr,Paula E. Ralph,et al.Ancestral polyploidy in seed plants and angiosperms. Nature. 2011;473(7345),97-100.Rieseberg LH, Sinervo B, Linder CR, Ungerer MC, Arias DM. –Role of gene interactions in hybrid speciation: –evidence from ancient and experimental hybrids. –Science. –1996; 272:-741 – -745. https://doi.org/10.1126/science.272.5262.741 PMID: 8662570

36. Yu Chen,Yingying Wang,Ting Zhao,Jianwei Yang,Shouli Feng,Wajad Nazeer,et al.A New Synthetic Amphiploid (AADDAA) between *Gossypium hirsutum* and *G.arboreum* Lays the Foundation for Transferring Resistances to Verticillium and Drought.PLoS One. 2015;10(6): e0128981. doi:10.1371/journal.pone.0128981.

37. Soltis,P.S.,Soltis,D.E.The role of genetic and genomic attributes in the success of polyploids.Proc Natl Acad Sci. 2000;97(13),7051-7057.

Formatted: Left, Line spacing: Double

Field Code Changed

Field Code Changed

Formatted: Left, Line spacing: Double, Numbered + Level: 1 + Numbering Style: 1, 2, 3, … + Start at: 1 + Alignment: Left + Aligned at: 0" + Indent at: 0.29"

Formatted: Not Highlight

Formatted: Not Highlight

Formatted: Font color: Auto

Formatted: Not Highlight

Formatted: Not Highlight

Formatted: Not Highlight

Formatted: Font color: Auto

Formatted: Not Highlight

Formatted: Font color: Auto

Formatted: Font: (Default) Times New Roman, 12 pt, Font color: Auto

Formatted: Not Highlight

Formatted: Left, Line spacing: Double

Formatted: Not Highlight

Formatted: Font: Italic, Not Highlight

Formatted: Not Highlight

Formatted: Font: Italic, Not Highlight

Formatted: Not Highlight

Formatted: Not Highlight

Formatted: Font: Not Highlight

Formatted: Font: (Default) SimSun

Formatted: Left, Line spacing: Double

38. Zhongfu Ni, Eun-Deok Kim, Misook Ha, Erika Lackey, Jianxin Liu, Yirong Zhang,et al.Altered circadian rhythms regulate growth vigour in hybrids and allopolyploids.Nature. 2009;457,327-331.

37. Kashkush K, Feldman M, Levy AA. Gene loss, silencing and activation in a newly synthesized wheat allotetraploid. Genetics. 2002;160(4),):1651-1659. https://doi.org/10.1017/S0016672301005511

38. Guo WZ，Peng ST. Studlies on the morphogenetics and cytogenetics of interspecific hybrids of *G. hirsutum L.* (4x) and *G. tomentosum* Nutt. (4x). Acta Gossypii Sinica. 1997; 9(1):21-24.

Loren H. Rieseberg. Evolution: replacing genes and traits through hybridization. Curr Biol. 2009; 19(3):119-122. https://doi.org/10.1016/j.cub.2008.12.016

39.

Shaked H, Kashkush K, Ozkan H, Feldman M, Levy A A. Sequence elimination and cytosine methylation are rapid and reproducible responses of the genome to wide hybridization and allopolyploidy in wheat. Plant Cell. 2001; 13(8):1749-1759. https://doi.org/ 10.1105/tpc.010083Loren H.Rieseberg.Evolution:replacing genes and traits through hybridization.Curr. Biol. 2009;19(3),119-122.

40.

H Shaked,K Kashkush,H Ozkan,M Feldman,A A Levy.Sequence elimination and cytosine methylation are rapid and reproducible responses of the genome to wide hybridization and allopolyploidy in wheat.Plant Cell. 2001;13(8),1749-1759.

39.

Formatted: Left, Line spacing: Double, Numbered + Level: 1 + Numbering Style: 1, 2, 3, … + Start at: 1 + Alignment: Left + Aligned at: 0" + Indent at: 0.29"

Formatted: Font color: Auto

Formatted: Font color: Auto

Field Code Changed

Formatted: Font color: Auto

Formatted: Font: (Default) Times New Roman, 12 pt, Font color: Auto

Formatted: Not Highlight

Formatted: Font color: Auto

Formatted: Numbered + Level: 1 + Numbering Style: 1, 2, 3, … + Start at: 1 + Alignment: Left + Aligned at: 0" + Indent at: 0.29", Don't adjust space between Asian text and numbers

Formatted: Font color: Auto

Formatted: Left, Space Before: Auto, After: Auto, Line spacing: Double, Numbered + Level: 1 + Numbering Style: 1, 2, 3, … + Start at: 1 + Alignment: Left + Aligned at: 0" + Indent at: 0.29"

Formatted: Font color: Auto

Formatted: Numbered + Level: 1 + Numbering Style: 1, 2, 3, … + Start at: 1 + Alignment: Left + Aligned at: 0" + Indent at: 0.29", Don't adjust space between Asian text and numbers

Formatted: Font:

Formatted: Font: Font color: Auto

Formatted: Space Before: 0 pt, After: 0 pt, Don't adjust space between Asian text and numbers

Formatted: Left

Formatted: Left, No bullets or numbering

H.Benbouza, J.M.Lacape, J.M.Jacquemin,B.Courtois,F.B.H.Diouf,D.Sarr,et al.Introgression of the low-gossypol seed & high-gossypol plant trait in upland cotton:Analysis of [(*Gossypium hirsutum*×*G. raimondii*)×*G.sturtianum*] trispecific hybrid and selected derivatives using mapped SSRs.Molecular Breeding.2010;25(2),273-86.

Supporting Information

S1 Fig. Growth and formation of the tetraploid S_1. (a) Tetraploid plants with a large pink flower accompanied by yellow pollen. (b) The growth process of the oval boll. (c) Four seeds with brown fibers.

S1 S2 **Fig. Fruits and Fibers of the tetraploid S_1.** (a)

Multiple bolls of S_1 on multiple fruit branches with the sunken navel of the boll tip showing an ergonomic shape with strong bolls. (b) Four-chambered boll with brown fibers. (c) The brown fibers with fiber length of 13 mm. (d) Three-chambered boll with light green and brown cotton fibers.

S2 Fig. Flowers of the tetraploid S2. (A) A pink, cylindrical flower with pink corolla. (B) A funnel-shaped flower with light pink corolla. (C) A funnel-shaped flower with

Formatted: Font color: Auto

Formatted: Don't adjust space between Asian text and numbers

Formatted: Font color: Auto

Formatted: Space Before: 0 pt, After: 0 pt, Don't adjust space between Asian text and numbers

Formatted: Font color: Auto

Formatted: Font color: Auto

Formatted: Font: Times New Roman

Formatted: src, Adjust space between Asian text and numbers, Pattern: Clear (White)

Formatted: Font: Times New Roman, Bold

Formatted: Font: Times New Roman

Formatted: Font: Times New Roman, Not Bold

Formatted: Font: 12 pt

Formatted: Left

Formatted: Font: 12 pt

Formatted: Font color: Auto

Formatted: Font: 12 pt, Not Bold

Formatted: Font: Not Bold

Formatted: Font: Not Bold, Not Superscript/ Subscript

Formatted: Font: Not Bold

white and pink alternating corolla. (D) A white, closed flower. (E) A bell-shaped flower with red corolla and a clear rose-colored spot on the petal base. (F) A semi-closed flower with pink corolla. (G) A bell-shaped flower with pale pink corolla. (H) A small flower with white corolla and no spot on the petal base.

S3 Fig. Fiber colors of the tetraploid S_2. (A) A four-chambered boll with brown fibers. (B) A three-chambered boll with light green and brown cotton fibers. (C) The brown cotton fibers of S1 with an average fiber length of 13 mm.

S3 Fig. Cotton bolls of the TM-1 and F_1 hybrid (TM-1 × S_1). (a)The normal conical boll of TM-1 shown by the red arrow. The abnormally conical boll of F_1 on the right (b) and on the left (c). (d) The deformed hybrid boll of F_1 indicated by yellow arrows.

Figure Legends

Figures:

Formatted: Font: 12 pt, Not Bold, Font color: Auto

Formatted: Font color: Auto

Formatted: Left, Line spacing: Double

Formatted: Font: Not Bold, Font color: Auto

Formatted: Font color: Auto, Not Superscript/ Subscript

Formatted: Font color: Auto

Formatted: Left

Formatted: Left, Line spacing: Double

Formatted: Font: Bold

Formatted: Left

Formatted: Font: Not Bold, Font color: Auto

Formatted: Left

Formatted: Font color: Auto

Formatted: Space Before: Auto, After: Auto, Line spacing: Double, Don't adjust space between Latin and Asian text

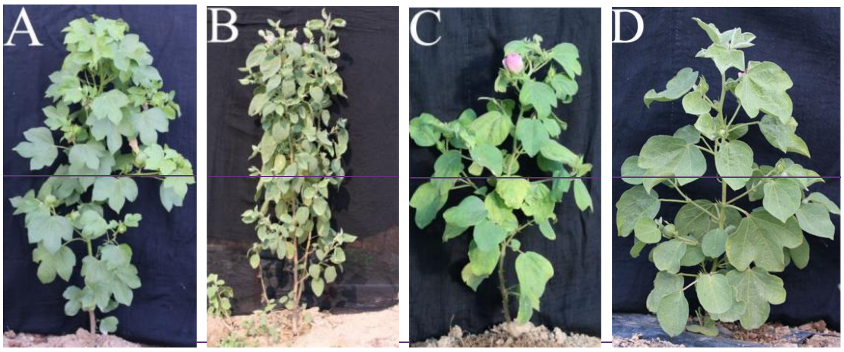

~~Figure 1~~

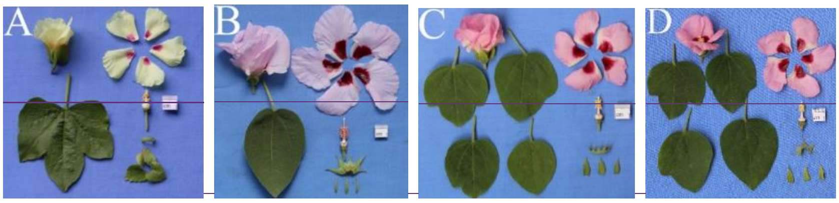

~~Figure 2~~

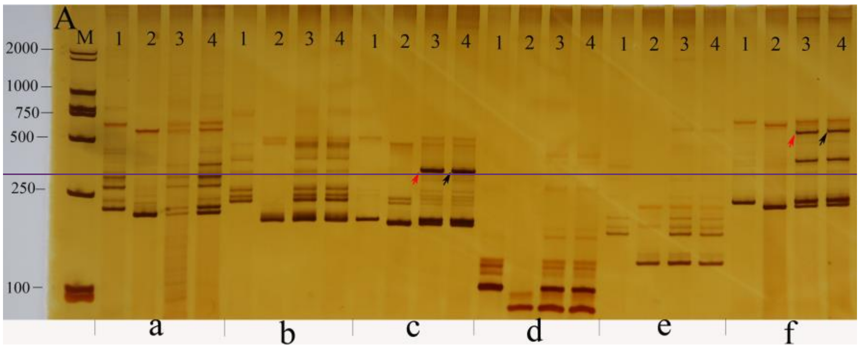

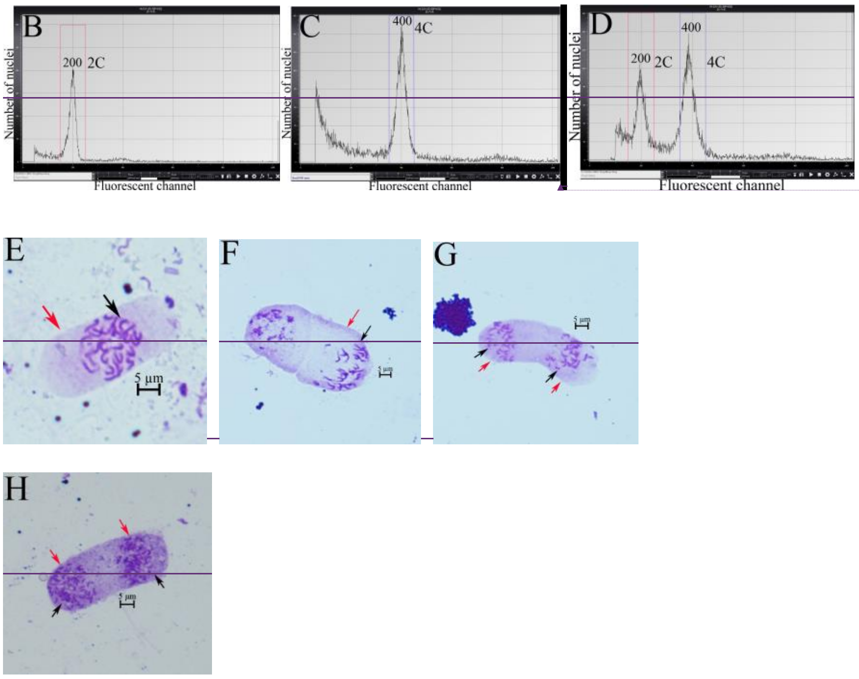

Figure 3

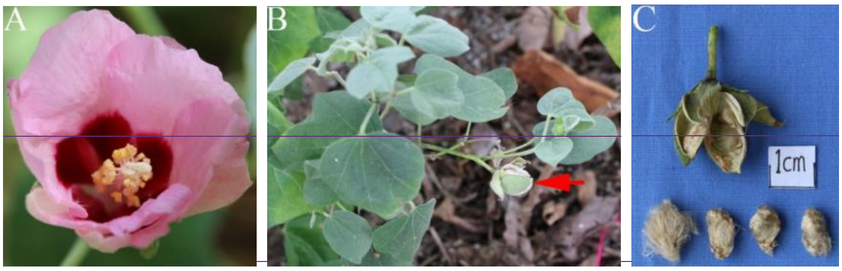

Figure 4

Formatted: Font color: Auto

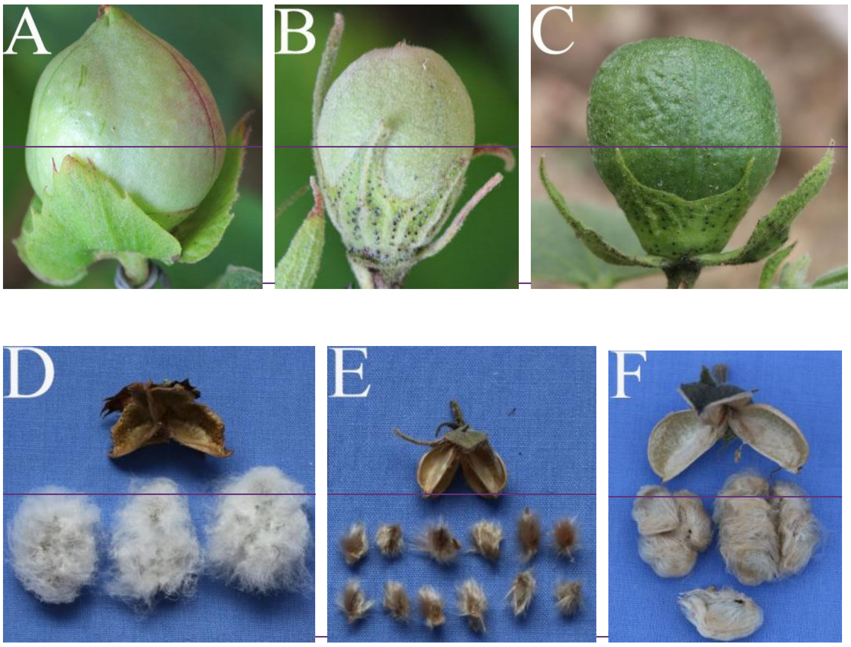

~~Figure 5~~

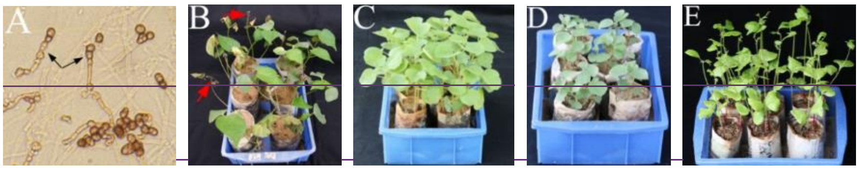

~~Figure 6~~

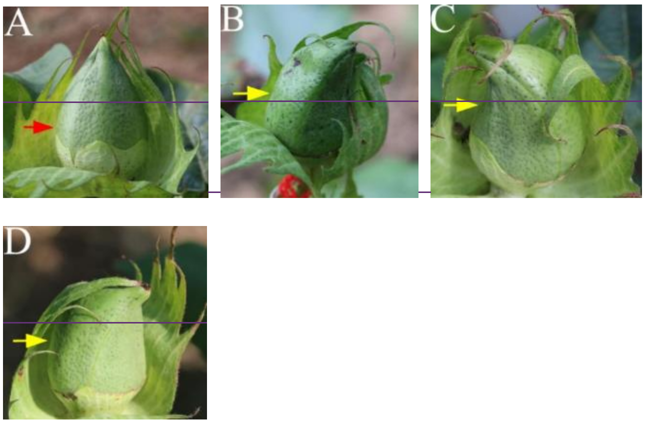

~~Figure 7~~

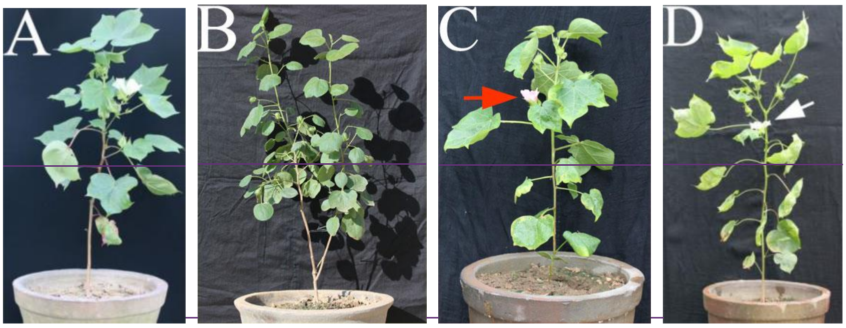

~~Figure 8~~

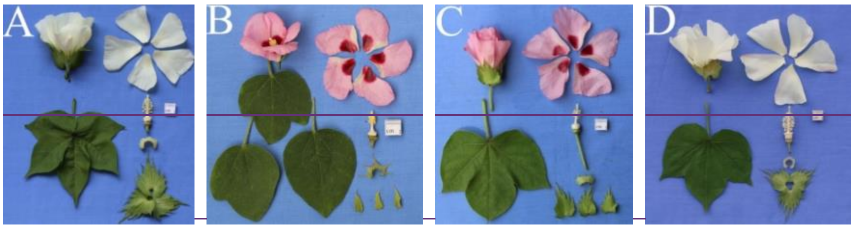

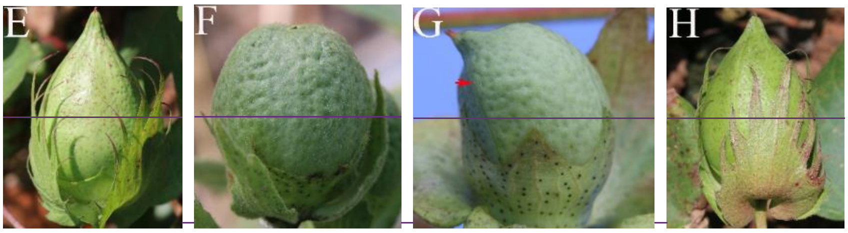

~~Figure 9~~

~~Supporting information~~

Formatted: Space Before: Auto, After: Auto, Line spacing: Double, Don't adjust space between Latin and Asian text

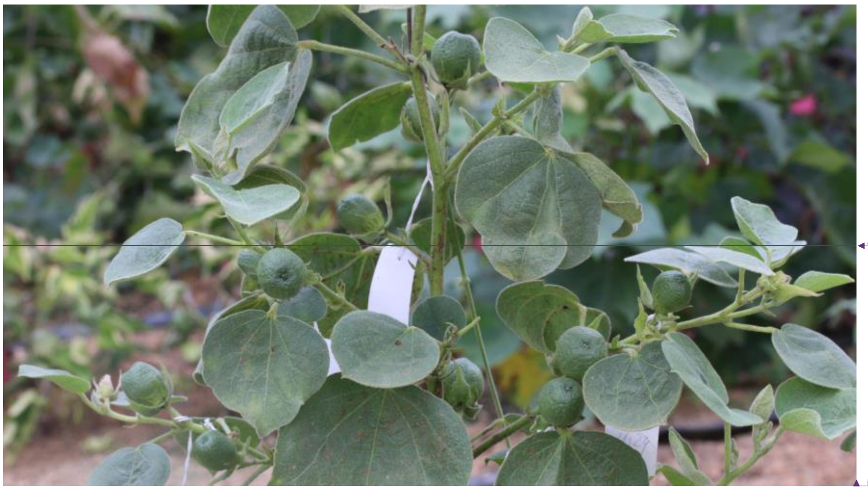

~~**Supporting Figure 1**~~

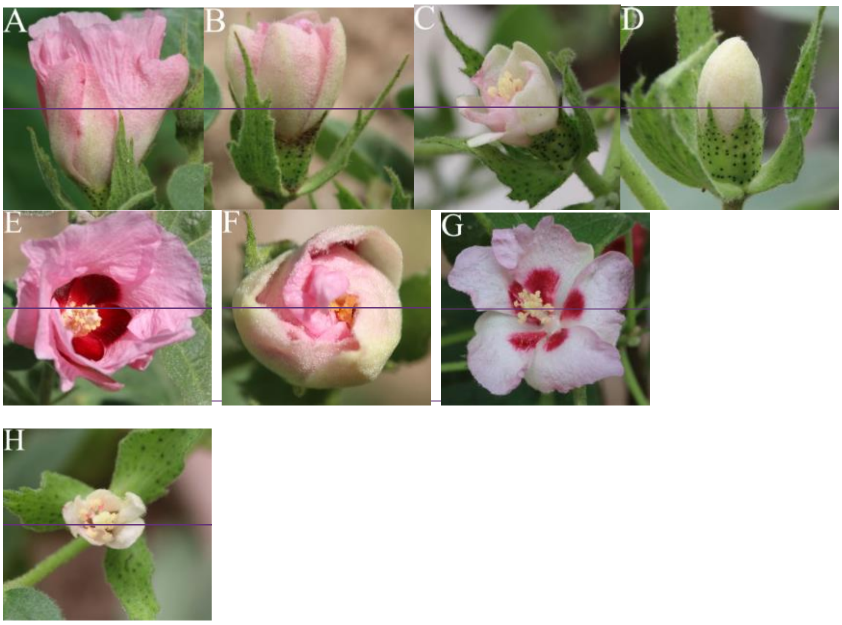

~~**Supporting Figure 2**~~

Formatted: Left, Space Before: Auto, After: Auto, Line spacing: Double, Adjust space between Asian text and numbers

Formatted: Not Highlight

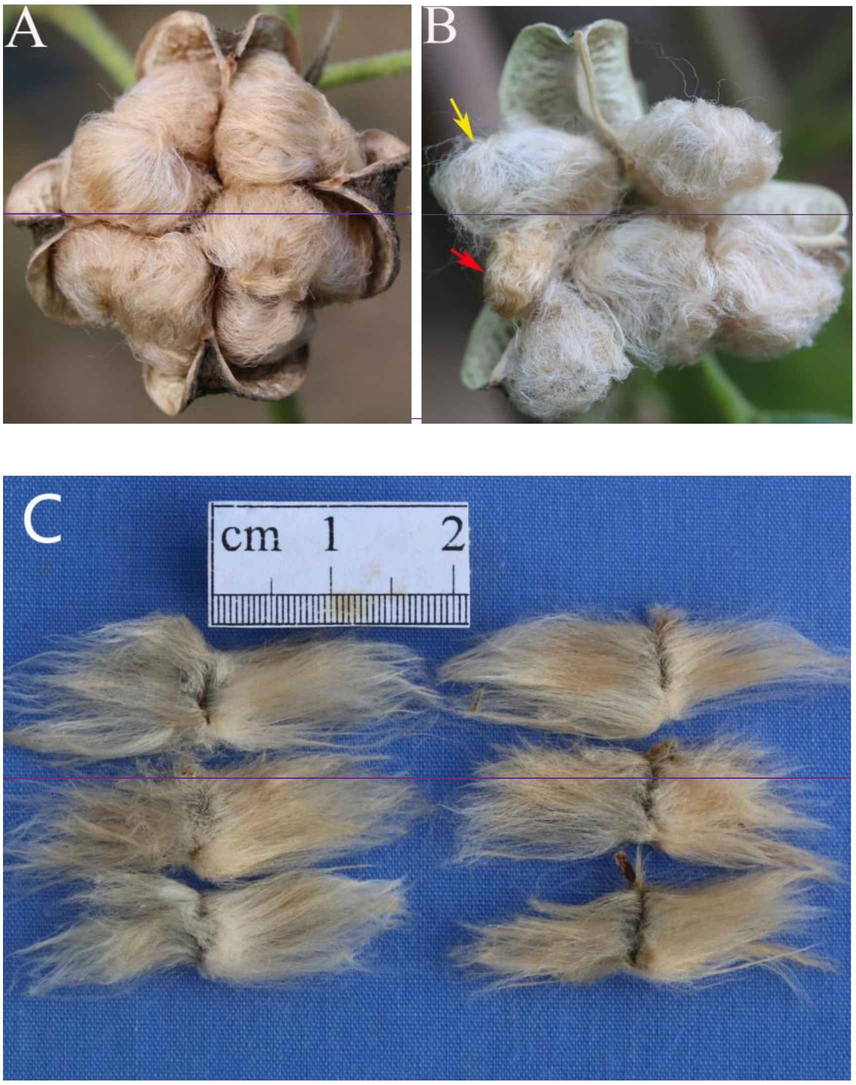

~~**Supporting Figure 3**~~

Formatted: Left, Line spacing: Double

Response to Reviewers

Reviewer #1: The author's concept of the distinction between wild cotton and cultivated cotton is vague. The content of the survey on qualitative and quantitative traits is unclear. The author is confused about the genomic properties of Gossypium. No substantial changes were given to all amendment proposals.

Answer question 1_ The *Gossypium* genus includes 54 species, of which 50 are wild cotton species and 4 are cultivated cotton species including 2 diploid and 2 tetraploid, namely diploid *Gossypium herbaceum* L. and *Gossypium arboreum* L., and tetraploid *G. hirsutum* L. and *G. barbadense* L. [1, 2].It is reported that diploid Gossypium herbaceum L. and Gossypium arboreum L. are classified as cultivated cotton, according to Zaitzev's classification method and named by Linnaeus. Modern discussion on the classification of cotton (Hutchinson, 1947; Mauer, 1954; Fryxell, 1969) are based on Zaitzev's classification method. Due to diploid *Gossypium herbaceum* L. and *Gossypium arboreum* L. is inferior to tetraploid cotton in terms of fiber length and quality, and has been gradually replaced by tetraploid *G. hirsutum* L. and *G. barbadense* L. At present, it is well known that tetraploid *G. hirsutum* L. and *G. barbadense* L. are used for the production and application of cultivated cotton.

1.Fryxell PA. A revised taxonomic interpretation of *Gossypium* L.(Malvaceae). Rheedea. 1992;2(2):108-165.

2.Wang KB, Wendel JF, Hua JP. Designations for individual genomes and chromosomes in Gossypium. J Cotton Res. 2018;1:3.

3.Wendel, JF. New world tetraploid cottons contain old world cytoplasm. *Proc. Natl Acad. Sci. USA* 86, 4132–4136 (1989).

4.Wendel JF, Brubaker CL & Seelanan T. The origin and evolution of *Gossypium*. in *Physiology of Cotton* (eds. Stewart, J. M. et al.) 1–18 (Springer Netherlands, Houten, the Netherlands, 2010).

Answer question 2_The primary purpose of this article is whether to obtain an F_1 interspecific hybrid, and whether the chromosome of the F_1 was successfull y doubled into a fertile tetraploid S_1 or not. The F_1 interspecific hybrid was in fertile, but a new tetraploid cotton S_1 was fertile and suitable for disease-resista nt breeding applications. Related to the content of the survey on qualitative an d quantitative traits about F_1 and S_1. The morphological traits of the F_1 plants (S_1) showed classical hybrid characteristicsaccording to the 14–item (25-item) in vestigation data analysis. The qualitative and quantitative morphological charact eristics of F_1 and S_1 are described in the results, according to the overall struc ture of the article, quantitative descriptions are made for quantitative traits, and qualitative analysis is made for qualitative traits.（1）In the part of plant mor phology identification, it is divided into quantitative and qualitative descriptions. The plants involved are F_1, S_1 and S_2, F_1-1 and F_1-2 and their parents. In ter ms of qualitative morphological characteristics, it involves the color of the flor al organs of the plant, the presence or absence of the floral basal spot, the ch aracteristics of the bracts, the amount of plant hairs, and the boll fertility，whi ch were traits that could be stably inherited and does not change with the exte rnal environment. In terms of quantitative morphological characteristics, it invol ves plant height, leaf size, bract size, bract tooth, No. of teeth，No. of lobes, No. of nectary, corolla length, No. of boll loculi, boll size, etc.（2）we describ e quantitative traits (F_1, S_1 and parents), regarding the number of sample lanes determined by flow cytometry and the observation of the number of chromoso mes in the root tip.（3）According to the statistical results of the test data in t he identification of disease resistance, the disease incidence and disease index

were quantitatively described (the S_2 descendants of S_1 and parents)(Table 5）. （4）Qualitative analysis was carried out in SSR molecular identification(F_1, S_1 and parents) and the investigation of the application potential of S_1 breeding. Qualitative indicators of the color and shape of plant biological characteristics (Table1, 3). Quantitative indicators for plant biological traits(Table 4).

Table 4. Comparison of quantifiable parameters in the S_2 descendants of S_1 with their parental plants

Plants	Plant height(cm)	Internode of stem(cm)	Vegetative branches	Fruiting branches	Leaf size(length×width, cm)	Bract size (length×width, cm)	Boll size (length×width, cm)	Corolla length(cm)	Stigma length(cm)	Length of fiber(mm)
S_2-1	156.68	5.18	5	13	8.26×9.76	1.58×1.10	3.11×2.56	4.35	1.14	12.86
S_2-2	170.00	5.21	7	15	9.23×10.81	1.60×1.10	3.26×2.65	4.46	1.15	11.12
S_2-3	146.23	5.06	5	14	9.51×10.72	1.50×0.98	2.89×2.67	4.14	1.22	10.46
S_2-4	138.17	4.89	6	15	8.62×9.92	1.30×0.89	3.05×2.38	3.95	1.21	12.98
S_2-5	129.28	5.07	5	12	9.81×10.61	1.25×0.76	2.97×2.56	4.28	1.36	10.56
S_2-6	89.86	4.86	4	9	6.33×7.50	1.38×0.72	2.84×2.80	2.51	1.28	9.78
S_2-7	70.00	3.16	4	7	5.71×6.20	1.3×0.72	2.78×2.36	2.46	0.95	8.23
S_2-8	98.56	3.38	4	8	8.21×9.80	1.35×0.72	2.75×2.58	2.67	0.89	7.46
S_2-9	158.46	4.78	5	42	8.63×10.20	1.35×0.68	3.08×2.59	4.39	1.16	10.95
S_2-10	169.76	4.86	5	15	9.90×11.00	1.59×1.02	3.19×2.98	4.34	1.35	10.96
G.herbaceum	80.00	3.45	4	7	5.21×6.18	1.10×1.32	3.56×2.87	2.18	0.85	17.70
G.nelsonii	200.00	4.96	7	15	12.86×13.98	1.2×0.25	2.31×2.06	3.53	1.48	5.60

Table 5. Identification of cotton Verticillium wilt resistance in the S_2 descendants of S_1 with their parental plants.

Cultivar	Disease index	Relative resistance index	Classification
Jimian11	44.00±3.62	22.00±1.81	S
S_2-1	0.00	0.00	I
S_2-2	0.00	0.00	I
S_2-3	0.28±0.68	0.14±0.34	HR
S_2-4	0.00	0.00	I
S_2-5	0.00	0.00	I
S_2-6	0.56±0.12	0.28±0.06	HR
S_2-7	0.00	0.00	I
S_2-8	0.00	0.00	I
S_2-9	0.16±2.86	0.8±1.43	HR
S_2-10	0.00	0.00	I
G. nelsonii Fryx.	0.00	0.00	I
G. herbaceum L.	1.68±1.64	0.84±0.82	HR

Answer question 3_According to the articles(Wang KB, 2018; Wendel, J. F.,2010) related to *Gossypium herbaceum* L. (Genome group A, Individual genome A_1) and *Gossypium nelsonii* Fryx. (Genome group G, Individual genome G_3)(Table 1, Table 2), the genus is divided into 8 diploid genome groups (A through G, and K), as well as one allopolyploid clade (AD genome) formed from ancient merger and chromosome doubling from A and D genome ancestors. These genome groups were initially defined based on comparative chromosome sizes and chromosome behavior in interspecific hybrids (Beasley 1942; Stephens 1947;Phillips 1966; Fryxell 1992; Endrizzi et al. 1985).

Table 1 Taxonomic, cytogenetic and geographic diversity of species of *Gossypium*. Included are descriptions are each genome group and the collective geographic distribution of the included species. Genomic placements and taxonomic status of species enclosed by parentheses remain to be established as they are poorly known

Genome group	Number of species	Species presently recognized	Geographic distribution
A	2	*G. arboreum, G. herbaceum*	Wild forms known only from Southern Africa, but with an indigenous domesticated range encompassing parts of Africa, the middle east, and Asia
B	3–4	*G. anomalum, G. triphyllum, G. capitis-viridis, (G. trifurcatum)*	Africa, Cape Verde Islands
C	2	*G. sturtianum, G. robinsonii*	Central and Western Australia
D	13–14	*G. thurberi, G. armourianum, G. harknessii, G. davidsonii, G. klotzschianum, G. aridum, G. raimondii, G. gossypioides, G. lobatum, G. trilobum, G. laxum, G. turneri, G. schwendimanii, (G. sp.nov.)*	Primarily Mexico, with range extensions into Peru, the Galapagos Islands, and southern Arizona
E	5–9	*G. stocksii, G. somalense, G. areysianum, G. incanum, G. trifurcatum, (G. benidirense), (G. bricchettii), (G. vollesenii), (G. trifurcatum)*	Poorly understood native ranges of many species, but collectively in parts of the Arabian Peninsula, Northeast Africa, and Southwest Asia
F	1	*G. longicalyx*	Endemic to East Africa
G	3	*G. bickii, G. australe, G. nelsonii*	Central Australia
K	12	*G. anapoides, G. costulatum, G. cunninghamii, G. enthyle, G. exiguum, G. londonderriense, G. marchantii, G. nobile, G. pilosum, G. populifolium, G. pulchellum, G. rotundifolium*	Northwest Australia, Cobourg Peninsula, Northern Territory, Australia
AD	7	*G. hirsutum, G. barbadense, G. tomentosum, G. mustelinum, G. darwinii, G. ekmanianum, G. stephensii*	Wild species are from the New World tropics and subtropics, including Hawaii, NE Brazil, the Dominican Republic, and the Galapagos Islands. A recently described species (*G. stephensii*) is from the Wake Atoll near French Polynesia. The indigenous cultivated range of the two domesticated species *G. barbadense* and *G. hirsutum* encompasses many parts of the drier New World tropics and subtropics, with overlapping ranges in the Caribbean and elsewhere

Table 2 Nomenclature of individual genomes and chromosomes for each species in *Gossypium*, with Chinese translation of species names

Species names		Individual genome[a]	Individual chromosome
Scientific names	Chinese translations		
G. hirsutum Linnaeus	陆地棉	$(AD)_1$	A_h01-A_h13; D_h01-D_h13
G. barbadense Linnaeus	海岛棉	$(AD)_2$	A_b01-A_b13; D_b01-D_b13
G. tomentosum Nuttall ex Seemann	毛棉	$(AD)_3$	$A_{tt}01-A_{tt}13$; $D_{tt}01-D_{tt}13$
G. mustelinum Miers ex Watt	黄褐棉	$(AD)_4$	A_m01-A_m13; D_m01-D_m13
G. darwinii Watt	达尔文氏棉	$(AD)_5$	A_d01-A_d13; D_d01-D_d13
G. ekmanianum Wittmack	艾克棉	$(AD)_6$	A_e01-A_e13; D_e01-D_e13
G. stephensii J. Gallagher, C. Grover & Wendel	斯蒂芬氏棉	$(AD)_7$	A_s01-A_s13; D_s01-D_s13
G. herbaceum Linnaeus	草棉	A_1	A_101-A_113
G. somalense (Gurke) Hutchinson	索马里棉	E_2	E_201-E_213
G. areysianum Deflers	亚雷西亚棉	E_3	E_301-E_313
G. incanum (Schwartz) Hillcoat	灰白棉	E_4	E_401-E_413
G. longicalyx Hutchinson & Lee	长萼棉	F_1	F_101-F_113
G. bickii Prokhanov	比克氏棉	G_1	G_101-G_113
G. australe Mueller	澳洲棉	G_2	G_201-G_213
G. nelsonii Fryxell	奈尔逊氏棉	G_3	G_301-G_313
G. exiguum Fryxell, Craven & Stewart	小小棉	K_1	K_101-K_113
G. rotundifolium Fryxell, Craven & Stewart	圆叶棉	K_2	K_201-K_213
G. populifolium (Bentham) Mueller ex Todaro	杨叶棉	K_3	K_301-K_313
G. pilosum Fryxell	稀毛棉	K_4	K_401-K_413

1.Wang KB, Wendel JF, Hua JP. Designations for individual genomes and chromosomes in Gossypium. J Cotton Res. 2018;1:3.

2.Wendel JF, Brubaker CL & Seelanan T. The origin and evolution of *Gossypium*. in *Physiology of Cotton* (eds. Stewart, J. M. et al.) 1–18 (Springer Netherlands, Houten, the Netherlands, 2010).

Reviewer #2: (No Response)

Reviewer #3: GENERAL COMMENT

Although the authors incorporated the suggestions from the reviewers, I feel th at this paper must be improved, by English Editing and a careful extension of discussion with mention to other research works and results found in internati onal references.

Answer question _ This paper has been edited to ensure that the language is c lear and free of errors. The logical presentation of ideas and the structure of t he paper were also checked during the editing process. The edit was performe d by professional editors at Editage, a division of Cactus Communications.

Answer question _ Added relevant research results published internationally in t he discussion section of the article.

We have already obtained an interspecific hybrid F_1 of *G. herbaceum* and *G. raimondii*. The F_1 plants are sterile in the field, leading to various methods being attempted to double their chromosome but without success [21]. In this study, we successfully obtained an infertile interspecific F_1 hybrid and managed to overcome the genetic incompatibility barriers between two distantly related species. Furthermore,We doubled the chromosomal content of this F_1 hybrid throug colchicine immersion, overcoming the considerable challenges of interspecific hybrid sterility, to successfully synthesize a new S_1 tetraploid cotton species.

21. Wu YX, Chen D, Zhu SJ, Zhang LF, Li LJ. A new sythetic hybrid (A_1D_5) between *Gossypium herbaceum* and *G. raimondii* and its morphological,

cytogenetic, molecular characterization. PLoS One. 2017; 12(2): e0169833. doi:10.1371/journal.pone.0169833.

In the present study, SSR detection revealed the presence of new specific bands in the hybrids that were not detected in the parents, indicating that new combinations or mutations of chromosomes are present in the interspecific progeny (Genomic A and Genomic C). This result might be due to chromosomal rearrangements in this newly formed hybrid [37], which was consistent with the previous reports on trispecific hybrid [20].

37. Kashkush K, Feldman M, Levy AA. Gene loss, silencing and activation in a newly synthesized wheat allotetraploid. Genetics. 2002;160(4):1651-1659.

20. Chen D, Wu YX, Zhang XL, Li FG. Analysis of [*Gossypium capitis-viridis*×(*G.hirsutum*× *G.australe*)^2] trispecific hybrid and selected characteristics. PLoS One. 2015;10(6):e0127023. doi:10.1371/journal.pone.0127023.

The preliminary examination showed that the disease resistance of the tetraploid S_2 was better than *G. herbaceum* and the same level as *G. nelsonii*.Increased disease resistance also occurs in interspecific hybrids between *G.hirsutum* × *G. arboretum* [19].

19. Chen Y, Wang YY, Zhao T, Yang JW, Feng SL, Nazeer W, et al. A new synthetic amphiploid (AADDAA) between *G. hirsutum* and *G.arboreum* lays the foundation for transferring resistances to verticillium and drought. PLoS One. 2015;10(6):e0128981. doi:10.1371/journal.pone.0128981.

The sterility of F_1-1 hybrid plants was consistent with the results of interspecific hybrids of *G. hirsutum* L. and *G. tomentosum* Nutt.[38], which may be caused by the incoordination of physiological processes in the hybrid offspring.

38. Guo WZ，Peng ST. Studlies on the morphogenetics and cytogenetics of interspecific hybrids of *G. hirsutum L.* (4x) and *G. tomentosum* Nutt. (4x). Acta Gossypii Sinica. 1997;9（1）:21-24.

I propose some differentiation in the subtitles and in titles of the tables (please find STIKCY NOTES in the manuscript).
(e.g p.p. 173 A title : Root tip chromosome observations changed as KARYOT YPE ANALYSIS)

All new suggestions and corrections are indicated by Highlight Notes in the M anuscript (please change all the proposed words or terms or terminology as pro posed in the NOTE BOX).

Answer question _ According to the prompts of STIKCY NOTES, correspondin g changes have been made in the article.
NOTICE: Micronaire value up to 6, is not a desirable trait for fiber quality. Please not confuse the reader.

THE RESULTS FROM THIS RESEARCH ARE VERY IMPORTANT.

The researchers offer lot of work onto these experiments but is important to clarify the useful data and give the most significant conclusions in a clear way.

Answer question _ Considering the role of the micronaire value of cotton fiber in the article, it is not our purposes, we have deleted it.

PLEASE WRITE THE CONCLUSION
