# Supplementary material for: Morphological description of a novel synthetic allotetraploid(A1A1G3G3) of Gossypium herbaceum L.and G.nelsonii Fryx. suitable for disease-resistant breeding applications
Source: PLoS One. 2020 Dec 3;15(12):e0242620. doi: 10.1371/journal.pone.0242620 (PMC7714114; doi:10.1371/journal.pone.0242620)
Supplement: Attachment — Submitted filename: REVIEWER (3).pdf [file pone.0242620.s010.pdf]

REVIEWER 3

ALL CORRECTIONS AND SUGGESTIONS INCLUDED IN THE ATTACHED TEXT

ABSTRACT

LINE 18 PLEASE REWRITE AS
Wild species of Gossypium ssp. are an important source of traits for improving commercial cotton cultivars.

LINE 23 SUBSTITUTE WITH
......cotton genotype..........

LINE 24 REWRITE AS

First of all, G. herbaceum and G. nelsonii were used as parents for interspecific hybridization to obtain F1 generation.

LINE 26 REWRITE AS

Afterwards, apical meristems of the F1 diploid cotton plants, were treated ......

LINE 27

The new interspecific F1 hybrid and S1 cotton plants originated from chromosome duplication, were tested via morphological and molecular markers and confirmed tetraploidy through flowrometric and cytological identification.

LINE 29 REWRITE AS

The S1 tetraploid cotton plants were crossed with ....

LINE 30 REWRITE AS

These S2 off springs, were tested for resistance to Verticillium wilt and demonstrated adequate tolerance to this fungus.

LINE 31 SUBSTITUTE WITH

The results show that the new S1 cotton line could be used as parental material for hybridization with G. hirsutum to produce ....

LINE 33 REPLACE WITH
This new S1 allotetraploid genotype will be contribute to the ....

LINE 62 REPLACE WITH

...............biological isolation barriers...........

LINE 63 SUBSTITUTE WITH

…………..hybridize wild diploid cotton species with…………

LINE 85 REPLACE

…………number and form ……….

LINE 87 REPLACE

genetic foundation

WITH germplasm of cotton

LINE 88 REPLACE

and breeding

WITH ………………..and a new tool for breeding

LINE 98 PLEASE SEE OUR SUGGESTIONS IN THE TEXT

LINE 118 PLEASE SEE OUR SUGGESTIONS IN THE TEXT

LINE 150 PLEASE SEE OUR SUGGESTIONS IN THE TEXT

LINE 157 PLEASE SEE OUR SUGGESTIONS IN THE TEXT

LINE 160 PLEASE SEE OUR SUGGESTIONS IN THE TEXT

LINE 163 PLEASE SEE OUR SUGGESTIONS IN THE TEXT

LINE 168 THIS PARAGRAPH

“A Nikon Eclipse Ni-E biological microscope and camera system was

used to observe, count, and photograph the chromosomes in the root-tip cells.

Photoshop software was used to edit the images”

IS NOT NECESSARY TO WRITE BECAUSE YOU EXPLAIN THE PROCESS ABOUT IMAGES IN A SPECIFIC PARAGRAPH (Lines 202 - 205)

LINE 217 PLEASE SEE OUR SUGGESTIONS IN THE TEXT

LINE 239 PLEASE SEE OUR SUGGESTIONS IN THE TEXT

LINE 240 PLEASE SEE OUR SUGGESTIONS IN THE TEXT

LINE 261 PLEASE SEE OUR SUGGESTIONS IN THE TEXT

LINE 285 PLEASE SEE OUR SUGGESTIONS IN THE TEXT

LINE 286 PLEASE SEE OUR SUGGESTIONS IN THE TEXT

LINE 326 PLEASE SEE OUR SUGGESTIONS IN THE TEXT

LINE 335 PLEASE SEE OUR SUGGESTIONS IN THE TEXT

LINE 336 PLEASE SEE OUR SUGGESTIONS IN THE TEXT

LINE 337 PLEASE SEE OUR SUGGESTIONS IN THE TEXT

LINE 366 PLEASE SEE OUR SUGGESTIONS IN THE TEXT

LINE 378 PLEASE SEE OUR SUGGESTIONS IN THE TEXT

LINE 413 PLEASE SEE OUR SUGGESTIONS IN THE TEXT

LINE 414 REPLACE WITH

.........some of them grew until flowering stage and others stopped their growth having low fertility or sterility.

LINE 443 REPLACE WITH

.....and molecular genetic techniques are required for understanding the nature and usefulness of this new germplasm.

LINE 528 PROPOSE for REPLACE AS

It was suggested a more detailed in - depth and comprehensive research to ......
