# Supplementary material for: Morphological description of a novel synthetic allotetraploid(A1A1G3G3) of Gossypium herbaceum L.and G.nelsonii Fryx. suitable for disease-resistant breeding applications
Source: PLoS One. 2020 Dec 3;15(12):e0242620. doi: 10.1371/journal.pone.0242620 (PMC7714114; doi:10.1371/journal.pone.0242620)
Supplement: Attachment — Submitted filename: REVIEWER 1 - SUGGESTIONS.docx [file pone.0242620.s005.docx]

**COMMENTS FROM REVIEWER 1**

**For PONE-D-20-02970**

**KEY MESSAGE:**

THE STUDY ENTITLED “Morphological and genetic description of a novel synthetic allotetraploid of Gossypium herbaceum L. and Gossypium nelsonii Fryx.“ IS REALLY AN INTERESTING TOPIC BECAUSE PRESENTED FOR THE FIRST TIME THE PRODUCTION OF A NEW HYBRID COMBINATION IN COTTON, BETWEEN THE SPECIES G. HERBACEUM X G. NELSONII.

THE FINAL RESULT IS USEFUL AS PLANT GENETIC RESOURCE AND ALSO FOR BASIC RESEARCH INFORMATION.

HOWEVER THIS STUDY HAS SOME METHODOLOGICAL FLAWS AND REQUIRES **MAJOR REVISIONS** MAINLY ON ENGLISH LANGUAGE (A CAREFULL EDITING) IN ORDER TO MEET THE PLOs REQUIREMENTS.

- FIRST OF ALL IS NECESSARY TO REWRITE SOME OF THE PARAGRAPHS

AS an EXAMPLE, you must REWRITE as presented below:

**ABSTRACT**

The fungus *Verticillium dahliae* Kleb. infects cotton under field conditions mainly during seed germination period and also during its growth until maturing of cotton bolls, having seriously effects to final cotton yield *per* hectare.

Unless, most of wild cottons exhibit high level of resistance to this pathogen, the cultivated upland cotton is susceptible to this kind of pathogen.

……………………………………………………….. interspecific hybridization can facilitate the development of new types of cotton resources. This newly synthesized tetraploid cotton species is valuable for the enrichment of cotton genetic resources and for breeders to transfer the resistance in verticillium to new upland breeding lines.

**TERMS & TERMINOLOGY**

**YOU WRITE**	**REWRITE as**

Hybridisation	Hybridization

an interspecies F1 hybrid.	an interspecific F1 hybrid

was characterised by morphological analysis,	…..was described using

polymorphism detection, morphological markers,

detecting DNA polymorphism

and following ploidy analysis

and microscopic observation …

This successful crossbreeding of S1	The S1 interspecific hybrid was crossed with the upland cotton

variety TM1 for…….

………………………………………………………………………………………………………………………………………….

**WHAT THE AUTHORS NEED TO DO FOR IMPROVE THEIR PAPER**

- I THEORIZED THAT YOU MUST FOLLOW A LANGUGE EDITING FIRSTLY AND AFTERWARDS WE COULD CAPABLE TO FOLLOW DETAILED CORRECTIONS AND SUGGESTIONS.
- I AM SORRY BUT THERE MAINY TERMS AND SENTENCES THAT MUST BE REWRITE USING BETTER TERMINOLOGY.

ALL THE SENTENCES, TERMS AND WORDS UNDERLINED INTO THE TEXT BY YELLOW COLOUR SHOULD BE CHANGED.

- PLEASE FOLLOW A LANGUAGE EDITIND FROM A PERSON WHO HAS ENGLISH AS NATIVE LANGUAGE BECAUSE YOU LOOSE MUCH OF THE VALUE OF YOUR WORK .

**PLEASE USE THESE REFERENCES FOR HELPING YOU TO THE BETTER USE OF TERMINOLOGY**

Benbouza H, Lacape JM, Jacquemin JM, Courtois B, Diouf FBH, Sarr D, et al. **Introgression of the low-gossypol seed & high-gossypol plant trait in upland cotton: Analysis of [(Gossypium hirsutum×G. raimondii)^2^ × G. sturtianum] trispecific hybrid and selected derivatives using mapped SSRs**.

Mol Breeding. 2010; 25: 273–286.

Mavromatis et al. (2018). **Hybridization between cotton and Malvaceae species as a tool for production of partial interspecific aneuploid cotton plants**

(EUPHYTICA, 2018)

**STUDY DESIGN :** is the appropriate for the question of the research

**METHODS :**

**TABLES :**

Table 1. Comparison of shape of some morphological traits in F1 and S1 interspecific hybrids

Table 2. Comparison of some quantitative traits in F1 and S1 F1 and S1 interspecific hybrids

Table 3. Comparison of morphological traits (shape and colour) of plant parts in TM1 upland cultivar used as female parent, S1 interspecific hybrid used as male parent and in 2 (F1-1 and F1-2) cotton plant progenies

**FIGURES :**

**In Figure 5 (FLOW CYTOMETRIC ANALYSIS) is necessary to present all parameters used into the instrument FC (GAIN , CV, FILTERS etc) under of each diagram**

**FINALLY** BECAUSE AS YOU WRITE IN THE DISCUSSION GIVING A PROPOSAL

It should be noted that we have only preliminarily assessed the authenticity of the F1 generation obtained from crosses between TM-1 and S1 through morphological characterisation. Further studies are required to confirm they are true hybrids using cytology and molecular genetics techniques.

IT IS NECESSARY TO INCORPORATE THESE FINDINGS IN THE CONCLUSIONS
