# Supplementary material for: Morphological description of a novel synthetic allotetraploid(A1A1G3G3) of Gossypium herbaceum L.and G.nelsonii Fryx. suitable for disease-resistant breeding applications
Source: PLoS One. 2020 Dec 3;15(12):e0242620. doi: 10.1371/journal.pone.0242620 (PMC7714114; doi:10.1371/journal.pone.0242620)
Supplement: Attachment — Submitted filename: renamed_26032.doc [file pone.0242620.s006.doc]

**A cover letter describing your response to our editorial requests**

Dear Editor,

First of all, I would like to thank the editorial staff for their preliminary approval of our article. At the same time, I would also like to thank the review experts for their careful review and valuable Suggestions.

We will respond to the questions raised as soon as possible, and will answer them truthfully one by one,and try our best to revise and write the article as standardized as possible.

I uploaded the original photo of the article picture, the processing format of this article pictures,If you have any questions, please reply immediately and we will respond as soon as possible.

lastly, we hope this article could be accepted by dear editing department and published as soon as possible.

Sincerely

Di Chen

2020-05-23

**Reviewer Comments:**

5. Review Comments to the Author
   Please use the space provided to explain your answers to the questions above. You may also include additional comments for the author, including concerns about dual publication, research ethics, or publication ethics. (Please upload your review as an attachment if it exceeds 20,000 characters)

Reviewer #1:

The manuscript “Morphological and genetic description of a novel synthetic allotetraploid of *Gossypium herbaceum* L. and *Gossypium nelsonii* Fryx” created a new germplasm of cotton through remote hybridisation for a resistant source of wild cotton. *G. herbaceum* L. crossed with *G. nelsonii* Fryx. to produce an interspecies F1 hybrid cotton. Colchicine was used to double its chromosomal content, leading to the successful generation of a synthetic S1 tetraploid. The newly created S1 tetraploid showed morphological variations in its flowers, cotton bolls, and fibres and was partially resistant to *Verticillium dahliae* Kleb . The S1 tetraploid was hybridised with upland cotton to produce two types of cotton with different characteristics. This newly synthesised tetraploid cotton species is a valuable resource for cotton resistance breeding and for the enrichment of cotton genetic resources. The results are informative, but there are some major concerns need to be addressed.
1. The description of phenotypic morphological analysis of the diploid F_1_ hybrid and the synthetic S_1_ tetraploid is poorly organized. The author's investigation of morphological in materials and methods has not been described in detail. Even with reference to the methods of previous reference, no specific survey data and criteria are given. In the survey results, the key traits such as the germinating stage and the flowering stage are unclear. Moreover, wild cotton is mostly perennial wild material, and its germinating stage and flowering stage belongs to agronomic traits. It is not suitable for investigation and comparative analysis of wild materials.

__Answer：We have added the key traits such as germination and flowering of new germplasm cotton.The F_1_ hybrid were sown in a pot and all germinated after 5 days. After the first true leaves were level, the seedlings were transplanted to the field. Floral buds appeared after 43-45 days and flowering occurred after 68-70 days. The S_1_ were sown in a pot and all germinated after 5 days. After the first true leaves were level, the seedlings were transplanted to the field. Floral buds appeared after 45-50 days and flowering occurred after 70-75 days. Boll opening occurred after 107-115 days.

The new cotton germplasm was created by using wild cotton through distant hybridization and is the descendant of wild cotton.When investigating and analyzing it, it involves genetic problems.To explain the genetic problems of new germplasm, the source needs to be traced back. Therefore, it is necessary to list wild cotton parents as a reference in the study in order to analyze and explain.The reviewer mentioned that wild cotton is mostly perennial wild material, this statement is correct.

In fact, all cotton is a perennial plant in tropical regions, including wild cotton and cultivated cotton (such as *G. hirsutum* L. *G. barbadense* L.*G.* *herbaceum* L.*G. arboreum* L. ). The morphological traits of various cotton species grown in the same environment difference.
2. Statistical analysis of leaf shape, plant height, boll shape and other traits is not appropriate. Because the leaf shape will change according to the different growth stages of the plant, the plant height will vary greatly due to changes in the growth environment, and the boll shape will also vary in different positions of the plant. The number of bolls per plant is also strongly related to nutritional status and planting density, so the author's description of these traits seems meaningless.

__Answer：Plant morphology is a characteristic expression of a species, mainly including plant morphology traits such as plant type, stem, leaf, flower and cotton boll.Regarding the color of the floral organs of the plant, the presence or absence of the floral basal spot, the characteristics of the bracts, the amount of plant hairs, and the boll fertility is a trait that can be stably inherited and does not change with the external environment.In a limited time, these descriptions of the new germplasm we created, please dear editors and review experts to understand.We describe morphology and only want to show the new cotton germplasm as much as possible. If we talk about its significance, the birth event of new cotton germplasm is meaningful in itself.

3. When statistically analysis the differences in phenotypic traits, it is necessary to distinguish between qualitative and quantitative traits. For F_1_, in the case of a limited single plant, it cannot be compared with a tetraploid with a larger number of offspring. As for the results of the investigation against Verticillium wilt, as it is a quantitative trait, it is recommended to compare and analyze the resistance differences of different isolated plants of S_1_.

__Answer：Due to the low number of diploid F_1_ hybrids and tetraploid S_1_ plants, these two materials cannot be used for cotton Verticillium wilt resistance experiments.In fact, in this study, we used the descendant material S_2_ of S_1_ to carry out disease resistance identification of seedlings in indoor nutrition bowls（Fig.5）, because S_2_ material is not only a large number, but also an ideal separation group with rich morphological diversity .In the next step, we will plant S_2_ in a specific field nursery, and analyze and compare the differences in disease resistance of different S_2_ isolated plants.
4. For the identification and analysis of F_1_ true or false hybrids, when doing cytological analysis, the chromosomes of both parents and the chromosome of F_1_ should be observed.

__Answer：We have shown the cytological observations of both parents, F_1_ and S_1_ in the article.
5. When conducting a flow cytometer test, both parents, F_1_, S_1_, and the TM-1 materials, should be tested and investigated to increase the scientificity and credibility of the test.

__Answer：We have shown the results of flow cytometer test of female parent, F_1_, S_1_ and TM-1 in this article.

6. At present, according to the taxonomic standards of the cotton genus, it has been recognized that Gossypium nelsonii Fryxell has been classified as the G3 species. So the newly synthesized double cotton germplasm should be A1A1G3G3.

__Answer：We have changed to A_1_A_1_G_3_G_3_ in the article.
7. According to the cotton genus classification and distribution research, 53-54 cotton species have been discovered and named, instead of 55.

__Answer：We have changed to 54 kinds in the article.

Reviewer #2:

The manuscript has potential for publication, i the authors have achieved good results, the discussion needs to be improved, with mention to other works, in the discussion it is necessary to mention other results found in the difference of ploidy, the use of colchicine, as well as resistance to *Verticillium dahliae*, which is one of the main objectives, however, the work has all merit for publication.

__Answer：We have added relevant content to the discussion of the article. The tetraploid S_1_ is a new germplasm obtained by chromosome doubling of the diploid F_1_ hybrid and is a polyploidy event in cotton. This not only restores fertility to sterile diploid F_1_ hybrids and the tetraploid S_1_ generation obtained showed segregation, which increases the phenotypic mutations in the species, enabling breeders to select for required traits. After the polyploid was generated, its stress resistance not only did not decrease but biotic stress resistance was maintained. For example, the preliminary examination showed that the disease resistance of the tetraploid S_2_ was 1 grade better than the female parent (*G. herbaceum*) and the same level as the male parent (*G. nelsonii*), and its disease resistance inheritance was closer to the male parent. In addition, the fiber quality of the tetraploid S_2_ exceeded that of both parents. From this we can see that diploid F_1_ hybrid polyploidy has important significance for cotton evolution, as the genome structure changed and the species genetic diversity enriched as well as the species adaptability and fiber quality were improved.

Reviewer #3:

WHAT THE AUTHORS NEED TO DO FOR IMPROVE THEIR PAPER• I THEORIZED THAT YOU MUST FOLLOW A LANGUGE EDITING FIRSTLY AND AFTERWARDS WE COULD CAPABLE TO FOLLOW DETAILED CORRECTIONS AND SUGGESTIONS.• I AM SORRY BUT THERE MAINY TERMS AND SENTENCES THAT MUST BE REWRITE USING BETTER TERMINOLOGY.ALL THE SENTENCES, TERMS AND WORDS UNDERLINED INTO THE TEXT BY YELLOW COLOUR SHOULD BE CHANGED.• PLEASE FOLLOW A LANGUAGE EDITIND FROM A PERSON WHO HAS ENGLISH AS NATIVE LANGUAGE BECAUSE YOU LOOSE MUCH OF THE VALUE OF YOUR WORK .
PLEASE SEE CAREFULLY OUR ADVICES INTO THE FILE COMMENTS & SUGGESTIONS (1)

__Answer：We have made changes to the questions raised by the review experts.
