# Supplementary material for: Morphological description of a novel synthetic allotetraploid(A1A1G3G3) of Gossypium herbaceum L.and G.nelsonii Fryx. suitable for disease-resistant breeding applications
Source: PLoS One. 2020 Dec 3;15(12):e0242620. doi: 10.1371/journal.pone.0242620 (PMC7714114; doi:10.1371/journal.pone.0242620)
Supplement: Attachment — Submitted filename: Response to Reviewers.doc [file pone.0242620.s008.doc]

Response to Reviewers

Reviewer #1: The author's concept of the distinction between wild cotton and cultivated cotton is vague. The content of the survey on qualitative and quantitative traits is unclear. The author is confused about the genomic properties of Gossypium. No substantial changes were given to all amendment proposals.

Answer question 1_ The *Gossypium* genus includes 54 species, of which 50 are wild cotton species and 4 are cultivated cotton species including 2 diploid and 2 tetraploid, namely diploid *Gossypium herbaceum* L. and *Gossypium arboreum* L., and tetraploid *G. hirsutum* L. and *G. barbadense* L*.* [1, 2].It is reported that diploid Gossypium herbaceum L. and Gossypium arboreum L. are classified as cultivated cotton, according to Zaitzev's classification method and named by Linnaeus. Modern discussion on the classification of cotton (Hutchinson, 1947; Mauer, 1954; Fryxell, 1969) are based on Zaitzev's classification method. Due to diploid *Gossypium herbaceum* L. and *Gossypium arboreum* L. is inferior to tetraploid cotton in terms of fiber length and quality, and has been gradually replaced by tetraploid *G. hirsutum* L. and *G. barbadense* L. At present, it is well known that tetraploid *G. hirsutum* L. and *G. barbadense* L. are used for the production and application of cultivated cotton.

1.Fryxell PA. A revised taxonomic interpretation of *Gossypium* L.(Malvaceae). Rheedea. 1992;2(2):108-165.

2.Wang KB, Wendel JF, Hua JP. Designations for individual genomes and chromosomes in Gossypium. J Cotton Res. 2018;1:3.

3.Wendel, JF. New world tetraploid cottons contain old world cytoplasm. *Proc. Natl Acad. Sci. USA* 86, 4132–4136 (1989).

4.Wendel JF, Brubaker CL & Seelanan T. The origin and evolution of *Gossypium*. in *Physiology of Cotton* (eds. Stewart, J. M. et al.) 1–18 (Springer Netherlands, Houten, the Netherlands, 2010).

Answer question 2_The primary purpose of this article is whether to obtain an F_1_ interspecific hybrid, and whether the chromosome of the F_1_ was successfully doubled into a fertile tetraploid S_1_ or not. The F_1_ interspecific hybrid was infertile, but a new tetraploid cotton S_1_ was fertile and suitable for disease-resistant breeding applications. Related to the content of the survey on qualitative and quantitative traits about F_1_ and S_1_. The morphological traits of the F_1_ plants(S_1_) showed classical hybrid characteristicsaccording to the 14–item (25-item) investigation data analysis. The qualitative and quantitative morphological characteristics of F_1_ and S_1_ are described in the results, according to the overall structure of the article, quantitative descriptions are made for quantitative traits, and qualitative analysis is made for qualitative traits.（1）In the part of plant morphology identification, it is divided into quantitative and qualitative descriptions. The plants involved are F_1_, S_1_ and S_2_, F_1_-1 and F_1_-2 and their parents. In terms of qualitative morphological characteristics, it involves the color of the floral organs of the plant, the presence or absence of the floral basal spot, the characteristics of the bracts, the amount of plant hairs, and the boll fertility，which were traits that could be stably inherited and does not change with the external environment. In terms of quantitative morphological characteristics, it involves plant height, leaf size, bract size, bract tooth, No. of teeth，No. of lobes, No. of nectary, corolla length, No. of boll loculi, boll size, etc.（2）we describe quantitative traits (F_1_, S_1_ and parents), regarding the number of sample lanes determined by flow cytometry and the observation of the number of chromosomes in the root tip.（3）According to the statistical results of the test data in the identification of disease resistance, the disease incidence and disease index were quantitatively described (the S_2_ descendants of S_1_ and parents)(Table 5）.（4）Qualitative analysis was carried out in SSR molecular identification(F_1_, S_1_ and parents) and the investigation of the application potential of S_1_ breeding.

Qualitative indicators of the color and shape of plant biological characteristics (Table1, 3). Quantitative indicators for plant biological traits(Table 4).

Table 4. Comparison of quantifiable parameters in the S_2_ descendants of S_1_ with their parental plants

| Plants | Plant height(cm) | Internode of stem(cm) | Vegetative branches | Fruiting branches | Leaf size(length×width, cm) | Bract size (length×width, cm) | Boll size (length×width,  cm) | Corolla length(cm) | Stigma length(cm) | Length of fiber(mm) |
| --- | --- | --- | --- | --- | --- | --- | --- | --- | --- | --- |
| S_2_-1 | 156.68 | 5.18 | 5 | 13 | 8.26×9.76 | 1.58×1.10 | 3.11×2.56 | 4.35 | 1.14 | 12.86 |
| S_2_-2 | 170.00 | 5.21 | 7 | 15 | 9.23×10.81 | 1.60×1.10 | 3.26×2.65 | 4.46 | 1.15 | 11.12 |
| S_2_-3 | 146.23 | 5.06 | 5 | 14 | 9.51×10.72 | 1.50×0.98 | 2.89×2.67 | 4.14 | 1.22 | 10.46 |
| S_2_-4 | 138.17 | 4.89 | 6 | 15 | 8.62×9.92 | 1.30×0.89 | 3.05×2.38 | 3.95 | 1.21 | 12.98 |
| S_2_-5 | 129.28 | 5.07 | 5 | 12 | 9.81×10.61 | 1.25×0.76 | 2.97×2.56 | 4.28 | 1.36 | 10.56 |
| S_2_-6 | 89.86 | 4.86 | 4 | 9 | 6.33×7.50 | 1.38×0.72 | 2.84×2.80 | 2.51 | 1.28 | 9.78 |
| S_2_-7 | 70.00 | 3.16 | 4 | 7 | 5.71×6.20 | 1.3×0.72 | 2.78×2.36 | 2.46 | 0.95 | 8.23 |
| S_2_-8 | 98.56 | 3.38 | 4 | 8 | 8.21×9.80 | 1.35×0.72 | 2.75×2.58 | 2.67 | 0.89 | 7.46 |
| S_2_-9 | 158.46 | 4.78 | 5 | 42 | 8.63×10.20 | 1.35×0.68 | 3.08×2.59 | 4.39 | 1.16 | 10.95 |
| S_2_-10 | 169.76 | 4.86 | 5 | 15 | 9.90×11.00 | 1.59×1.02 | 3.19×2.98 | 4.34 | 1.35 | 10.96 |
| *G.herbaceum* | 80.00 | 3.45 | 4 | 7 | 5.21×6.18 | 1.10×1.32 | 3.56×2.87 | 2.18 | 0.85 | 17.70 |
| *G.nelsonii* | 200.00 | 4.96 | 7 | 15 | 12.86×13.98 | 1.2×0.25 | 2.31×2.06 | 3.53 | 1.48 | 5.60 |

Table 5. Identification of cotton Verticillium wilt resistance in the S_2_ descendants of S_1_ with their parental plants.

| Cultivar | Disease index | Relative resistance index | Classification |
| --- | --- | --- | --- |
|  |  |  |  |
| *Jimian11* | 44.00±3.62 | 22.00±1.81 | S |
| S_2_-1 | 0.00 | 0.00 | I |
| S_2_-2 | 0.00 | 0.00 | I |
| S_2_-3 | 0.28±0.68 | 0.14±0.34 | HR |
| S_2_-4 | 0.00 | 0.00 | I |
| S_2_-5 | 0.00 | 0.00 | I |
| S_2_-6 | 0.56±0.12 | 0.28±0.06 | HR |
| S_2_-7 | 0.00 | 0.00 | I |
| S_2_-8 | 0.00 | 0.00 | I |
| S_2_-9 | 0.16±2.86 | 0.8±1.43 | HR |
| S_2_-10 | 0.00 | 0.00 | I |
| *G. nelsonii* Fryx. | 0.00 | 0.00 | I |
| *G. herbaceum* L. | 1.68±1.64 | 0.84±0.82 | HR |

Answer question 3_According to the articles(Wang KB, 2018; Wendel, J. F.,2010) related to *Gossypium* *herbaceum* L. (Genome group A, Individual genome A_1_) and *Gossypium* *nelsonii* Fryx. (Genome group G, Individual genome G_3_)(Table 1, Table 2), the genus is divided into 8 diploid genome groups (A through G, and K), as well as one allopolyploid clade (AD genome) formed from ancient merger and chromosome doubling from A and D genome ancestors. These genome groups were initially defined based on comparative chromosome sizes and chromosome behavior in interspecific hybrids (Beasley 1942; Stephens 1947;Phillips 1966; Fryxell 1992; Endrizzi et al. 1985).

1.Wang KB, Wendel JF, Hua JP. Designations for individual genomes and chromosomes in Gossypium. J Cotton Res. 2018;1:3.

2.Wendel JF, Brubaker CL & Seelanan T. The origin and evolution of *Gossypium*. in *Physiology of Cotton* (eds. Stewart, J. M. et al.) 1–18 (Springer Netherlands, Houten, the Netherlands, 2010).

Reviewer #2: (No Response)

Reviewer #3: GENERAL COMMENT
Although the authors incorporated the suggestions from the reviewers, I feel that this paper must be improved, by English Editing and a careful extension of discussion with mention to other research works and results found in international references.

Answer question _ This paper has been edited to ensure that the language is clear and free of errors. The logical presentation of ideas and the structure of the paper were also checked during the editing process. The edit was performed by professional editors at Editage, a division of Cactus Communications.

Answer question _ Added relevant research results published internationally in the discussion section of the article.

We have already obtained an interspecific hybrid F_1_ of *G. herbaceum* and *G. raimondii*. The F_1_ plants are sterile in the field, leading to various methods being attempted to double their chromosome but without success [21]. In this study, we successfully obtained an infertile interspecific F_1_ hybrid and managed to overcome the genetic incompatibility barriers between two distantly related species. Furthermore,We doubled the chromosomal content of this F_1_ hybrid throug colchicine immersion, overcoming the considerable challenges of interspecific hybrid sterility, to successfully synthesize a new S_1_ tetraploid cotton species.

21. Wu YX, Chen D, Zhu SJ, Zhang LF, Li LJ. A new sythetic hybrid (A_1_D_5_) between *Gossypium herbaceum* and *G. raimondii* and its morphological, cytogenetic, molecular characterization. [PLoS One](https://www.ncbi.nlm.nih.gov/pmc/articles/PMC5302248/). 2017; 12(2): e0169833. doi:10.1371/journal.pone.0169833.

In the present study, SSR detection revealed the presence of new specific bands in the hybrids that were not detected in the parents, indicating that new combinations or mutations of chromosomes are present in the interspecific progeny (Genomic A and Genomic C). This result might be due to chromosomal rearrangements in this newly formed hybrid [37], which was consistent with the previous reports on trispecific hybrid [20].

37. Kashkush K, Feldman M, Levy AA. Gene loss, silencing and activation in a newly synthesized wheat allotetraploid. Genetics. 2002;160(4):1651-1659.

20. Chen D, Wu YX, Zhang XL, Li FG. Analysis of [*Gossypium capitis-viridis*×(*G.hirsutum*× *G.australe*)^2^] trispecific hybrid and selected characteristics. [PLoS One](https://www.ncbi.nlm.nih.gov/pmc/articles/PMC5302248/). 2015;10(6):e0127023. doi:10.1371/journal.pone.0127023.

The preliminary examination showed that the disease resistance of the tetraploid S_2_ was better than *G. herbaceum* and the same level as *G. nelsonii*.Increased disease resistance also occurs in interspecific hybrids between *G.hirsutum* × *G. arboretum* [19].

19. Chen Y, Wang YY, Zhao T, Yang JW, Feng SL, Nazeer W, et al. A new synthetic amphiploid (AADDAA) between *G. hirsutum* and *G.arboreum* lays the foundation for transferring resistances to verticillium and drought. PLoS One. 2015;10(6):e0128981. doi:10.1371/journal.pone.0128981.

The sterility of F_1_-1 hybrid plants was consistent with the results of interspecific hybrids of *G. hirsutum* L. and *G. tomentosum* Nutt.[38], which may be caused by the incoordination of physiological processes in the hybrid offspring.

38. Guo WZ，Peng ST. Studlies on the morphogenetics and cytogenetics of interspecific hybrids of *G. hirsutum L.* (4x) and *G. tomentosum* Nutt. (4x). Acta Gossypii Sinica. 1997;9（1）:21-24.

I propose some differentiation in the subtitles and in titles of the tables (please find STIKCY NOTES in the manuscript).
(e.g p.p. 173 A title : Root tip chromosome observations changed as KARYOTYPE ANALYSIS)

All new suggestions and corrections are indicated by Highlight Notes in the Manuscript (please change all the proposed words or terms or terminology as proposed in the NOTE BOX).

Answer question _ According to the prompts of STIKCY NOTES, corresponding changes have been made in the article.
NOTICE: Micronaire value up to 6, is not a desirable trait for fiber quality.
Please not confuse the reader.
THE RESULTS FROM THIS RESEARCH ARE VERY IMPORTANT.
The researchers offer lot of work onto these experiments but is important to clarify the useful data and give the most significant conclusions in a clear way.

Answer question _ Considering the role of the micronaire value of cotton fiber in the article, it is not our purposes, we have deleted it.

PLEASE WRITE THE CONCLUSION
