# Supplementary material for: Morphological description of a novel synthetic allotetraploid(A1A1G3G3) of Gossypium herbaceum L.and G.nelsonii Fryx. suitable for disease-resistant breeding applications
Source: PLoS One. 2020 Dec 3;15(12):e0242620. doi: 10.1371/journal.pone.0242620 (PMC7714114; doi:10.1371/journal.pone.0242620)
Supplement: Attachment — Submitted filename: Response to Reviewers.doc [file pone.0242620.s011.doc]

Response to Reviewers

6. Review Comments to the Author

Please use the space provided to explain your answers to the questions above. You may also include additional comments for the author, including concerns about dual publication, research ethics, or publication ethics. (Please upload your review as an attachment if it exceeds 20,000 characters)

Reviewer #3: ALL CORRECTIONS AND SUGGESTIONS INCLUDED IN THE ATTACHED FILES (I) (Full Text with Suggestions )
AND (II) (Reviewer Comments)

Answer question 1_ We are lucky enough to meet you as our review expert. We carefully checked the language of the manuscript according to the comments of Reviewer 3 and the sticky notes in the pdf file to ensure the rationality of the article structure. The concerns raised by the reviewers have been addressed and added related references in our revised manuscript. In the process of revising the article, thank you for your patient review again and again in line with the realistic attitudes, respecting the originality of science. Let us deeply feel the precision of your words in place, increase our knowledge. These valuable experiences will be remembered for us forever.Thanks again.

Answer question 2_ added related references in our revised manuscript.

The above results indicate that the S_2_ generation underwent intense segregation in both morphological characteristics and fertility. This phenomenon exists in varying degrees in other interspecific hybrids [28] and is common in segregated generations [7]. However, it was particularly prominent in the S_2_ generation of this hybrid.

28. [Saitwal V M,](https://xueshu.baidu.com/s?wd=author%3A(Saitwal%2C V. M.) &tn=SE_baiduxueshu_c1gjeupa&ie=utf-8&sc_f_para=sc_hilight%3Dperson) [Mehetre SS,](https://xueshu.baidu.com/s?wd=author%3A(Mehetre%2C S. S.) &tn=SE_baiduxueshu_c1gjeupa&ie=utf-8&sc_f_para=sc_hilight%3Dperson) [Sonone A H,](https://xueshu.baidu.com/s?wd=author%3A(Sonone%2C A. H.) &tn=SE_baiduxueshu_c1gjeupa&ie=utf-8&sc_f_para=sc_hilight%3Dperson) [Gawande VL.](https://xueshu.baidu.com/s?wd=author%3A(Gawande%2C V. L.) &tn=SE_baiduxueshu_c1gjeupa&ie=utf-8&sc_f_para=sc_hilight%3Dperson) [Cytomorphology of F_1_, F_2_, Backcross Generations of Interspecific Cross Between *Gossypium arboreum*×*Gossypium anomalum*](http://www.researchgate.net/publication/247836906_Cytomorphology_of_F1_F2_Backcross_Generations_of_Interspecific_Cross_Between_Gossypium_arboreumGossypium_anomalum). Cytologia. 2003; 68:317-327.
https://doi.org/10.1508/cytologia.68.317

7. Wendel JF, Cronn RC. Polyploidy and the evolutionary history of cotton. Advances in Agronomy. 2003; 78:139-186.
